# Effects of cropping, smoothing, triangle count, and mesh resolution on 6 dental topographic metrics

**DOI:** 10.1371/journal.pone.0216229

**Published:** 2019-05-06

**Authors:** Michael A. Berthaume, Julia Winchester, Kornelius Kupczik

**Affiliations:** 1 Department of Bioengineering, Imperial College London, London, United Kingdom; 2 Max Planck Weizmann Center for Integrative Archaeology and Anthropology, Max Planck Institute for Evolutionary Anthropology, Leipzig, Saxony, Germany; 3 Department of Anthropology, Durham University, Durham, United Kingdom; 4 Department of Evolutionary Anthropology, Duke University, Durham, North Carolina, United States of America; Monash University, AUSTRALIA

## Abstract

Dental topography is a widely used method for quantifying dental morphology and inferring dietary ecology in animals. Differences in methodology have brought into question the comparability of different studies. Using primate mandibular second molars, we investigated the effects of mesh preparation parameters smoothing, cropping, and triangle count/mesh resolution (herein, resolution) on six topographic variables (Dirichlet normal energy, DNE; orientation patch count rotated, OPCR; relief index, RFI; ambient occlusion, portion de ciel visible, PCV; enamel surface area, SA; tooth size) to determine the effects of smoothing, cropping, and triangle count/resolution on topographic values and the relationship between these values and diet. All topographic metrics are sensitive to smoothing, cropping method, and triangle count/resolution. In general, smoothing decreased DNE, OPCR, RFI, and SA, increased PCV, and had no predictable effect on tooth size. Relative to the basin cut off (BCO) cropping method, the entire enamel cap (EEC) method increased RFI, SA, and size, and had no predictable effect on DNE and OPCR. Smoothing and cropping affected DNE/OPCR and surfaces with low triangle counts more than other metrics and surfaces with high triangle counts. There was a positive correlation between DNE/OPCR and triangle count/resolution, and the rate of increase was weakly correlated to diet. PCV tended to converge or decrease with increases in triangle count/resolution, and RFI, SA, and size converged. Finally, there appears to be no optimal triangle count or resolution for predicting diet from this sample, and constant triangle count appeared to perform better than constant resolution for predicting diet.

## Introduction

Dental ecology, the study of interactions between an organism’s teeth and its environment, provides a link between teeth, diet, and behavior [1]. As teeth are the most common element of the fossil record, dental morphology has been a primary way of inferring ecology and behavior of extinct mammals (e.g., [2–4]). Advances in scanning methodologies and computer technologies have led to the development of new methods for quantifying dental morphology to better understand dental ecology, with a focus on correlating postcanine mammalian tooth shape and diet [5,6].

The relationship between tooth shape and diet is particularly strong in mammals, which chew their food prior to ingestion [7,8]. Mammals chew foods to increase their surface area to volume ratio and mix food particles with saliva, increasing swallowability and digestibility [8,9]. Chewing ability is often quantified using chewing efficiency, which measures particle size of foods after they are chewed [10]. Kay and Sheine were able to show how one aspect of postcanine tooth shape, relative shearing crest length, was positively correlated to chewing efficiency in small mammals [11–13]. This is because relatively longer shearing crests increase the cutting ability of a tooth. A derivative of this metric has been shown to be correlated to reproductive success in lemurs [14], and several researchers have hypothesized how this and other aspects of tooth shape may relate to dietary mechanical properties (e.g. [8,9,15–19]). This and related work have shown that, in primates, shearing crest length is longer in insectivores/folivores compared to omnivores/frugivores/hard object feeders [20–24]. This is because the calorically poor and difficult to digest insect chitin and plant fiber need to be chewed more completely. In omnivores, frugivores, and hard object feeders, other selective pressures (e.g., crushing ability, reduced risk of enamel fracture, food item stabilization) may be more important [13,16,20,22,25–28]. This is supported by a lack of correlation between hard food item fracture efficiency and hypothetical molars [19].

While highly successful, quantifying relative shearing crest length has two issues: it can only be performed on teeth with preserved shearing crests. This is problematic for worn teeth, and species without prominent shearing crests (e.g. *Daubentonia madagascariensis*) [20,23]. This problem is circumvented through the use of dental topographic methods [29]. Dental topography is a landmark free method of quantifying whole tooth shape, often with a single metric. Originally methodologically limited to the use of Geographic information systems (GIS), non-GIS metrics now exist [5,6,19,23,30–32].

### Dental topographic metrics

Four topographic metrics will be focused on here: Dirichlet normal energy (DNE), orientation patch count rotated (OPCR), relief index (RFI), and ambient occlusion (portion de ciel visible, PCV). DNE was first introduced by [23] as a measure for tooth curviness. Since curvier teeth are sharper, DNE can be used as a measure for tooth sharpness. Orientation patch count (OPC), a variant of OPCR, measures dental complexity. Originally, OPC was measured by laying a 2.5D grid on the surface of teeth/tooth rows, giving each dental surface the same number of data rows and roughly the same number of polygons. The normal vector to each polygon was calculated, and polygons were binned based on the direction the normal vector pointed: anteriorly, anterior-medially, medially, etc. If three or more polygons with common edges were in the same bin, they formed a “patch.” More complex teeth had more patches [32,33]. To account for differences in scan orientation, OPCR was developed [34], which averages eight OPC values for eight tooth orientations.

Mathematically, RFI is the ratio of a tooth’s 3D surface area (herein, surface area) to 2D projected area, where projected area is tooth size [31]. Depending on the cropping method used (see below), RFI can either reflect relative tooth or cusp area. (Note, when the basin cropping method is used, some authors refer to RFI as occlusal relief, OR [24,35,36].) Relatively taller teeth/cusps have higher RFI values. RFI can be reported as a strict ratio, or the natural log of the square root of the ratio (see Materials and Methods). In general, primates with insectivorous and folivorous diets have relatively taller teeth with taller cusps and higher RFI [20,31].

Ambient occlusion, quantified through PCV, is the newest metric considered here, and has only recently been shown to be correlated to diet in primates: the claims made about PCV throughout this manuscript are tested and defended in [37]. Ambient occlusion is a visualization method used to determine how light should fall on a digital object, making it look more realistic. Depending on which direction the light is coming from, different parts of an object can be more or less exposed to the light. For example, if the light is shining down from the sun onto a mountain range at high noon, mountaintops and valleys would both be highly exposed. But if light is coming from the horizon, as the sun is rising or setting, mountaintops would be more exposed than valleys. Portion de ciel visible, or PCV, is a method for calculating ambient occlusion in CloudCompare (http://www.danielgm.net/cc/), freely available 3D point cloud processing software. When a tooth is positioned so the occlusal surface is pointed in the positive z-direction, and light is shining down from the positive z-direction, areas of the tooth that are more exposed to wear, such as cusps and crests, have relative higher PCV values than areas that are less exposed to wear, such as basins or fissures. Areas of a tooth that are more exposed have an increased chance of contacting the bolus/occluding tooth during a masticatory cycle, and more likely to experience wear. When PCV values are averaged over the entire tooth, teeth that are shaped in a manner that efficiently resists wear during a masticatory cycle have lower average PCV values than teeth that are shaped in a manner that ineffectively resists wear. As such, PCV is suggested to be a measure of morphological wear resistance [19,37,38].

### Technical considerations

To perform dental topographic analyses, 2.5D or 3D scans of teeth or dental molds are acquired, commonly using tactile, laser, white light, or microcomputed tomography (microCT) scanners, at resolutions between 10–100 μm [14,31,39–41], and scans are postprocessed. This often involves some level of segmentation/processing to produce a triangular mesh representing the surface of a tooth, which is cropped, resampled, and smoothed [31,42].

#### Cropping

In preparing surfaces, a region of interest is selected. The process of isolating this region is referred to as cropping. The two most popular methods involve 1) isolating the portion of the enamel cap superior to the lowest point on the central basin (herein, basin cut off, BCO), and 2) including the whole, outer surface of the enamel cap (herein, entire enamel cap, EEC). The first dental topographic studies employed 2.5D scanning methods, meaning the entire dental crown was not imaged [6,29]: this led to the development of the BCO cropping method, which isolated the occlusal surface of the tooth [35,43]. However, a later study investigating RFI stated, “some taxa exhibit such deep basins and such mesially-inclined crown-root margins that using this landmark (the lowest point on the basin) would have resulted in the inclusion of a substantial portion of the roots,” (pg. 1121, [31]), and the BCO method could not be used. [31] suggested using the EEC, producing a value for whole tooth shape. Later studies with larger datasets (n> 40) have showed the two cropping methods produce significantly different results for all metrics considered (i.e., DNE, OPCR, RFI, and PCV) [38,44].

#### Resampling: Triangle count

After surfaces are cropped, they are often resampled. Some dental topographic values, such as DNE and OPC(R) appear to be sensitive to triangle count, while others, like RFI and PCV, appear to be relatively insensitive to triangle count [44–47]. The simplest way of thinking about DNE and OPC(R) are the quantification of changes in angles between adjacent triangles. The current formulation for DNE sums these changes. For OPC(R), changes in angles can change the bin the triangles fall into, potentially changing the number of patches that are summed. In addition, more triangles increase the potential number of patches, meaning both metrics can be sensitive to the total number of triangles (aka, triangle count; [5,23,32,44,45,48]). At high enough mesh resolutions (herein, resolution), DNE is independent of triangle count for objects lacking edges, such as hemispheres, as long as the surface does not become more “bumpy” and therefore curvier as triangle count increases [47]. DNE is not theoretically independent of triangle count for objects with edges [47], however, like boxes or teeth with shearing crests, as there will be an increasing number of sharp edges being considered in the DNE calculation.

Once a high enough triangle count has been reached, RFI should be unaffected by triangle count, as it will only cause small changes to surface area and tooth size [46]. Three studies have used PCV to quantify tooth shape [19,37,38], and the effects of resampling have not been formally investigated. Once a high enough resolution is reached to properly represent the surface, exposure to ambient light should not be affected by triangle count, as an increase in triangle count should not cause more/less portions of the tooth to be hidden by ambient light.

#### Resampling: Resolution

Usually, teeth are resampled to a constant triangle count, as is done with DNE and OPCR, but this is potentially problematic. Larger teeth have lots of features, sometimes more than smaller teeth [49], and may require a larger number of triangles for the tooth to be fully represented. Simplifying to a lower triangle count runs a higher risk of erasing occlusal features on larger teeth compared to smaller ones, as larger teeth can have more features compared to smaller ones. The erasure of features in large teeth was addressed in a study investigating the evolution of dental complexity in the horse lineage, where it was shown teeth represented by more triangles were more complex (family Equidae [45]). When scans were overly simplified, results showed no temporal change in complexity, but at higher triangle counts, a pattern of increasing in dental complexity over the past 60 million years emerged [45].

This problem can be addressed by increasing triangle counts for all teeth, but this is met with another set of problems. First, small teeth (e.g., *Microcebus*) and teeth scanned with lower resolution scanners (e.g. the NextEngine scanner, http://www.nextengine.com/) have a lower maximum triangle count, which may be lower than the desired triangle count for larger teeth [41]. And triangle count cannot simply be increased by subdividing the surface, as this threatens to bring about a phenomenon known as the Coastline Paradox because of the fractal nature of the tooth’s surface. (The paradox states the length of a coastline is not well-defined but depends on the scale used to measure it. At higher resolutions, the curves of the coastline are better defined, but also measure small features previously ignored (e.g., large boulders on the shore), leading to an increase in shore length. In teeth, such features may be the pits/scratches used in microwear analyses.) Second, extremely high resolution scans can easily contain singularities (e.g., bumps or spikes) that sometimes cannot be fully erased with smoothing. And third, how would the optimal triangle count be determined?

In DNE studies, teeth are usually simplified to 10,000 triangles, but there is no biologically based reason this number was chosen [20,23]. [50] found 10,000 triangles was too low a triangle count for great ape molars, and calculated DNE with 20,000. Other studies using different dental topographic metrics simplified surfaces to 22,000 and 55,000 triangles [51,52]; again, these were arbitrary numbers. One study investigated the effect of triangle count on surface area and the average Euclidean distance between the decimated and original surface, and argued that when there was little change in surface area and/or distance, there is no significant change in tooth shape [46]. Shape is the geometrical information that remains when location, scale, and rotational effects are removed [53]. While changes in Euclidean distances are sometimes an effective way of quantifying changes in shape, they can also represent changes in size, as Euclidean distances can increase with scale. Changes in surface area are changes in size (scale) that may or may not reflect underlying changes in shape. This later point is evidenced by data in this paper, which shows how some dental topographic metrics (e.g. DNE and OPCR) can continue to change after surface area has converged (see Results).

Another potential solution is to analyze teeth at a constant resolution (e.g., 10 triangles/mm^2^). To the authors’ knowledge, no topographic study has held resolution, and not triangle count, constant.

#### Smoothing

After cropping and resampling, surfaces are generally smoothed to reduce noise and small features. Different programs employ different smoothing algorithms, all of which can significantly affect dental topographic values [44,47]. One dental topographic metric, DNE, is known to be sensitive to smoothing, which can be problematic when comparing results between studies [47].

### Aims of the present study

The main aims of this study are to:

1. investigate the effects of smoothing and cropping, and see if these effects change with triangle count/resolution.
2. test, separately, the relationship between tooth shape and diet at each triangle count and resolution
  - To see if this relationship changes with triangle count and resolution.
  - To see if it is better to hold triangle count or resolution constant.
3. investigate how the relationship between tooth shape and diet changes with triangle count/resolution,
  - Is there an optimal triangle count or resolution for correlating tooth shape to diet?
  - Does the change in DNE/OPCR with triangle count/resolution predict diet better than just comparing DNE/OPCR at a constant triangle count/resolution, as suggested by [45]?

Four dental topographic metrics (DNE, OPCR, RFI, and PCV) are considered, along with two size metrics (surface area and tooth size). While sometimes correlated to each other [19,20,23,38], these dental topographic metrics were chosen because they capture different aspects of the occlusal morphology: DNE measures sharpness, OPCR measures complexity, RFI measures degree of relative tooth crown height (if EEC is used) or relative cusp height (if BCO is used), and PCV measures morphological wear resistance. Because they are summative metrics that are relatively sensitive to the angles between adjacent triangles and the total number of triangles, DNE and OPCR are expected to increase with triangle count and mesh resolution. No predictions are made on the effects of cropping on DNE or OPCR, but smoothing is expected to decrease both metrics, as it reduces variation in angles between triangles [47]. At very low resolutions, RFI is expected to be highly sensitive to triangle count and mesh resolution, but at higher resolutions, when surface area (SA) and tooth size (i.e., projected tooth area) converge, RFI is expected to converge [31]. Cropping will lead to decreases in both SA and size, but is expected to affect SA more than size, and thus RFI is predicted to be higher with EEC compared to BCO. Smoothing is not expected to affect RFI at higher triangle counts/resolutions [31]. Because PCV is an average of the PCV values across the surface of the tooth, it is expected to be relatively insensitive to triangle count and resolution if there are minimal changes to tooth shape. PCV values calculated with EEC and BCO are predicted to be different, as the two cropping methods produce two different shapes on which ambient light will fall differently. Time constraints prevent us from investigating this topic. Smoothing is expected to reduce wrinkles and crevices on the surface, erasing areas that would be more hidden from ambient light, and as such increase average PCV values.

As dental topography estimates diet through tooth shape, we will investigate how this relationship is affected by triangle count, resolution, cropping, and smoothing. Shape is hypothesized to be more correlated to diet at higher triangle counts/resolutions. No hypotheses are made concerning the relationship between tooth shape and diet as cropping method and smoothing change.

## Materials and methods

Our sample consisted of 209 primate lower second molars, representing two paraphyletic groups (Prosimii and Platyrrhini), 35 genera, 54 species, and five diets. Unsmoothed surface files of the teeth were downloaded from http://morphosource.org [54], and were previously used in other dental topographic studies [20,21,31,40] (see Supplementary Information S1 Table for sample list). Information concerning the acquisition and production of the surface files can be found in [31] and [20]. The dietary classifications used in this study were taken from [20,21,31], and can be found in S1 Table, with two changes. *Galago alleni* was coded as an omnivore in [23] but was coded with the other Galagos as an insectivore in this study, and *Nycticebus javanicus*, which was coded as unknown in [23] was coded with the other *Nycticebus* as an omnivore in this study.

Downloaded tooth surface files were already cropped using the EEC cropping method, but were oriented upside-down. Surfaces were uploaded into Geomagic Studio and rotated 180° so the occlusal surface was pointed in the positive z-direction. Teeth were viewed from a lateral side and the Trim with Plane option was used to produce cropped surfaces for the BCO cropping method. This of course means EEC teeth have a higher maximum triangle count than their BCO counterparts. Surfaces were exported and saved in PLY file format.

Two datasets were produced for this study: one where surface files were standardized for triangle count and one where they were standardized for resolution. For the triangle count dataset, surface files were uploaded into AVIZO 8.1, replicated 10 times each, and replications were separately down sampled to 100, 200, 500, 1,000, 2,000, 5,000, 10,000, 20,000, 50,000, and 100,000 triangles, respectively. Each simplified surface was created from the original, unaltered surface downloaded from MorphoSource, and not from an already simplified surface (i.e. the 100,000 triangle surface was not re-simplified to create the 50,000 triangle surface). These values were chosen to encompass triangle counts lower and higher than triangle counts traditionally used [20,23,46,50–52]. Some of the smaller teeth (e.g. some *Microcebus* specimens) had triangle counts between 50,000 and 100,000, and could therefore not be simplified to 100,000 triangles.

For the resolution dataset, teeth were first cropped using either the EEC or BCO cropping method, and the surface area of the cropped surface was measured in MorphoTester [55]. Since the BCO cropping method uses only a portion of the enamel cap, but the EEC method uses the entire enamel cap, the surface area of a given tooth was always lower for BCO. The number of triangles necessary to produce a surface with a resolution of 1, 2, 5, 10, 20, 50, 100, 200, 500, and 1,000 triangles/mm^2^ was calculated, and surfaces were simplified to that number of triangles in Avizo 8.1. At a resolution of 1 triangle/mm^2^, this produced triangle counts ranging from 4 (*Microcebus griseorufus*, AMNH-174498, AMNH-174531, AMNH-174533) to 247 (*Varecia variegata*, USNM-084382) per tooth when EEC was used, and triangle counts from 2–101 when BCO was used. At a resolution of 1000 triangles/mm^2^, triangle counts ranged from 4,454–247,121 for EEC and 3,238–101,679 for BCO.

After surfaces were simplified to the desired number of triangles, surfaces were saved in PLY file format. The surfaces were then smoothed in Avizo using the smooth surface command (lambda = 0.6, iterations = 100) [31]. Smoothing must occur after simplification, otherwise the final surface will not be smooth, as decimation can create an irregular surface. In the end, 4 versions of each tooth (BCO/EEC, smoothed/unsmoothed) were produced at each triangle count/resolution, producing 76–80 versions of each tooth, and a total of 16,620 surfaces.

### Dental topographic measures

Four dental topographic metrics were taken per tooth. Three (DNE, OPCR, and RFI) were taken with MorphoTester [55]. MorphoTester outputs 5 metrics of tooth shape: DNE, RFI, OPCR, surface area, and outline area. Surface area and outlines area (herein, tooth size) are used to calculate RFI (RFI = surface area/tooth size) [55]. Here, we recalculated RFI using the reported surface area and outline area to match that of [31], using the following formula.

$$RFI\;=\;\text{ln}\;\left(sqrt(\frac{surface\;area}{tooth\;size})\right)$$

to rescale the data on RFI. Here, DNE is reported without implicit fair smoothing and with 1% outlier removal (i.e. reporting 99% in Energy x area in Outlier removal). This way of calculating DNE is referred to as DNE1 in [38]. OPCR was calculated with a minimum patch size of 3, as has been done in previous OPCR studies [20,32]. PCV was calculated using CloudCompare [56], an open source 3D point cloud and mesh processing software, and previously described methods [19]. Briefly, surfaces were uploaded and the PCV command was executed. The average PCV over the surface of the tooth was calculated using the Fit a Statistical Model to Scalar Field command.

**Fig 1:**
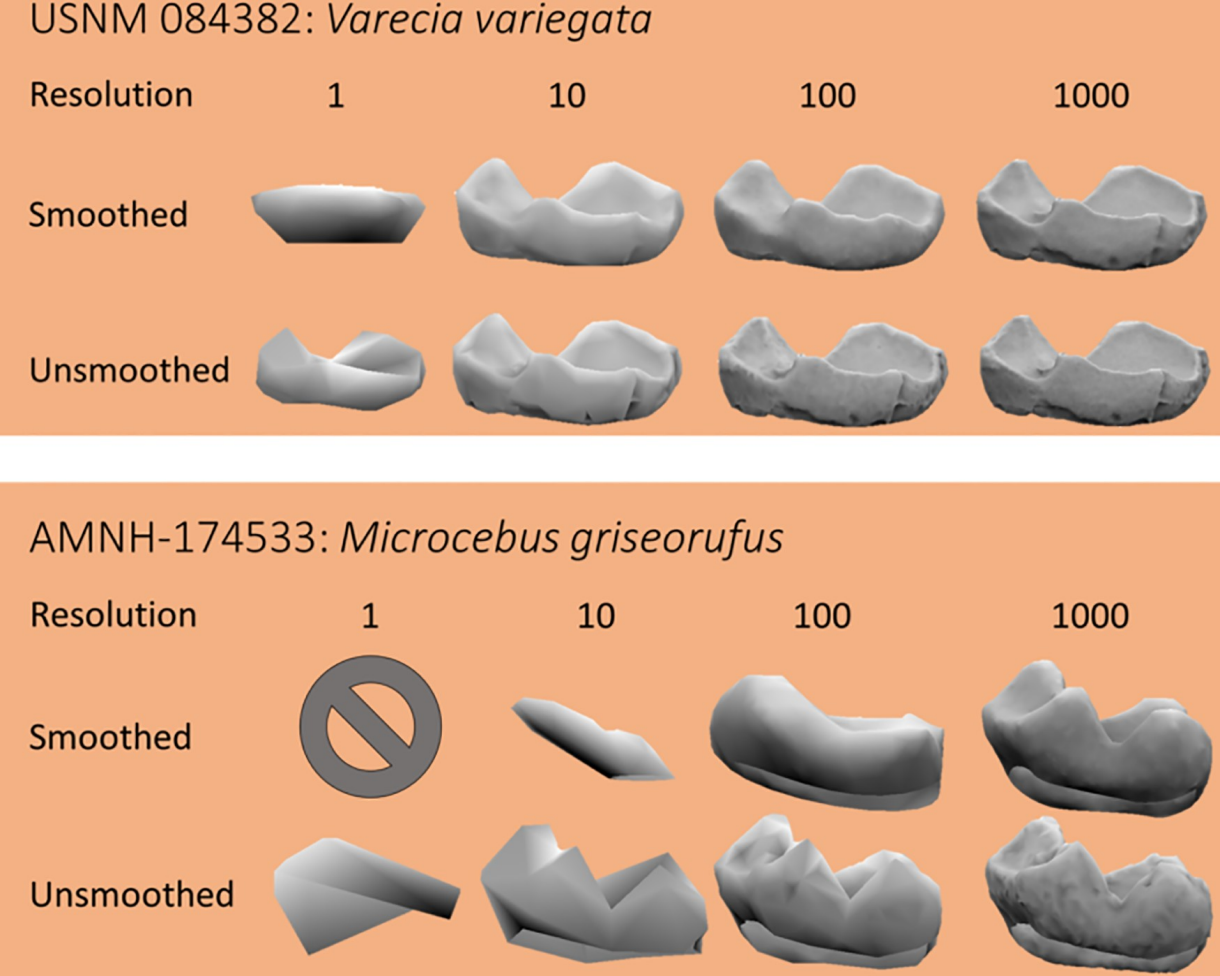
Effect of simplification and smoothing on the largest (top) and smallest (bottom) teeth used in this study, where size is quantified by surface area (SA). The cropping method shown here is EEC, and resolution is defined as triangles/mm^2^. At a resolution of 1, the *Microcebus griseorufus* tooth was composed of 4 triangles which, when smoothed, disappeared. Smoothing drastically changes tooth shape at low resolutions, but has a larger effect on smaller teeth than larger teeth. Corresponding triangle counts for the smoothed *Varecia variegata* surfaces are 247, 2471, 24712, and 247121, and for smoothed *Microcebus griseorufus* are 4, 45, 449, and 4489.

All dental topographic metrics could not be calculated for all surfaces due to program limitations and or destruction of the surface during the smoothing process (Fig 1). This was particularly true for extremely low-resolution teeth, as the effects of smoothing on these surfaces can be severe. For example, one *Nycticebus bengalensis* specimen (AMNH-183827, EEC, resolution = 1 triangle/mm, triangle count = 28) had a surface area of 0.001 and a DNE of 294.244 when the tooth was not smoothed, but during the smoothing process became oversimplified and MorphoTester reported size of 0, meaning RFI, the ratio of surface area to size, could not be calculated. When topographic analyses could not be calculated, that data point was omitted from analyses.

### Statistical analyses

Statistical analyses were performed using R v3.4.2 and RStudio v1.0.136 [57,58]. The following packages were used for data checking, analysis, and visualization: ggplot2, grid, plyr, gridExtra, and MASS [59–62].

### Statistics: Effects of smoothing and cropping

The effects of smoothing on all dental topographic metrics (DNE, OPCR, RFI, and PCV), surface area, and tooth size were investigated at different triangle counts and resolutions. Surface area and tooth size were investigated as they are used to calculate RFI and tooth size is loosely correlated with diet. The effects of smoothing were quantified by calculating the percent difference between the smoothed and unsmoothed surfaces (e.g. (DNE_smoothed-DNE_unsmoothed) / DNE_smoothed*100). Positive values indicate an increase in topographic value due to smoothing, negative, a decrease.

All data (triangle count and resolution, EEC and BCO) was pooled and divided into 4, roughly equally sized categories based on triangle count: low (< 210 triangles, n = 2101), medium-low (210–1799 triangles, n = 1973), medium-high (1800–9999 triangles, n = 1979), and high (10000 + triangles, n = 2060). This was done because surfaces with low triangle count can experience more erratic changes in shape due preprocessing methodology (c.f. [31,47]). The possible effects of clade and diet were ignored here to investigate the general effects of smoothing on metrics. In some cases, smoothed surfaces had one or more values of zero, in which case the percent difference was “infinite.” Those values were excluded from further analysis. Samples sizes were smaller for PCV, as PCV was only calculated on EEC surfaces.

Density plots were created of the percent difference in topographic values between the smoothed and unsmoothed surfaces. The probability that a percent difference was greater than zero was calculated, along with the mean, standard deviation, median, and the 2.5% and 97.5% quantiles to determine the magnitude and consistency of the effects of smoothing. The probability that a percent difference was greater than zero is the p-value indicating whether the percent difference is less than zero. For example, a probability of 0.001 indicates smoothing decreases the topographic value (p = 0.001), while a probability of 0.998 indicates smoothing increases the topographic value (p = 0.002).

The same protocol was followed to investigate the effects of cropping. Percent difference was calculated as (EEC-BCO)/EEC*100. While preprocessing the data, there were some instances where using the BCO brought the total number of triangles below 100,000. In these instances, the EEC 100,000 value was removed from the dataset for the cropping analysis, as there was no corresponding BCO value to compare it to. As PCV was not calculated for the BCO surfaces, it was not included in this analysis.

Paired Mann-Whitney U-tests were not performed to investigate differences due to smoothing and/or cropping, as they compare the central tendencies of the two groups, and we are interested in the relative magnitude of the effect smoothing and cropping has on the topographic results. This can be learned from density distributions of the percent differences, but not from Mann-Whitney U-tests.

### Statistics: Tooth shape and diet: Triangle count and resolution

In [20], the effect of phylogeny was found to be an important factor in discerning the relationship between tooth shape and diet. We investigate the effect of diet, clade, smoothing, cropping, and triangle count/resolution on dental topographic values by performing two five-way ANOVAs, one for triangle count and one for resolution. We found the interactions of nearly all factors to be significant (see Results and S2 Table), and as such completed subsequent analyses for each diet, clade (i.e., prosimian vs. platyrrhine), smoothing, cropping, and triangle count/resolution separately.

Boxplots were created to visualize the differences in dental topographic values for each diet. Separate boxplots were created for each triangle count, resolution, cropping method, and smoothing method (herein, groups) and clade. Each topographic metric was plotted separately, creating 440 boxplots. Averages and standard deviations are reported for each group individually.

One-way ANOVAs and Tukey Honest Significant Difference (HSD) tests were run for each clade and group to determine if topographic metrics varied with dietary category, and if so, how. Linear discriminant function analyses (DFAs) were run for each clade and group to investigate the ability of topographic metrics at predicting diet using the lda function, part of the MASS package, in R [61]. The prior probabilities of class membership were set to be equal for all classes, meaning it was equally likely for each tooth to be classified to each diet, and leave-one-out cross-validation was used. Although unequal sampling could lead to biased results, a main function of dental topography is to reconstruct the diets of extinct animals, for whom we do not know the diets and the prior probabilities would be equal. We aimed to mimic this situation.

As some topographic variables are correlated to one another [19,20,38], Pearson’s correlations were run between all topographic variables for each group (n = 1000 correlations). Data were not separated by clade to investigate general correlations between variables. As there is no way to compare triangle count to resolution (i.e. which triangle count corresponds to which resolution?), a qualitative comparison was made to see if triangle count or resolution tended to perform better.

### Statistics: Changes in topography as a function of triangle count and resolution

Linear plots with 95% confidence intervals were constructed of topographic metrics against the natural log of triangle count and resolution for Prosimian, Platyrrhine, EEC, BCO, smoothed and unsmoothed surfaces, separately, using ggplot2 and gridExtra [59,60]. Topographic metrics were plotted against the natural log of triangle count and resolution as triangle count and resolution were increasing exponentially (i.e. 1, 2, 5, 10, 20, 100…). It became evident that DNE and OPCR were also increasing exponentially, so the natural logs of these variables were also taken for plotting purposes.

It has been suggested that, for OPCR, the slope of OPCR vs. triangle count curve may be able to distinguish between dietary categories more accurately than the OPCR values themselves [45]. Because of this, we investigated whether slope and/or intercept of our OPCR/DNE vs. triangle count/resolution curves varied between dietary categories using ANOVAs and Tukey HSD tests, and tested the ability for slope/intercept to predict dietary categories using DFAs. To calculate the slopes and intercepts, the DNE/OPCR values for the 7 highest triangle counts and resolutions were used. The first 3 were excluded as there can be drastic changes to dental morphology at low triangle counts/resolutions due to oversimplification, thereby producing unrepresentative topographic values.

When calculating slopes, some very small teeth (e.g. *Microcebus griseorufus* AMNH-174498) had no reported changes in SA and occlusal area (size) for the 7 highest resolutions, meaning the slopes for these variables was zero, as the data produced horizontal lines. This does not imply there was no change in SA or size, but is a limitation of MorphoTester, which reports at an accuracy of 0.0005 mm^2^, regardless of tooth size or surface resolution. Because of this, and the fact that insectivores have the smallest teeth of all the primates considered here, the slope and intercept data for insectivores may be inaccurate. However, the slope and intercept would not be high, regardless, as the maximum intercept cannot be greater than the maximum SA or size, and the slope cannot be greater than that SA or size divided by the maximum resolution (assuming the intercept cannot be negative), meaning insectivores would never produce high slopes or intercepts.

ANOVAs indicate whether there are differences between categories and Tukey HSDs discern which categories are different. To visualize how the differences in dental topographic values, or lack thereof, between dietary categories varied with triangle count and resolution, the p-values indicating the probability of a difference in dental topographic values between each dietary category was plotted against the natural log of triangle count/resolution. If all comparisons between dietary categories yield a larger number of high p-values, this shows there are few differences in dental topographic values between dietary categories at that triangle count/resolution. If the comparisons yield more low p-values, this indicates there are more differences between dietary categories, and this may be a better triangle count/resolution to use for future studies. This is a qualitative, visual way of determining if there is an optimal triangle count or resolution for differentiating teeth into different dietary categories.

Since the point of dental topographic studies is not only to differentiate teeth based on diet, but to predict diet based on dental morphology, the same graphs were created for p-values from the DFAs. If there were consistently low p-values for a given triangle count or resolution, this would indicate these triangle counts and/or resolutions were better at predicting diet from tooth shape.

## Results and discussion

Raw data can be found in S1 Table. Reducing triangle count and lowering resolution can cause drastic changes in the digital representation of the tooth. These effects are exacerbated by smoothing, particularly at lower resolutions (Fig 1). Small teeth are more affected by smoothing, for a constant resolution, than larger teeth, because they are made up of fewer triangles. Smoothing appears to affect large and small teeth equally when triangle count is held constant.

### Effects of smoothing

**Fig 2:**
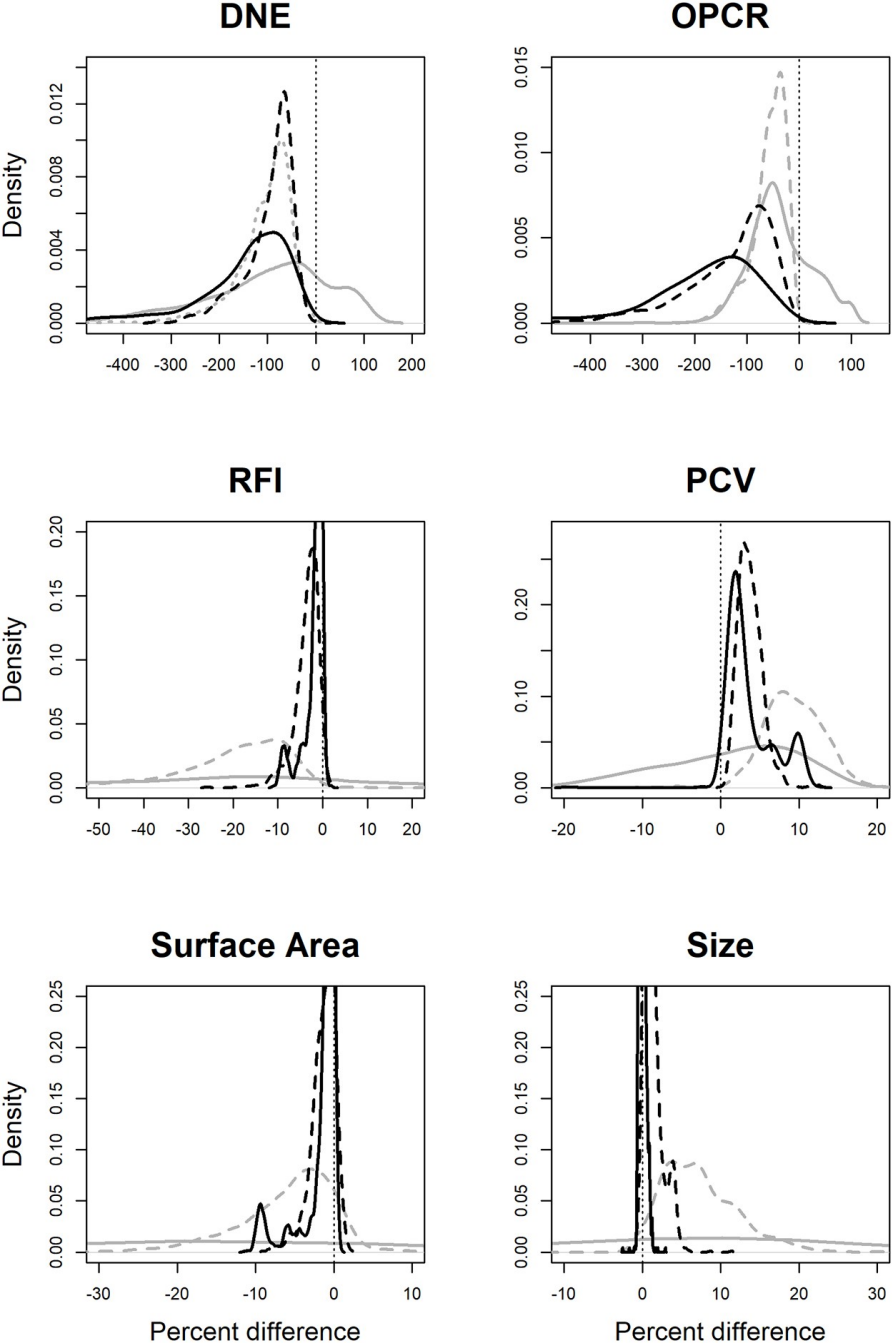
Density plots showing the effect of smoothing on DNE, OPCR, RFI, PCV, surface area (SA), and tooth size. A negative value indicates a decrease in the topographic value due to smoothing. Grey, solid lines are low triangle counts (< 210 triangles), dashed grey is medium-low triangle counts (210–1799 triangles), dashed black lines are medium-high triangle counts (1800–9999 triangles), and solid black lines are high triangle counts (10000+ triangles). For RFI, PCV, SA, and size, as triangle count increases, the effects of smoothing become more predictable, as is evidenced by the narrowing of the density distributions. DNE and OPCR are more sensitive to smoothing than the other metrics.

Smoothing affected topographic variables differently. In general, at low triangle counts (< 210), smoothing caused drastic, inconsistent changes in topographic metrics, as is evidenced by the large means/medians and wide confidence intervals (Fig 2, Table 1). This is because, at low triangle counts, the shape of the surface can change drastically with smoothing (Fig 1). Therefore, teeth with low triangle counts and that are subjected to different smoothing protocols should not be compared.

**Table 1:** Descriptive statistics for density curves in Fig 1, showing the effects of smoothing. Both mean and median are reported as the curves sometimes do not follow a normal distribution. Probabilities < 0.05 indicate a statistically significant result that smoothing decreases the topographic metric (p < 0.05), and probabilities > 0.95 indicate a statistically significant result that smoothing increases the topographic metric (p > 0.95). The 2.5% and 97.5% quantiles are given to represent the 95% confidence interval. Triangle counts of L = low (< 210), ML = medium-low (210–1799), MH = medium-high (1800–9999), and H = high (10000+). Bold p-values are significant for greater than zero, bold and italics for less than zero.

Topographic metric	Triangle count	Sample size	Probability greater than zero	Mean	Stdev	2.5% quantile	Median	97.5% quantile
DNE	L	2079	0.2097	-914.4	13627.4	-842.9	-86	93.2
	ML	2007	**0.001**	-106.9	60.4	-267.5	-92.3	-24
	MH	2019	**0.001**	-96.2	51.2	-230.4	-80.4	-33
	H	2104	**0**	-240	298.8	-1247.6	-132.4	-32.9
OPCR	L	2117	0.2518	-36.2	62.2	-140.7	-43.1	94.8
	ML	2007	**0.003**	-56.8	34.3	-147.1	-50	-11.8
	MH	2019	**0**	-133.1	87.1	-356.2	-105.7	-30.1
	H	2103	**0**	-332.3	418.3	-1686.7	-181.3	-38.7
RFI	L	2067	0.1892	-28	4585.3	-985.8	-32.6	1497.2
	ML	2007	**0.0025**	-23.4	21.4	-81.9	-17.9	-3.7
	MH	2019	**0.0367**	-3.5	2.8	-10.8	-2.9	0.2
	H	2104	0.1012	-1.8	2.3	-9	-1	0.4
PCV	L	1009	0.5857	-5.1	131.7	-18.5	2.5	14.6
	ML	1004	***0*.*993***	9.3	3.7	2.3	9.2	16.5
	MH	1030	***0*.*999***	3.8	1.6	1.4	3.6	7.3
	H	1092	***0*.*9908***	3.7	3.1	0.2	2.5	10.4
Surface area	L	2096	0.0663	-12208.1	106131.5	-56341	-22.8	5
	ML	2007	0.1375	-5.9	7	-20.7	-4.5	3.1
	MH	2019	0.1902	-1.3	1.6	-5.4	-1	1
	H	2104	0.1407	-1.6	2.5	-9.5	-0.7	0.3
Size	L	2104	0.5238	-9221.1	61256	-116109	1.7	17.4
	ML	2007	***0*.*9696***	7.2	5.8	0	6.7	18.4
	MH	2019	0.9277	1.4	1.3	-0.2	1	4.3
	H	2104	0.4658	0	0.3	-0.6	0	0.9

At high triangle counts (10000+), the effects of smoothing decrease. Relative to lower triangle counts, the mean/median percent changes approach zero and the confidence intervals decrease, indicating the effects of smoothing are smaller and more predictable. At these triangle counts, DNE and OPCR always decrease with smoothing (p = 0; Table 1). DNE and OPCR are most affected by smoothing, with the mean/median effects being a 240/132% and 332/181% decrease in DNE/OPCR, respectively (Table 1). DNE and OPCR decrease because smoothing attempts to reduce angles between triangles, causing the surface to appear less “curvy” and complex. The magnitude of change due to smoothing is equal to or much greater than the percent difference between dietary categories in primates [20]. For example, there is a 47/247% difference in mean DNE scores between insectivorous and hard object feeding platyrrhines and prosimians, respectively, and an 18/13% difference in mean OPCR scores. This means that the error associated with smoothing is equal to or greater than the difference due to diet, and DNE/OPCR data subjected to different smoothing protocols should not be directly compared. The large confidence intervals indicate an unpredictability in the change in DNE/OPCR due to smoothing, and therefore smoothed values should not be transformed to unsmoothed values, or vice versa (Fig 2, Table 1). This is in line with conclusions from a previous DNE smoothing study [47].

At a triangle count of 10000+, RFI, PCV, SA and size are all less affected by smoothing. Smoothing decreases RFI and SA in 89.88% and 85.93% of the cases, and the probabilities of an increase in RFI and SA are not negligible (p = 0.1012 and 0.1407). Smoothing attempts to reduce local shape variability, which tends to cause bumpier surfaces to become more regular and decrease SA. Since RFI is proportional to SA, this generally causes RFI to decrease as well. Small increases in surface area can occur, and changes in surface area are smaller at high triangle counts. Smoothing increases PCV (p = 0.0092), and is just as likely to cause tooth size to increase or decrease, but by a small amount (95% confidence interval: -0.6% to 0.9%).

The magnitudes of the mean/median percent changes in RFI, PCV, SA and size due to smoothing are 1.8/1%, 3.7/2.5%, 1.6/0.7%, and 0/0%. The 95% confidence intervals for all metrics are relatively small, being less than 10.5%, meaning changes in these metrics due to smoothing are small and consistent. As such, our sample suggests RFI, PCV, SA, and size values gathered using different smoothing protocols can be directly compared. Should a transformation be needed, we have provided equations for transforming smoothed values to unsmoothed ones for each cropping method, triangle count, and resolution (see S3 Table). We also provide the coefficient of correlation, *p*-value, and test statistics so the strength of the correlation can be judged for each situation. However, **we stress transformations should only be used when it is truly not possible to gather the data in a standardized manner,** and should not be used for DNE or OPCR.

As these results encompass teeth cropped using both cropping methods (EEC and BCO) and across two major primate clades (platyrrhines and prosimians), we believe they are generalizable to all primate dental topographic studies.

### Effects of cropping

**Fig 3:**
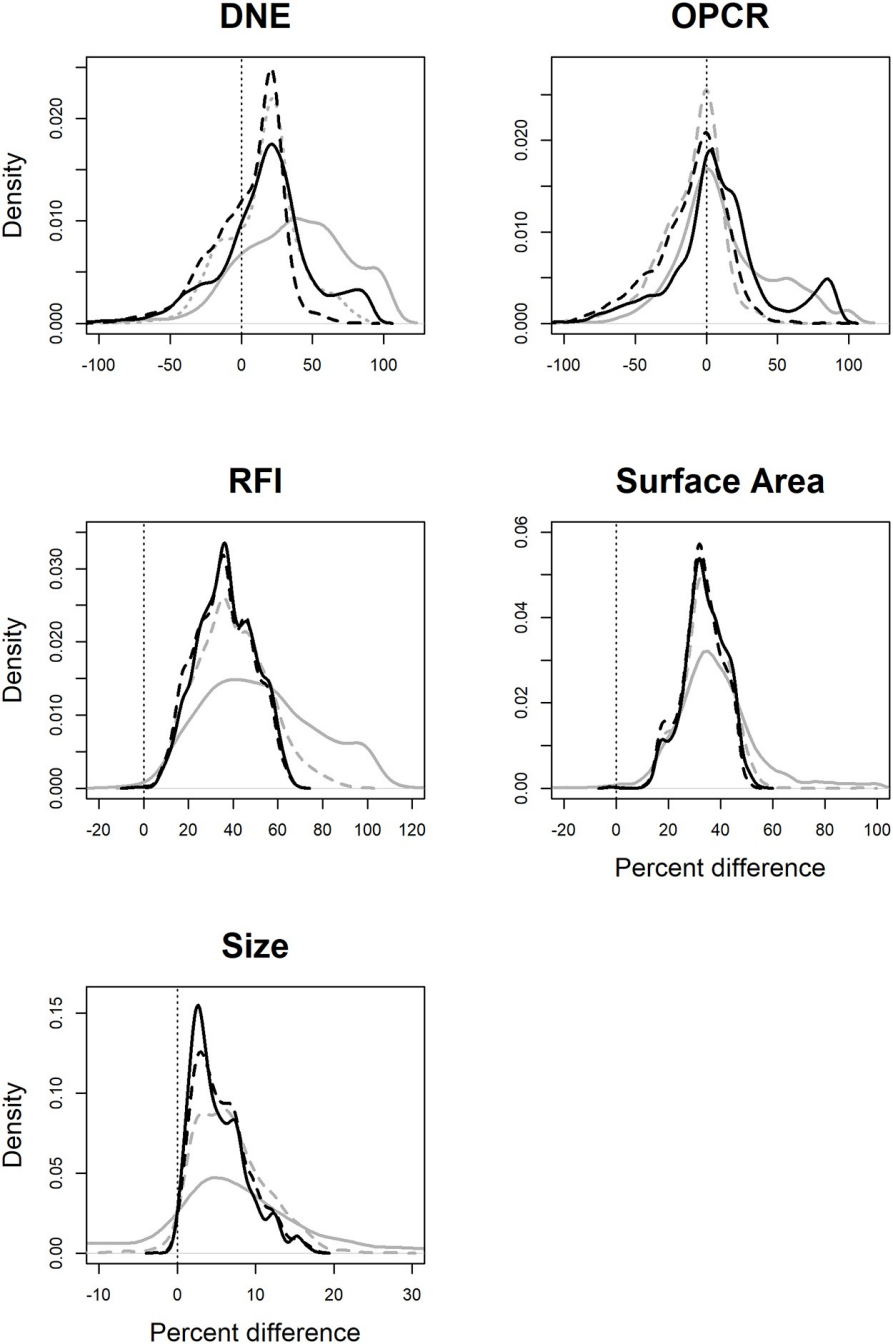
Density plots showing the effect of cropping method on DNE, OPCR, RFI, surface area (SA), and tooth size. PCV was not calculated for surfaces cropped with the BCO method due to time constraints. A negative value indicates an increase in the topographic value going from EEC to BCO. Grey, solid lines are low triangle counts (< 210 triangles), dashed grey is medium-low triangle counts (210–1799 triangles), dashed black lines are medium-high triangle counts (1800–9999 triangles), and solid black lines are high triangle counts (10000+ triangles). In general, RFI, SA, and size values are smaller with BCO compared to EEC. DNE and OPCR are just as likely to increase or decrease. This is because, for a given triangle count, there are more triangles representing the occlusal surface using the BCO method, which can increase DNE and OPCR. But for a given resolution, fewer triangles represent the tooth with the BCO than the EEC, leading to a decrease in DNE and OPCR, which are summative metrics.

As with smoothing, cropping affected topographic metrics differently. SA and tooth size were affected most at low triangle counts, but at triangle counts of 210+, the effects of triangle count were small (Table 2, Fig 3). This is because representing surfaces with such few triangles creates an unrealistic digital representation of the tooth and introduces a large level of error (Fig 1, [46]). Extreme values for SA and size at low triangle counts cause the mean to be an unrealistic representation of the average percent change.

**Table 2:** Statistics for density curves in Fig 2, showing the difference between the EEC and BCO cropping methods. Both mean and median are reported as the curves sometimes do not follow a normal distribution. Probabilities < 0.05 indicate a statistically significant result that smoothing decreases the topographic metric (p < 0.05), and probabilities > 0.95 indicate a statistically significant result that smoothing increases the topographic metric (p > 0.95). The 2.5% and 97.5% quantiles are given to represent the 95% confidence interval. Triangle counts of L = low (< 210), ML = medium-low (210–1799), MH = medium-high (1800–9999), and H = high (10000+). Bold p-values are significant for greater than zero, bold and italics for less than zero.

Topographic metric	Triangle count	Sample size	Percent greater than zero	Mean	Stdev	2.5% quantile	Median	97.5% quantile
DNE	L	2000	0.837	14.9	599.7	-50.2	38.9	100
	ML	2012	0.7381	15	27.2	-34.5	18.2	68.1
	MH	1988	0.6398	4.5	24.8	-54.5	11.2	39.8
	H	2230	0.7552	17	32.9	-53	18.7	84.8
OPCR	L	1984	0.6003	11.3	42.2	-48.2	6.4	84.9
	ML	2012	0.3434	-10.5	22.1	-64	-5.7	25.9
	MH	1988	0.3732	-10.9	25.4	-71	-5.6	29.3
	H	2229	0.6348	9	35.7	-64.9	6.2	87.4
RFI	L	1970	***0*.*9919***	51.5	32.2	10.8	49.9	100.8
	ML	2012	***0*.*9985***	39.9	15.8	12.8	38.4	74.1
	MH	1988	***0*.*9985***	35.7	12.8	12.8	35.5	58.5
	H	2230	***0*.*9991***	36.9	12.5	13.2	36.6	60
Surface area	L	1987	0.9472	-458.1	6732.9	-305.6	35.8	79.7
	ML	2012	***0*.*9985***	34.3	8.9	16.5	34.2	50.6
	MH	1988	***0*.*9985***	32.7	8	16.4	32.8	46.2
	H	2230	***0*.*9991***	33.8	8	16.9	33.7	47.6
Size	L	1993	0.7336	-784	8899.5	-1611	5.5	58.1
	ML	2012	***0*.*9707***	6.7	4.7	-0.3	6.2	16.6
	MH	1988	***0*.*9945***	5.7	3.5	0.7	5.2	13.8
	H	2230	***0*.*9991***	5.3	3.5	0.7	4.4	13.6

Both DNE and OPCR were highly affected by cropping. Regardless of triangle count, the average change due to difference in cropping methods was close to zero, with the mean/medians ranging from 4.5–38.9% for DNE and -10.9 to 11.3% for OPCR (Table 2). At high triangle counts, the 95% confidence interval were wide at -53% to 84.8% for DNE and -64.9% to 87.4% for OPCR, implying that, although the average effect is close to zero, it is not consistently so. Consistent with some previous studies [38,44], DNE and OPCR values gathered using different cropping methods cannot be compared, and their wide 95% confidence intervals suggest no acceptable transformation factor.

As the BCO method uses a subset of the triangles used by the EEC method, at higher triangle counts (210+), teeth had higher SA, sizes, and RFIs when cropped with the EEC compared to the BCO (p = 0.0009–0.0293). The 95% confidence intervals were narrower than for DNE and OPCR, being 13.2–60%, 16.9–47.6%, and 0.7–13.6% for RFI, SA and size, respectively, at triangle counts of 10000+. We do not recommend RFI and SA values gathered with the EEC and BCO cropping methods be directly compared. First, because the confidence intervals are wide, and second, when EEC is used, RFI and SA are descriptors of tooth shape and size, but when BCO are used, they are descriptors of cusp shape and size, respectively. Since they are measuring different aspects of dental morphology, they should not be directly compared. As before, we have provided transformations for all topographic variables in cases where original data cannot be accessed (S3 Table).

### Predicting diet at different triangle counts and resolutions

The two five-way ANOVAs were run investigating the effect of diet, clade, smoothing, cropping, and triangle count/resolution on the topographic metrics, one for triangle count and resolution, separately. There was a significant relationship between all individual factors and all topographic metrics, except for triangle count and size (Table 3, Table 4). Nearly all higher-level interactions were significant with DNE, OPCR, RFI and PCV, but few were with SA and size. Because of this, subsequent analyses were carried out for each group and clade, separately.

**Table 3:** Five-way ANOVA testing the influence of diet, clade, smoothing, cropping, and triangle count on dental topographic values. In the factor column, d = diet, g = group (clade), s = smoothing, cm = cropping method, tc = triangle count. F-values are given followed by p-values in parentheses. P-values of 0 are ≤0.0005. Bold and italics indicates p < 0.05.

Factor	DF	DNE	OPCR	RFI	PCV	Surface area	Size
d	4	***127*.*282 (0)***	***291*.*35 (0)***	***1479*.*95 (0)***	***143*.*589 (0)***	***1832*.*089 (0)***	***1634*.*25 (0)***
g	1	***748*.*236 (0)***	***779*.*502 (0)***	***1074*.*784 (0)***	***170*.*662 (0)***	***1457*.*093 (0)***	***778*.*898 (0)***
s	1	***4965*.*285 (0)***	***5104*.*69 (0)***	***1266*.*728 (0)***	***846*.*061 (0)***	***16*.*474 (0)***	***14*.*177 (0)***
cm	1	***1428*.*359 (0)***	***1632*.*55 (0)***	***24017*.*689 (0)***	***2139*.*23 (0)***	***1670*.*42 (0)***	***25*.*151 (0)***
tc	1	***28044*.*369 (0)***	***26495*.*271 (0)***	***761*.*158 (0)***	---	***33*.*647 (0)***	0.728 (0.394)
d x g	3	***83*.*49 (0)***	***28*.*457 (0)***	***18*.*412 (0)***	***21*.*855 (0)***	***1029*.*752 (0)***	***763*.*577 (0)***
d x s	4	***165*.*929 (0)***	***197*.*858 (0)***	***5*.*858 (0)***	0.696 (0.595)	1.013 (0.399)	0.838 (0.501)
g x s	1	***624*.*648 (0)***	***734*.*053 (0)***	2.652 (0.103)	***16*.*052 (0)***	0.624 (0.43)	0.654 (0.419)
d x cm	4	***111*.*901 (0)***	***115*.*244 (0)***	***352*.*001 (0)***	---	***82*.*613 (0)***	2.048 (0.085)
g x cm	1	***279*.*544 (0)***	***526*.*494 (0)***	***204*.*768 (0)***	---	***99*.*276 (0)***	0.081 (0.775)
s x cm	1	***1501*.*917 (0)***	***1913*.*089 (0)***	***15*.*826 (0)***	---	0.077 (0.782)	0.004 (0.951)
d x tc	4	***549*.*026 (0)***	***616*.*228 (0)***	0.729 (0.572)	***4*.*532 (0*.*001)***	2.346 (0.052)	0.789 (0.532)
g x tc	1	***2506*.*026 (0)***	***2485*.*307 (0)***	***18*.*654 (0)***	***82*.*1 (0)***	1.924 (0.165)	1.13 (0.288)
s x tc	1	***13488*.*688 (0)***	***14179*.*981 (0)***	***224*.*365 (0)***	***5*.*257 (0*.*022)***	0.361 (0.548)	***5*.*315 (0*.*021)***
cm x tc	1	***7690*.*758 (0)***	***8361*.*752 (0)***	***7*.*884 (0*.*005)***	---	***5*.*583 (0*.*018)***	0.053 (0.818)
d x g x s	3	***43*.*325 (0)***	***21*.*726 (0)***	1.462 (0.223)	***3*.*276 (0*.*02)***	0.288 (0.834)	0.311 (0.817)
d x g x cm	3	***41*.*085 (0)***	***35*.*687 (0)***	***12*.*162 (0)***	---	***41*.*04 (0)***	0.212 (0.888)
d x s x cm	4	***133*.*977 (0)***	***136*.*898 (0)***	0.629 (0.642)	---	0.007 (1)	0.008 (1)
g x s x cm	1	***340*.*5 (0)***	***558*.*468 (0)***	0.555 (0.456)	---	0.09 (0.765)	0.016 (0.899)
d x g x tc	3	***204*.*386 (0)***	***88*.*571 (0)***	1.048 (0.37)	***7*.*948 (0)***	1.439 (0.229)	0.393 (0.758)
d x s x tc	4	***528*.*5 (0)***	***408*.*586 (0)***	***7*.*25 (0)***	***9*.*875 (0)***	0.396 (0.811)	0.312 (0.87)
g x s x tc	1	***2279*.*226 (0)***	***2474*.*083 (0)***	2.545 (0.111)	***11*.*224 (0*.*001)***	0.312 (0.577)	0.267 (0.606)
d x cm x tc	4*	***483*.*212 (0)***	***360*.*633 (0)***	2.213 (0.065)	0.546 (0.651)	0.288 (0.886)	0.027 (0.999)
g x cm x tc	1	***1488*.*442 (0)***	***2187*.*61 (0)***	***7*.*293 (0*.*007)***	---	1.509 (0.219)	0.009 (0.925)
s x cm x tc	1	***7874*.*414 (0)***	***8845*.*623 (0)***	***55*.*5 (0)***	---	1.979 (0.16)	0.001 (0.977)
d x g x s x cm	3	***42*.*069 (0)***	***35*.*039 (0)***	0.027 (0.994)	---	0.007 (0.999)	0.009 (0.999)
d x g x s x tc	3	***126*.*941 (0)***	***77*.*252 (0)***	0.244 (0.866)	---	0.028 (0.994)	0.114 (0.952)
d x g x cm x tc	3	***162*.*632 (0)***	***117*.*446 (0)***	0.418 (0.74)	---	0.19 (0.903)	0.015 (0.998)
d x s x cm x tc	4	***509*.*243 (0)***	***401*.*756 (0)***	0.493 (0.741)	---	0.139 (0.968)	0.004 (1)
g x s x cm x tc	1	***1528*.*631 (0)***	***2229*.*395 (0)***	1.765 (0.184)	---	0.427 (0.513)	0 (0.996)
d x g x s x cm x tc	3	***156*.*011 (0)***	***109*.*876 (0)***	0.596 (0.618)	---	0.027 (0.994)	0.003 (1)

*degree of freedom for PCV is 3

**Table 4:** Five-way ANOVA testing the influence of diet, clade, smoothing, cropping, and resolution on dental topographic values. In the factor column, d = diet, g = group (clade), s = smoothing, cm = cropping method, r = resolution. F-values are given followed by p-values in parentheses. P-values of 0 are ≤0.0005. Bold and italics indicates p < 0.05.

Factor	DF	DNE	OPCR	RFI	PCV	Surface area	Size
d	4	***181*.*748 (0)***	***289*.*563 (0)***	***319*.*835 (0)***	***67*.*566 (0)***	***1590*.*518 (0)***	***1453*.*767 (0)***
g	1	***450*.*227 (0)***	***469*.*955 (0)***	***501*.*109 (0)***	***151*.*738 (0)***	***1323*.*522 (0)***	***728*.*294 (0)***
s	1	***1559*.*974 (0)***	***2332*.*933 (0)***	***480*.*694 (0)***	***263*.*52 (0)***	***79*.*597 (0)***	***10*.*741 (0*.*001)***
cm	1	***532*.*537 (0)***	***123*.*84 (0)***	***10470*.*333 (0)***	***1190*.*934 (0)***	***1479*.*428 (0)***	***27*.*187 (0)***
r	1	***12326*.*275 (0)***	***14984*.*379 (0)***	***431*.*841 (0)***	---	***72*.*013 (0)***	***13*.*619 (0)***
d x g	3	***49*.*083 (0)***	***102*.*496 (0)***	***9*.*5 (0)***	***3*.*136 (0*.*024)***	***928*.*815 (0)***	***701*.*657 (0)***
d x s	4	***62*.*25 (0)***	***101*.*599 (0)***	***25*.*699 (0)***	***4*.*496 (0*.*001)***	0.202 (0.938)	***2*.*432 (0*.*045)***
g x s	1	***210*.*193 (0)***	***266*.*711 (0)***	1.568 (0.211)	0.071 (0.789)	0 (0.988)	1.983 (0.159)
d x cm	4	***14*.*507 (0)***	***7*.*342 (0)***	***135*.*535 (0)***	---	***82*.*214 (0)***	1.996 (0.092)
g x cm	1	***34*.*696 (0)***	***17*.*033 (0)***	***80*.*184 (0)***	---	***103*.*511 (0)***	0 (0.987)
s x cm	1	***59*.*832 (0)***	***61*.*021 (0)***	***251*.*953 (0)***	---	0.055 (0.814)	0.365 (0.546)
d x r	4	***284*.*535 (0)***	***410*.*837 (0)***	***12*.*656 (0)***	***2*.*786 (0*.*025)***	0.185 (0.946)	0.94 (0.44)
g x r	1	***935*.*962 (0)***	***685*.*373 (0)***	0.067 (0.796)	***31*.*772 (0)***	0.46 (0.498)	0.578 (0.447)
s x r	1	***2686*.*977 (0)***	***3747*.*154 (0)***	***115*.*241 (0)***	***8*.*837 (0*.*003)***	***23*.*518 (0)***	***4*.*75 (0*.*029)***
cm x r	1	***625*.*949 (0)***	***240*.*716 (0)***	***106*.*635 (0)***	---	0.002 (0.965)	0.084 (0.772)
d x g x s	3	***34*.*597 (0)***	***53*.*405 (0)***	0.706 (0.548)	1.084 (0.354)	0.049 (0.986)	1.256 (0.288)
d x g x cm	3	1.921 (0.124)	2.517 (0.056)	***2*.*877 (0*.*035)***	---	***43*.*109 (0)***	0.252 (0.86)
d x s x cm	4	***7*.*249 (0)***	***4*.*023 (0*.*003)***	***2*.*671 (0*.*03)***	---	0.128 (0.972)	0.067 (0.992)
g x s x cm	1	***24*.*853 (0)***	***17*.*916 (0)***	0.03 (0.863)	---	0.004 (0.95)	0.001 (0.97)
d x g x r	3	***96*.*678 (0)***	***146*.*756 (0)***	0.734 (0.531)	***7*.*455 (0)***	0.014 (0.998)	0.482 (0.695)
d x s x r	4	***113*.*634 (0)***	***143*.*739 (0)***	***8*.*436 (0)***	2.116 (0.076)	0.027 (0.999)	0.942 (0.438)
g x s x r	1	***522*.*798 (0)***	***381*.*391 (0)***	0.373 (0.541)	0.004 (0.949)	0.038 (0.845)	0.82 (0.365)
d x cm x r	4*	***37*.*636 (0)***	***14*.*055 (0)***	1.302 (0.267)	0.343 (0.794)	0.041 (0.997)	0.027 (0.999)
g x cm x r	1	***89*.*096 (0)***	***30*.*216 (0)***	0.402 (0.526)	---	0.003 (0.954)	0.002 (0.963)
s x cm x r	1	***252*.*596 (0)***	***101*.*944 (0)***	***94*.*314 (0)***	---	0 (0.997)	0.163 (0.686)
d x g x s x cm	3	***6*.*638 (0)***	***4*.*024 (0*.*007)***	2.298 (0.075)	---	0.043 (0.988)	0.018 (0.997)
d x g x s x r	3	***67*.*517 (0)***	***88*.*332 (0)***	0.183 (0.908)	---	0.034 (0.992)	0.492 (0.688)
d x g x cm x r	3	***12*.*826 (0)***	***4*.*657 (0*.*003)***	1.123 (0.338)	---	0.016 (0.997)	0.006 (0.999)
d x s x cm x r	4	***14*.*292 (0)***	***5*.*796 (0)***	0.919 (0.452)	---	0.067 (0.992)	0.029 (0.998)
g x s x cm x r	1	***77*.*192 (0)***	***30*.*204 (0)***	0.071 (0.79)	---	0.024 (0.878)	0.001 (0.973)
d x g x s x cm x r	3	***9*.*998 (0)***	***6*.*996 (0)***	0.871 (0.455)	---	0.03 (0.993)	0.007 (0.999)

*degrees of freedom for PCV is 3

**Fig 4:**
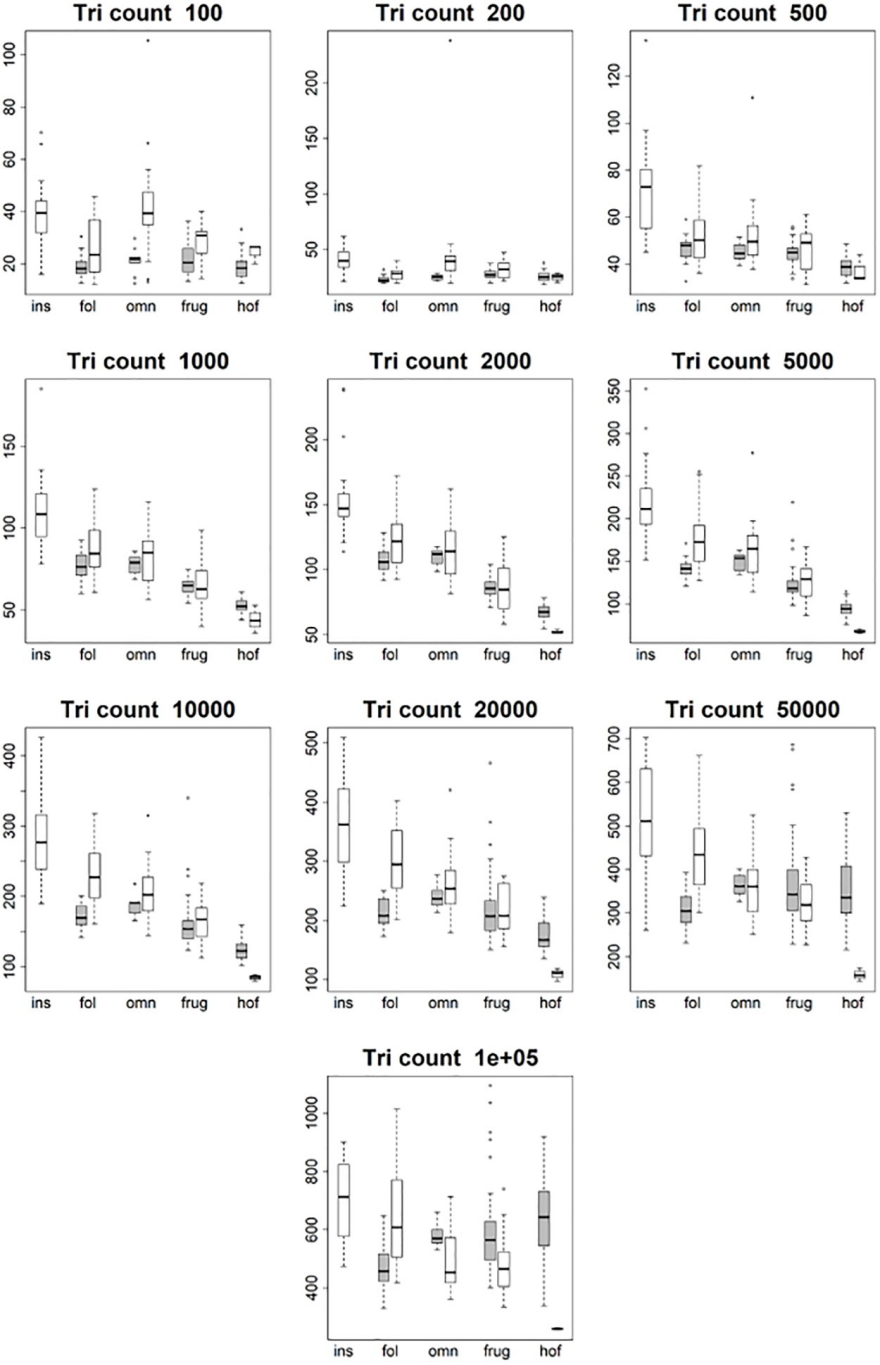
Boxplots showing prosimian (white) and platyrrhine (grey) DNE values for each diet at different triangle counts (smoothed, EEC). Diets are shown on the x-axis: ins = insectivore, fol = folivore, omn = omnivore, frug = frugivore, and hof = hard object feeder. Results at a triangle count of 10000 are comparable to those from [20]. Within prosimians, as triangle count increases, there appears to be more separation between dietary categories. At a triangle count of about 1000, the pattern of insectivores having the highest DNE, followed by folivores, omnivores, frugivores, and finally hard object feeders begins to emerge. This pattern generally holds true up until a triangle count of 100000. Within platyrrhines, there is no great distinction between folivores, omnivores, and frugivores up until a triangle count of 10000, but hard object feeders consistently have the dullest teeth (i.e. lowest DNE value). At higher resolutions, however, this changes, and at a triangle count of 100000 hard object feeders have the higher average DNE value, and folivores have the lowest. This pattern is better seen when the BCO cropping method is used, and/or surfaces are unsmoothed (see S1 Fig).

**Fig 5:**
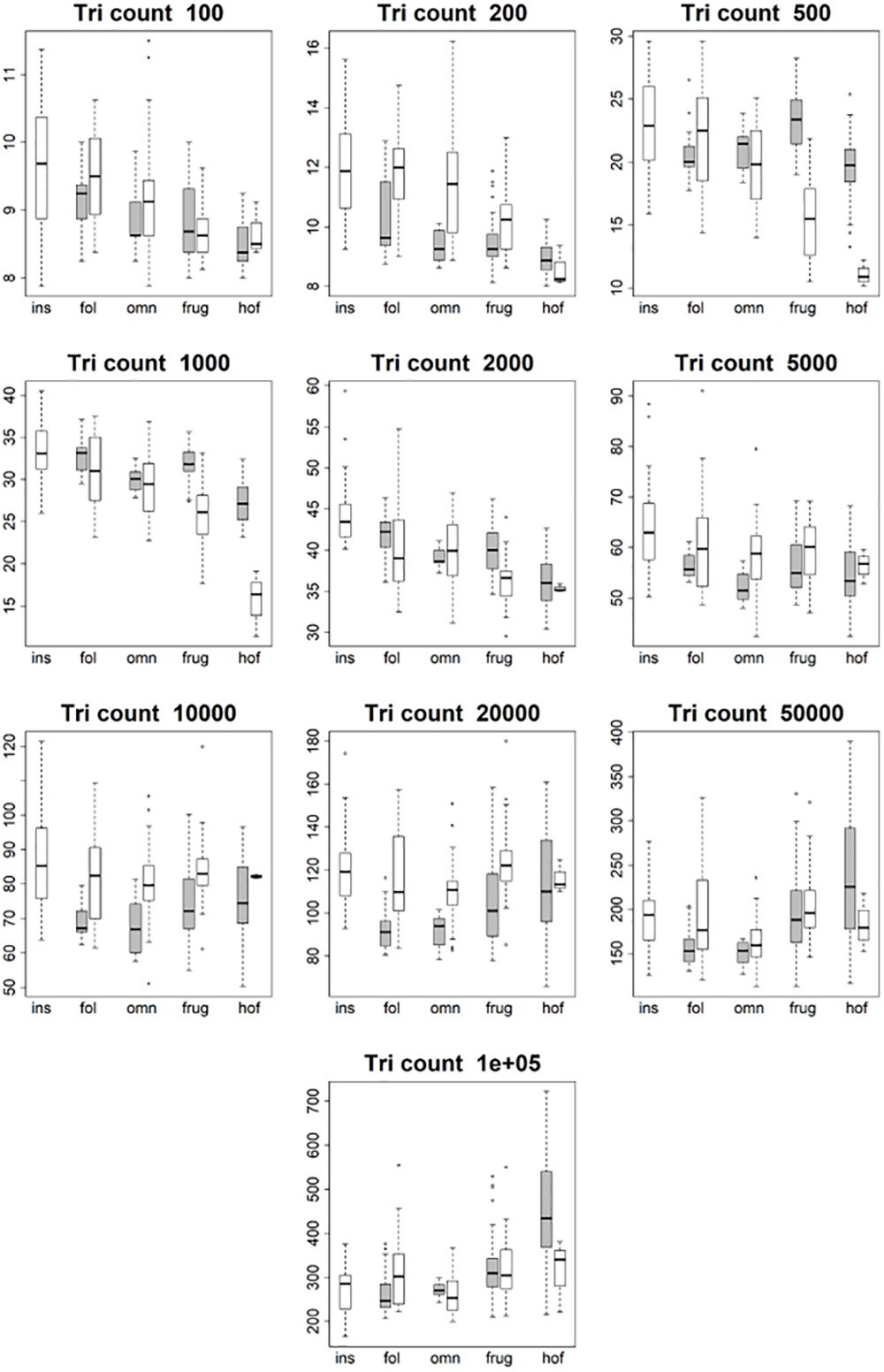
Boxplots showing prosimian (white) and platyrrhine (grey) OPCR values for each diet at different triangle counts (smoothed, EEC). Diets are shown on the x-axis: ins = insectivore, fol = folivore, omn = omnivore, frug = frugivore, and hof = hard object feeder. Results at a triangle count of 10000 are comparable to those from [20]. As with DNE, the relationship between diet and OPCR changes with resolution. E.g. hard object feeding platyrrhines have the highest OPCR value at a triangle count of 100000, but the lowest at a count of 2000.

**Fig 6:**
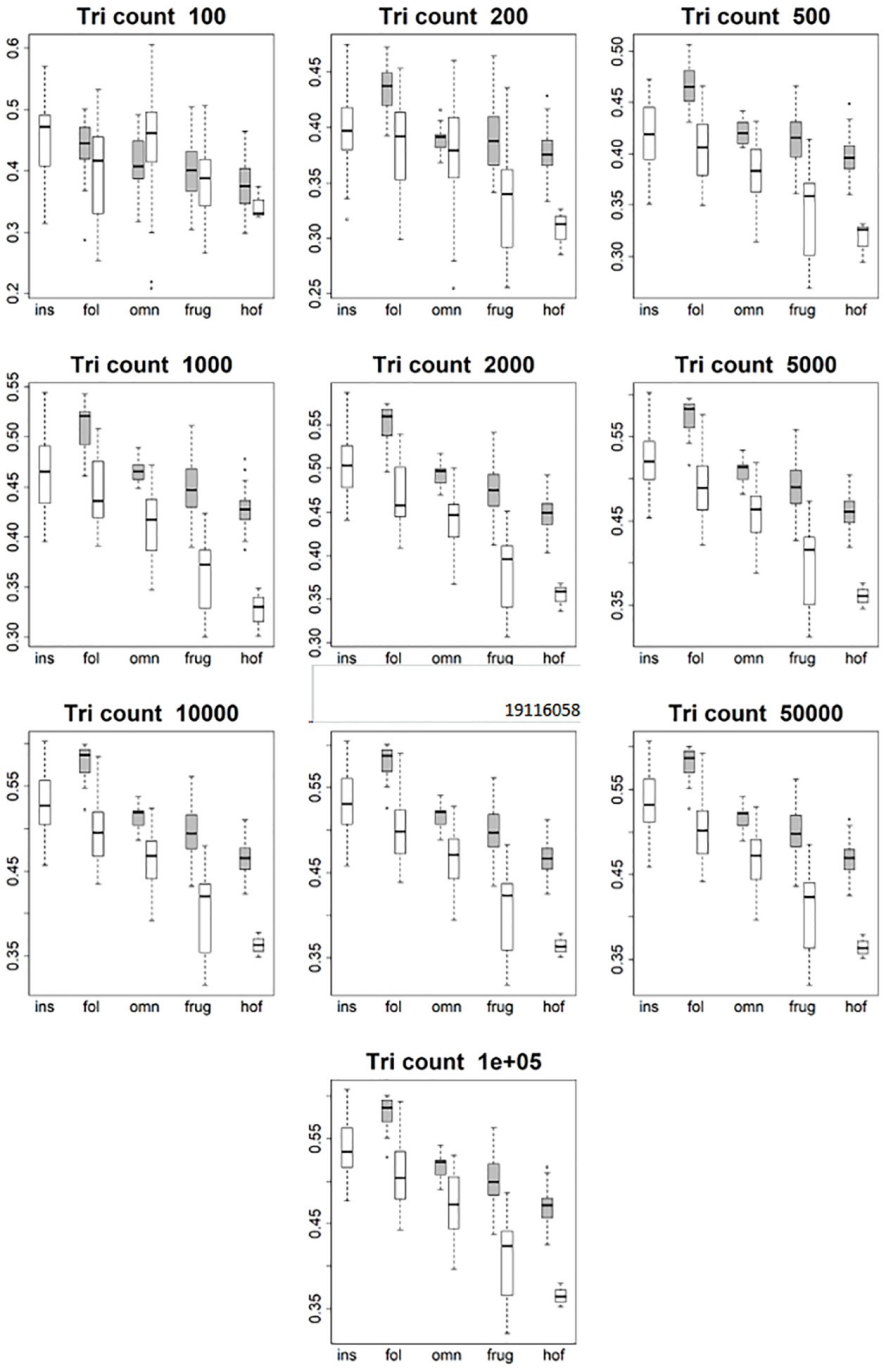
Boxplots showing prosimian (white) and platyrrhine (grey) RFI values for each diet at different triangle counts (smoothed, EEC). Diets are shown on the x-axis: ins = insectivore, fol = folivore, omn = omnivore, frug = frugivore, and hof = hard object feeder. Results at a triangle count of 10000 are comparable to those from [20]. Once an adequate triangle count is reached (about 500), a pattern develops in both prosimians and platyrrhines where insectivores have the highest values, followed by folivores, frugivores, and hard object feeders.

**Fig 7:**
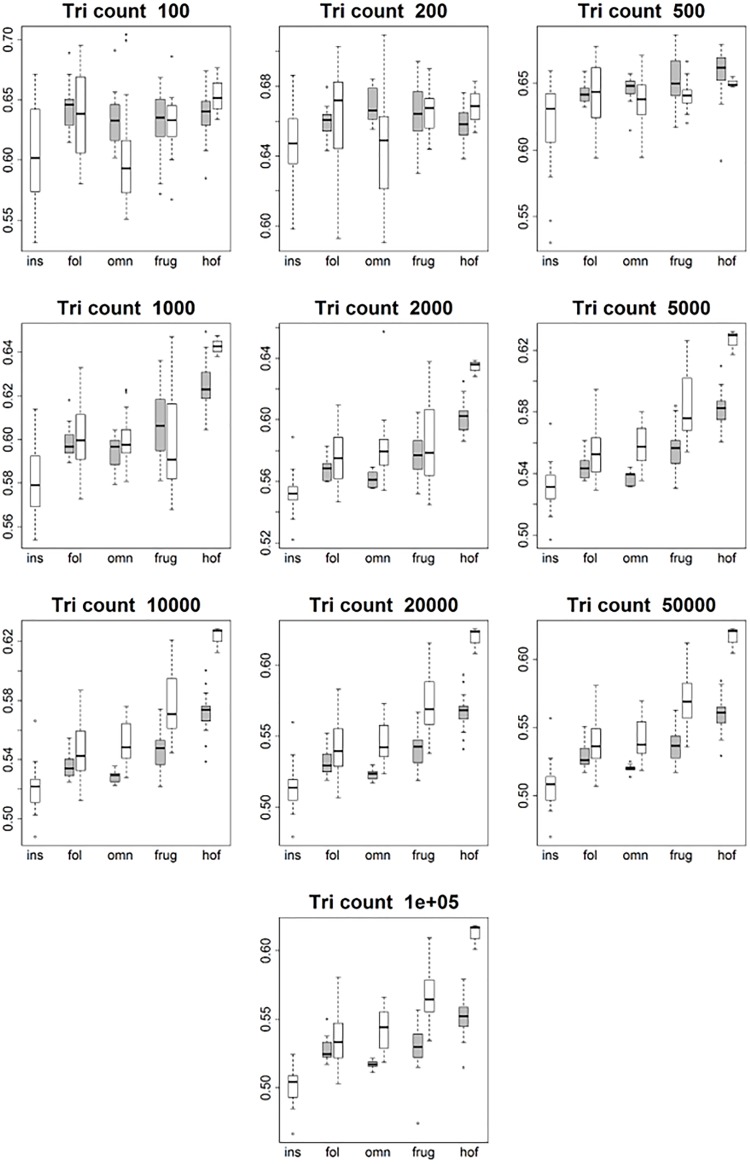
Boxplots showing prosimian (white) and platyrrhine (grey) PCV values for each diet at different triangle counts (smoothed, EEC). Diets are shown on the x-axis: ins = insectivore, fol = folivore, omn = omnivore, frug = frugivore, and hof = hard object feeder. Results at a triangle count of 10000 are comparable to those from [20]. As with RFI, once a sufficient triangle count is reached, a stable relationship develops between PCV and diet.

**Fig 8:**
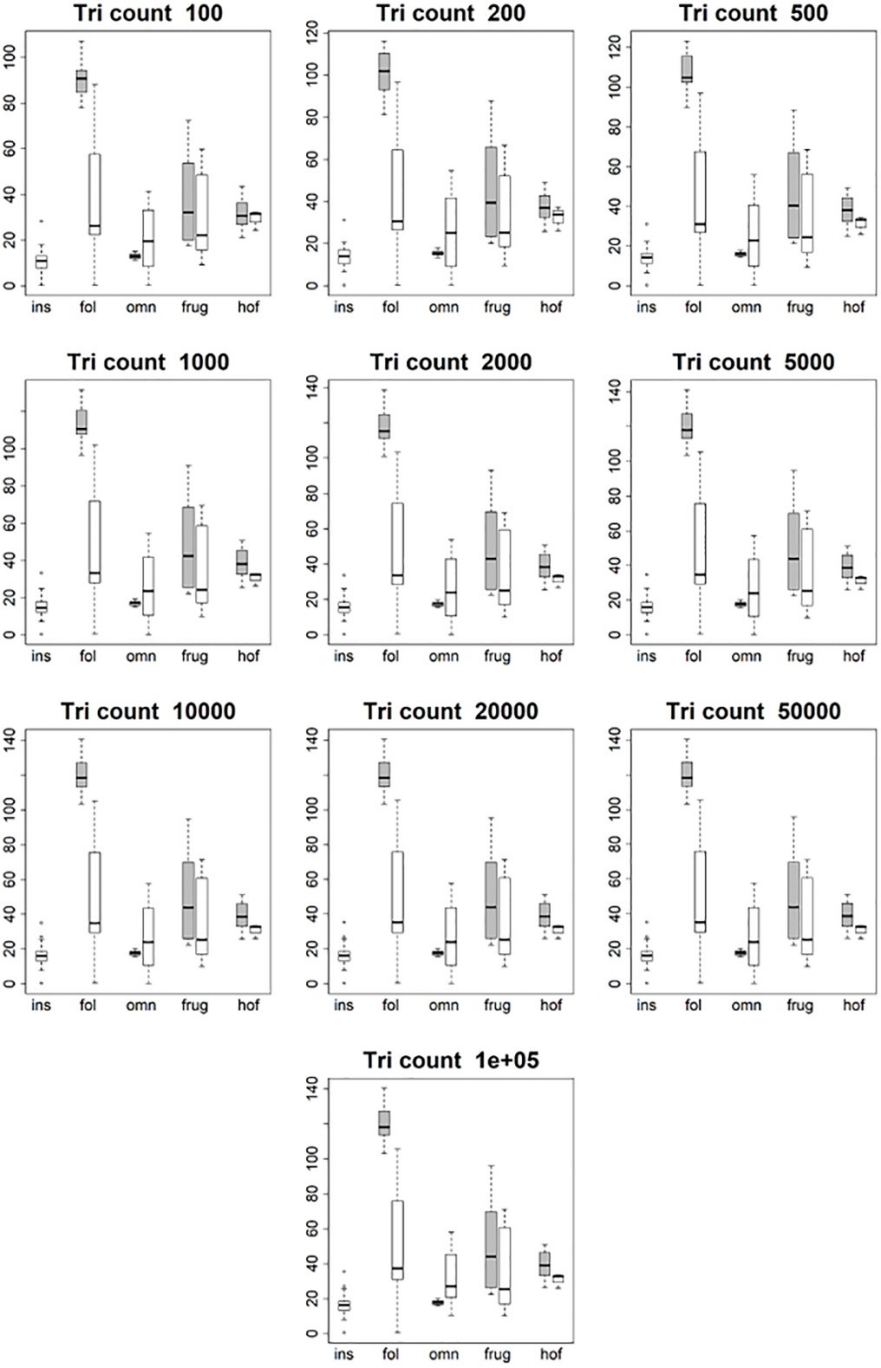
Boxplots showing prosimian (white) and platyrrhine (grey) surface area (SA) values for each diet at different triangle counts (smoothed, EEC). Diets are shown on the x-axis: ins = insectivore, fol = folivore, omn = omnivore, frug = frugivore, and hof = hard object feeder. As surface area is a measure of tooth size, no expected relationship is expected to emerge, other than folivores having larger teeth and insectivores having smaller teeth.

**Fig 9:**
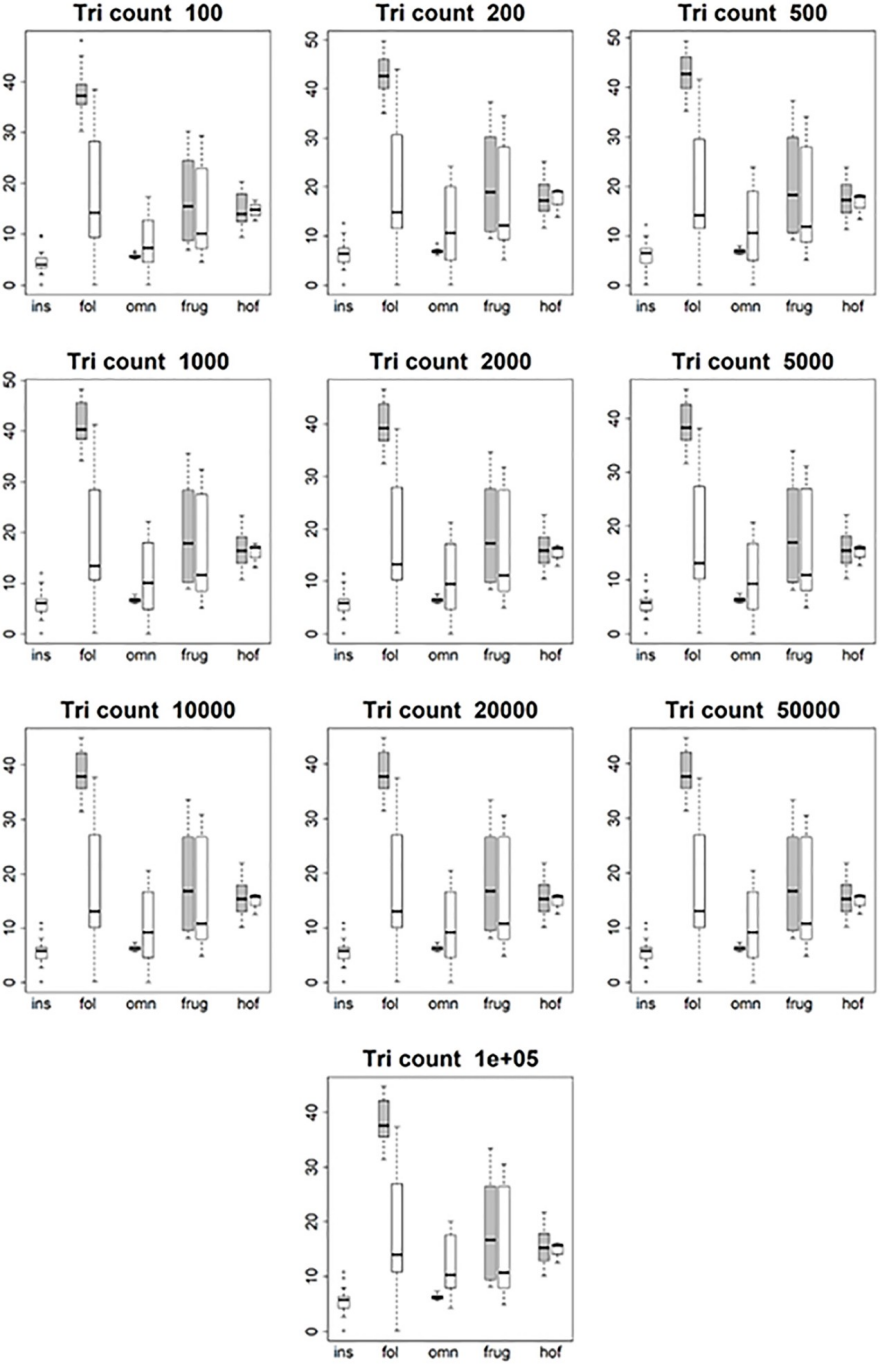
Boxplots showing prosimian (white) and platyrrhine (grey) tooth size (quantified through outline area, MorphoTester) values for each diet at different triangle counts (smoothed, EEC). Diets are shown on the x-axis: ins = insectivore, fol = folivore, omn = omnivore, frug = frugivore, and hof = hard object feeder. Results are almost identical to those in Fig 8 showing surface area (SA), as both size and SA are measures of size, not shape.

These differences in dietary categories were visualized with boxplots. 44 sets of 10 boxplots were created: four sets of 10 (EEC smoothed, EEC unsmoothed, BCO smoothed, BCO unsmoothed) for each topographic metric for triangle count and resolution separately. No boxplots were created for PCV using the BCO cropping method as those data were not gathered. Only 6 sets of boxplots are shown here, one set of 10 for DNE, OPCR, RFI, PCV, SA, and size (EEC, smoothed; Figs 4–9): all other boxplots can be found in the Supplementary Information section (S1 Fig). Descriptive statistics (sample size, mean, and standard deviation) were calculated for each topographic metric, subdivided by group and clade. As the descriptive statistics do not provide any new information that cannot be visualized by the boxplots, all descriptive statistics have been placed in the Supplementary Information section (S4 Table).

At the highest triangle counts (100,000), the relationship between DNE and dietary categories is opposite in prosimians and platyrrhines. In prosimians, insectivores have the highest median DNE scores, followed by folivores, omnivores, frugivores, and hard object feeders. In platyrrhines, hard object feeders have the highest median DNE score, followed by frugivores, omnivores, and folivores. There were no insectivorous platyrrhines (Fig 4). This pattern is also seen in the smoothed, BCO and the unsmoothed, EEC data. Unsmoothed, BCO data reveals the same pattern at a triangle count of 10,000, but not 100,000 (S1 Fig, S4 Table). There is no apparent reason why there are different relationships between DNE and diet at different triangle counts and with different smoothing and cropping methods.

As with DNE, the relationship between diet and OPCR changes with resolution. The relationship between OPCR and diet remains relatively constant once a triangle count of 10,000 is reached, and roughly holds constant regardless of smoothing or cropping method (Fig 5). However, as with DNE, unsmoothed BCO seems to be providing a unique set of results (S1 Fig, S4 Table). For both DNE and OPCR, when resolution, and not triangle count, is held constant, the relationship between DNE/OPCR and diet is consistent regardless of smoothing or cropping method once a high enough resolution is reached (about 5 triangles/mm^2^). The relationship between DNE/OPCR and diet, when resolution is held constant, does not resemble the relationship between DNE/OPCR and diet when triangle count is held constant: instead, it resembles the relationship between SA/size and diet (Figs 8 and 9). This is because larger teeth are comprised of more triangles when resolution are held constant, and since DNE and OPCR are summative metrics, this leads to higher DNE/OPCR values for larger teeth, and smaller DNE/OPCR values for smaller teeth. Visually, it appears that when resolution is held constant, DNE and OPCR are as accurate at differentiating teeth based on diet as SA or tooth size, and that DNE and OPCR are quantifying tooth size more than tooth shape. Therefore, it appears the conclusions of studies that use DNE and OPCR are sensitive to the methods used to gather the data (e.g., [23,45,48,63]). However, if triangle count (and not resolution), smoothing and cropping method are held constant, and triangle count is high enough to represent the tooth accurately, the conclusions should be robust.

Regardless of triangle count/resolution and clade, RFI always had the same relationship with diet: insectivores had the highest RFI values, followed by folivores, omnivores, frugivores, and then hard object feeders (Fig 6, S1 Fig, S4 Table). The same is true for PCV, which could be because of the previously reported high correlation between RFI and PCV [19,38]. It should be reiterated that PCV data were not gathered for the BCO cropping method, so the results for PCV could change when this cropping method is used (Fig 7, S1 Fig, S4 Table).

Both SA and tooth size showed the same pattern of results regardless of smoothing and cropping method (Figs 8 and 9). This is not surprising given the relatively low level of sensitivity of SA and size to smoothing and cropping methods (Figs 2 and 3). Within prosimians, folivores, hard object feeders, omnivores, and frugivores tend to have the largest teeth, and insectivores tend to have smaller teeth. In platyrrhines, folivores have the largest teeth, followed by frugivores and hard object feeders, and then omnivores.

One-way ANOVAs were run for each group and clade, separately, to investigate the relationship between tooth shape and size and diet. As it is not feasible to present the results of all 880 ANOVAs in this paper, results are presented in the Supplementary Information section (S5 Table) and summarized here. Out of the 440 ANOVAs where triangle count was held constant, 421 (95.9% of the ANOVAs) yielded p-values below 0.05, 408 (92.7%) yielded p-values less than 0.01, and 373 (84.8%) yielded p-values less than 0.0005. This suggests the means of DNE, OPCR, RFI, PCV, SA, and size were significantly different between dietary categories when smoothing, cropping method, and triangle count were held constant. When resolution was held constant, only 424 (96.4%) of the ANOVAs yielded p-values less than 0.05, 419 (95.2%) yielded p-values greater than 0.01, and 399 (90.7%) yielded p-values greater than 0.0005 (Table 5). Similar to triangle count, this suggests the means of DNE, OPCR, RFI, PCV, SA, and size were significantly different between dietary categories when smoothing, cropping method, and resolution were held constant. Additionally, triangle count and resolution yielded approximately the same number of ANOVAs with significant results.

**Table 5:** 880 one-way ANOVAs were run to test for differences due to dietary category, one for each combination of topographic metric, smoothing, cropping method, and triangle count/resolution. ANOVAs are divided between those run keeping triangle count constant (440) and those keeping resolution constant (440). Results here are the number of one-way ANOVAs with p-values less than 0.05, 0.01, and 0.0005, followed by the percent of the total ANOVAs in parentheses In general, all topographic metrics could successfully differentiate between dietary categories with a high level of accuracy, but DNE and OPCR seemed to perform slightly worse. Holding resolution constant yielded roughly the same results as holding triangle count constant.

	p < 0.05	p < 0.01	p < 0.0005
***Triangle Count***			
DNE	430 (97.7%)	426 (96.8%)	416 (94.5%)
OPCR	432 (98.2%)	425 (96.6%)	406 (92.3%)
RFI	440 (100%)	440 (100%)	438 (99.5%)
PCV	439 (99.8%)	437 (99.3%)	433 (98.4%)
SA	440 (100%)	440 (100%)	440 (100%)
Size	440 (100%)	440 (100%)	440 (100%)
***Resolution***			
DNE	430 (97.7%)	428 (97.3%)	426 (96.8%)
OPCR	440 (100%)	440 (100%)	438 (99.5%)
RFI	436 (99.1%)	435 (98.9%)	433 (98.4%)
PCV	438 (99.5%)	436 (99.1%)	433 (98.4%)
SA	440 (100%)	440 (100%)	440 (100%)
Size	440 (100%)	440 (100%)	440 (100%)

Therefore, average DNE, OPCR, RFI, PCV, SA, and tooth size nearly always differed between dietary categories in regardless of clade, smoothing, cropping method, and triangle count/resolution, if those factors are held constant. Further, SA and tooth size seemed to perform as good, if not better, than DNE and OPCR (Table 5).

To determine which dietary categories were statistically different, Tukey HSD tests were run in combination with each of the one-way ANOVAS. Feasibility issues preclude the presentation of the 880 Tukey HSD tests in the text, but results are presented in the Supplementary Information section (S5 and S6 Tables, S2 Fig) and summarized here in Tables 6 and 7. Regardless of triangle count, resolution, or p-value, there was always a significant difference between insectivores and folivores in SA and size. In the constant resolution, but not constant triangle count dataset, there was also always a significant difference between these groups in OPCR. There was also never or nearly never a difference in SA or size between insectivores and hard object feeders and omnivores, and between hard object feeders and frugivores in tooth size. Other notable results in the triangle count dataset include nearly consistent differences in RFI in the insectivore-frugivore, insectivore-hard object feeder, insectivore-omnivore, folivore-frugivore, folivore-hard object feeder, and omnivore-hard object feeder, and a nearly consistent difference in PCV between insectivores and all other primates. For the resolution dataset, nearly consistent differences existed in OPCR between folivores and omnivores, and similar results were seen in differences in RFI.

**Table 6:** Sum of number of Tukey HSD comparisons that yielded significant results (p < 0.05, 0.01, and 0.005) followed by percent of all Tukey HSD tests in parentheses for the triangle count dataset. P-values adjusted for multiple comparisons using the TukeyHSD() function in R. ins = insectivore, fol = folivore, frug = frugivore, omn = omnivore, and hof = hard object feeder. Regardless of clade, smoothing, cropping method, or triangle count, tooth size never differed between hard object feeders and frugivores, and surface area and tooth size never differentiate between insectivores and hard object feeders, and nearly never differentiated between insectivores and omnivores.

		ins-fol	ins-frug	ins-hof	ins-omn	fol-frug	fol-hof	fol-omn	omn-frug	omn-hof	hof-frug
DNE	Sample size	40	40	40	40	80	80	80	80	80	80
	*p* < 0.05	14 (35%)	33 (82.5%)	34 (85%)	31 (77.5%)	51 (63.8%)	63 (78.8%)	18 (22.5%)	37 (46.3%)	49 (61.3%)	24 (30%)
	*p* < 0.01	12 (30%)	33 (82.5%)	32 (80%)	29 (72.5%)	48 (60%)	53 (66.3%)	13 (16.3%)	27 (33.8%)	42 (52.5%)	20 (25%)
	*p* < 0.0005	7 (17.5%)	30 (75%)	26 (65%)	25 (62.5%)	26 (32.5%)	42 (52.5%)	5 (6.3%)	13 (16.3%)	25 (31.3%)	20 (25%)
OPCR	Sample size	40	40	40	40	80	80	80	80	80	80
	*p* < 0.05	6 (15%)	26 (65%)	15 (37.5%)	7 (17.5%)	31 (38.8%)	44 (55%)	24 (30%)	21 (26.3%)	36 (45%)	36 (45%)
	*p* < 0.01	2 (5%)	21 (52.5%)	12 (30%)	6 (15%)	27 (33.8%)	43 (53.8%)	11 (13.8%)	13 (16.3%)	29 (36.3%)	31 (38.8%)
	*p* < 0.0005	0 (0%)	14 (35%)	8 (20%)	2 (5%)	18 (22.5%)	33 (41.3%)	4 (5%)	6 (7.5%)	20 (25%)	18 (22.5%)
RFI	Sample size	40	40	40	40	80	80	80	80	80	80
	*p* < 0.05	24 (60%)	40 (100%)	39 (97.5%)	38 (95%)	78 (97.5%)	77 (96.3%)	47 (58.8%)	58 (72.5%)	74 (92.5%)	37 (46.3%)
	*p* < 0.01	20 (50%)	39 (97.5%)	38 (95%)	38 (95%)	77 (96.3%)	76 (95%)	26 (32.5%)	56 (70%)	72 (90%)	37 (46.3%)
	*p* < 0.0005	20 (50%)	39 (97.5%)	37 (92.5%)	38 (95%)	76 (95%)	74 (92.5%)	18 (22.5%)	34 (42.5%)	39 (48.8%)	35 (43.8%)
PCV	Sample size	20	20	20	20	40	40	40	40	40	40
	*p* < 0.05	19 (95%)	18 (90%)	17 (85%)	18 (90%)	18 (45%)	32 (80%)	6 (15%)	27 (67.5%)	35 (87.5%)	32 (80%)
	*p* < 0.01	19 (95%)	17 (85%)	17 (85%)	15 (75%)	14 (35%)	32 (80%)	4 (10%)	23 (57.5%)	34 (85%)	31 (77.5%)
	*p* < 0.0005	13 (65%)	15 (75%)	17 (85%)	13 (65%)	12 (30%)	28 (70%)	2 (5%)	17 (42.5%)	32 (80%)	27 (67.5%)
SA	Sample size	40	40	40	40	80	80	80	80	80	80
	*p* < 0.05	40 (100%)	40 (100%)	0 (0%)	0 (0%)	40 (50%)	40 (50%)	78 (97.5%)	40 (50%)	40 (50%)	34 (42.5%)
	*p* < 0.01	40 (100%)	8 (20%)	0 (0%)	0 (0%)	40 (50%)	40 (50%)	78 (97.5%)	40 (50%)	24 (30%)	17 (21.3%)
	*p* < 0.0005	40 (100%)	0 (0%)	0 (0%)	0 (0%)	40 (50%)	40 (50%)	43 (53.8%)	40 (50%)	0 (0%)	0 (0%)
Tooth size	Sample size	40	40	40	40	80	80	80	80	80	80
	*p* < 0.05	40 (100%)	40 (100%)	0 (0%)	2 (5%)	40 (50%)	40 (50%)	78 (97.5%)	41 (51.3%)	40 (50%)	0 (0%)
	*p* < 0.01	40 (100%)	40 (100%)	0 (0%)	0 (0%)	40 (50%)	40 (50%)	76 (95%)	40 (50%)	40 (50%)	0 (0%)
	*p* < 0.0005	40 (100%)	35 (87.5%)	0 (0%)	0 (0%)	40 (50%)	40 (50%)	41 (51.3%)	40 (50%)	40 (50%)	0 (0%)

**Table 7:** Sum of number of Tukey HSD comparisons that yielded significant results (p < 0.05, 0.01, and 0.005) followed by percent of all Tukey HSD tests in parentheses for the resolution dataset. P-values adjusted for multiple comparisons using the TukeyHSD() function in R. ins = insectivore, fol = folivore, frug = frugivore, omn = omnivore, and hof = hard object feeder.

		ins-fol	ins-frug	ins-hof	ins-omn	fol-frug	fol-hof	fol-omn	omn-frug	omn-hof	hof-frug
DNE	Sample size	40	40	40	40	80	80	80	80	80	80
	*p* < 0.05	28 (70%)	4 (10%)	7 (17.5%)	2 (5%)	63 (78.8%)	63 (78.8%)	69 (86.3%)	26 (32.5%)	4 (5%)	27 (33.8%)
	*p* < 0.01	18 (45%)	1 (2.5%)	0 (0%)	0 (0%)	60 (75%)	58 (72.5%)	67 (83.8%)	19 (23.8%)	3 (3.8%)	23 (28.8%)
	*p* < 0.0005	10 (25%)	0 (0%)	0 (0%)	0 (0%)	51 (63.8%)	40 (50%)	58 (72.5%)	8 (10%)	1 (1.3%)	16 (20%)
OPCR	Sample size	40	40	40	40	80	80	80	80	80	80
	*p* < 0.05	40 (100%)	14 (35%)	1 (2.5%)	0 (0%)	61 (76.3%)	46 (57.5%)	80 (100%)	45 (56.3%)	33 (41.3%)	16 (20%)
	*p* < 0.01	40 (100%)	8 (20%)	0 (0%)	0 (0%)	54 (67.5%)	39 (48.8%)	80 (100%)	40 (50%)	21 (26.3%)	11 (13.8%)
	*p* < 0.0005	40 (100%)	3 (7.5%)	0 (0%)	0 (0%)	36 (45%)	38 (47.5%)	74 (92.5%)	27 (33.8%)	10 (12.5%)	3 (3.8%)
RFI	Sample size	40	40	40	40	80	80	80	80	80	80
	*p* < 0.05	13 (32.5%)	35 (87.5%)	34 (85%)	33 (82.5%)	71 (88.8%)	69 (86.3%)	65 (81.3%)	42 (52.5%)	61 (76.3%)	36 (45%)
	*p* < 0.01	12 (30%)	35 (87.5%)	31 (77.5%)	32 (80%)	70 (87.5%)	69 (86.3%)	51 (63.8%)	37 (46.3%)	54 (67.5%)	36 (45%)
	*p* < 0.0005	11 (27.5%)	33 (82.5%)	28 (70%)	30 (75%)	66 (82.5%)	63 (78.8%)	30 (37.5%)	21 (26.3%)	39 (48.8%)	31 (38.8%)
PCV	Sample size	20	20	20	20	40	40	40	40	40	40
	*p* < 0.05	9 (45%)	13 (65%)	12 (60%)	11 (55%)	27 (67.5%)	31 (77.5%)	22 (55%)	11 (27.5%)	28 (70%)	27 (67.5%)
	*p* < 0.01	7 (35%)	11 (55%)	12 (60%)	11 (55%)	25 (62.5%)	30 (75%)	18 (45%)	10 (25%)	21 (52.5%)	26 (65%)
	*p* < 0.0005	4 (20%)	8 (40%)	11 (55%)	8 (40%)	18 (45%)	29 (72.5%)	12 (30%)	7 (17.5%)	14 (35%)	22 (55%)
SA	Sample size	40	40	40	40	80	80	80	80	80	80
	*p* < 0.05	40 (100%)	38 (95%)	0 (0%)	0 (0%)	40 (50%)	40 (50%)	80 (100%)	39 (48.8%)	36 (45%)	34 (42.5%)
	*p* < 0.01	40 (100%)	7 (17.5%)	0 (0%)	0 (0%)	40 (50%)	40 (50%)	80 (100%)	37 (46.3%)	22 (27.5%)	16 (20%)
	*p* < 0.0005	40 (100%)	0 (0%)	0 (0%)	0 (0%)	40 (50%)	40 (50%)	43 (53.8%)	37 (46.3%)	1 (1.3%)	0 (0%)
Size	Sample size	40	40	40	40	80	80	80	80	80	80
	*p* < 0.05	40 (100%)	38 (95%)	0 (0%)	1 (2.5%)	40 (50%)	40 (50%)	80 (100%)	40 (50%)	36 (45%)	4 (5%)
	*p* < 0.01	40 (100%)	38 (95%)	0 (0%)	0 (0%)	40 (50%)	40 (50%)	79 (98.8%)	39 (48.8%)	36 (45%)	2 (2.5%)
	*p* < 0.0005	40 (100%)	35 (87.5%)	0 (0%)	0 (0%)	40 (50%)	40 (50%)	42 (52.5%)	38 (47.5%)	36 (45%)	0 (0%)

Across all 60 comparisons with a p-value of 0.05, the triangle count dataset had a larger percentage of statistically significant differences between dietary categories than the resolution dataset 34 times, a smaller percentage 15 times, and the same percentage 11 times. This implies keeping a constant triangle count instead of a constant resolution could be beneficial because it results in a larger percentage of differences in dietary categories. However, the simplest surfaces in the resolution dataset (i.e., 1 triangle/mm^2^) arguably simplify the surfaces to a greater extent than the simplest surfaces in the triangle count dataset (i.e., 100 triangles), and so less significant differences could be occurring in the resolution dataset because of overly simplified surfaces.

Linear discriminant function analyses were run to test the ability to predict dietary category based on topographic metrics. In general, platyrrhines had a larger number of higher rates of correct classification than prosimians, regardless of smoothing, cropping, triangle count/resolution or topographic metric, as is evidenced by the bottom halves of Table 8 and Table 9 being more highlighted than the top halves. For constant triangle count, 91 of the 220 (41.4%) and 98 of the 220 (44.5%) of the cases using that were smoothed and unsmoothed, respectively, and 99 of the 240 (41.3%) and 90 of the 200 (45%) cases using EEC and BCO, respectively, had classification success rates greater than 50%. This suggests, that when triangle count is held constant, the number of higher rates of classification is not affected by smoothing or cropping. For constant resolution, 75 of the 220 (34.1%) and 108 of the 220 (49.1%) of the cases using that were smoothed and unsmoothed, respectively, and 96 of the 240 (40%) and 87 of the 200 (43.5%) cases using EEC and BCO, respectively, had classification success rates greater than 50%. This suggests, that when resolution is held constant, leaving the surfaces unsmoothed may lead to more high rates of correct classification, but the number of higher rates of classification is not affected by smoothing.

**Table 8:** Results of the linear discriminant function analyses when triangle count is held constant (Pros. = prosimian, Plat. = platyrrhine). Values reported are the cross-validated success rate of correctly classifying diet. Classifications greater than 50% are in bold and colored tan. In general, topographic metrics correctly classify diet in platyrrhines more often than in prosimians.

Clade	Cropping method	Smoothing	Topographic variable	Triangle count									
				100	200	500	1000	2000	5000	10000	20000	50000	100000
Pros.	EEC	smoothed	DNE	33	27.4	34	35.8	43.4	**50.9**	40.6	42.5	40.6	37.4
Pros.	EEC	smoothed	OPCR	31.1	24.5	29.2	31.1	31.1	19.8	27.4	30.2	33	23.1
Pros.	EEC	smoothed	RFI	28.3	29.2	34.9	38.7	48.1	44.3	43.4	43.4	46.2	47.3
Pros.	EEC	smoothed	PCV	32.1	17.9	30.2	38.7	38.7	**50.9**	**50.9**	**50.9**	**54.7**	**56**
Pros.	EEC	smoothed	SA	40.6	36.8	37.7	37.7	37.7	37.7	37.7	37.7	37.7	36.3
Pros.	EEC	smoothed	Size	41.5	39.6	37.7	38.7	38.7	38.7	37.7	37.7	37.7	40.7
Pros.	EEC	unsmoothed	DNE	43.4	44.3	37.7	41.5	45.3	41.5	39.6	25.5	41.5	28.6
Pros.	EEC	unsmoothed	OPCR	28.3	40.6	43.4	39.6	19.8	27.4	24.5	39.6	26.4	24.2
Pros.	EEC	unsmoothed	RFI	40.6	45.3	44.3	44.3	41.5	46.2	47.2	48.1	**52.8**	45.1
Pros.	EEC	unsmoothed	PCV	42.5	39.6	38.7	39.6	35.8	49.1	49.1	**53.8**	**51.9**	46.2
Pros.	EEC	unsmoothed	SA	38.7	37.7	37.7	37.7	37.7	37.7	37.7	37.7	37.7	36.3
Pros.	EEC	unsmoothed	Size	37.7	37.7	37.7	37.7	37.7	37.7	37.7	37.7	37.7	40.7
Pros.	BCO	smoothed	DNE	29.2	35.8	41.5	**51.9**	**53.8**	47.2	**52.8**	49.1	43.4	28.2
Pros.	BCO	smoothed	OPCR	30.2	31.1	34.9	34.9	34	35.8	35.8	17.9	31.1	28.2
Pros.	BCO	smoothed	RFI	39.6	43.4	47.2	**50.9**	**55.7**	**58.5**	**58.5**	**58.5**	**58.5**	**62**
Pros.	BCO	smoothed	SA	38.7	35.8	36.8	36.8	35.8	35.8	35.8	35.8	35.8	46.5
Pros.	BCO	smoothed	Size	41.5	39.6	40.6	40.6	40.6	40.6	40.6	40.6	40.6	45.1
Pros.	BCO	unsmoothed	DNE	40.6	**52.8**	46.2	42.5	43.4	43.4	34.9	32.1	23.6	29.6
Pros.	BCO	unsmoothed	OPCR	31.1	34	34.9	31.1	30.2	19.8	13.2	29.2	37.7	31
Pros.	BCO	unsmoothed	RFI	**56.6**	**58.5**	**57.5**	**57.5**	**58.5**	**55.7**	**54.7**	**54.7**	**54.7**	**62**
Pros.	BCO	unsmoothed	SA	35.8	35.8	35.8	35.8	35.8	35.8	35.8	35.8	35.8	46.5
Pros.	BCO	unsmoothed	Size	39.6	40.6	40.6	40.6	40.6	40.6	40.6	40.6	40.6	45.1
Plat.	EEC	smoothed	DNE	44.7	37.9	49.5	**75.7**	**82.5**	**74.8**	**59.2**	39.8	23.3	40.8
Plat.	EEC	smoothed	OPCR	37.9	37.9	**52.4**	**53.4**	46.6	17.5	28.2	32	38.8	52.4
Plat.	EEC	smoothed	RFI	35	45.6	49.5	**55.3**	**59.2**	**62.1**	**62.1**	**60.2**	**62.1**	**62.1**
Plat.	EEC	smoothed	PCV	27.2	32	40.8	**54.4**	**67**	**68.9**	**71.8**	**68.9**	**64.1**	**59.2**
Plat.	EEC	smoothed	SA	**61.2**	**60.2**	**58.3**	**58.3**	**62.1**	**63.1**	**62.1**	**62.1**	**61.2**	**61.2**
Plat.	EEC	smoothed	Size	**60.2**	**55.3**	**57.3**	**57.3**	**57.3**	**57.3**	**57.3**	**57.3**	**57.3**	**57.3**
Plat.	EEC	unsmoothed	DNE	**64.1**	**71.8**	**79.6**	**69.9**	**52.4**	35.9	35.9	44.7	**53.4**	**54.4**
Plat.	EEC	unsmoothed	OPCR	37.9	**57.3**	38.8	28.2	41.7	**61.2**	**58.3**	**50.5**	**60.2**	38.8
Plat.	EEC	unsmoothed	RFI	**58.3**	**61.2**	**59.2**	**62.1**	**61.2**	**61.2**	**60.2**	**59.2**	**57.3**	**60.2**
Plat.	EEC	unsmoothed	PCV	**53.4**	**58.3**	**71.8**	**63.1**	**67**	**62.1**	**55.3**	45.6	36.9	42.7
Plat.	EEC	unsmoothed	SA	**61.2**	**62.1**	**62.1**	**61.2**	**61.2**	**60.2**	**61.2**	**61.2**	**60.2**	**59.2**
Plat.	EEC	unsmoothed	Size	**57.3**	**57.3**	**57.3**	**57.3**	**57.3**	**57.3**	**57.3**	**57.3**	**57.3**	**57.3**
Plat.	BCO	smoothed	DNE	25.2	48.5	**68**	**74.8**	**63.1**	48.5	19.4	22.3	29.1	36.9
Plat.	BCO	smoothed	OPCR	47.6	**55.3**	**50.5**	**51.5**	39.8	36.9	41.7	43.7	42.7	48.5
Plat.	BCO	smoothed	RFI	49.5	47.6	**51.5**	**56.3**	**62.1**	**62.1**	**63.1**	**64.1**	**63.1**	**60.2**
Plat.	BCO	smoothed	SA	**62.1**	**64.1**	**66**	**65**	**63.1**	**65**	**65**	**65**	**65**	**65**
Plat.	BCO	smoothed	Size	**53.4**	**57.3**	**57.3**	**57.3**	**58.3**	**58.3**	**58.3**	**58.3**	**58.3**	**58.3**
Plat.	BCO	unsmoothed	DNE	71.8	71.8	61.2	44.7	34	33	34	28.2	33	39.8
Plat.	BCO	unsmoothed	OPCR	45.6	35	30.1	49.5	**59.2**	**58.3**	39.8	31.1	23.3	25.2
Plat.	BCO	unsmoothed	RFI	**58.3**	**61.2**	**61.2**	**62.1**	**59.2**	**58.3**	**60.2**	**59.2**	**59.2**	**59.2**
Plat.	BCO	unsmoothed	SA	**64.1**	**65**	**65**	**65**	**65**	**65**	**65**	**65**	**65**	**65**
Plat.	BCO	unsmoothed	Size	**57.3**	**57.3**	**58.3**	**58.3**	**58.3**	**58.3**	**58.3**	**58.3**	**58.3**	**58.3**

**Table 9:** Results of the linear discriminant function analyses when resolution is held constant (Pros. = prosimian, Plat. = platyrrhine). Values reported are the cross-validated success rate of correctly classifying diet. Classifications greater than 50% are in bold and colored tan. In general, topographic metrics correctly classify diet in platyrrhines more often than in prosimians.

Clade	Cropping method	Smoothing	Topographic variable	Resolution (triangles/mm^2^)									
				1	2	5	10	20	50	100	200	500	1000
Pros.	EEC	smoothed	DNE	21.7	13.2	16	27.4	22.6	30.2	27.4	29.2	34.9	31.1
Pros.	EEC	smoothed	OPCR	38.7	37.7	26.4	21.7	21.7	19.8	36.8	36.8	40.6	35.8
Pros.	EEC	smoothed	RFI	22	22.5	33.3	37.7	31.1	25.5	36.8	38.7	41.5	41.5
Pros.	EEC	smoothed	PCV	18.9	16	36.8	17.9	31.1	22.6	27.4	34.9	45.3	**52.8**
Pros.	EEC	smoothed	SA	21.7	33	40.6	40.6	37.7	38.7	37.7	38.7	38.7	38.7
Pros.	EEC	smoothed	Size	23.6	35.8	39.6	42.5	39.6	39.6	39.6	39.6	38.7	37.7
Pros.	EEC	unsmoothed	DNE	34	31.1	28.3	34.9	41.5	41.5	37.7	40.6	34.9	30.2
Pros.	EEC	unsmoothed	OPCR	36.8	37.7	44.3	29.2	33	17.9	34.9	45.3	45.3	36.8
Pros.	EEC	unsmoothed	RFI	24.5	24.5	34.9	40.6	45.3	46.2	44.3	43.4	44.3	45.3
Pros.	EEC	unsmoothed	PCV	28.3	29.2	33	24.5	34	32.1	46.2	**52.8**	**53.8**	**57.5**
Pros.	EEC	unsmoothed	SA	38.7	38.7	37.7	38.7	38.7	38.7	38.7	38.7	38.7	38.7
Pros.	EEC	unsmoothed	Size	39.6	38.7	37.7	37.7	37.7	37.7	37.7	37.7	37.7	37.7
Pros.	BCO	smoothed	DNE	24.5	29.2	28.3	32.1	22.6	28.3	28.3	31.1	30.2	34
Pros.	BCO	smoothed	OPCR	34	34.9	24.5	23.6	24.5	34	38.7	38.7	41.5	45.3
Pros.	BCO	smoothed	RFI	17.5	25.2	38.5	44.3	22.6	46.2	**54.7**	**58.5**	**59.4**	**60.4**
Pros.	BCO	smoothed	SA	37.7	34.9	36.8	36.8	36.8	34.9	34.9	34.9	34.9	34.9
Pros.	BCO	smoothed	Size	34.9	37.7	40.6	41.5	39.6	40.6	40.6	40.6	40.6	40.6
Pros.	BCO	unsmoothed	DNE	30.2	22.6	22.6	31.1	34.9	34	33	30.2	34.9	34
Pros.	BCO	unsmoothed	OPCR	32.1	34	36.8	34	23.6	37.7	35.8	41.5	38.7	31.1
Pros.	BCO	unsmoothed	RFI	37.7	43.4	**53.8**	**58.5**	**57.5**	**57.5**	**58.5**	**56.6**	**55.7**	**59.4**
Pros.	BCO	unsmoothed	SA	35.8	35.8	35.8	34.9	34.9	34.9	34.9	34.9	34.9	34.9
Pros.	BCO	unsmoothed	Size	40.6	39.6	40.6	40.6	40.6	40.6	40.6	40.6	40.6	40.6
Plat.	EEC	smoothed	DNE	21.4	35.9	42.7	**62.1**	**67**	28.2	**60.2**	**57.3**	37.9	35.9
Plat.	EEC	smoothed	OPCR	42.7	**52.4**	**54.4**	46.6	**68**	48.5	41.7	47.6	48.5	40.8
Plat.	EEC	smoothed	RFI	**52.6**	29.1	42.7	**60.2**	**56.3**	**61.2**	**60.2**	**61.2**	**59.2**	**61.2**
Plat.	EEC	smoothed	PCV	47.6	**53.4**	43.7	**51.5**	47.6	36.9	**55.3**	**58.3**	**59.2**	**56.3**
Plat.	EEC	smoothed	SA	**50.5**	48.5	29.1	**62.1**	**63.1**	**64.1**	**63.1**	**62.1**	**62.1**	**60.2**
Plat.	EEC	smoothed	Size	46.6	44.7	**57.3**	**58.3**	**57.3**	**57.3**	**57.3**	**57.3**	**57.3**	**57.3**
Plat.	EEC	unsmoothed	DNE	**69.9**	**66**	**61.2**	**50.5**	**53.4**	38.8	43.7	**50.5**	**54.4**	**54.4**
Plat.	EEC	unsmoothed	OPCR	**52.4**	**57.3**	**62.1**	**52.4**	**52.4**	40.8	**57.3**	**58.3**	**56.3**	**56.3**
Plat.	EEC	unsmoothed	RFI	**57.3**	45.6	**53.4**	**60.2**	**61.2**	**62.1**	**61.2**	**61.2**	**63.1**	**63.1**
Plat.	EEC	unsmoothed	PCV	48.5	**56.3**	**55.3**	**56.3**	**56.3**	**52.4**	**57.3**	**57.3**	**56.3**	**50.5**
Plat.	EEC	unsmoothed	SA	**61.2**	**62.1**	**61.2**	**64.1**	**61.2**	**62.1**	**63.1**	**63.1**	**63.1**	**63.1**
Plat.	EEC	unsmoothed	Size	**56.3**	**57.3**	**57.3**	**57.3**	**57.3**	**57.3**	**57.3**	**57.3**	**57.3**	**57.3**
Plat.	BCO	smoothed	DNE	10.7	8.7	**61.2**	34	**57.3**	**62.1**	**60.2**	**60.2**	44.7	33
Plat.	BCO	smoothed	OPCR	43.7	**57.3**	**66**	46.6	**61.2**	49.5	47.6	46.6	44.1	41.7
Plat.	BCO	smoothed	RFI	18.8	36.9	**58.3**	**52.4**	**61.2**	**54.4**	**54.4**	**60.2**	**62.1**	**62.1**
Plat.	BCO	smoothed	SA	48.5	**56.3**	**62.1**	**67**	**66**	**63.1**	**64.1**	**65**	**65**	**65**
Plat.	BCO	smoothed	Size	43.7	46.6	**53.4**	**58.3**	**58.3**	**58.3**	**58.3**	**58.3**	**58.3**	**58.3**
Plat.	BCO	unsmoothed	DNE	**57.3**	**60.2**	**55.3**	**53.4**	**54.4**	41.7	37.9	38.8	49.5	45.6
Plat.	BCO	unsmoothed	OPCR	**56.3**	**55.3**	**54.4**	**54.4**	**54.4**	**50.5**	**50.5**	46.6	44.7	46.6
Plat.	BCO	unsmoothed	RFI	**53.4**	**52.4**	**62.1**	**64.1**	**58.3**	**59.2**	**58.3**	**58.3**	**61.2**	**60.2**
Plat.	BCO	unsmoothed	SA	**62.1**	**66**	**63.1**	**64.1**	**65**	**65**	**65**	**65**	**65**	**65**
Plat.	BCO	unsmoothed	Size	**59.2**	**56.3**	**57.3**	**58.3**	**58.3**	**58.3**	**58.3**	**58.3**	**58.3**	**58.3**

Correlations between topographic variables for each combination of smoothing, cropping method, and triangle count/resolution were calculated. The 1000 correlations with their coefficients of correlation, p-values, and test statistics are found in the Supplementary Information section (S7 Table) and summarized in Table 10. When triangle count was held constant, DNE was frequently correlated to OPCR, RFI, and PCV, and RFI was frequently/always correlated to OPCR/PCV. SA and size were infrequently or never correlated to DNE, OPCR, RFI, and PCV, but always correlated to each other. When resolution was held constant, all topographic variables were frequently or always correlated to one another. This variable correlation between topographic variables is consistent with other studies [19,20,38].

**Table 10:** Percent of times significant correlations (Bonferroni corrected p-value of 0.05/15 = 0.0033) were found between variables. More correlations were found between variables when resolution was held constant.

***Triangle count***						
	DNE	OPCR	RFI	PCV	SA	Size
DNE	---	---	---	---	---	---
OPCR	92.5	---	---	---	---	---
RFI	85	67.5	---	---	---	---
PCV	100	55	100	---	---	---
SA	22.5	35	45	0	---	---
Size	42.5	30	30	5	100	---
***Resolution***						
	DNE	OPCR	RFI	PCV	SA	Size
DNE	---	---	---	---	---	---
OPCR	95	---	---	---	---	---
RFI	90	67.5	---	---	---	---
PCV	90	85	95	---	---	---
SA	92.5	100	70	85	---	---
Size	92.5	100	60	80	100	---

**Fig 10:**
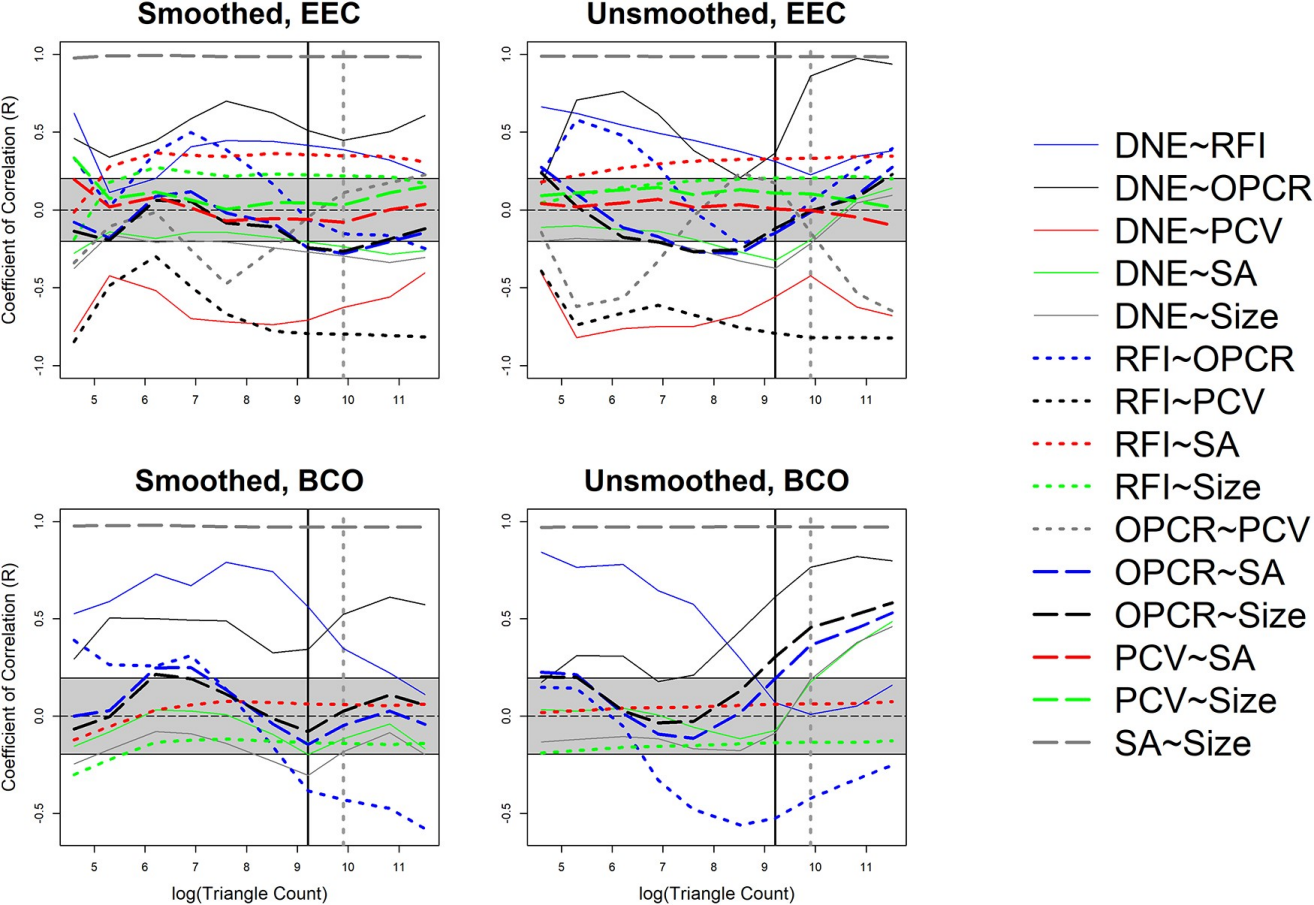
Coefficients of correlations (R) plotted against the natural log of triangle count to show how correlations between topographic variables change with triangle count. The grey area shows non-significant correlations between variables with a Bonferroni corrected p-value (0.05/15 = 0.00333). Vertical solid and dashed lines indicate triangle counts of 10000 and 20000, respectively.

**Fig 11:**
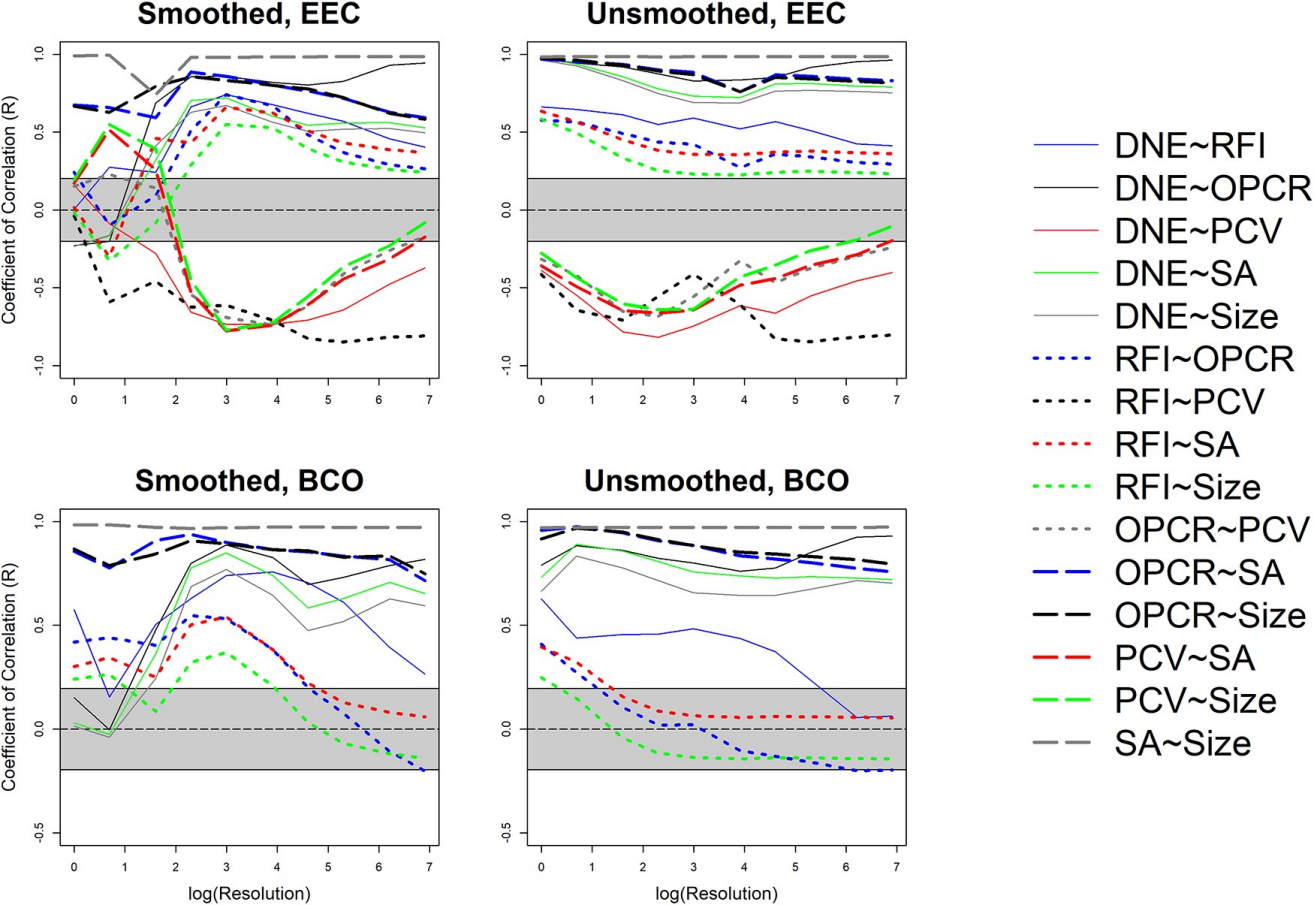
Coefficients of correlations (R) plotted against the natural log of resolution to show how correlations between topographic variables change with resolution. The grey area shows non-significant correlations between variables with a Bonferroni corrected p-value (0.05/15 = 0.00333).

Coefficients of correlation are plotted against triangle count and resolution in Figs 10 and 11, to give a visualization about how the strength of relationships between variables changes with triangle count and resolution. Unsurprisingly, SA and size are always strongly correlated to each other. Some metrics are always/never correlated to one another (e.g., PCV and SA), while others are correlated at some triangle counts/resolutions. What is particularly interesting is some topographic metrics are significantly positively correlated to each other at some triangle counts/resolutions, and significantly negatively correlated to each other at others (e.g. RFI and OPCR, smoothed, BCO, Fig 10, or PCV and SA/size, smoothed, EEC, Fig 11). Others (e.g., RFI and OPCR with triangle count, or PCV and SA with resolution) begin positively correlated at low triangle counts/resolutions, and end up being negatively correlated.

There is large variability in correlations between metrics, and correlations appear to be stronger when resolution is held constant, compared to triangle count. There are no consistent trends between correlation and triangle count/resolution: this is important when choosing topographic metrics, as correlations between two metrics in one study/clade does not dictate a correlation between metrics in another.

Overall, it appears triangle count and resolution performed similarly, with resolution performing slightly worse. There appears to be more overlap in dietary categories when resolution is held constant (S1 Fig, S4–S6 Tables and there appears to be more differentiation in dietary categories when triangle count, and not resolution, is held constant (Table 6, Table 7). Linear DFAs also appear to yield more significant results more often when triangle count is held constant (see below). The high level of correlations between topographic variables when resolution is held constant indicates the topographic variables are all highly correlated to surface area when resolution is held constant. This implies the topographic metrics may be acting more like form variables (shape and size, together) than shape variables. Because of these reasons, and because it is done more frequently in dental topographic studies, we suggest triangle count, and not resolution, be held constant.

### Effect of triangle count and resolution on results

**Fig 12:**
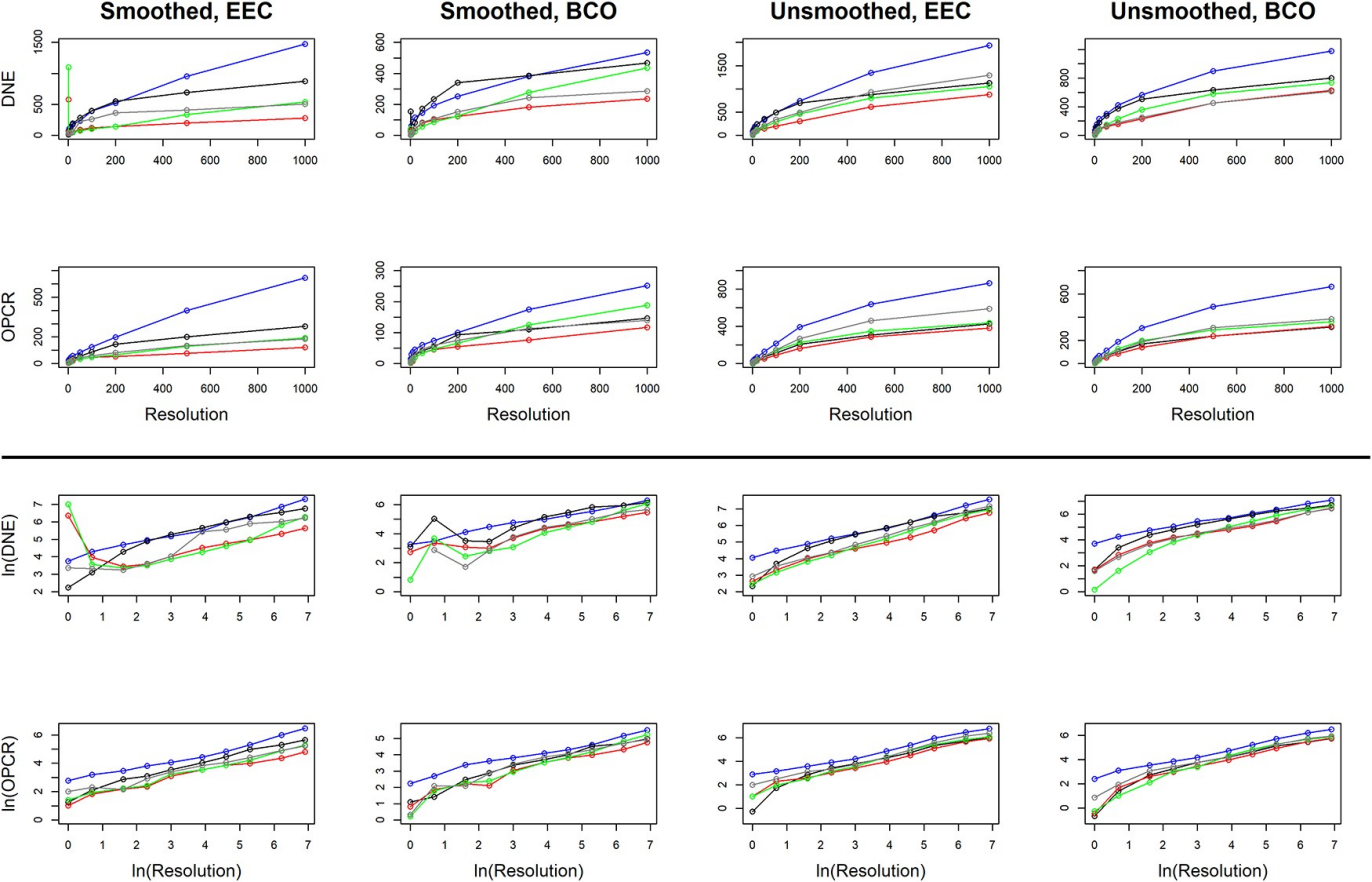
Effect of resolution on DNE and OPCR in five primates. The blue line is a folivorous prosimian (AMNH100503), black line is an insectivorous prosimian (AMNH207949), red line is a folivorous platyrrhine (AMNH211465), and green and grey lines are hard object feeding platyrrhines (MCZ30720 and USNM291128).

**Fig 13:**
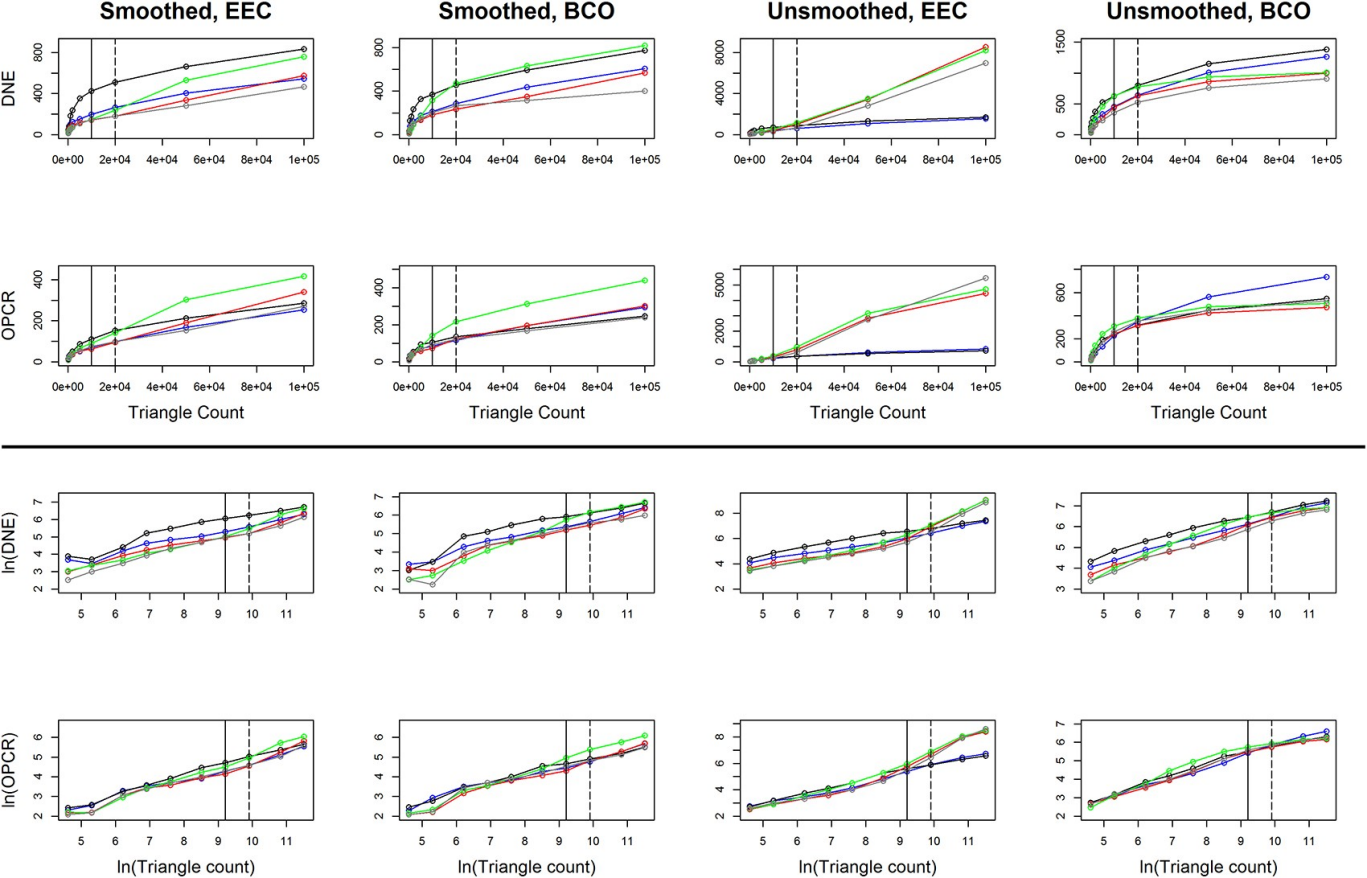
Effect of triangle count on DNE and OPCR in five primates. The blue line is a folivorous prosimian (AMNH100503), black line is an insectivorous prosimian (AMNH207949), red line is a folivorous platyrrhine (AMNH211465), and green and grey lines are hard object feeding platyrrhines (MCZ30720 and USNM291128).

**Fig 14:**
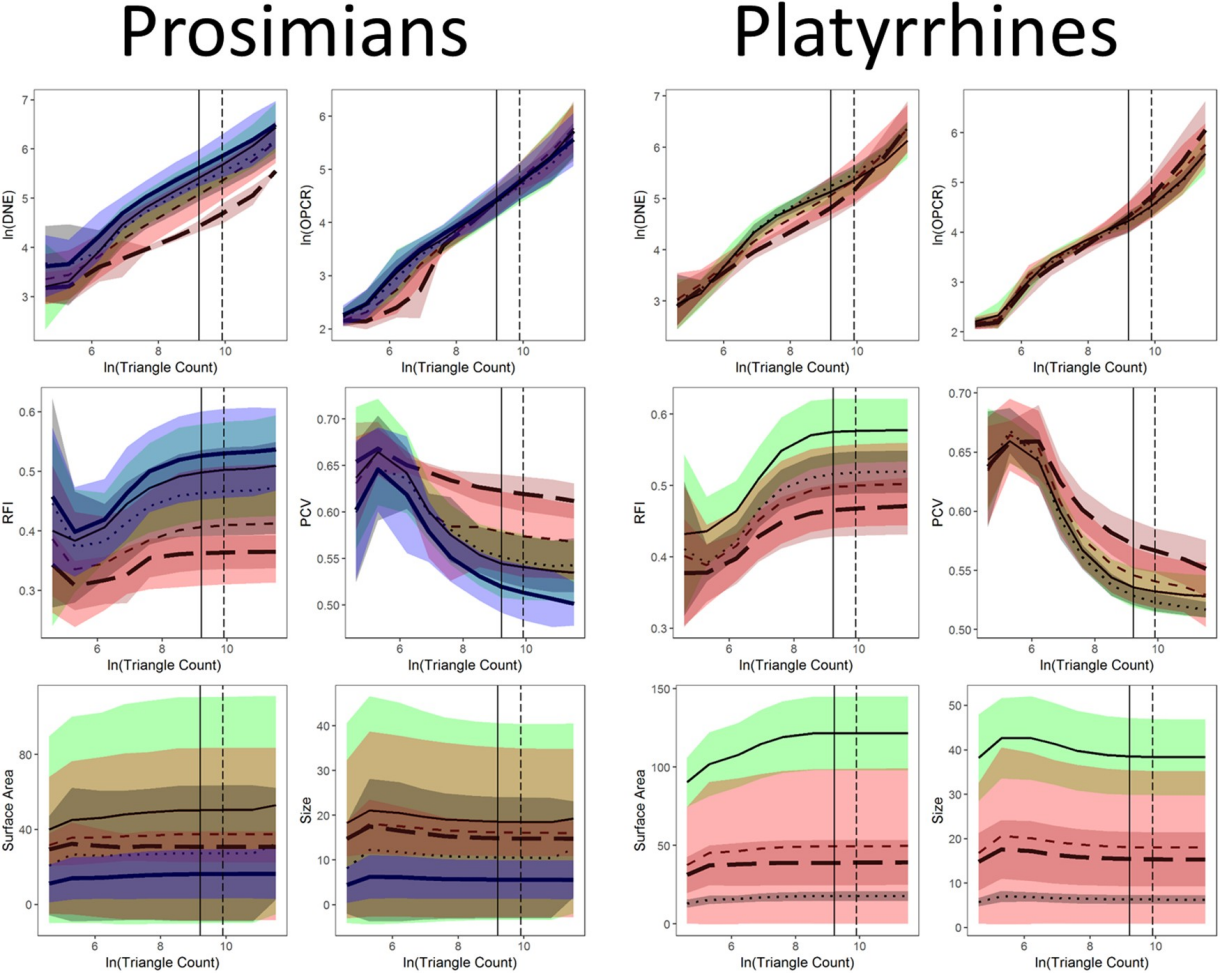
Effect of triangle count on topographic metrics (smoothed, EEC). Averages for each topographic value are given with a 95% confidence interval. Blue = insectivore, green = folivore, grey = omnivore, red = frugivore, brown = hard object feeder. Thick solid line = insectivore, thin solid line = folivore, small dotted line = omnivore, thin dashed line = frugivore, thick dashed line = hard object feeder. Vertical solid and dashed lines indicate triangle counts of 10000 and 20000, respectively.

**Fig 15:**
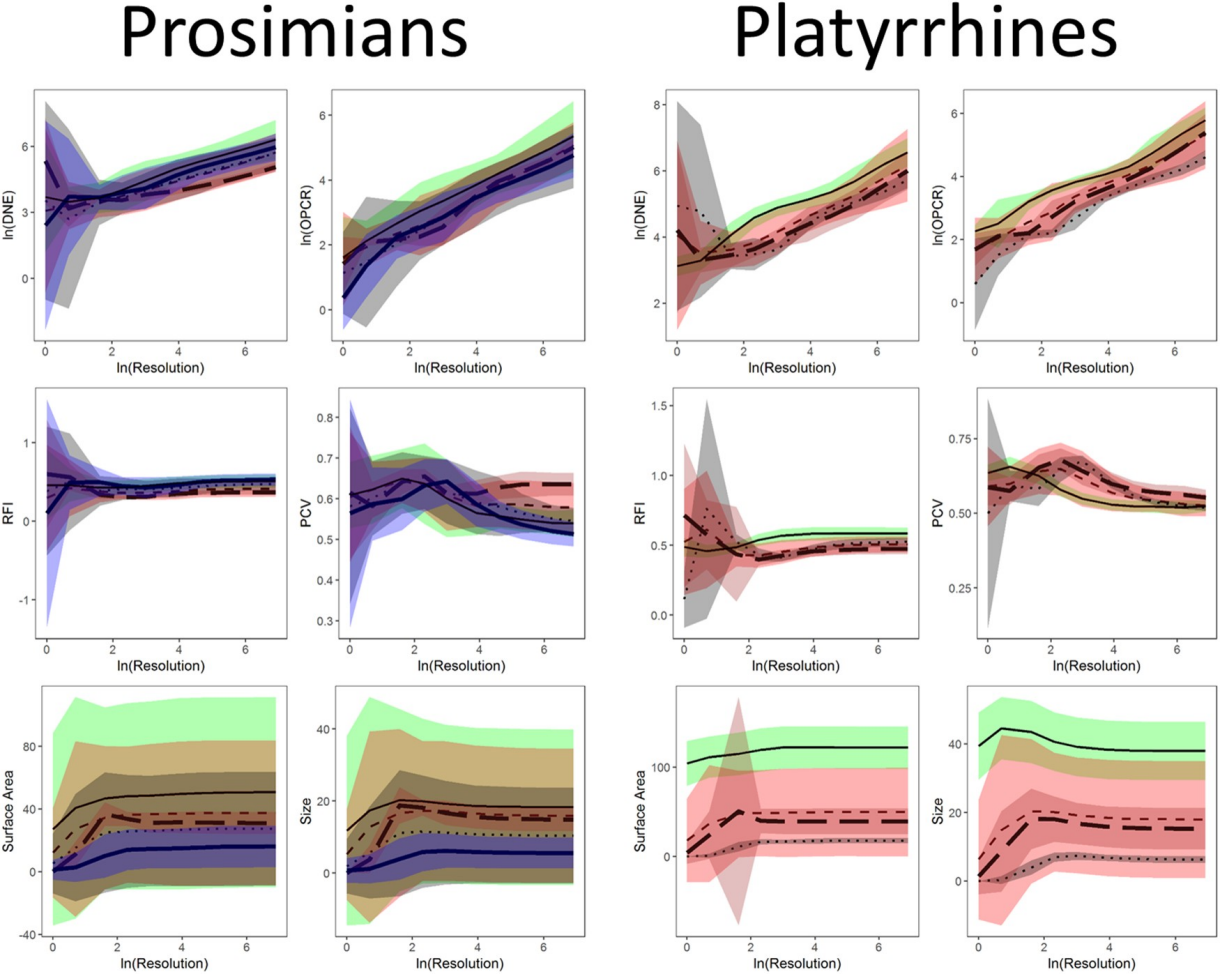
Effect of resolution on topographic metrics (smoothed, EEC). Averages for each topographic value are given with a 95% confidence interval. Blue = insectivore, green = folivore, grey = omnivore, red = frugivore, brown = hard object feeder. Thick solid line = insectivore, thin solid line = folivore, small dotted line = omnivore, thin dashed line = frugivore, thick dashed line = hard object feeder.

The values for triangle count/resolution increased exponentially, and as such the natural log of triangle count/ resolution was used. Both DNE and OPCR tend to increase exponentially with increases in triangle count/resolution (Figs 12 and 13). Linear plots were created to investigate the effect of triangle count/resolution on topographic variables. Each line type represents a different diet, and each shaded region represents the 95% confidence interval for that diet (see Figs 14 and 15, and S3 Fig). Linear plots were created for each smoothing and cropping method, separately, and only the plots for smoothed, EEC are presented here: other plots are presented in the Supplementary Information (S3 Fig). When triangle count is held constant, the natural log of DNE and OPCR increase linearly with the natural log of triangle count. Not all relationships are perfectly linear (e.g., DNE, platyrrhines, Fig 14), which is consistent with the OPCR results presented in [45] and [48]. At triangle counts above roughly 3000 (ln(3000) = 8.006), the average OPCR values converge for all dietary categories, and OPCR no longer appears to be a good predictor of diet in prosimians or platyrrhines. At higher triangle count, above 20000, the average OPCR values appear to differentiate again for platyrrhines, but not prosimians. For DNE, platyrrhines follow an odd pattern, with folivores having the highest values at triangle counts around 3000, and then the lowest at the highest triangle counts, a pattern reflected in the boxplots (Figs 4 and 14). The large degree of overlap in 95% confidence intervals for many metrics (e.g., DNE, OPCR, RFI, and PCV with resolution) highlights the overlap in topographic values for different dietary categories. There appears to be less overlap in triangle count (compared to resolution), suggesting it may be easier to predict diet when triangle count is held constant instead of resolution.

At high triangle counts, SA and size converge on an asymptote for both prosimians and platyrrhines, causing RFI to converge. PCV follows a unique pattern, initially increasing and then steadily decreasing with triangle count. It appears PCV decreases with an increase in triangle count at different rates for different diets, with insectivores/folivores decreasing at a faster rate than frugivores and hard object eaters. This could be because insectivores and folivores have taller cusps and increases in triangle count could lead to an increase in the percentage of triangles more hidden by ambient light relative to those more exposed to ambient light. That is to say, increasing triangle count places relatively more triangles in the basins and on the sides of the tooth, which are more hidden, compared to the tips of the cusps and the crests, which are more exposed, because there is more surface area in the basins and on the sides of the tooth. This pattern is seen in both prosimians and platyrrhines (Fig 14). Unlike DNE and OPCR, PCV does appear to be converging on an asymptote, and more so for hard object feeders than other diets.

When keeping resolution constant, many of the same patterns emerge. At resolutions below 10 triangles/mm^2^ (ln(10) = 2.303), the surfaces tend to change radically with smoothing, which causes erratic values for topographic metrics, and for large standard deviations to develop (Fig 15). As with triangle count, RFI, SA and size converge relatively quickly, usually after a resolution of 20 (ln(20) = 2.996), the natural log of DNE and OPCR increase linearly with the natural log of resolution, and after reaching a peak around a resolution of 20, PCV steadily decreases, and at a faster rate for insectivores/folivores than frugivores/hard object feeders.

A solid vertical line is drawn at a triangle count of 10000, a triangle count commonly used in DNE studies (e.g., [20]), and a dotted vertical line is drawn at a triangle count of 20000, a triangle count used in a DNE study by [38,50] (Fig 14). Dietary categories appear to have similar levels of overlap and differentiation at triangle counts of 10000 and 20000: the only exception is there may be more differentiation in OPCR between dietary categories in platyrrhines, but the difference is small. At a triangle count of 10000, SA, size, and RFI appear to have already converged in prosimians and platyrrhines. However, RFI does not appear to have converged in platyrrhines until this point, indicating the minimum triangle count for calculating RFI should be 10000 triangles. PCV appears to be converging in some cases (Figs 14 and 15, S3 Fig), but at variable triangle counts/resolutions.

**Fig 16:**
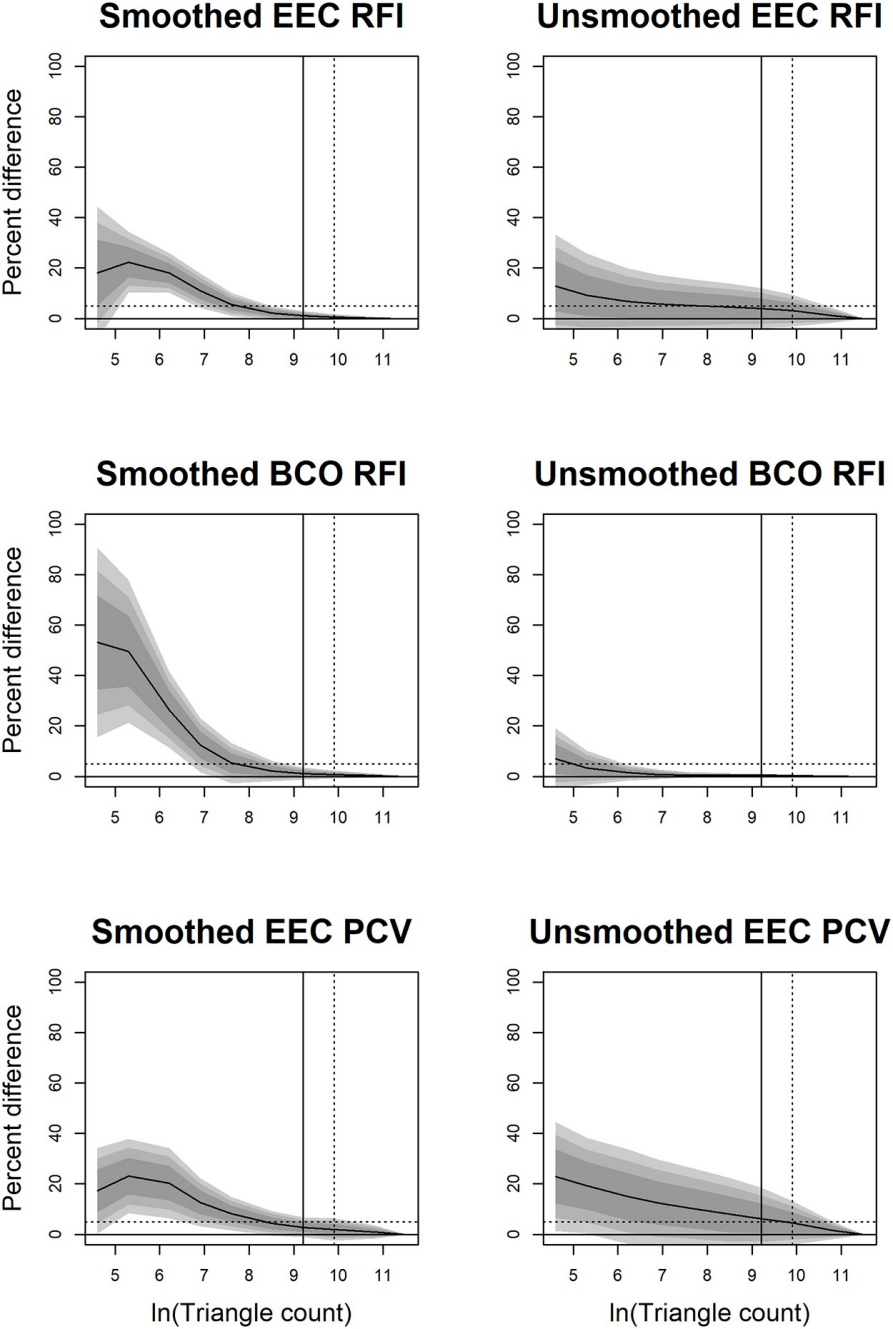
Absolute value of the percent difference between RFI and PCV values at the highest triangle count and lower triangle counts. Dotted horizontal lines represent 5% difference. The confidence intervals are drawn at 80%, 95%, and 99%. Vertical solid and dashed lines indicate triangle counts of 10000 and 20000, respectively.

**Fig 17:**
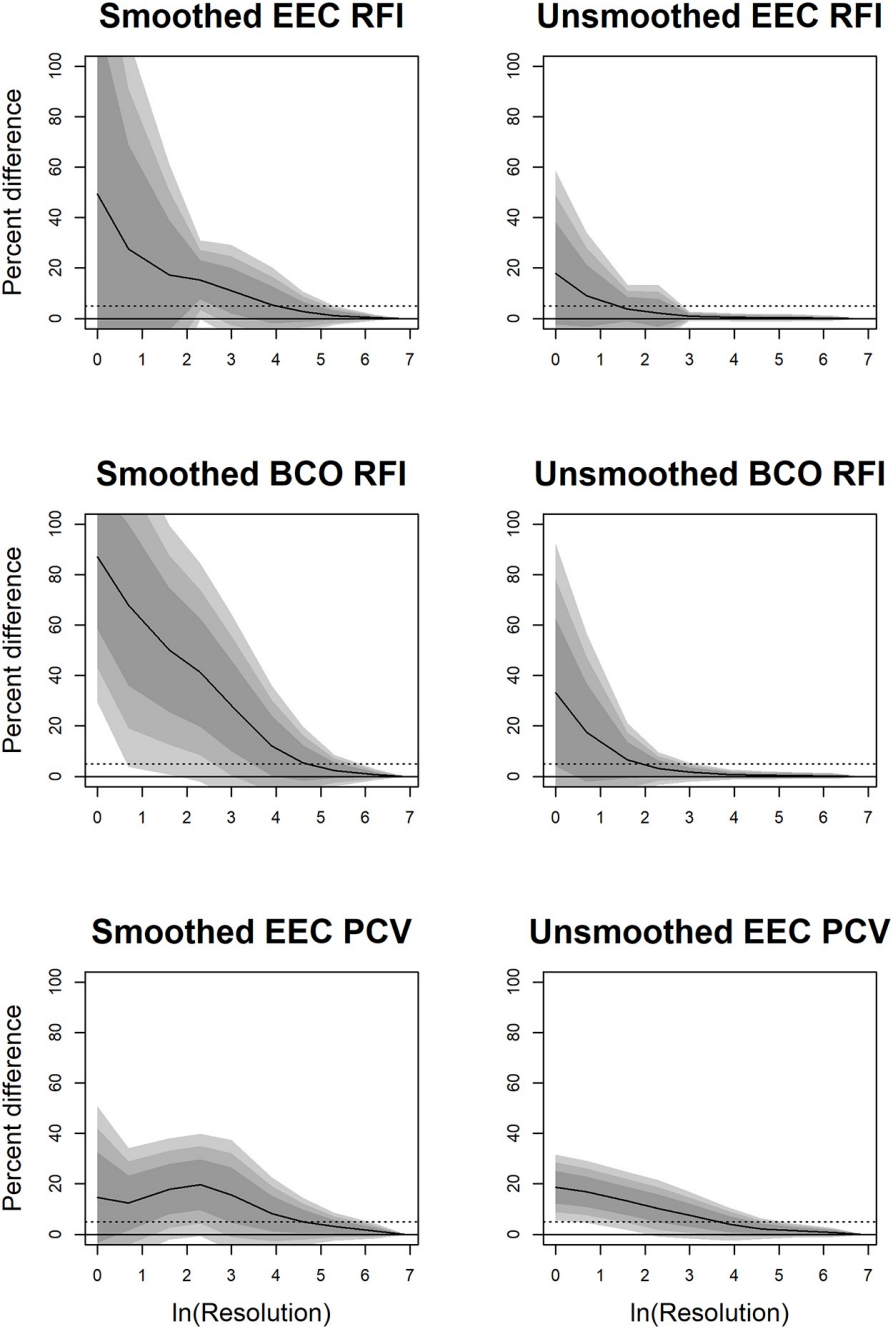
Absolute value of the percent difference between RFI and PCV values at the highest resolution and lower resolutions. Dotted horizontal lines represent 5% difference. The confidence intervals are drawn at 80%, 95%, and 99%.

Convergence tests were run to determine at what point RFI and/or PCV values converged, and therefore the minimum triangle count/resolution needed if surfaces with different triangle counts/resolutions were to be compared. We assumed the RFI/PCV value at the highest triangle count/resolution was the most accurate, and calculated the absolute value of the percent difference between that RFI/PCV and all other RFI/PCV values for that individual. RFI and PCV converge faster when triangle count is held constant compared to when resolution is held constant, and RFI and PCV values tend to converge faster in unsmoothed surfaces compared to smoothed surfaces (Figs 16 and 17). [31] noted the effects of smoothing on convergence of SA, and noted that SA steadily changes with the number of smoothing iterations, initially decreasing and then subsequently increasing. In the triangle count dataset, unsmoothed, BCO, RFI values converged faster than unsmoothed, EEC, RFI values, but smoothed, EEC, RFI values converged faster than smoothed, BCO, RFI values, suggesting cropping method may not have a consistent effect on convergence (Fig 16). In the resolution dataset, convergence does not occur in RFI when surfaces are smoothed until a resolution of 500 triangles/mm^2^ is reached. When surfaces are unsmoothed, convergence is reached at a resolution of 20 triangles/mm^2^. PCV reaches convergence for unsmoothed surfaces at a resolution of 200 triangles/mm^2^, but does not appear to converge when surfaces are smoothed (Fig 17).

When triangle count is held constant, a triangle count of 10000 is generally sufficient to obtain a converged RFI value, and therefore triangle count does not need to be held constant for RFI, as long as a triangle count of 10000 is reached. There is no triangle count at which PCV consistently converges, although the percent difference drops significantly at a triangle count of 50000. As such, we recommend triangle counts of 10000+ for RFI, and for triangle count to be held constant for DNE, OPCR, and PCV analyses. When resolution is held constant and surfaces are not smoothed, resolutions of 20+ and 200+ triangles/mm^2^ are recommended for RFI and PCV, respectively. When surfaces are unsmoothed, and for all cases in which DNE and OPCR are being measured, we recommend resolution be held constant.

It has been suggested that the rate of change in topographic metrics (specifically, OPCR) with respect to triangle count/resolution may be more informative than the value of a topographic metric at a single triangle count/resolution [45]. Since both the natural log of DNE and OPCR increase linearly with the natural log of triangle count and resolution in the same manner, we investigated the potential relationship between the slopes and intercepts of these linear relationships and diet. As stated in the materials and methods, slopes and intercepts were calculated for each specimen separately, using triangle counts 1000+ and resolutions 10+ triangles/mm^2^, and raw data is supplied in S8 Table.

**Fig 18:**
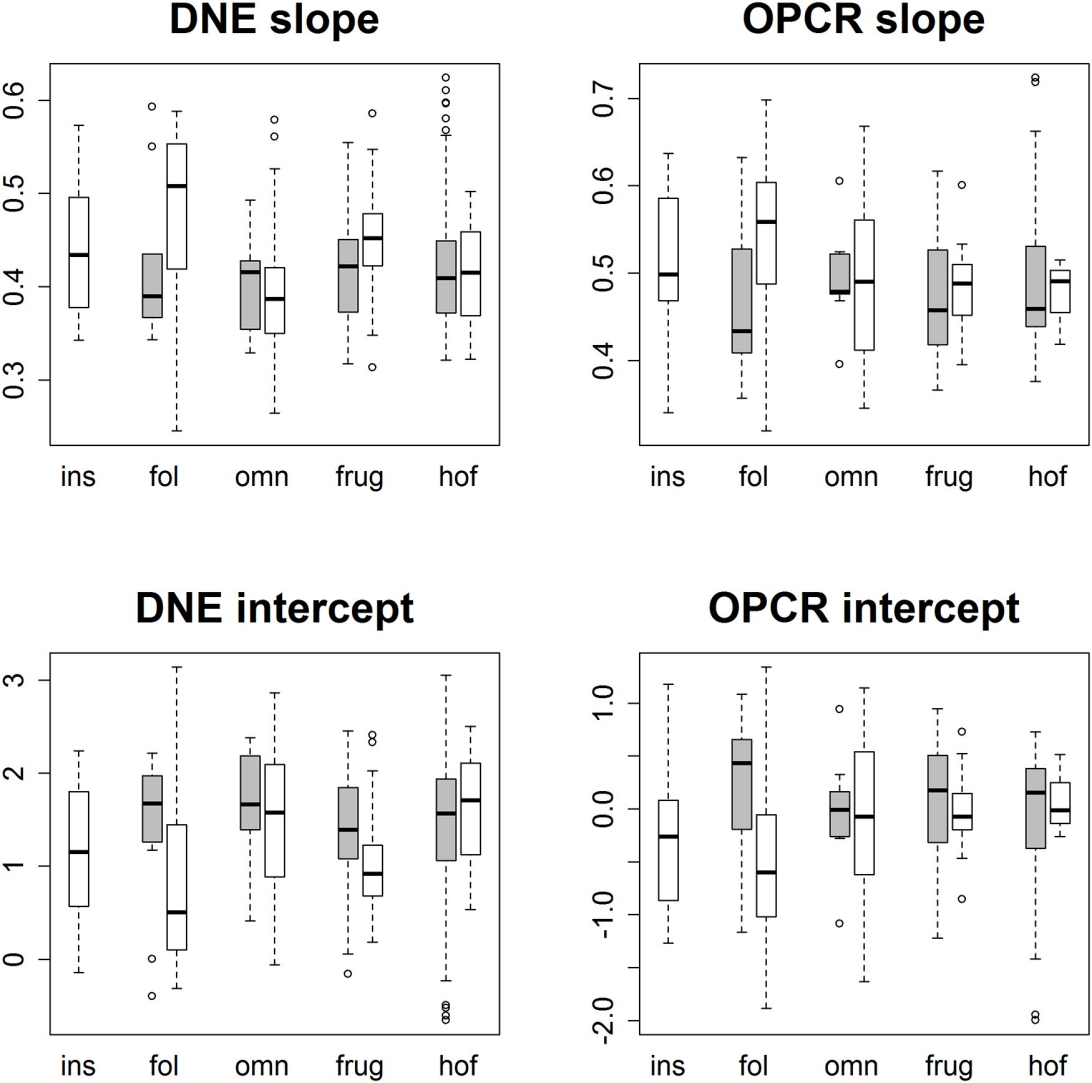
Boxplots showing slope and intercept values for DNE and OPCR regressed against triangle count (smoothed, EEC). Prosimians are in white and platyrrhines in grey. Diets are shown on the x-axis: ins = insectivore, fol = folivore, omn = omnivore, frug = frugivore, and hof = hard object feeder.

**Fig 19:**
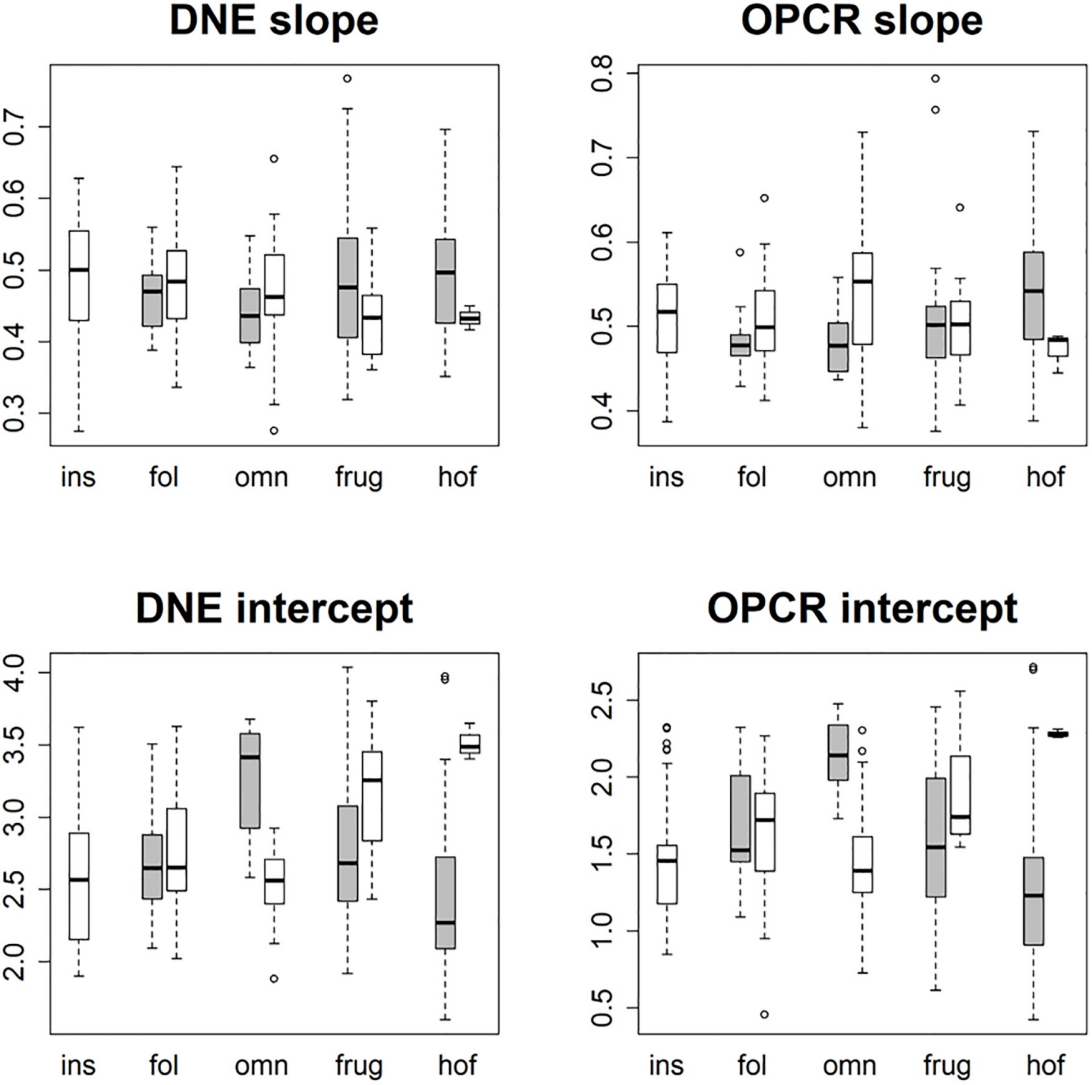
Boxplots showing slope and intercept values for DNE and OPCR regressed against resolution (smoothed, EEC). Prosimians are in white and platyrrhines in grey. Diets are shown on the x-axis: ins = insectivore, fol = folivore, omn = omnivore, frug = frugivore, and hof = hard object feeder.

Boxplots of slope and intercept data for smoothed surfaces cropped using EEC are shown in Figs 18 and 19; other boxplots are in S4 Fig. The boxplots suggest there is little dietary difference in slope or intercept for DNE or OPCR: this is supported by one-way ANOVAs, Tukey HSD tests, and linear DFAs, run using the same standards as previously. Results for these tests are presented in the Supplementary Information (S9 Table), and not presented here.

To determine if there existed an “optimal” triangle count/resolution to perform dental topography at, the p-values from the Tukey HSD tests and the linear DFAs were plotted against triangle count and resolution. The p-values for the linear DFAs obtained for the slopes and the intercepts of DNE and OPCR being regressed against triangle count and resolution are plotted on these graphs as horizontal lines, so the predictive ability of slope and intercept at predicting diet could be compared to the predictive ability of the other methods.

**Fig 20:**
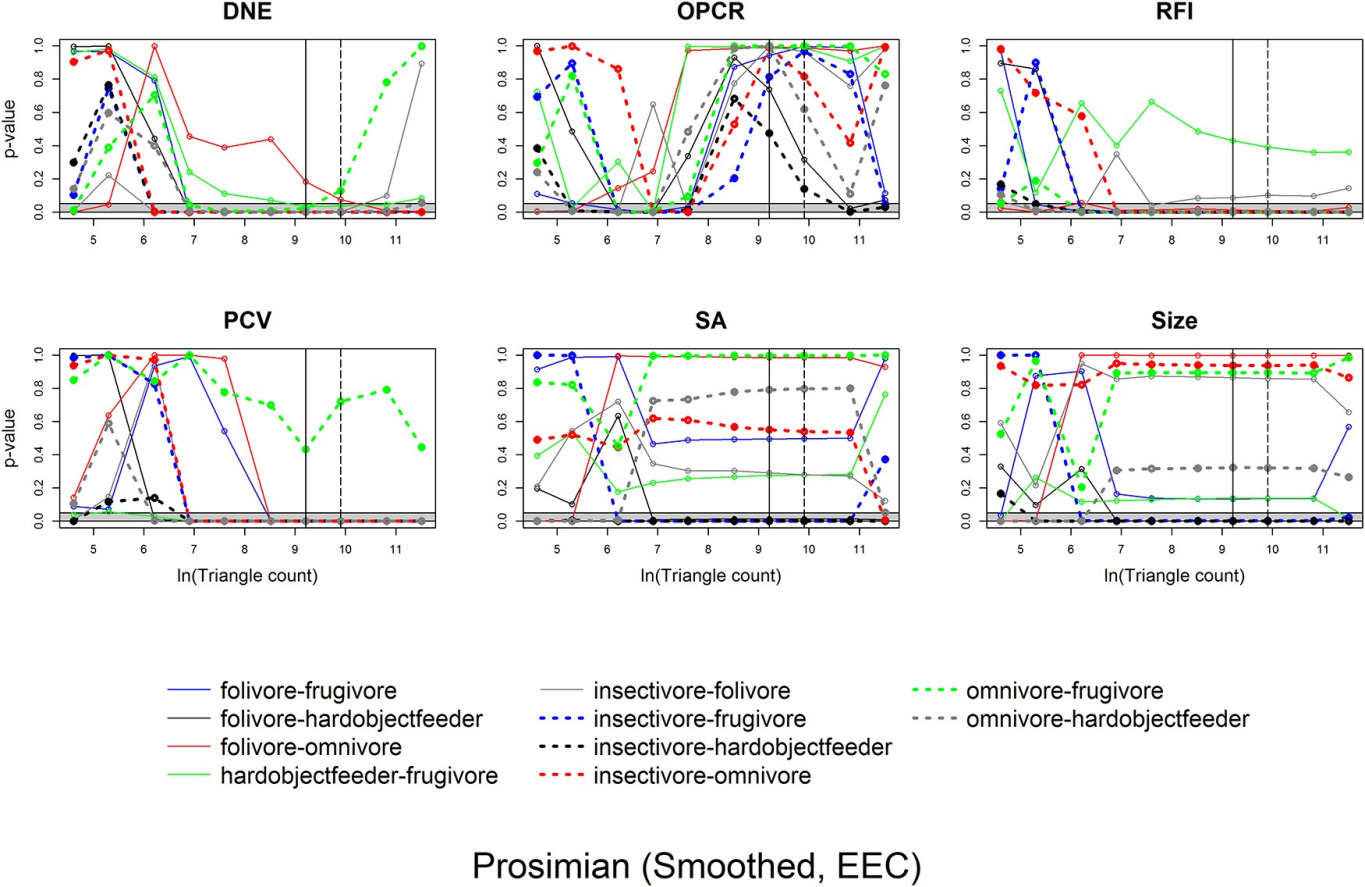
Changes in p-values from Tukey HSD tests plotted against triangle count (prosimian, smoothed, EEC). The grey shaded regions at the bottom of the graphs show p-values from 0–0.05. Vertical solid and dashed lines indicate triangle counts of 10000 and 20000, respectively.

**Fig 21:**
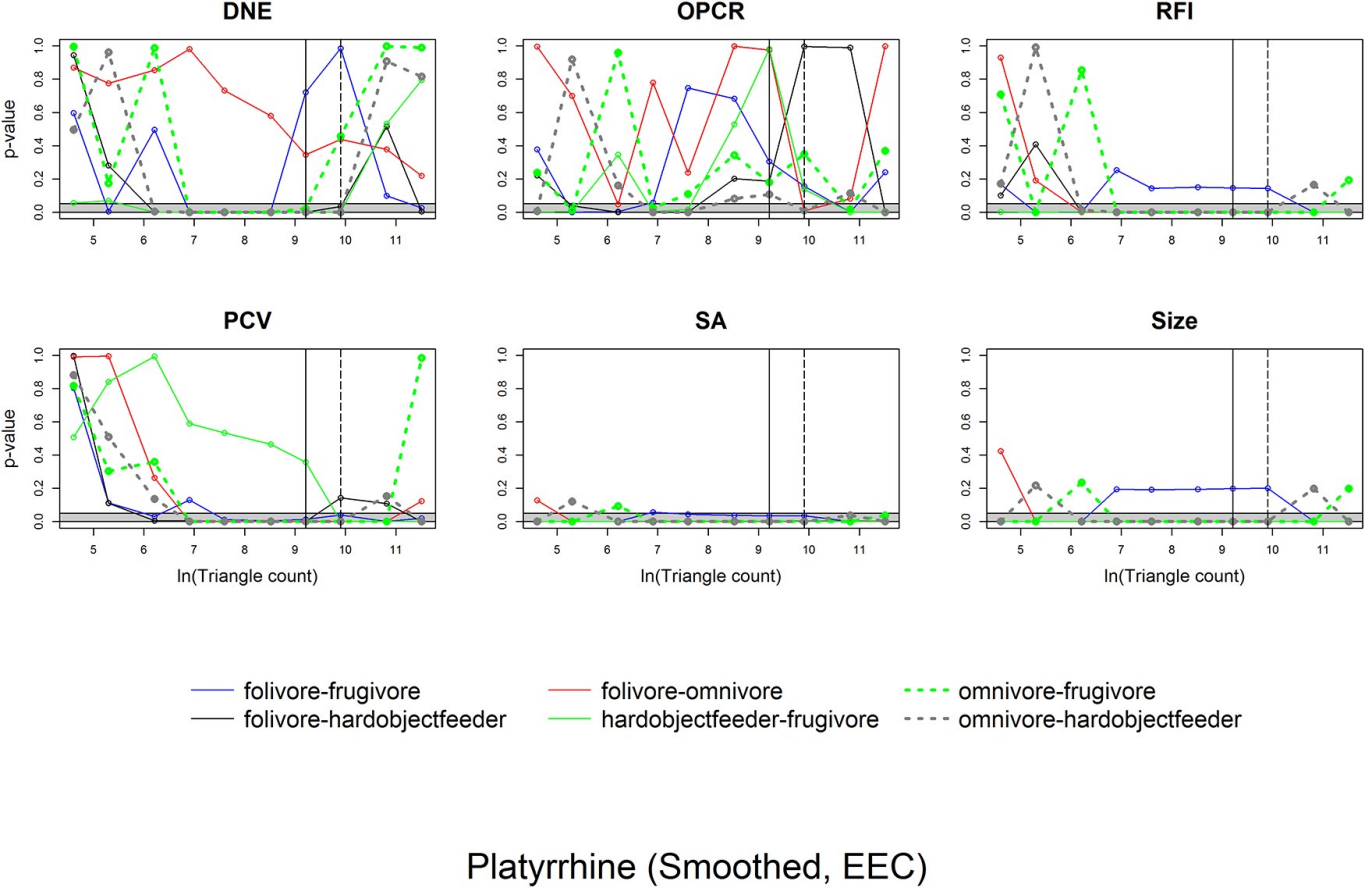
Changes in p-values from Tukey HSD tests plotted against triangle count (platyrrhine, smoothed, EEC). The grey shaded regions at the bottom of the graphs show p-values from 0–0.05. Vertical solid and dashed lines indicate triangle counts of 10000 and 20000, respectively.

**Fig 22:**
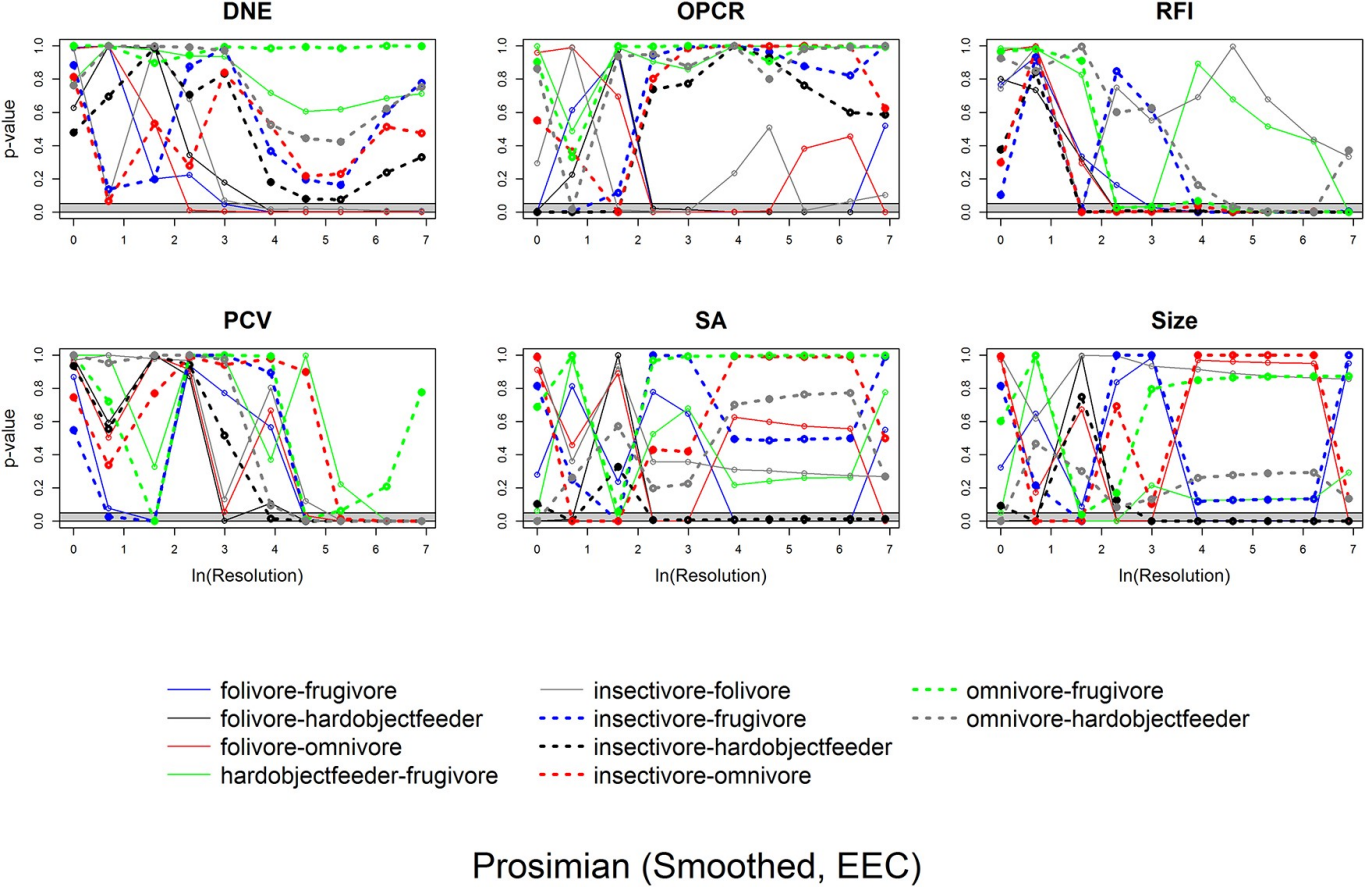
Changes in p-values from Tukey HSD tests plotted against resolution (prosimian, smoothed, EEC). The grey shaded regions at the bottom of the graphs show p-values from 0–0.05.

**Fig 23:**
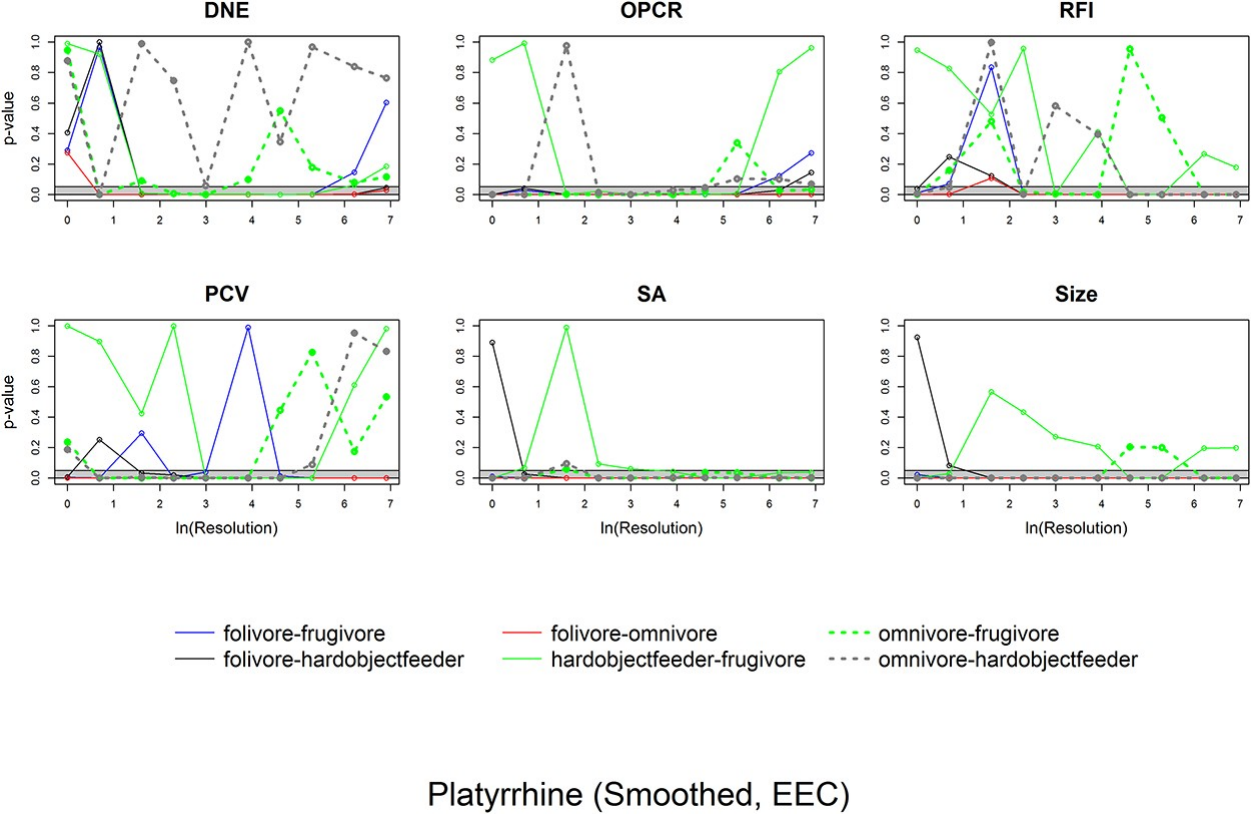
Changes in p-values from Tukey HSD tests plotted against resolution (platyrrhine, smoothed, EEC). The grey shaded regions at the bottom of the graphs show p-values from 0–0.05.

Graphs for the p-values from the Tukey HSD tests for smoothed, EEC are presented here (Figs 20, 21, 22, and 23). All other graphs are in the Supplementary Information (S5 Fig). In prosimians, when triangle count is held constant, DNE performs best at triangle counts around 10,000–20,000. Above 20,000, the previously significant differences between omnivores/frugivores and insectivores/folivores loses significance. In general, OPCR consistently does a poor job at differentiating between diets at triangle counts above 1000. There is no improvement in the performance of RFI, PCV, SA, and size once a triangle count of 20000 is reached (Fig 20). In platyrrhines, DNE and OPCR tend to perform worse than the other topographic metrics, and perform better at triangle counts between 1000 and 5000 than at either higher or lower triangle counts. PCV, RFI, SA and size tend to perform better than OPCR and DNE. Interestingly, SA tends to consistently perform the best of all the variables (Fig 21). This is not a variable that has been investigated as a dietary correlate in primates, although differences in the surface area of the tooth roots indicate dietary specializations well [64–66]. When resolution is held constant, all topographic metrics tend to perform very poorly in prosimians. DNE and OPCR tend to perform better than when triangle count is held constant within platyrrhines, but RFI, PCV, and size tend to perform worse at high resolutions.

**Fig 24:**
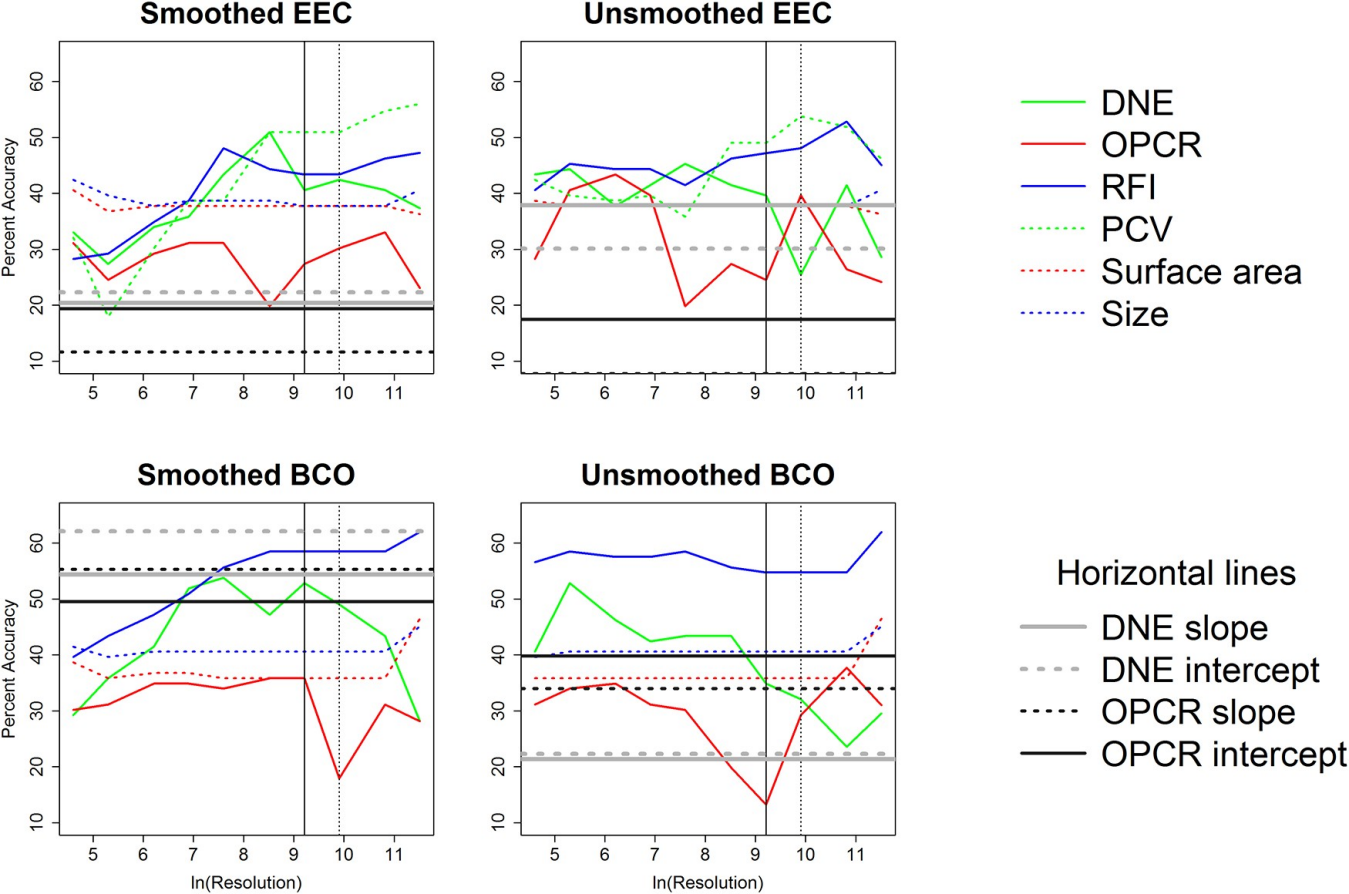
Changes in p-values from linear DFAs plotted against triangle count (prosimians). Horizontal lines represent p-values from linear DFAs constructed from the slopes/intercepts of DNE/OPCR being regressed against triangle count. Vertical solid and dashed lines indicate triangle counts of 10000 and 20000, respectively.

**Fig 25:**
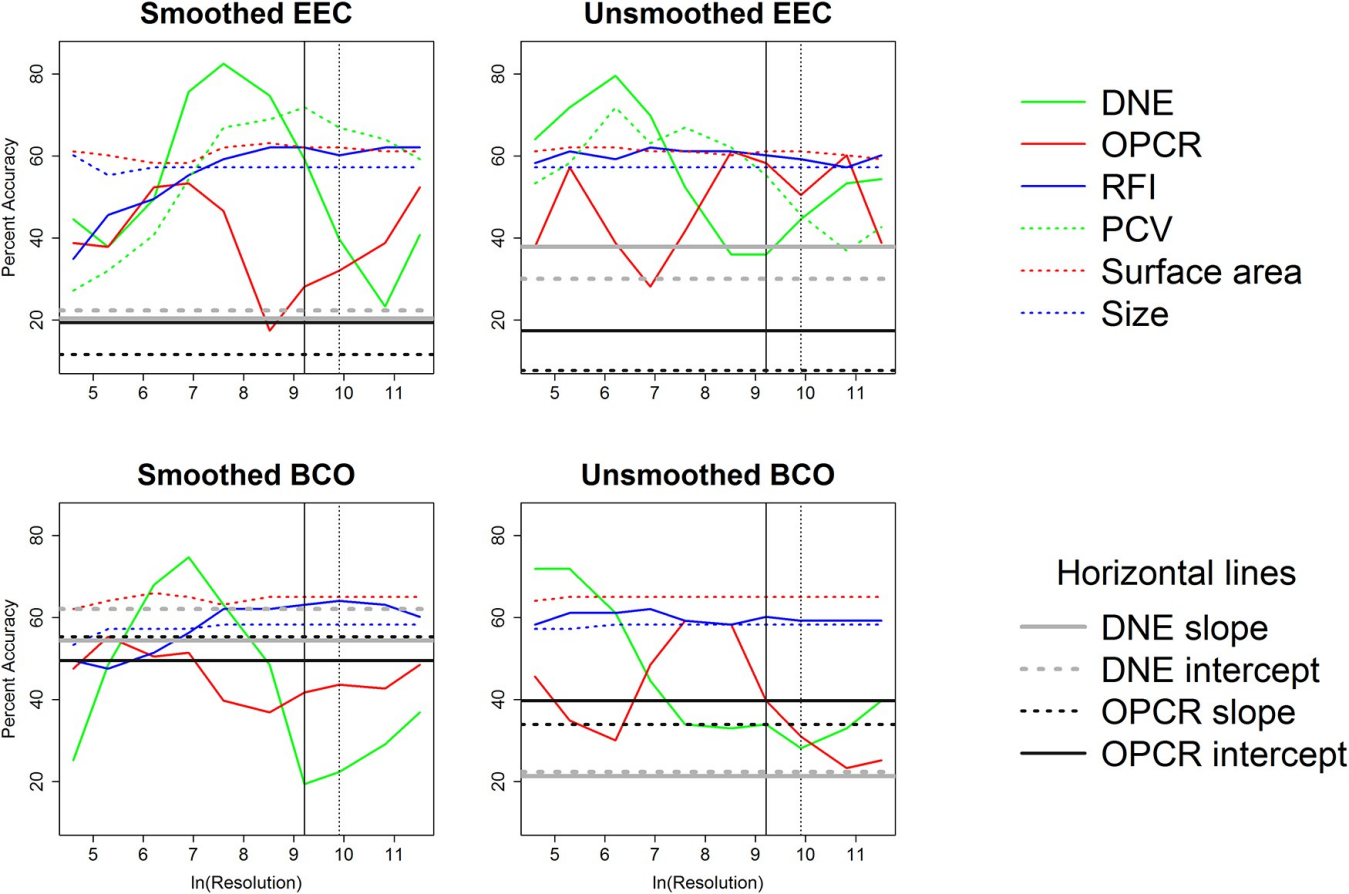
Changes in p-values from linear DFAs plotted against triangle count (platyrrhines). Horizontal lines represent p-values from linear DFAs constructed from the slopes/intercepts of DNE/OPCR being regressed against triangle count. Vertical solid and dashed lines indicate triangle counts of 10000 and 20000, respectively.

**Fig 26:**
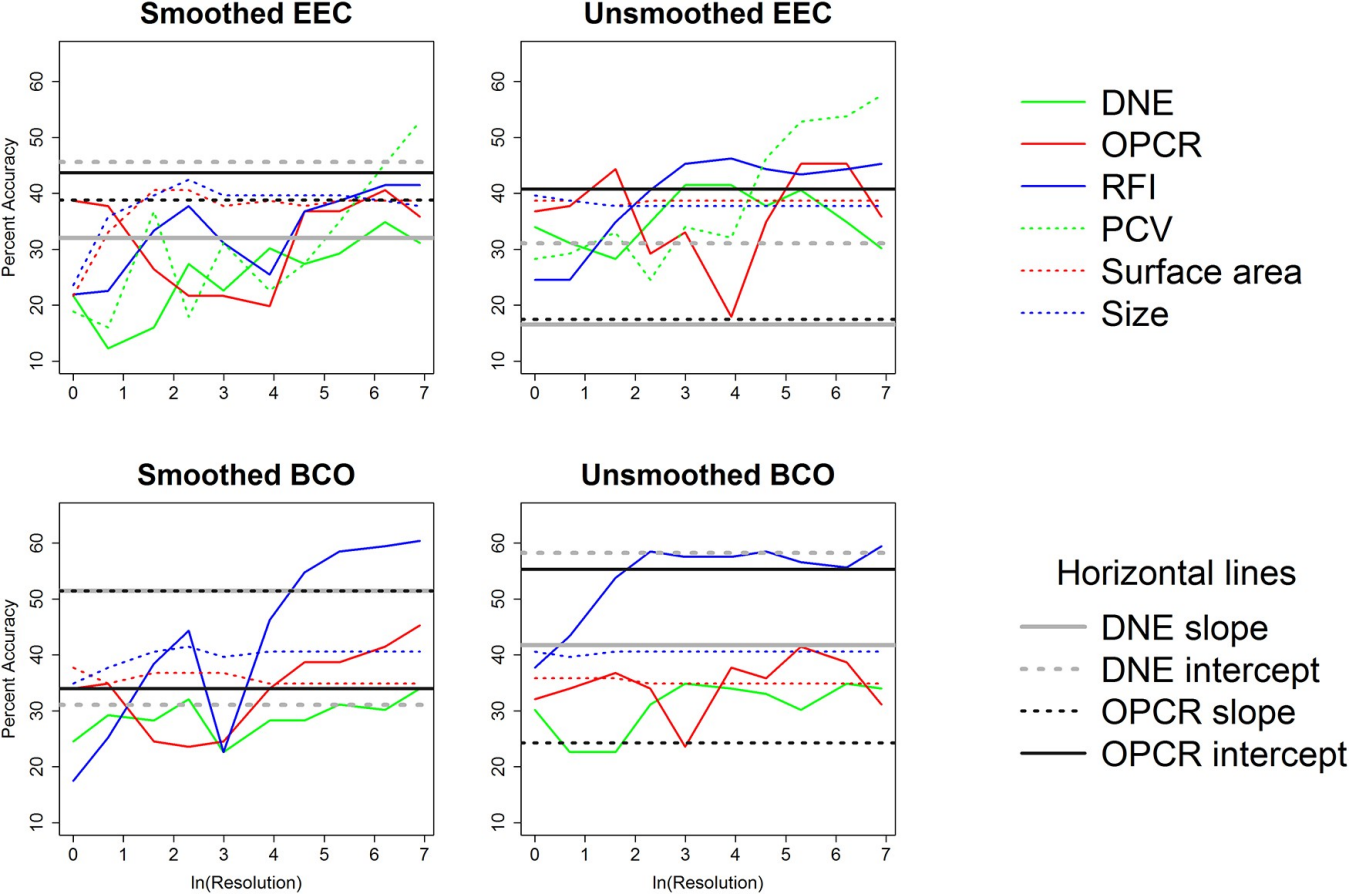
Changes in p-values from linear DFAs plotted against resolution (prosimians). Horizontal lines represent p-values from linear DFAs constructed from the slopes/intercepts of DNE/OPCR being regressed against resolution.

**Fig 27:**
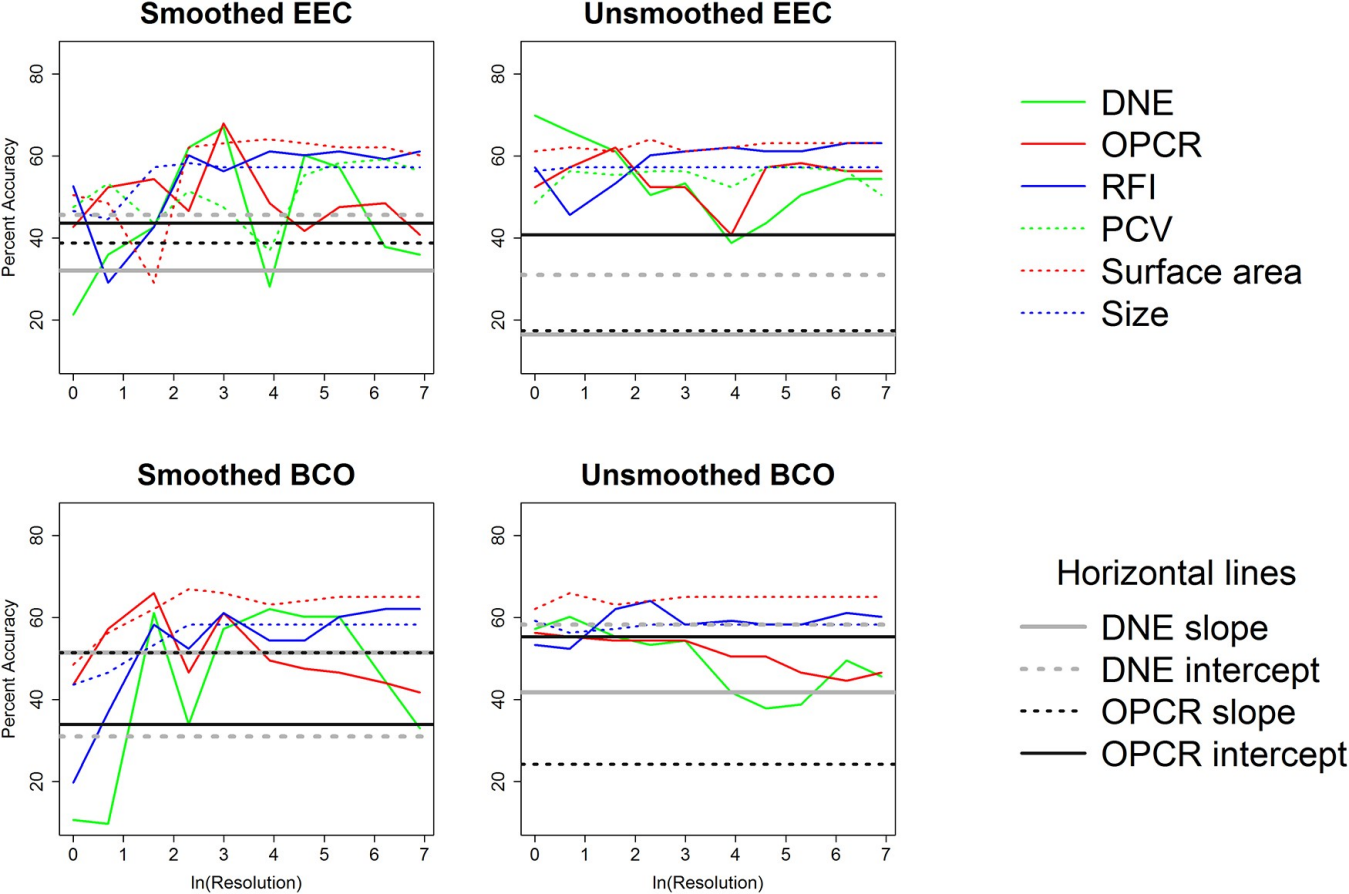
Changes in p-values from linear DFAs plotted against resolution (platyrrhines). Horizontal lines represent p-values from linear DFAs constructed from the slopes/intercepts of DNE/OPCR being regressed against resolution.

Graphs for the *p*- values for the linear DFAs are presented in Figs 24, 25, 26, and 27. In general, there are not large changes in p-value with triangle count/resolution, and the predictive ability of the different topographic parameters changes sporadically. The exception is of course SA, size, and occasionally RFI, as these metrics tend to converge on a single value.

Overall, these results do not support the idea that there is an “optimal” triangle count or resolution for performing dental topographic studies: triangle count and resolution should be high enough to properly represent the surface, and triangle count/resolution should be held constant for DNE, OPCR, and PCV. It also appears it may be beneficial to keep triangle count, and not resolution, constant in dental topographic studies.

## Conclusion

All topographic metrics are sensitive to smoothing, cropping method, and triangle count/resolution. In general, smoothing causes DNE, OPCR, RFI, and SA to decrease, PCV to increase, and is just as likely to cause size to increase or decrease. Relative to the BCO cropping method, EEC causes RFI, SA, and size to increase, and is just as likely to cause DNE and OPCR to increase or decrease, although DNE is slightly more likely to increase. Topographic metrics are more sensitive to smoothing and cropping at low triangle counts compared to high. There is a positive, logarithmic correlation between DNE and OPCR and triangle count/resolution, and the slope/intersection of the regressions between the natural log of DNE/OPCR and triangle count/resolution is no better at predicting diet than topographic values at a constant triangle count/resolution. PCV tends to converge to a constant value or decrease with increases in triangle count/resolution, and RFI, SA, and size converge to a constant value as triangle count/resolution increases. When resolution is held constant, topographic variables are more correlated to each other than when triangle count is held constant. There appears to be no optimal triangle count or resolution for predicting diet, and diet appears to be more correlated to topographic metrics when triangle count, and not resolution, is held constant.

### Summary recommendations for smoothing, cropping, and triangle count/resolution

If a dataset exists with a combination of smoothed and unsmoothed surfaces, DNE and OPCR should not be compared. Size can be directly compared between smoothed and unsmoothed surfaces, and no transformation is needed to compare RFI, SA, or PCV. We do not recommend comparing any topographic metrics gathered using the different cropping methods (BCO vs. EEC), but have provided transformation equations in the supplementary material should the original material be inaccessible. We suggest triangle count, and not resolution, be held constant as it tends to yield more desirable results. Further, triangle count should be high enough to digitally represent the entire tooth accurately, and there appears to be no ideal smoothing technique, cropping method, or triangle count for performing topographic analyses. Therefore, all these factors should be held constant in each study.

## Supporting information

S1 Table All data dental topography. Raw data for analyses.
(XLSX)

S2 Table Five-way ANOVA results. Results from five-way ANOVA.
(XLSX)

S3 Table Transformation equations. Transformation equations for smoothing and cropping.
(XLSX)

S4 Table Descriptive statistics. Descriptive statistics for data.
(XLSX)

S5 Table One-way ANOVA results. Results from one-way ANOVAs.
(XLSX)

S6 Table Tukey HSD results. Results for Tukey HSD analyses.
(XLSX)

S7 Table Correlations among metrics. Correlations between topographic variables.
(XLSX)

S8 Table Slopes and intercepts. Slopes and intercepts of DNE and OPCR vs. triangle count and resolution.
(XLSX)

S9 Table Statistics for slopes and intercepts. ANOVAS, Tukey HSDs, and DFAs for intercepts and slopes.
(XLSX)

S1 Fig Boxplots. 880 boxplots for topographic values.
(PPTX)

S2 Fig Tukey HSD visualization. Tukey HSD visualized for triangle count and resolution.
(PPTX)

S3 Fig Linear plots. Linear plots, effect of triangle count and resolution on topographic variables.
(PPTX)

S4 Fig Boxplots for slopes and intercepts. . Boxplots for DNE and OPCR slopes and intercepts vs. diet.
(PPTX)

S5 Fig Tukey HSD visualization. Graphical representations of Tukey HSD results.
(PPTX)

## Abbreviations

BCO: basin cut off
DNE: Dirichlet normal energy
EEC: entire enamel cap
MicroCT: microcomputed tomography
OPCR: orientation patch count rotated
PCV: ambient occlusion (portion de ciel visible or “portion of visible sky”)
RFI: relief index
SA: surface area
