# Supplementary material for: Effects of cropping, smoothing, triangle count, and mesh resolution on 6 dental topographic metrics
Source: PLoS One. 2019 May 6;14(5):e0216229. doi: 10.1371/journal.pone.0216229 (PMC6502444; doi:10.1371/journal.pone.0216229)
Supplement: S1 Fig — 880 boxplots for topographic values. (PPTX) [file pone.0216229.s010.pptx]

## Slide 1
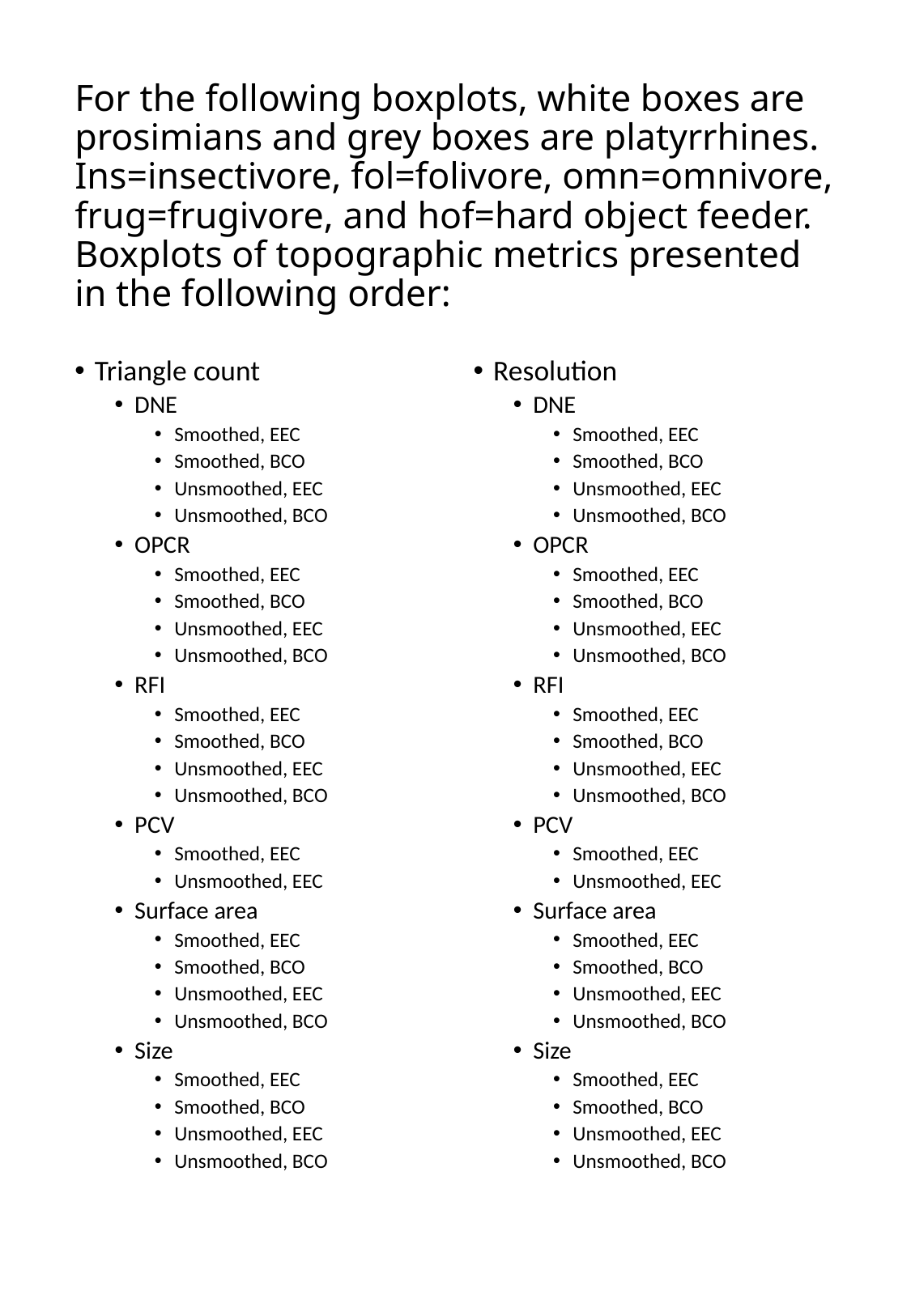

# For the following boxplots, white boxes are prosimians and grey boxes are platyrrhines. Ins=insectivore, fol=folivore, omn=omnivore, frug=frugivore, and hof=hard object feeder. Boxplots of topographic metrics presented in the following order:
Triangle count
DNE
Smoothed, EEC
Smoothed, BCO
Unsmoothed, EEC
Unsmoothed, BCO
OPCR
Smoothed, EEC
Smoothed, BCO
Unsmoothed, EEC
Unsmoothed, BCO
RFI
Smoothed, EEC
Smoothed, BCO
Unsmoothed, EEC
Unsmoothed, BCO
PCV
Smoothed, EEC
Unsmoothed, EEC
Surface area
Smoothed, EEC
Smoothed, BCO
Unsmoothed, EEC
Unsmoothed, BCO
Size
Smoothed, EEC
Smoothed, BCO
Unsmoothed, EEC
Unsmoothed, BCO
Resolution
DNE
Smoothed, EEC
Smoothed, BCO
Unsmoothed, EEC
Unsmoothed, BCO
OPCR
Smoothed, EEC
Smoothed, BCO
Unsmoothed, EEC
Unsmoothed, BCO
RFI
Smoothed, EEC
Smoothed, BCO
Unsmoothed, EEC
Unsmoothed, BCO
PCV
Smoothed, EEC
Unsmoothed, EEC
Surface area
Smoothed, EEC
Smoothed, BCO
Unsmoothed, EEC
Unsmoothed, BCO
Size
Smoothed, EEC
Smoothed, BCO
Unsmoothed, EEC
Unsmoothed, BCO

## Slide 2
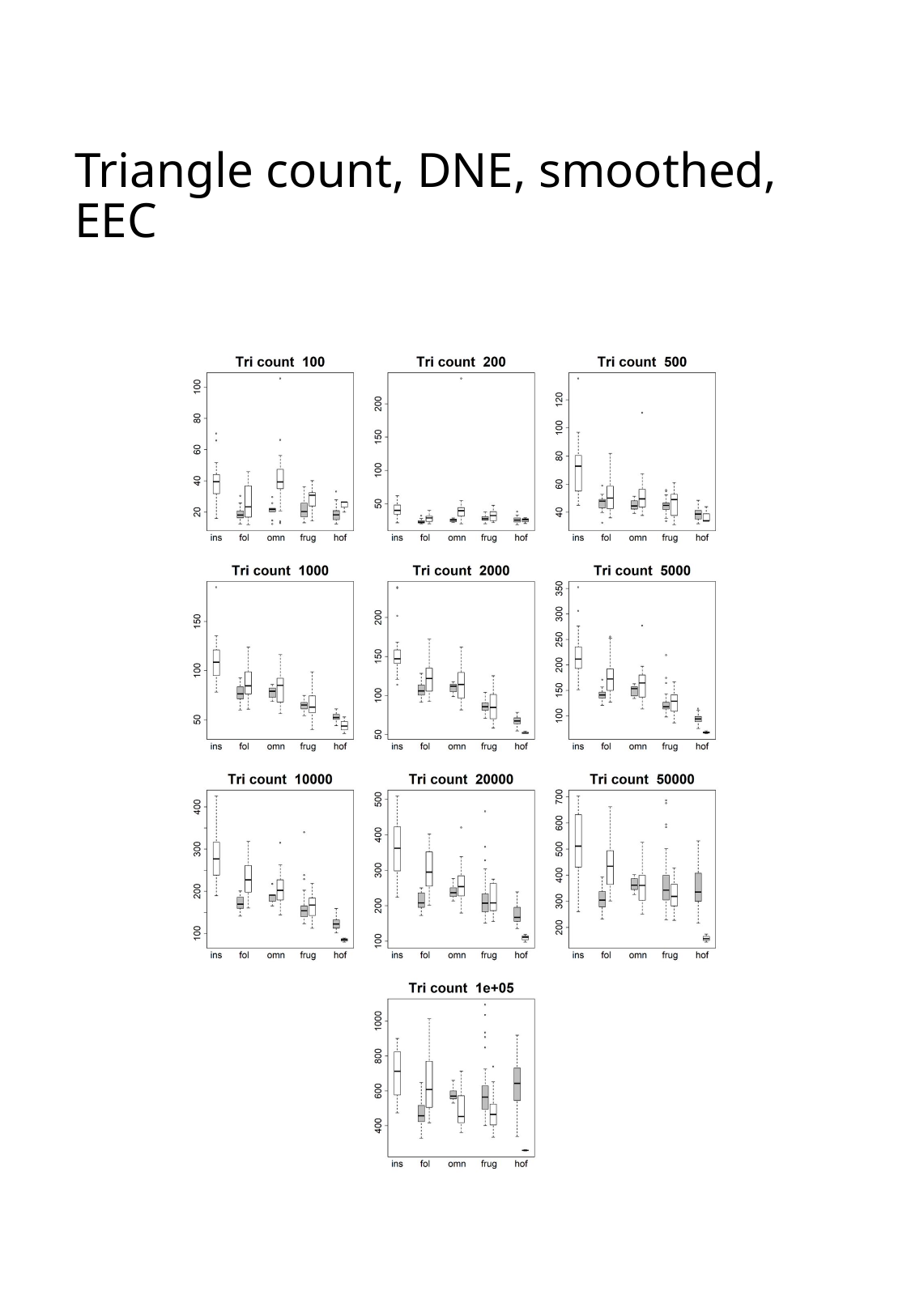

# Triangle count, DNE, smoothed, EEC

## Slide 3
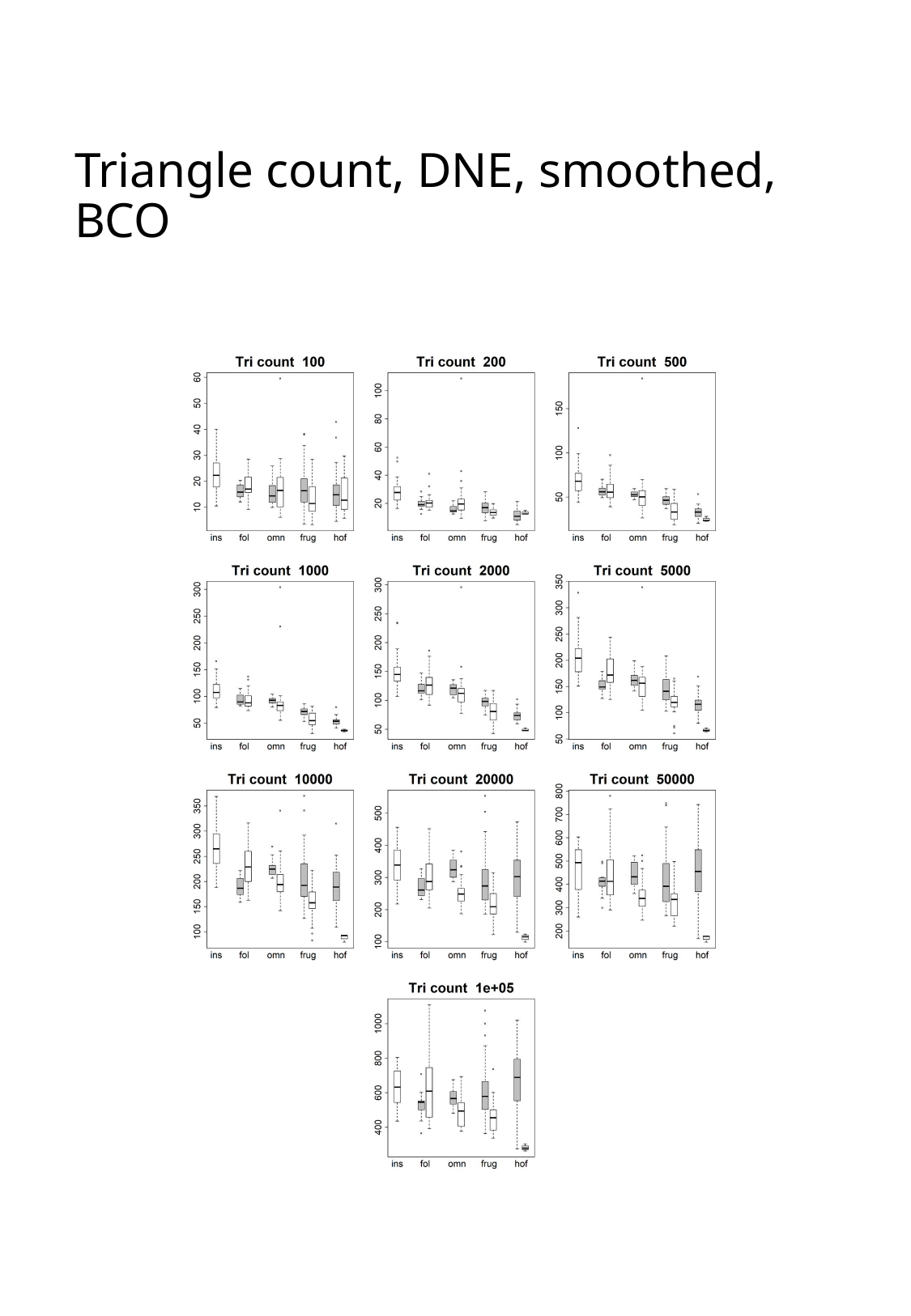

# Triangle count, DNE, smoothed, BCO

## Slide 4
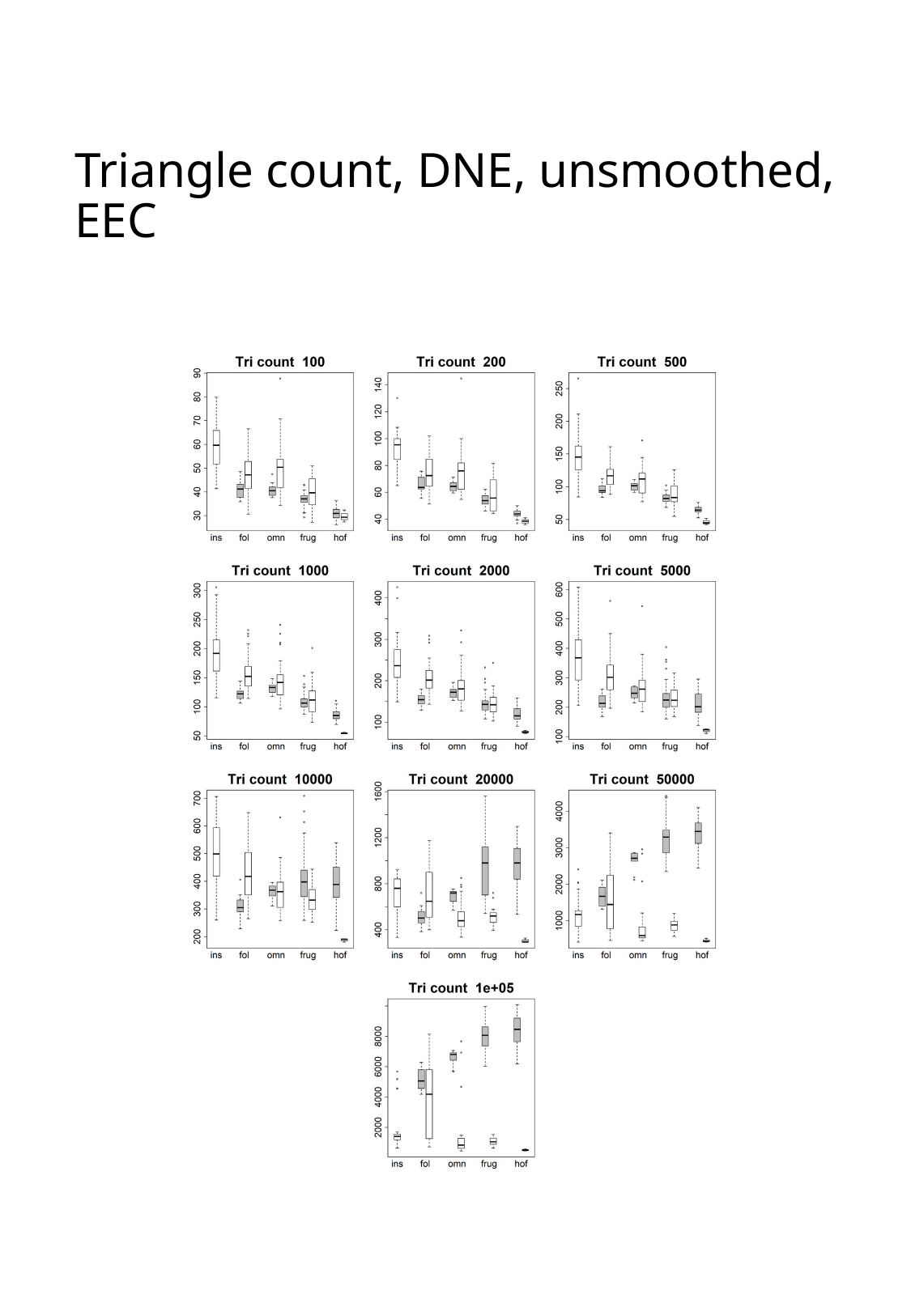

# Triangle count, DNE, unsmoothed, EEC

## Slide 5
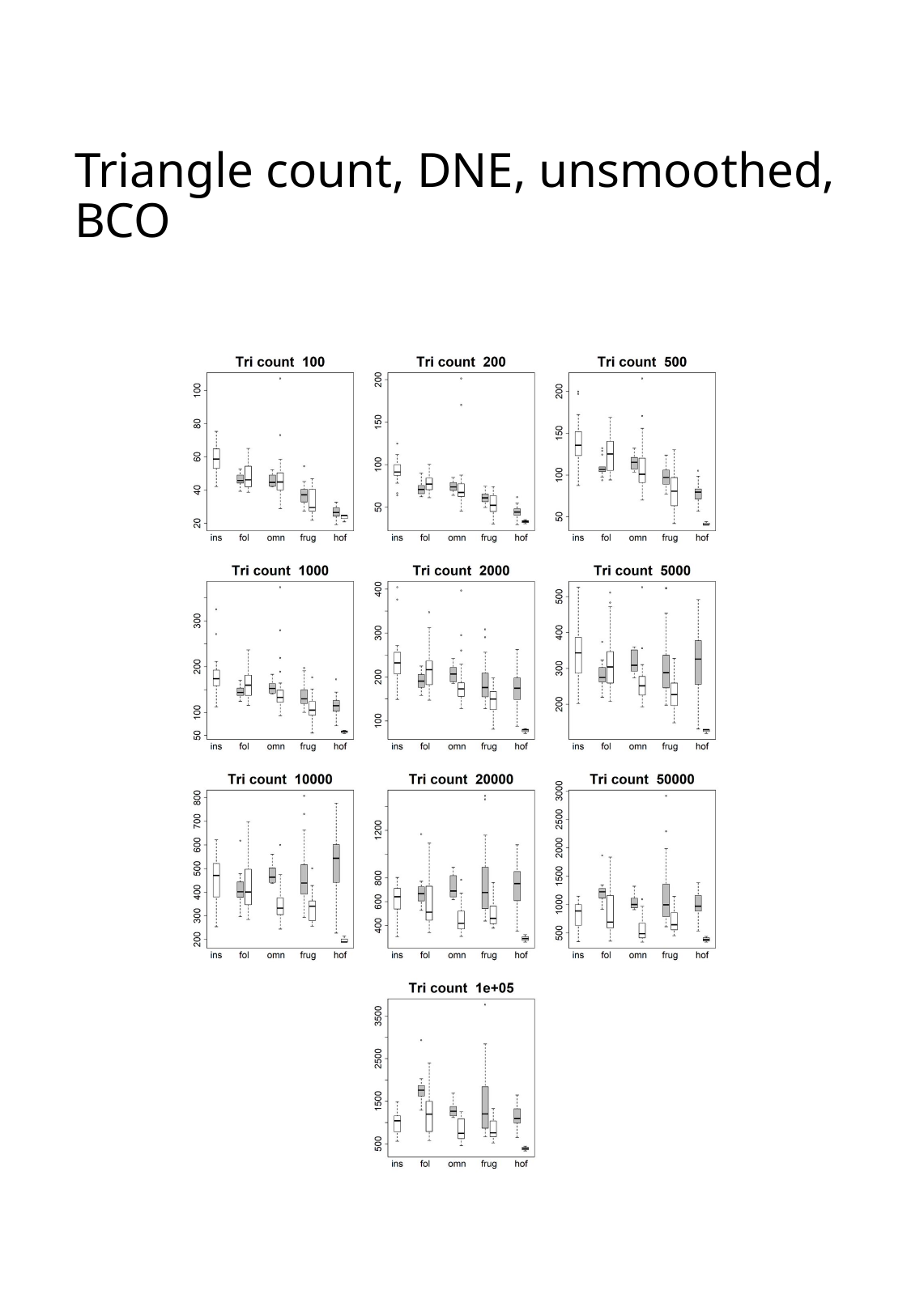

# Triangle count, DNE, unsmoothed, BCO

## Slide 6
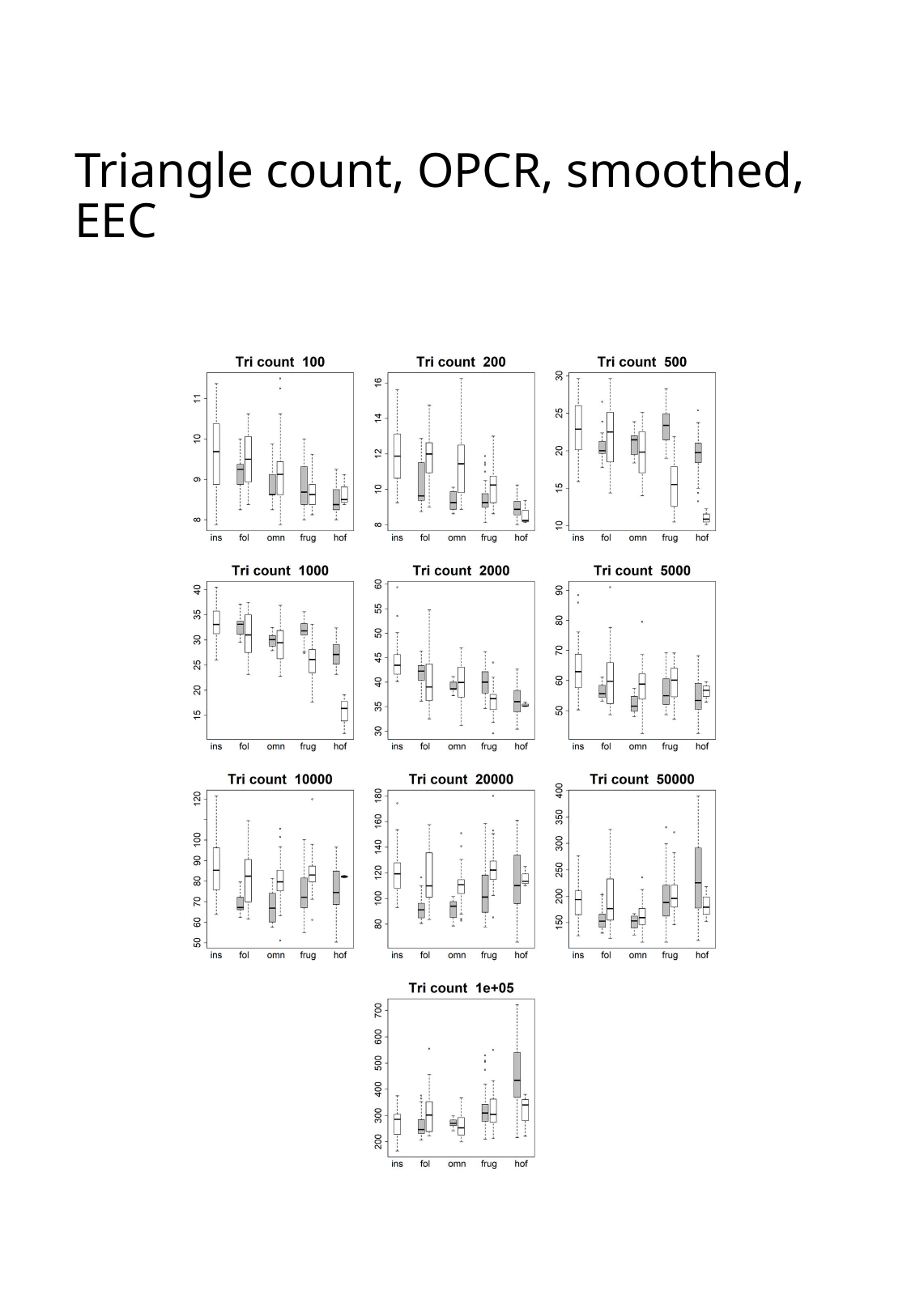

# Triangle count, OPCR, smoothed, EEC

## Slide 7
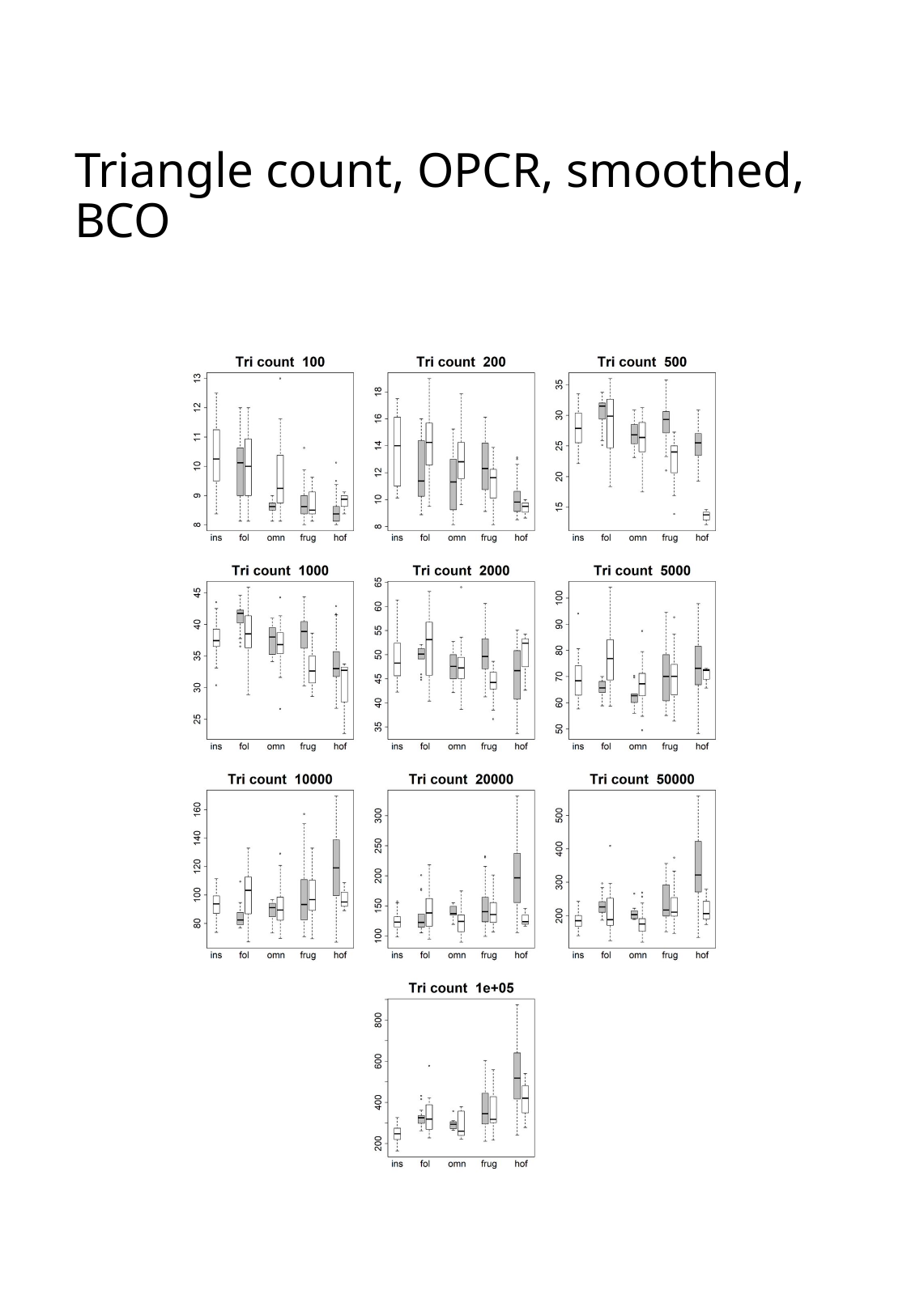

# Triangle count, OPCR, smoothed, BCO

## Slide 8
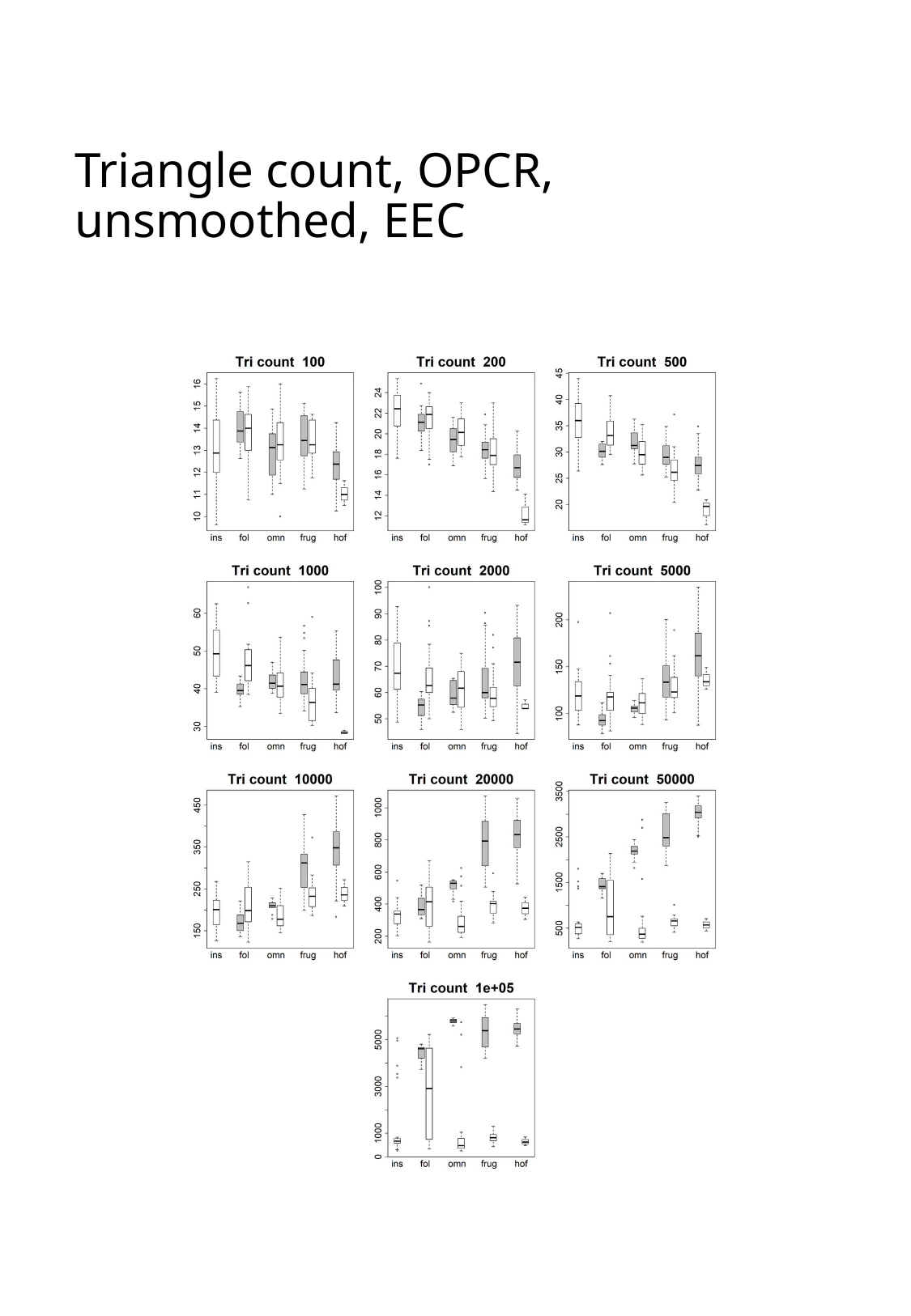

# Triangle count, OPCR, unsmoothed, EEC

## Slide 9
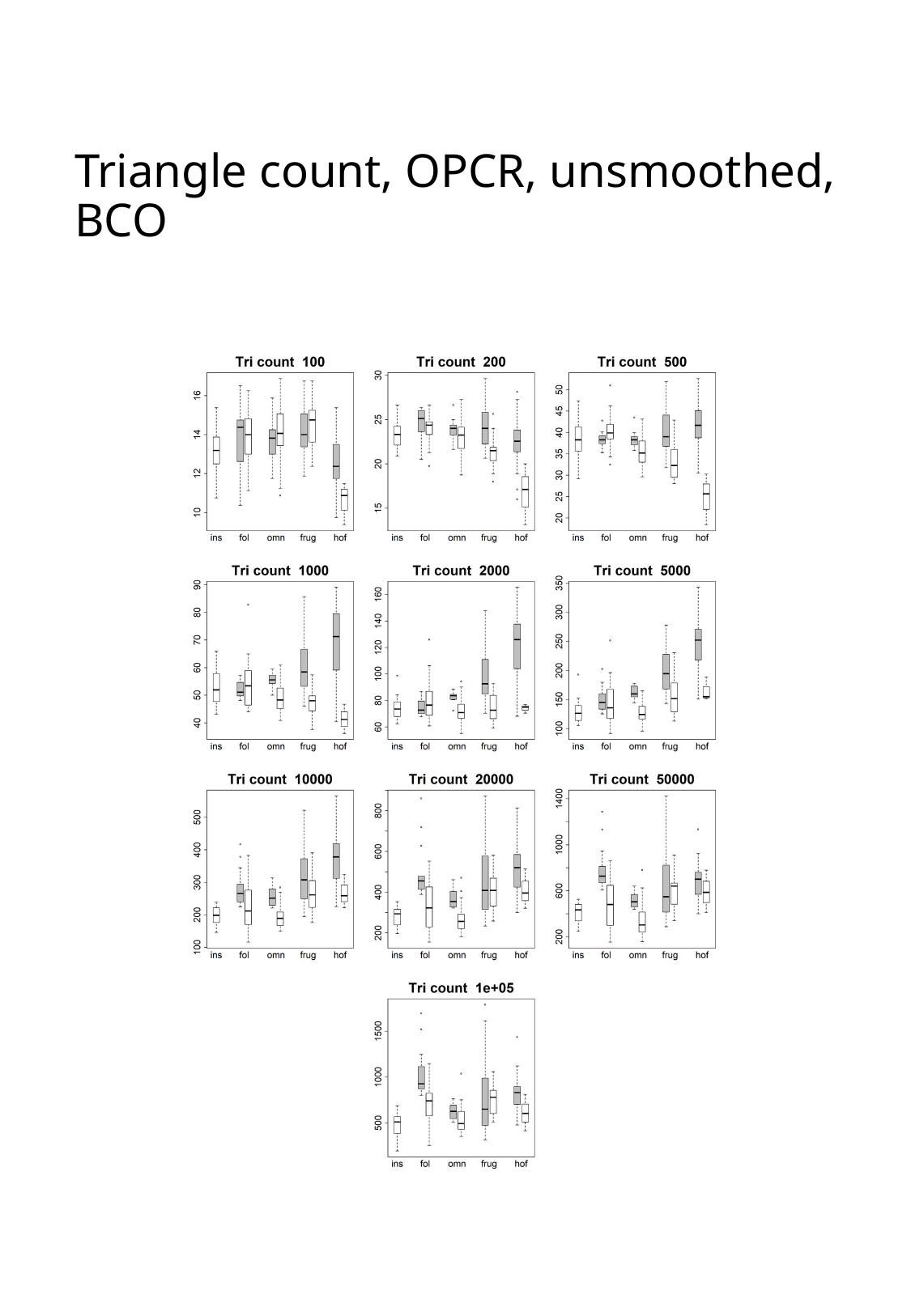

# Triangle count, OPCR, unsmoothed, BCO

## Slide 10
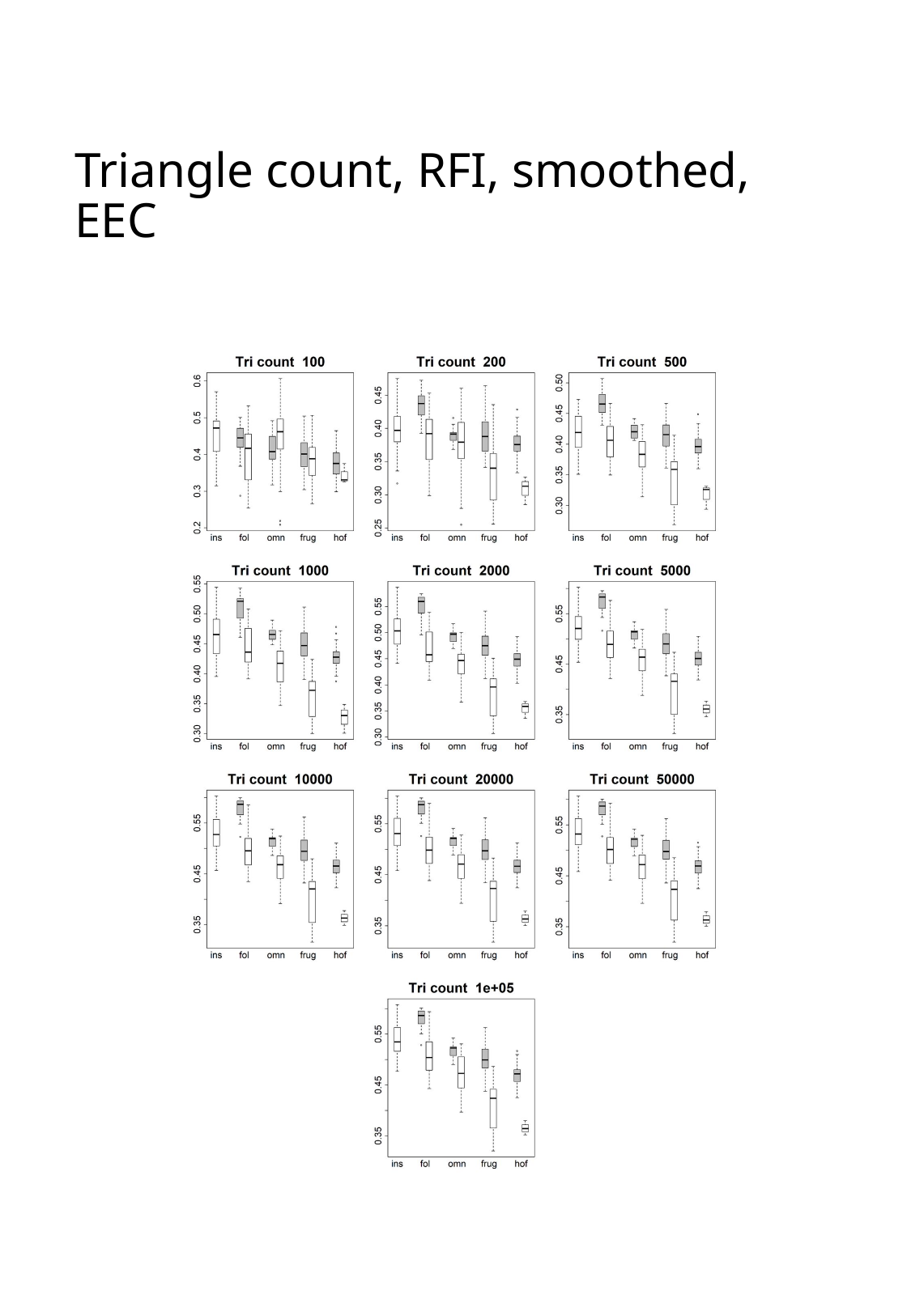

# Triangle count, RFI, smoothed, EEC

## Slide 11
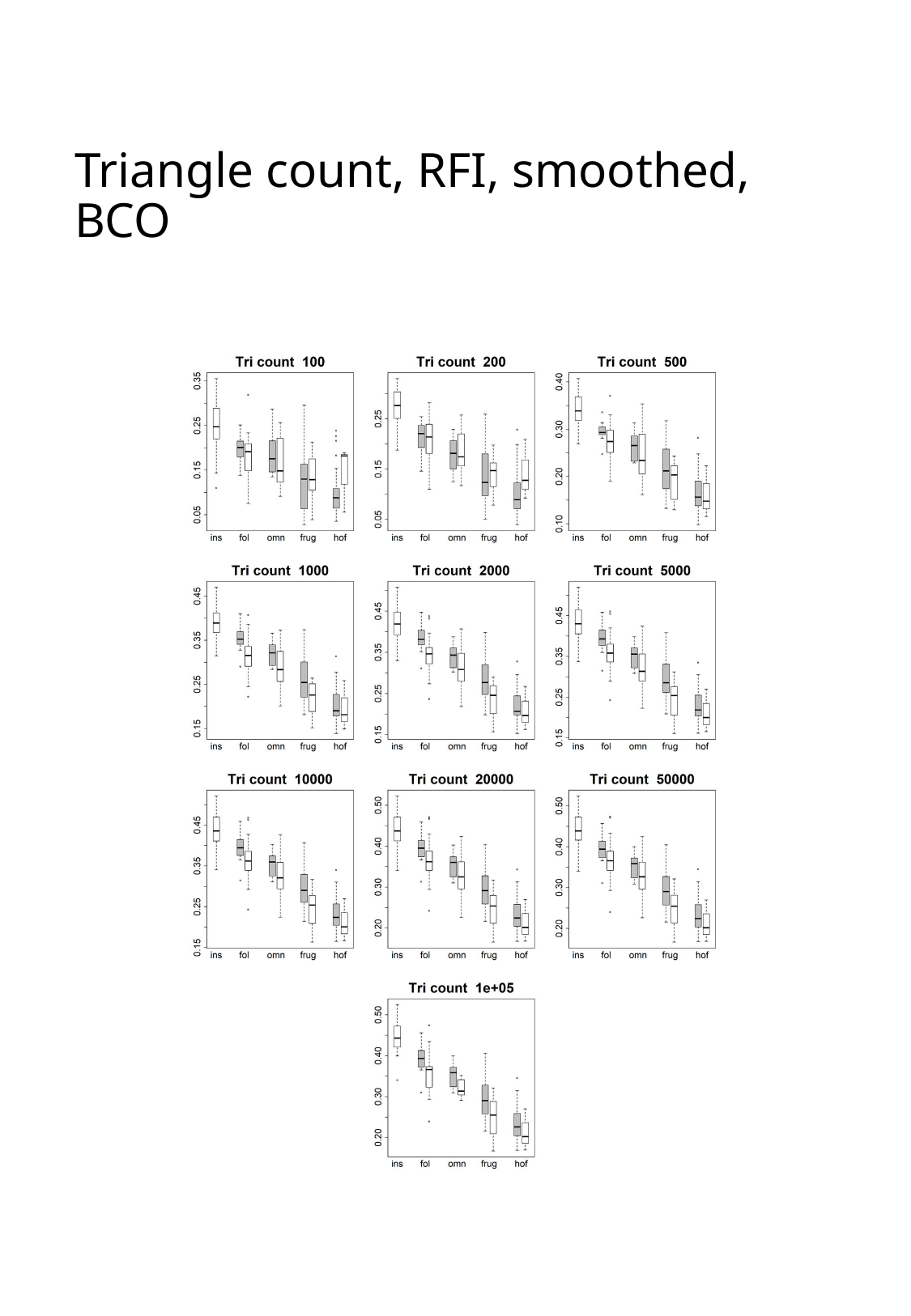

# Triangle count, RFI, smoothed, BCO

## Slide 12
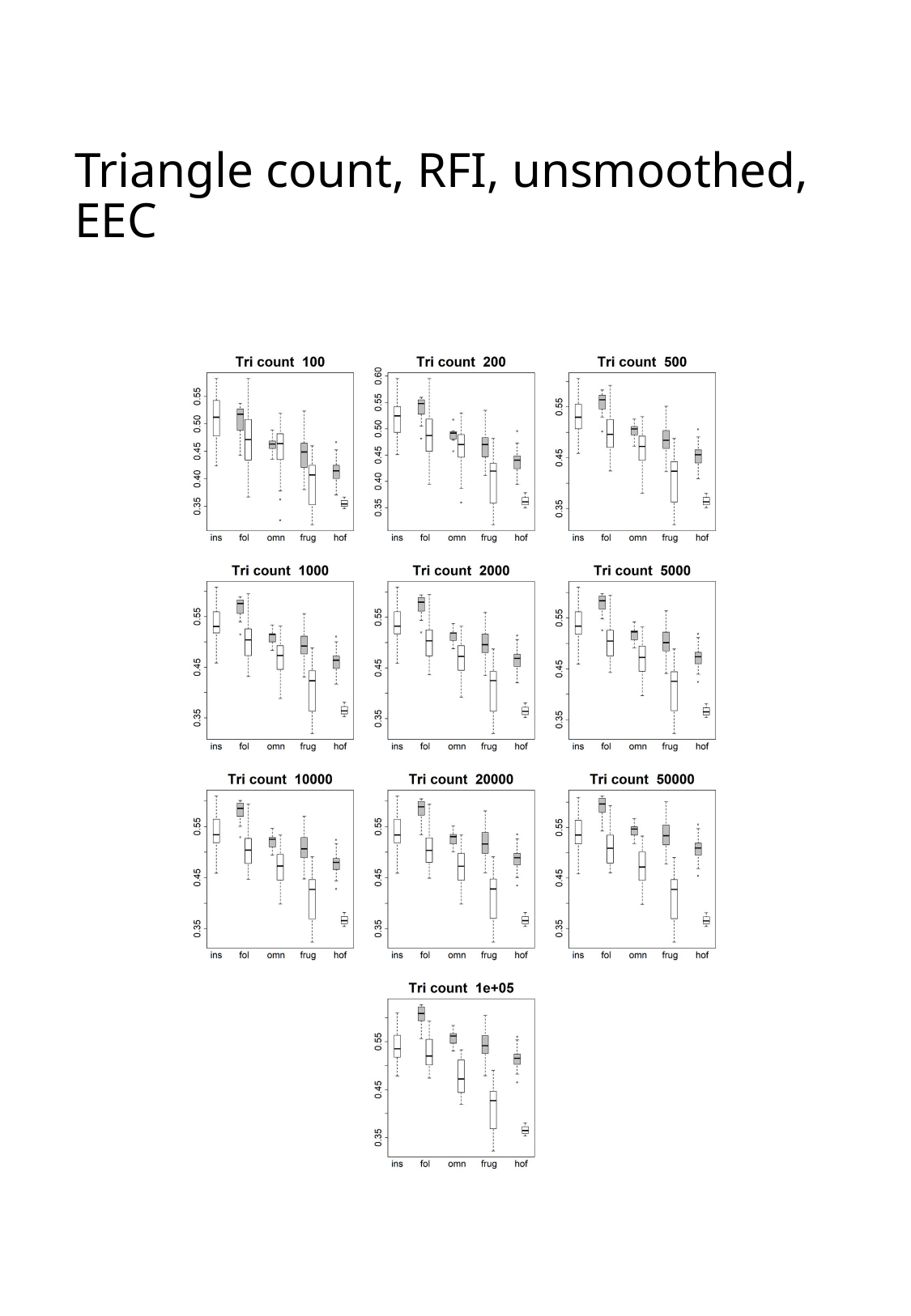

# Triangle count, RFI, unsmoothed, EEC

## Slide 13
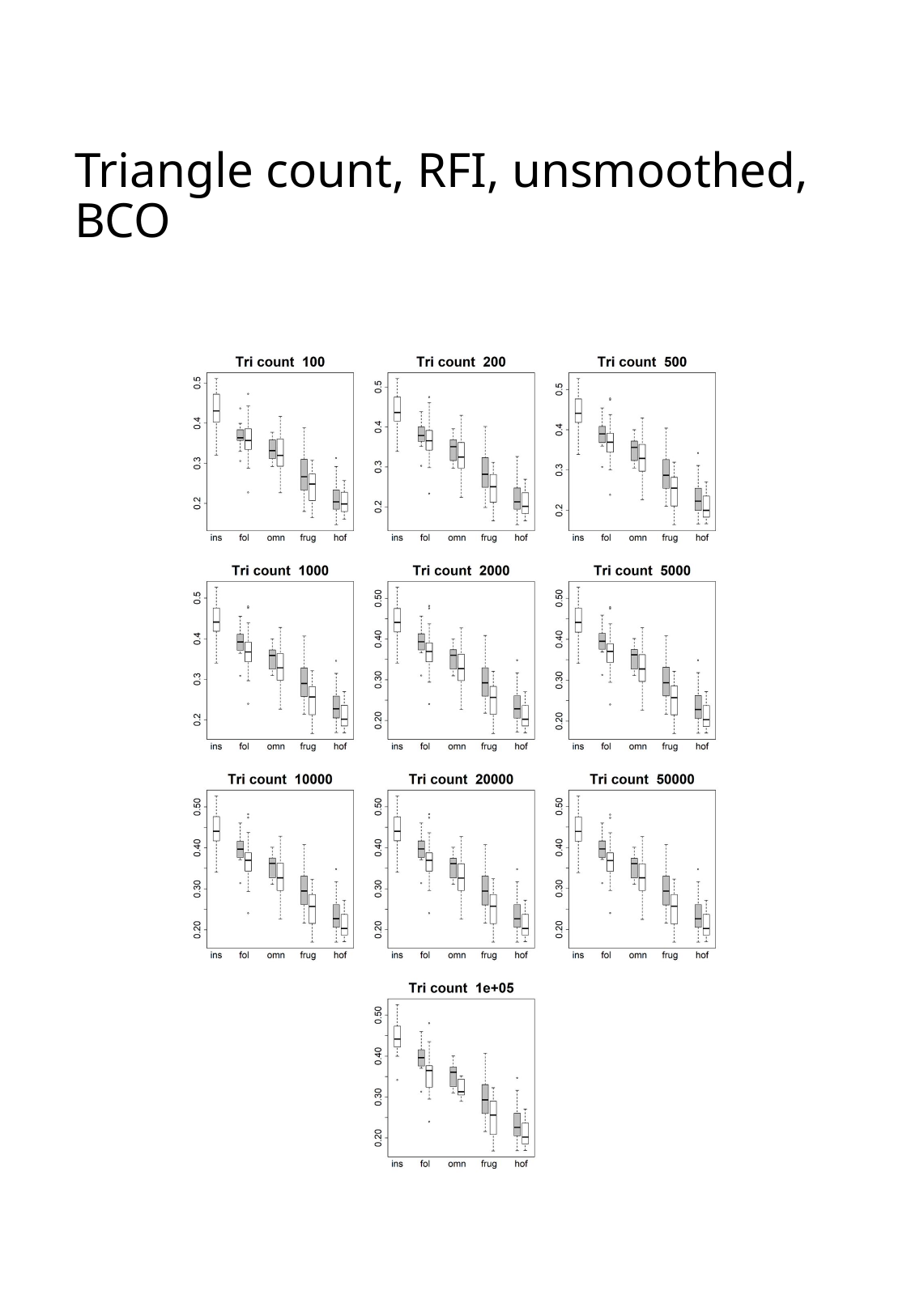

# Triangle count, RFI, unsmoothed, BCO

## Slide 14
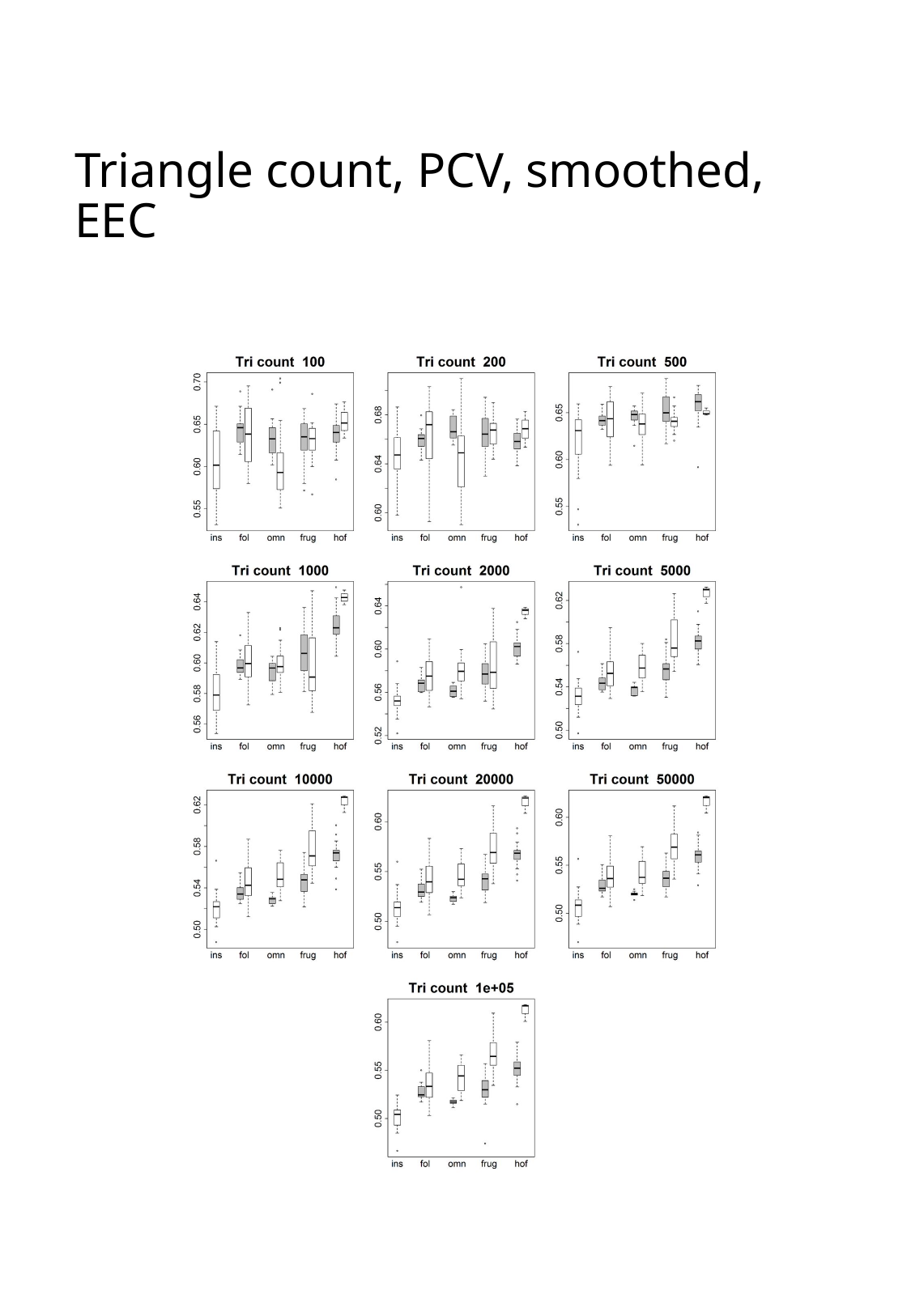

# Triangle count, PCV, smoothed, EEC

## Slide 15
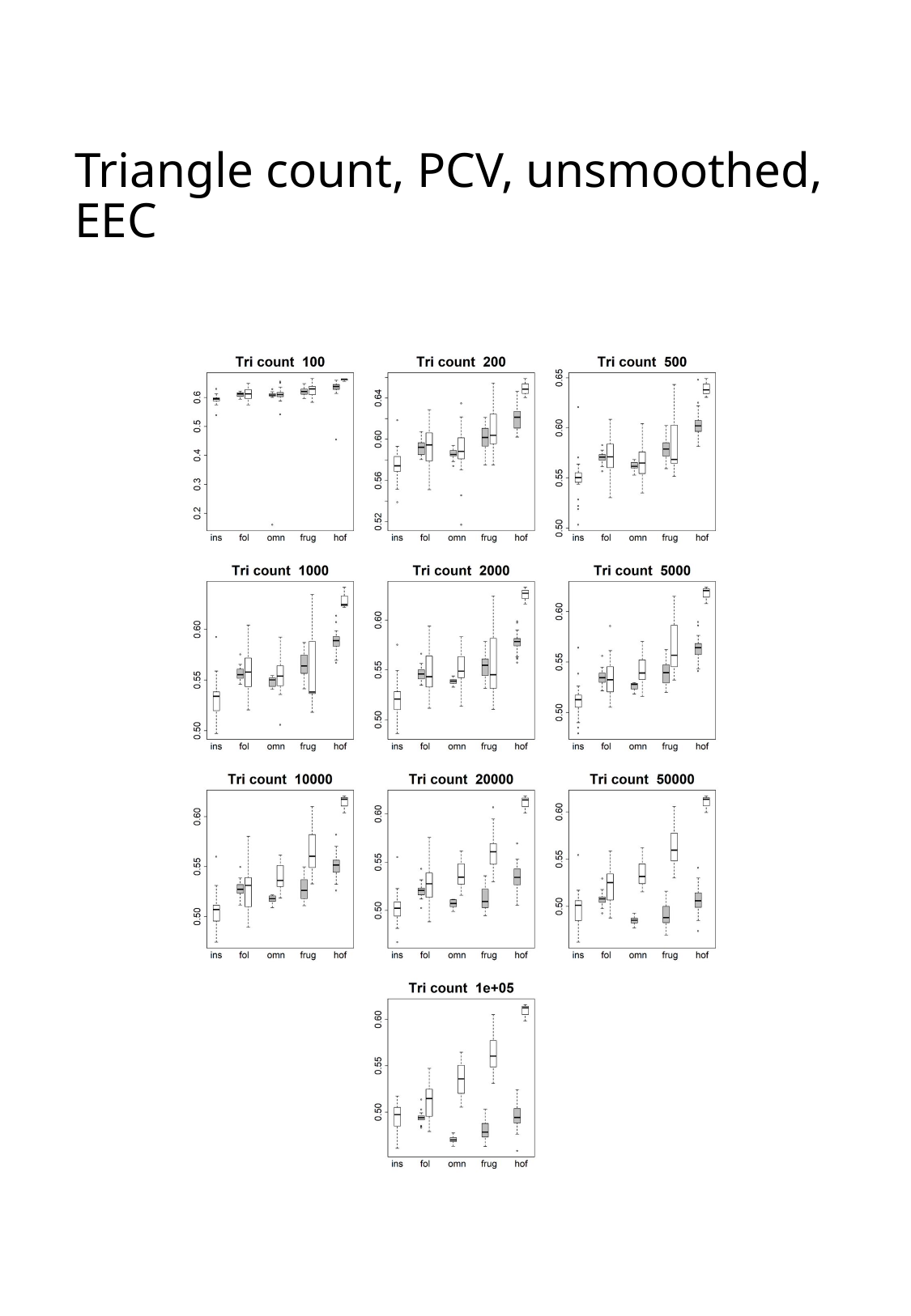

# Triangle count, PCV, unsmoothed, EEC

## Slide 16
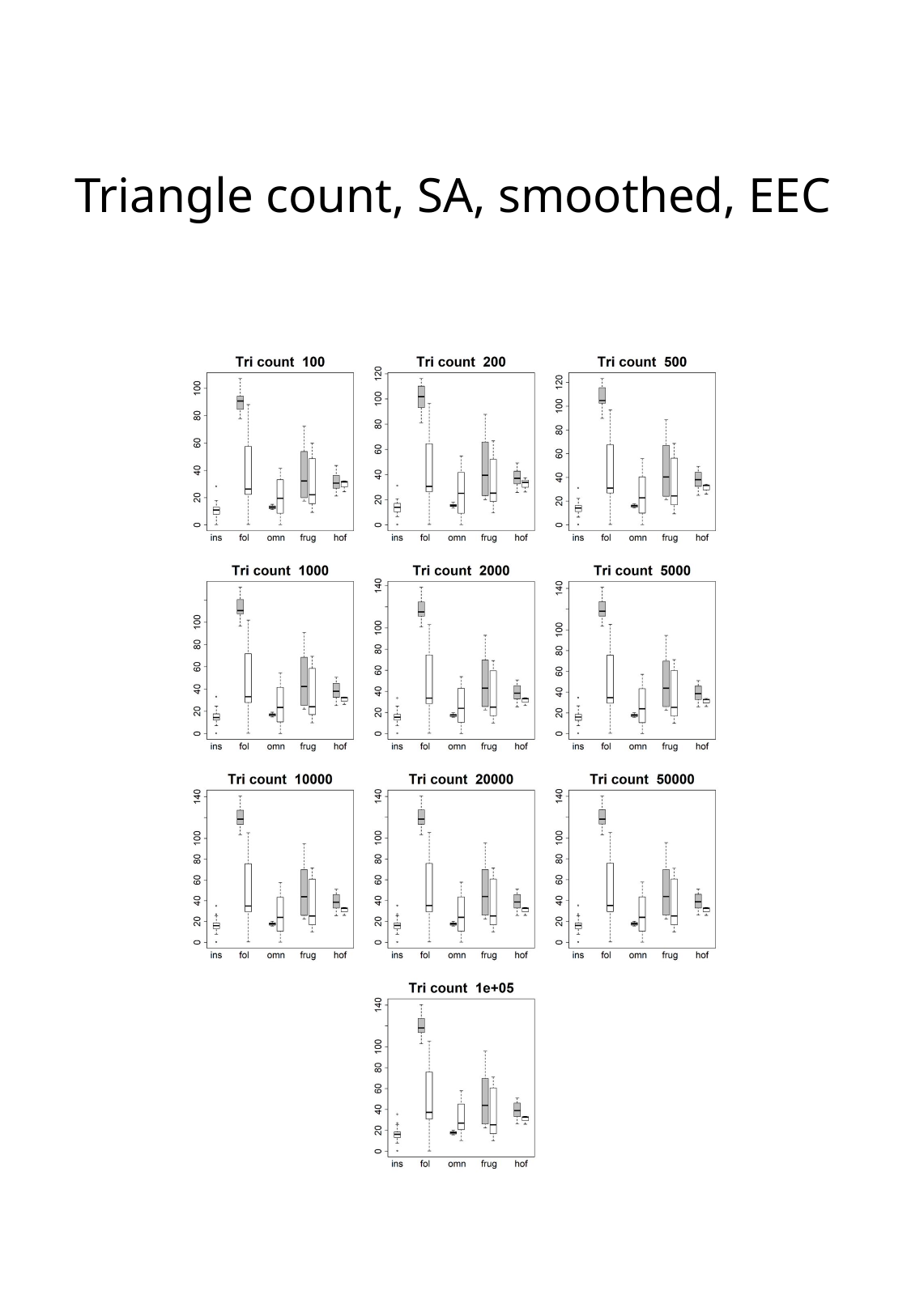

# Triangle count, SA, smoothed, EEC

## Slide 17
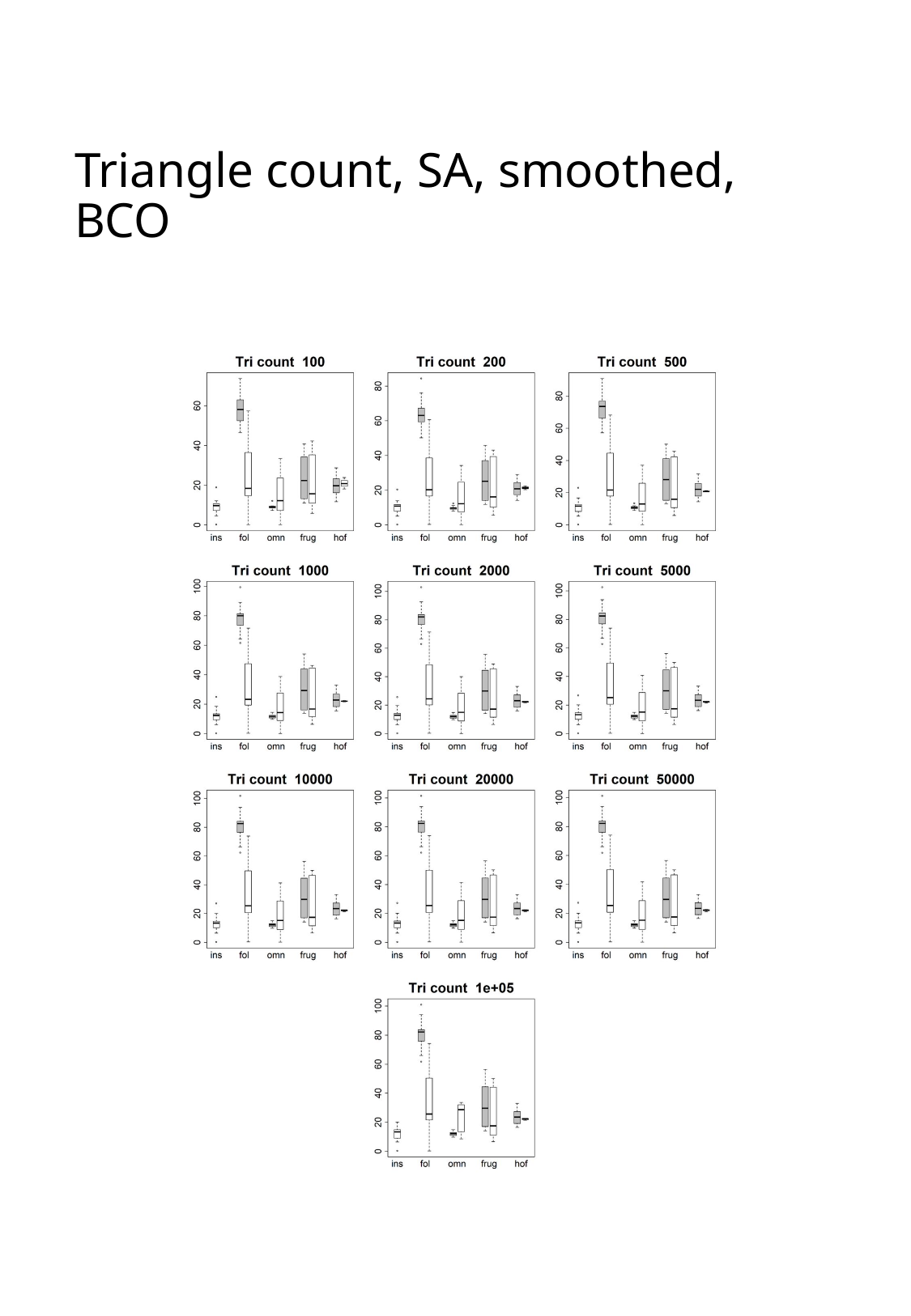

# Triangle count, SA, smoothed, BCO

## Slide 18
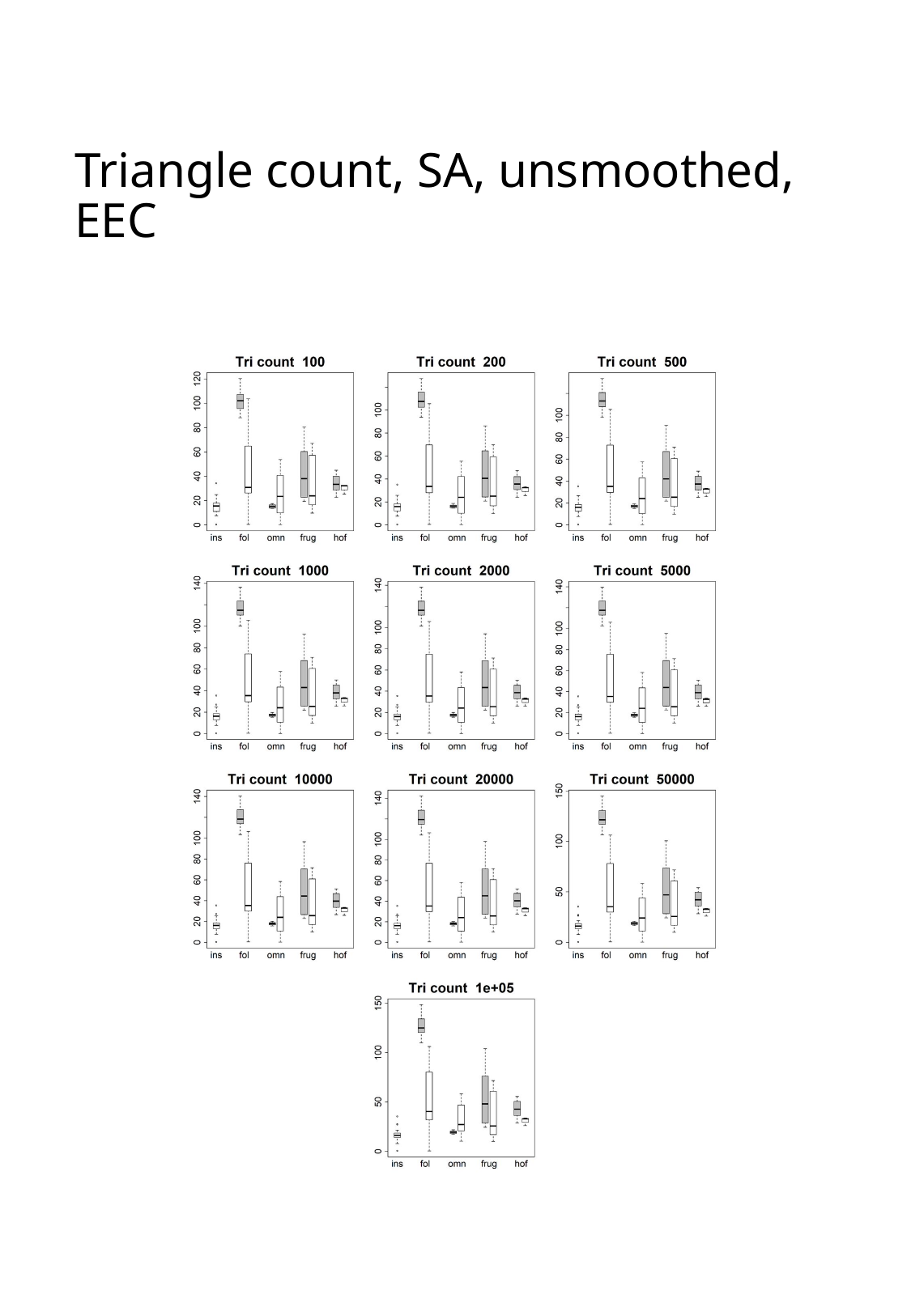

# Triangle count, SA, unsmoothed, EEC

## Slide 19
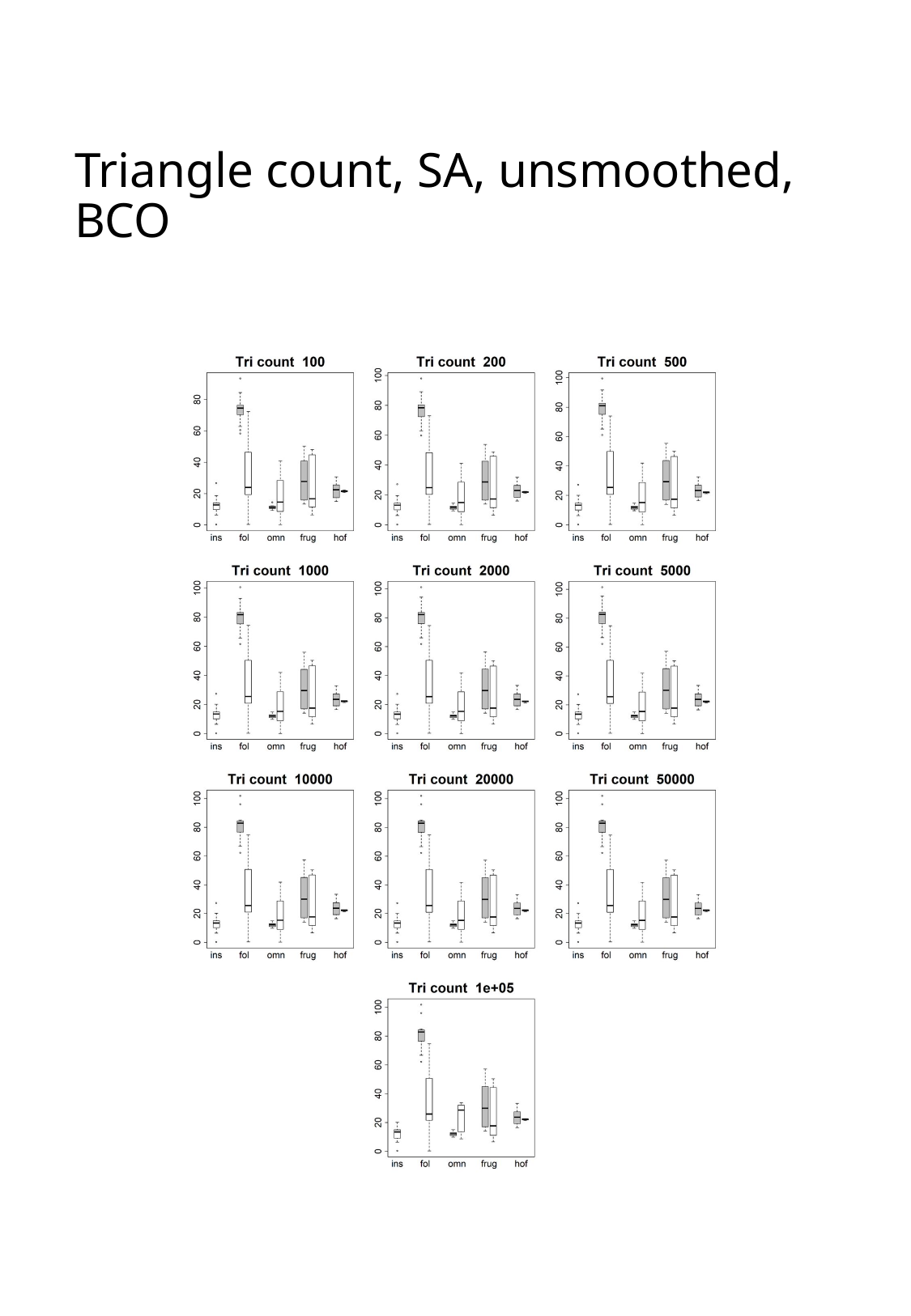

# Triangle count, SA, unsmoothed, BCO

## Slide 20
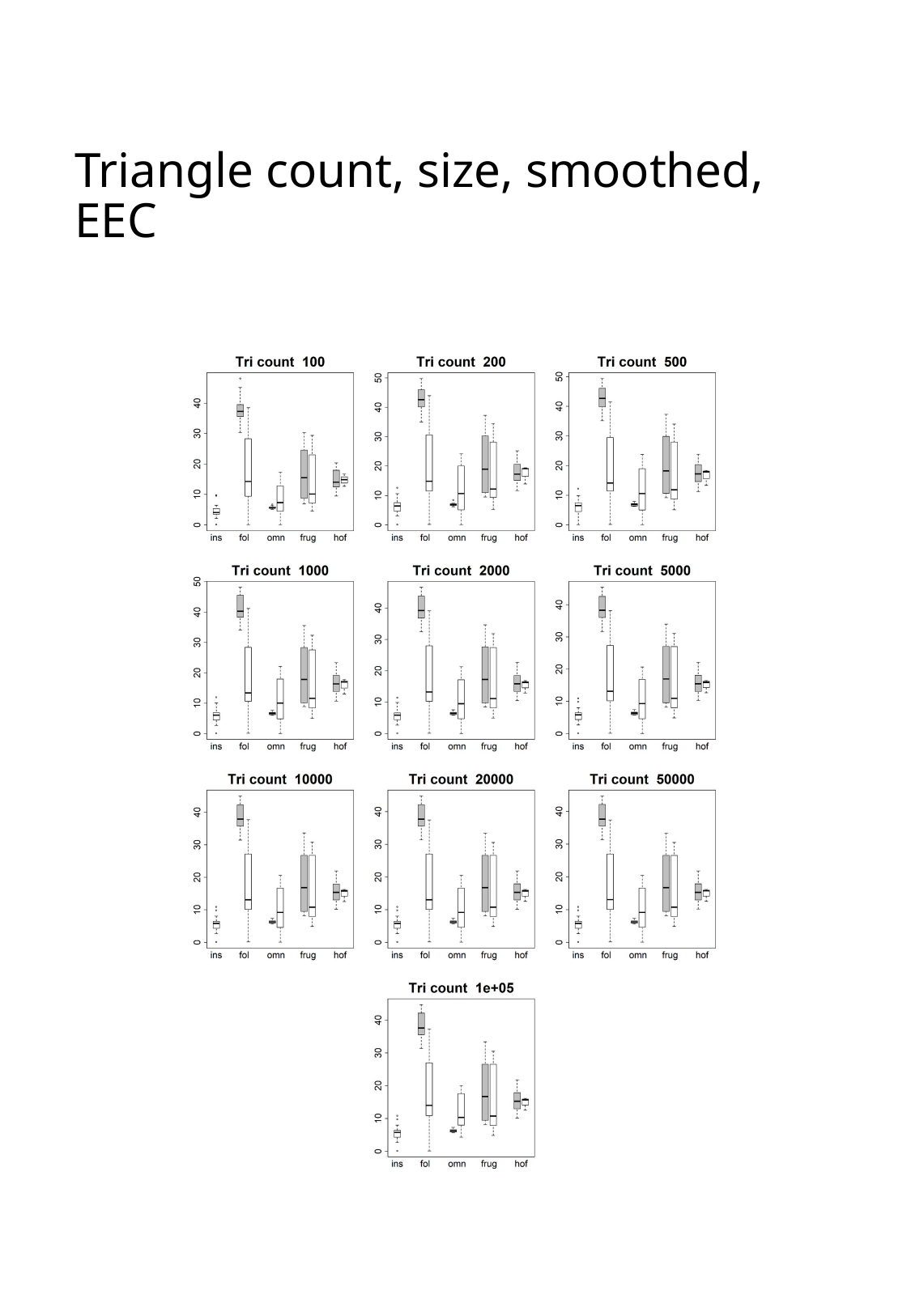

# Triangle count, size, smoothed, EEC

## Slide 21
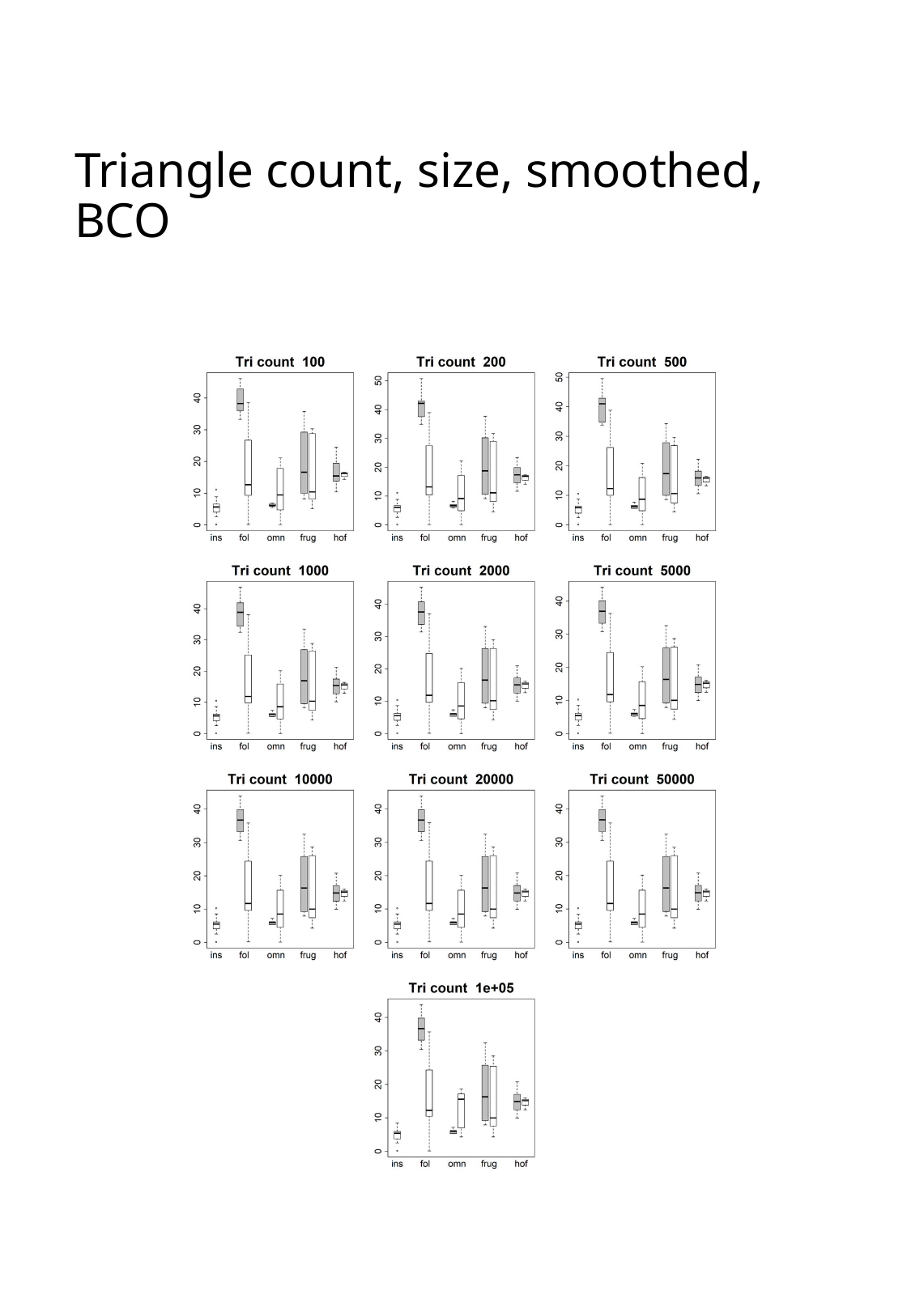

# Triangle count, size, smoothed, BCO

## Slide 22
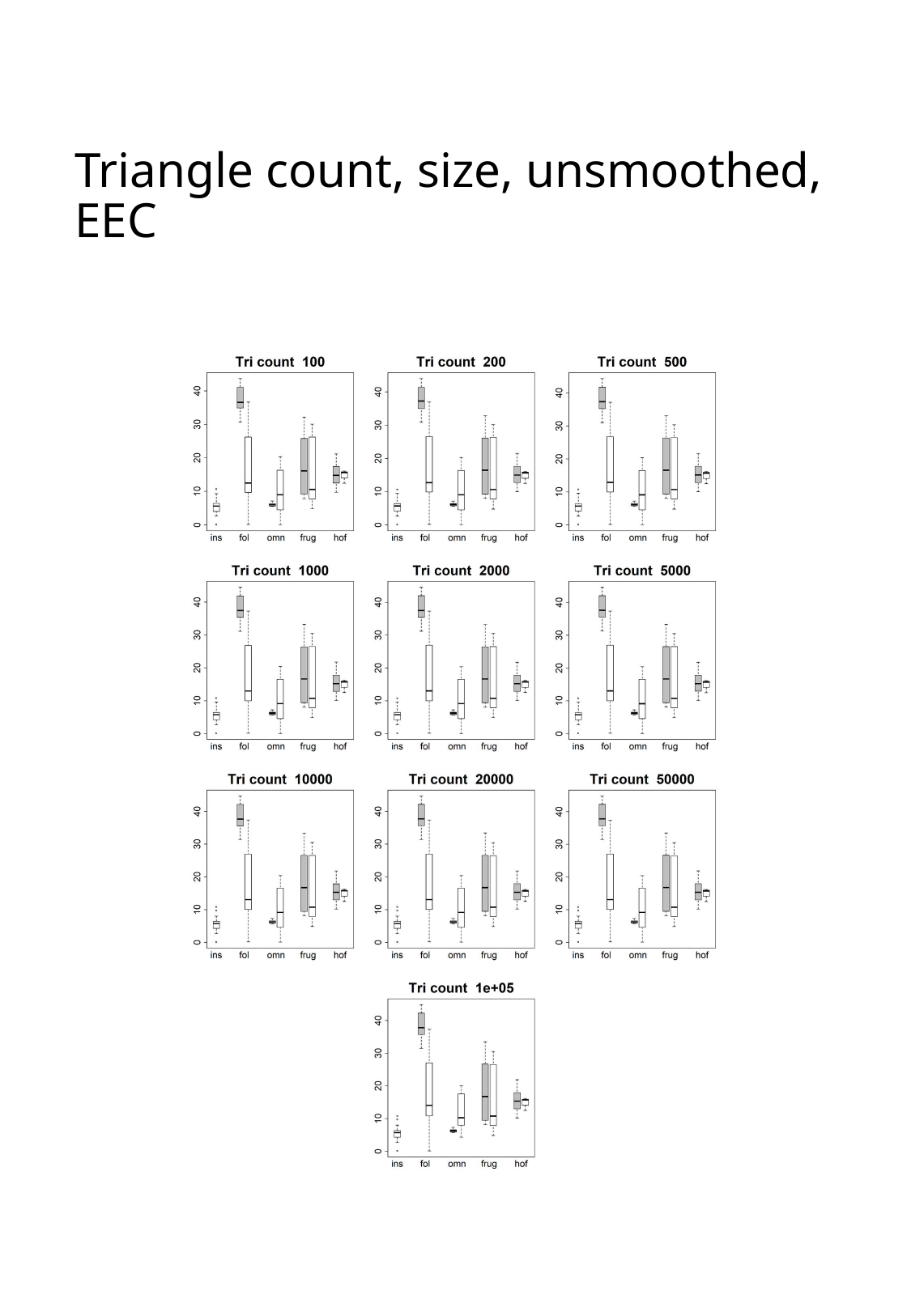

# Triangle count, size, unsmoothed, EEC

## Slide 23
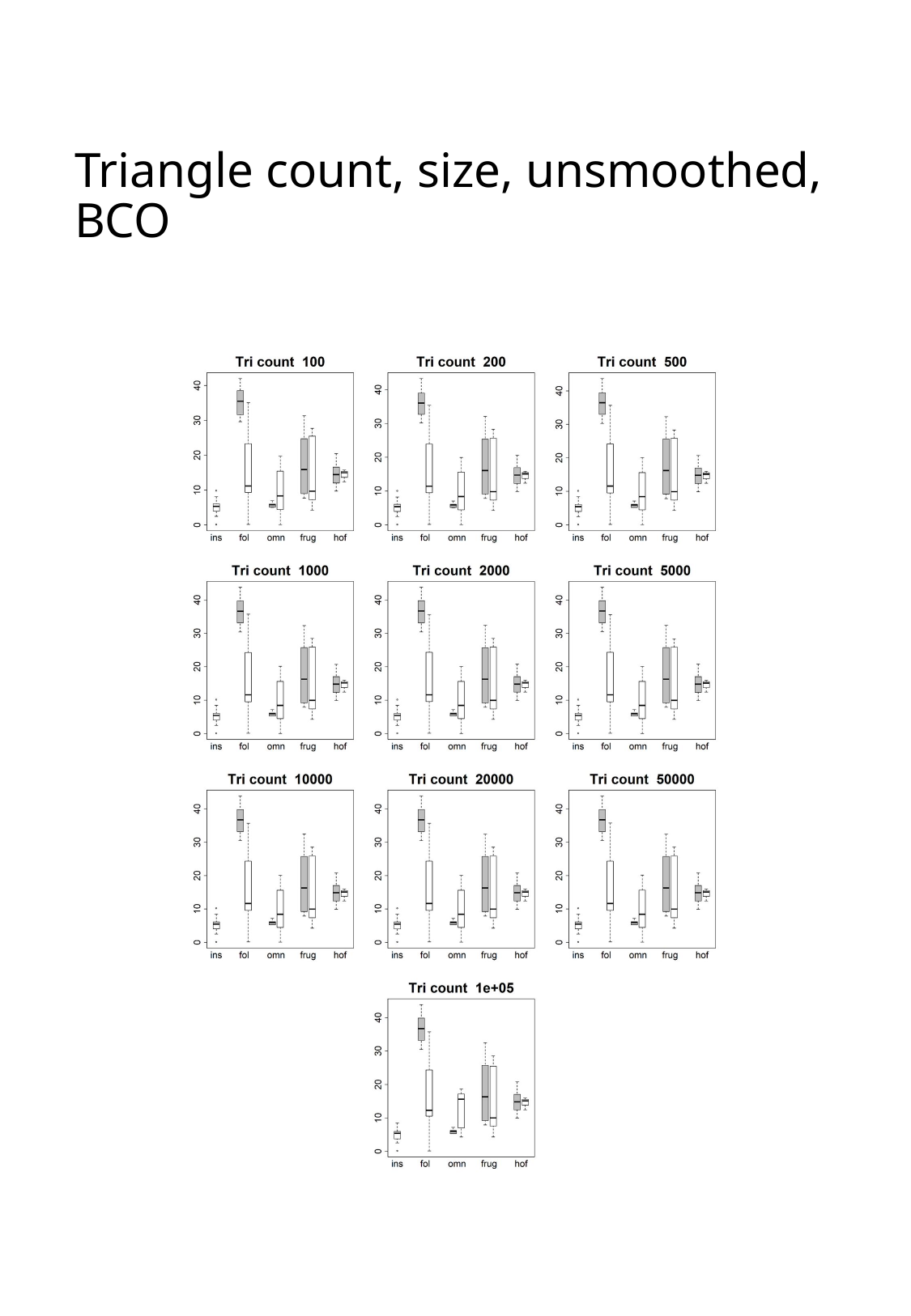

# Triangle count, size, unsmoothed, BCO

## Slide 24
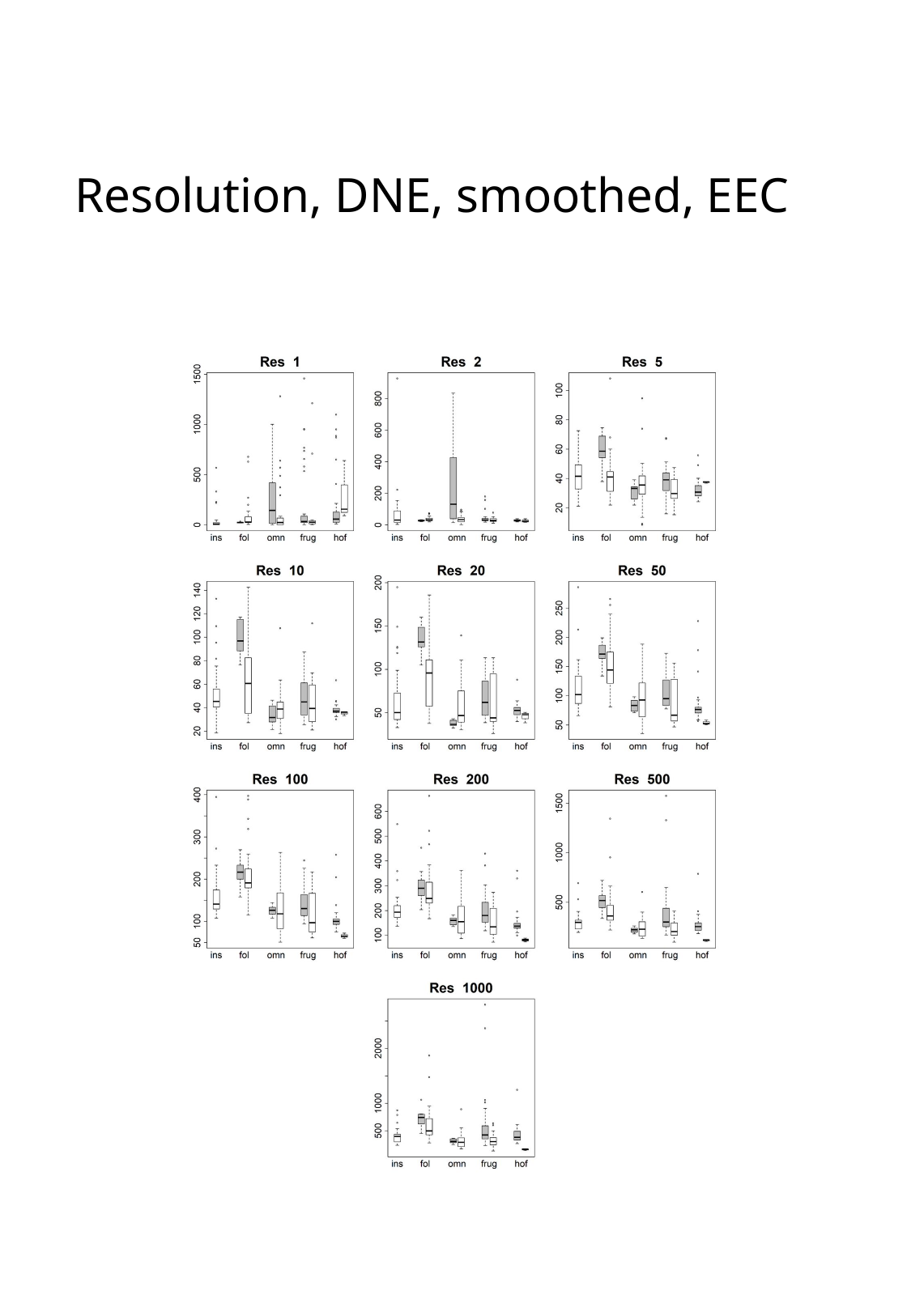

# Resolution, DNE, smoothed, EEC

## Slide 25
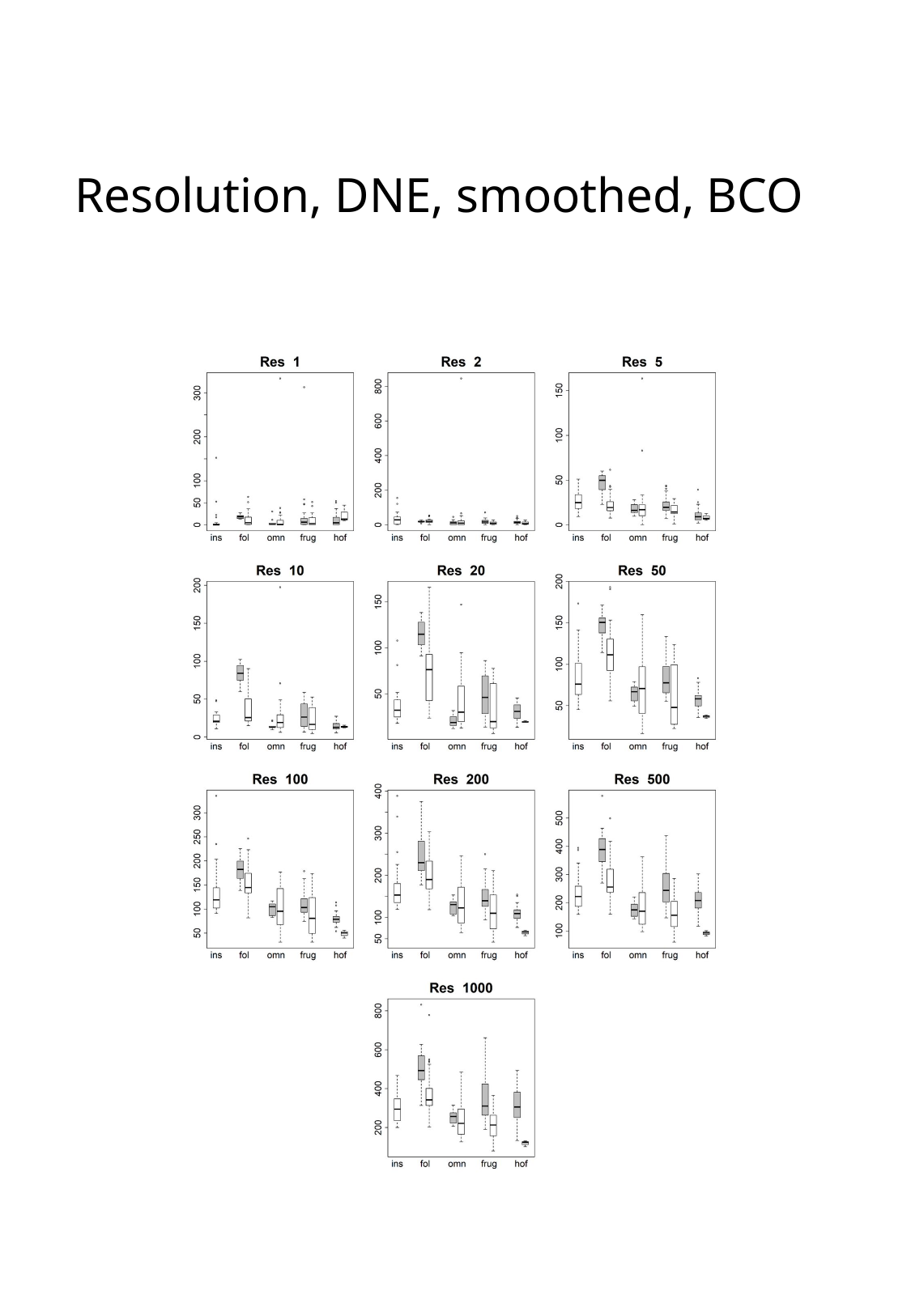

# Resolution, DNE, smoothed, BCO

## Slide 26
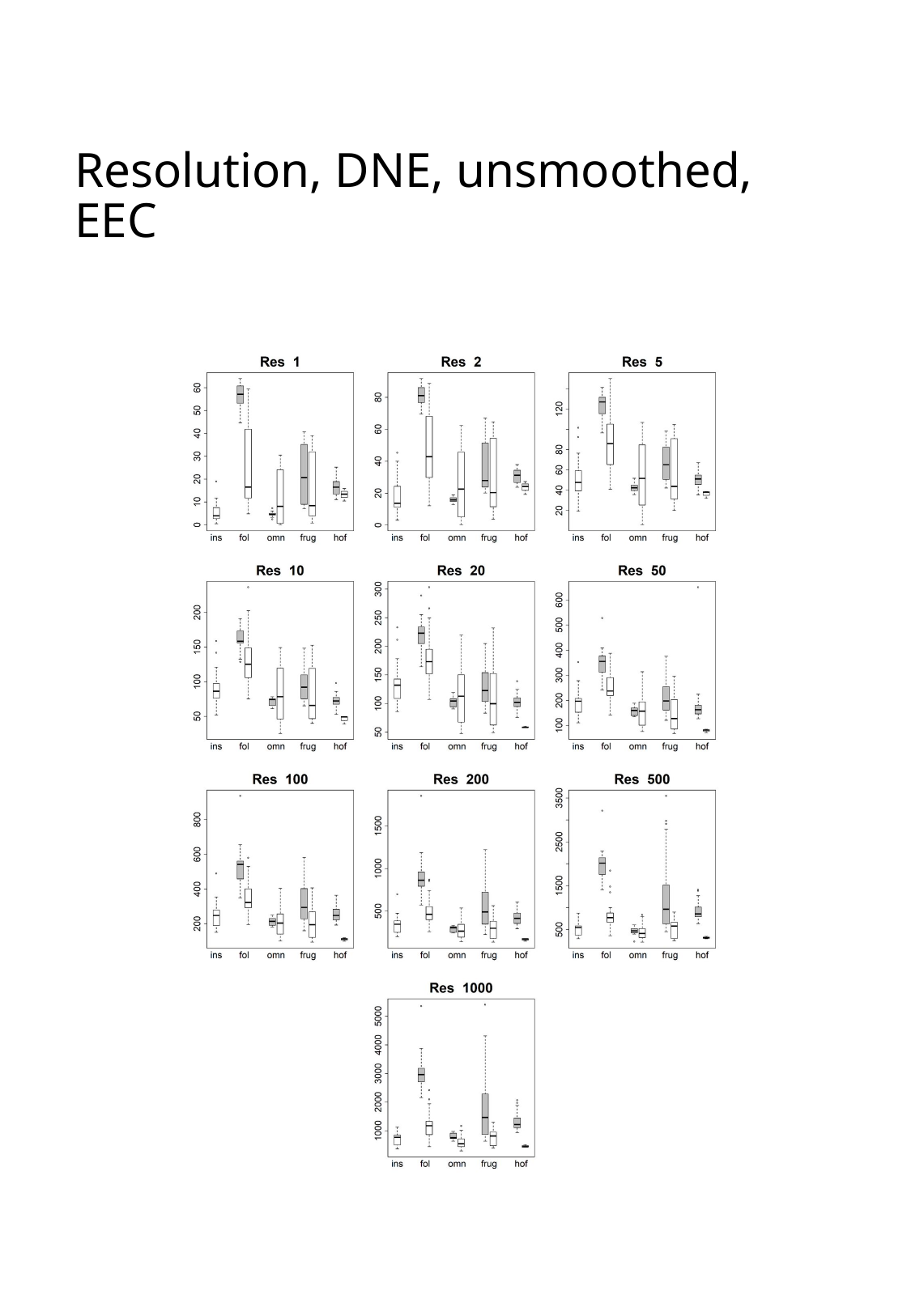

# Resolution, DNE, unsmoothed, EEC

## Slide 27
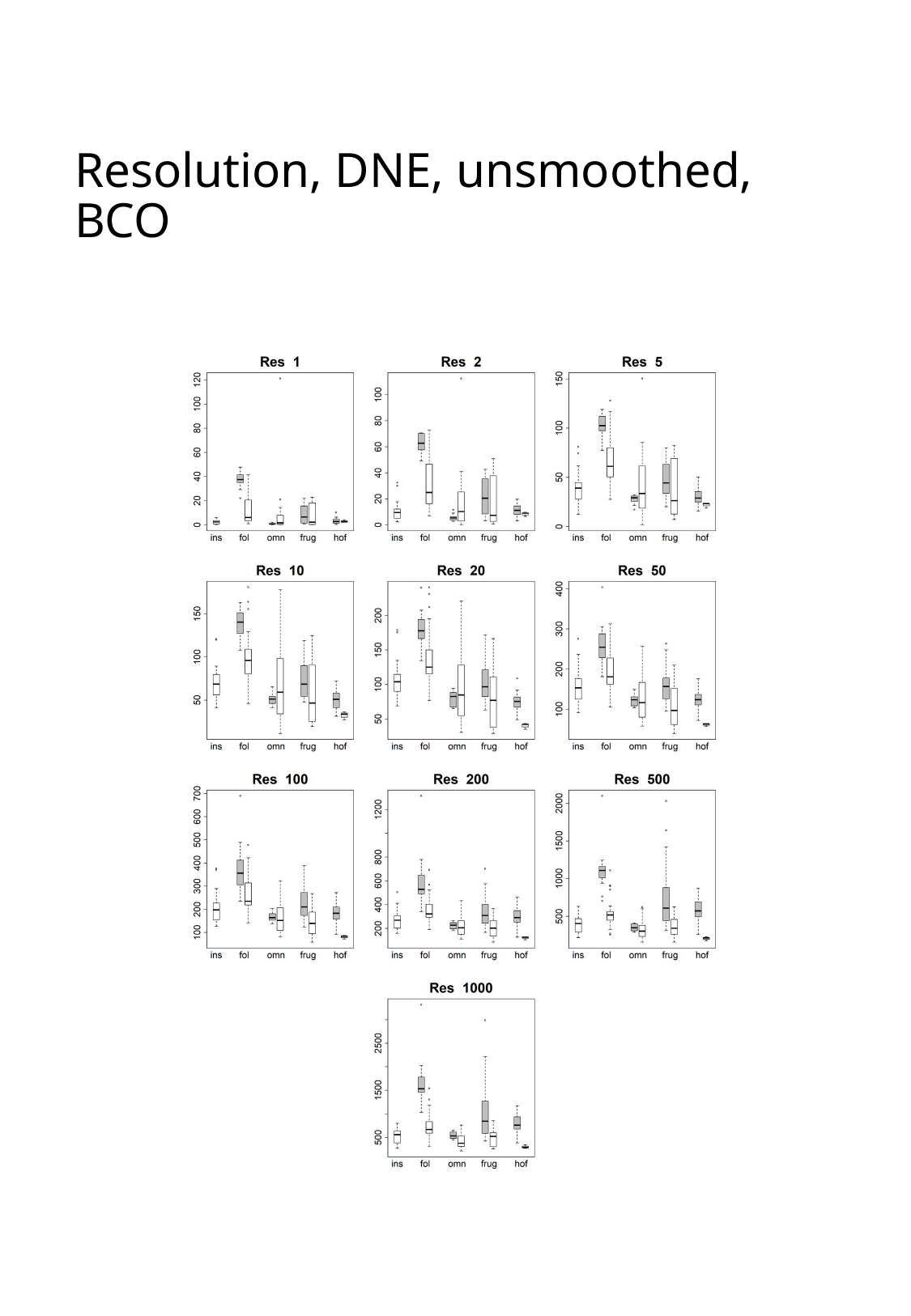

# Resolution, DNE, unsmoothed, BCO

## Slide 28
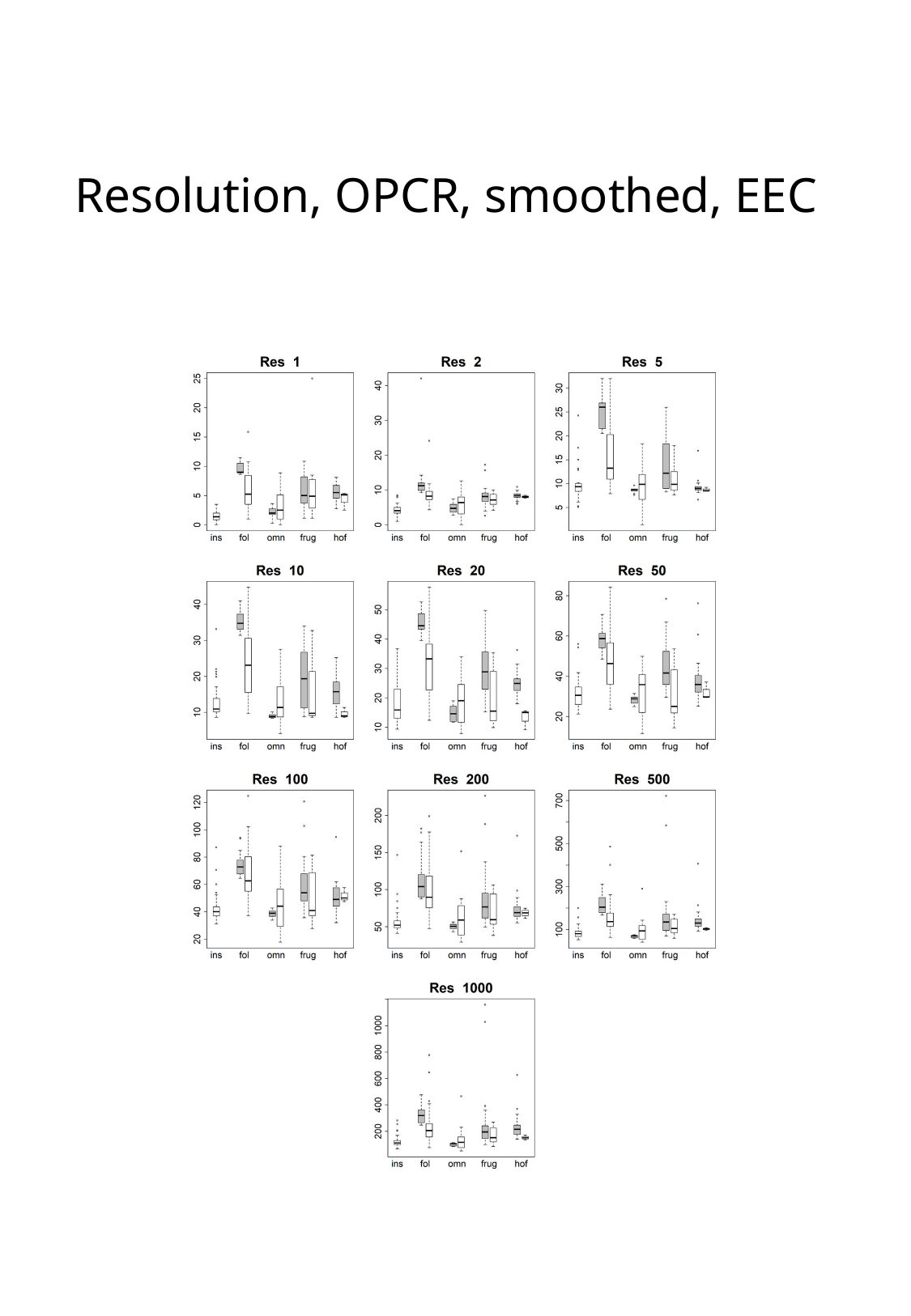

# Resolution, OPCR, smoothed, EEC

## Slide 29
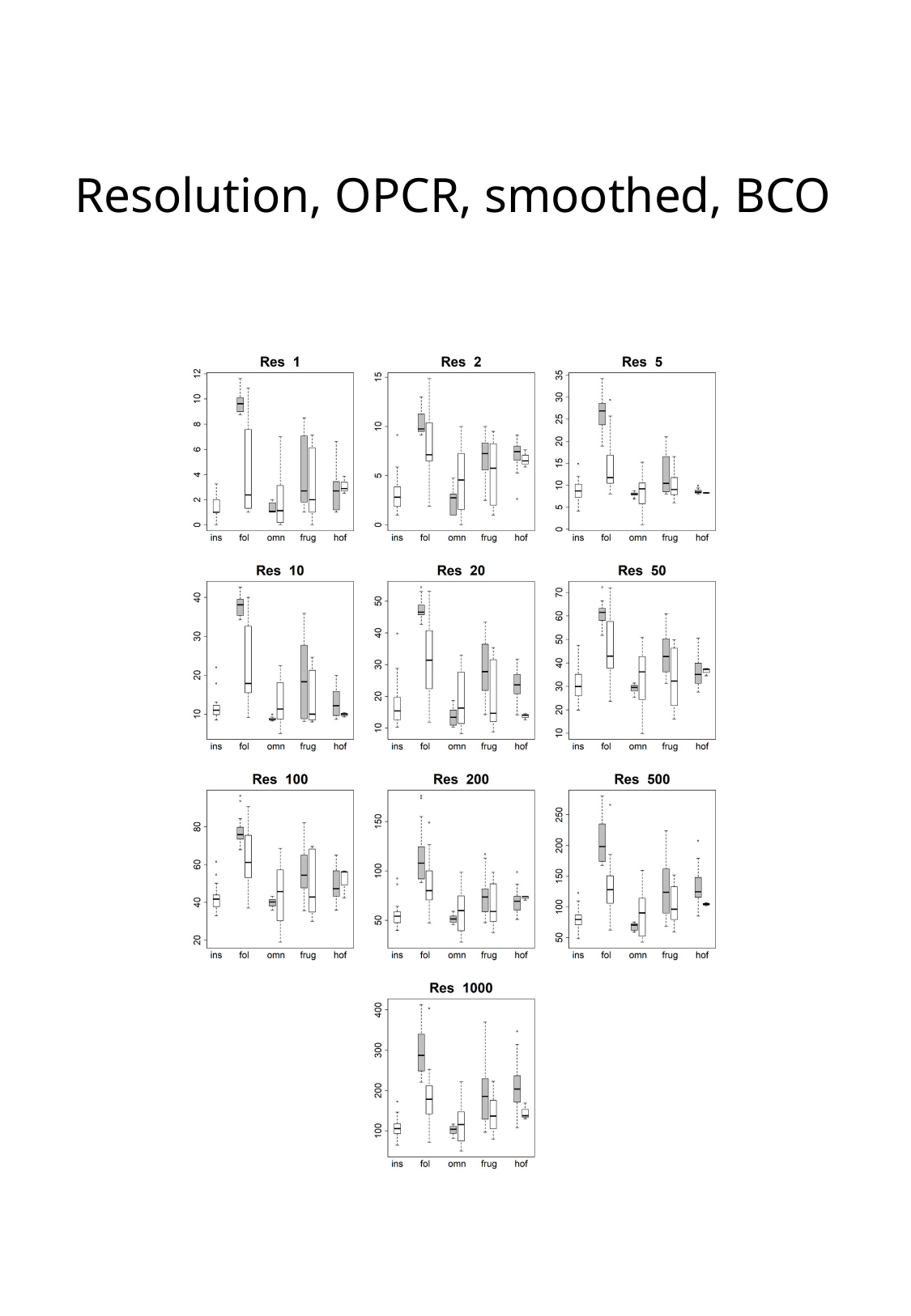

# Resolution, OPCR, smoothed, BCO

## Slide 30
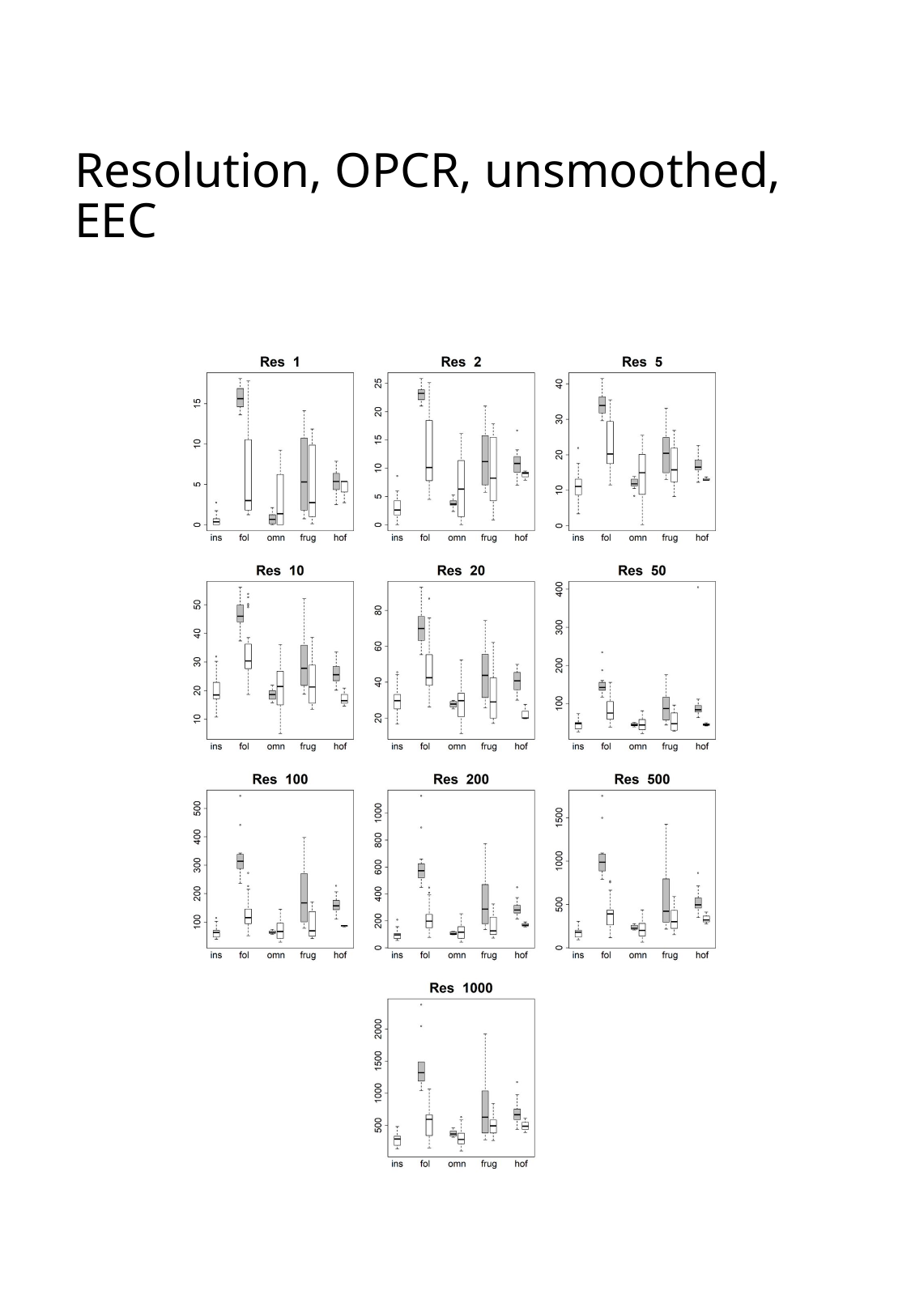

# Resolution, OPCR, unsmoothed, EEC

## Slide 31
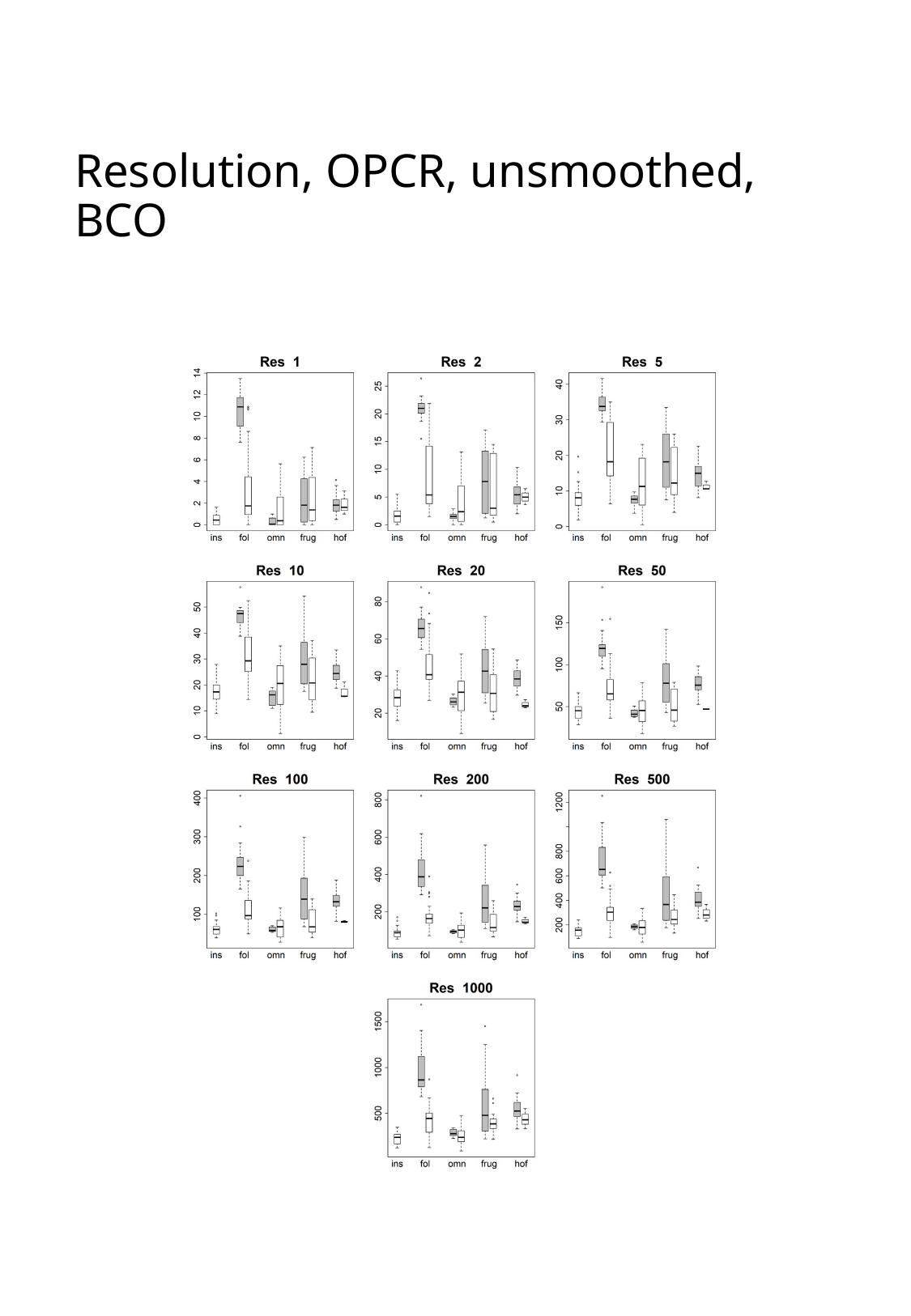

# Resolution, OPCR, unsmoothed, BCO

## Slide 32
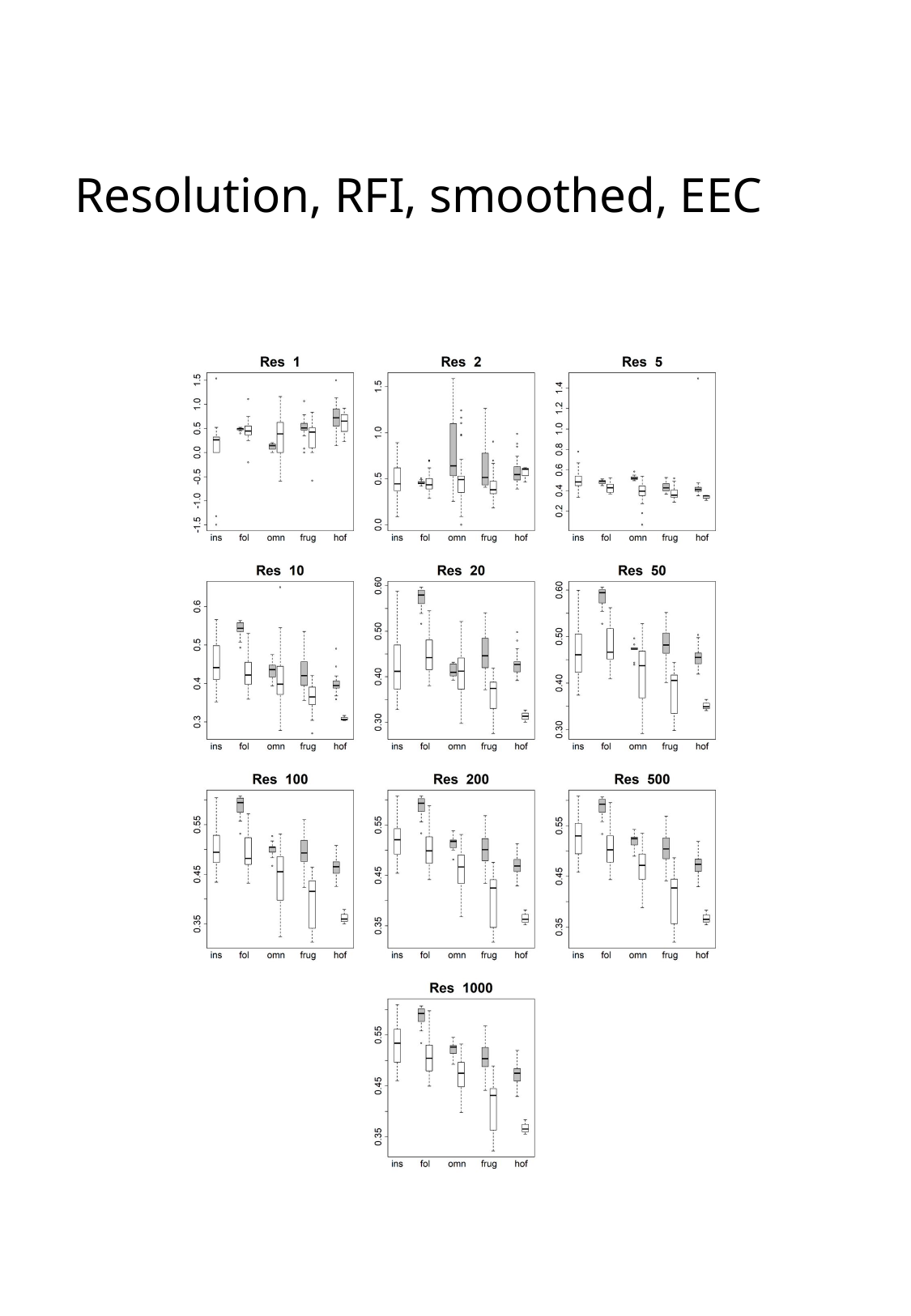

# Resolution, RFI, smoothed, EEC

## Slide 33
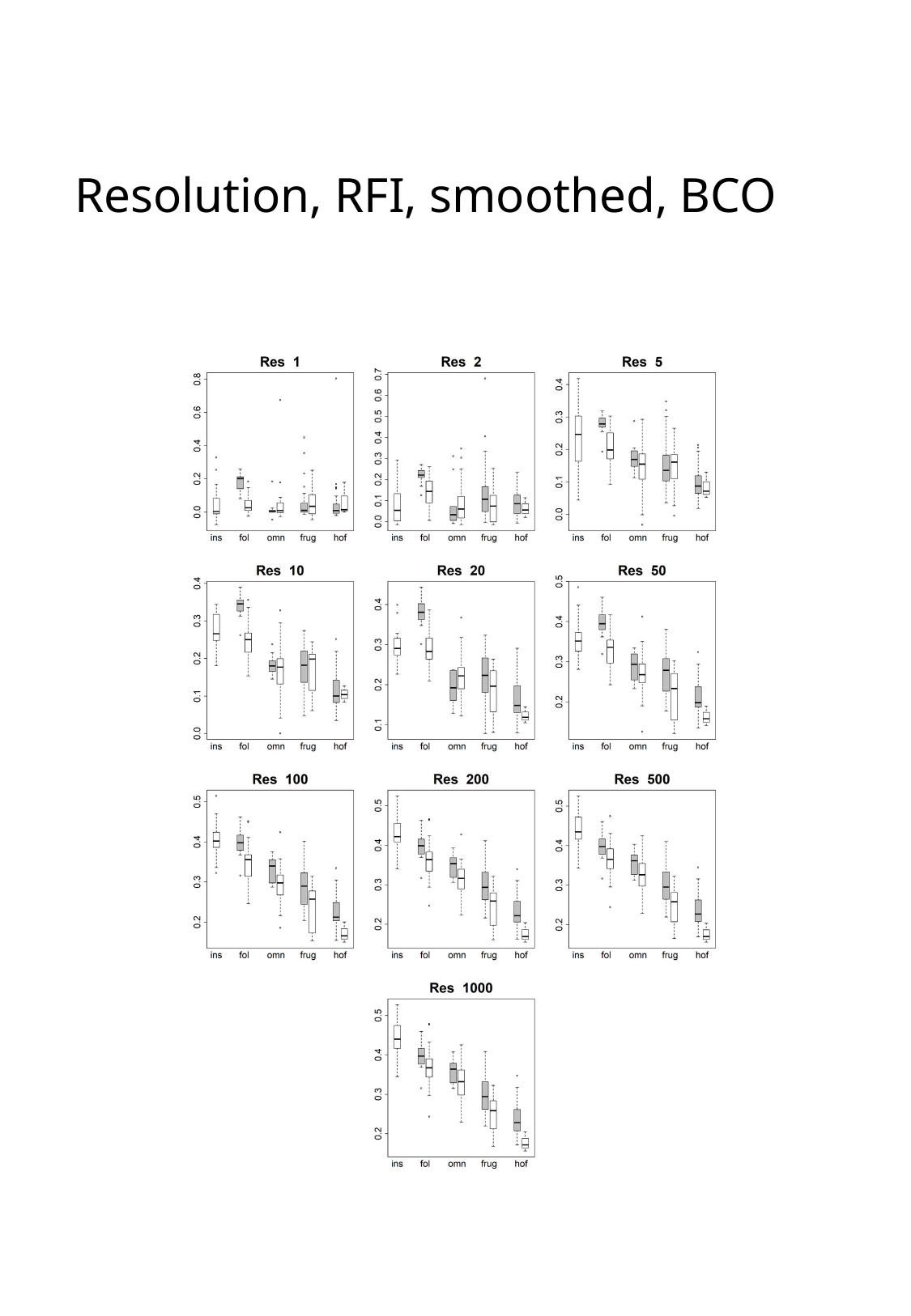

# Resolution, RFI, smoothed, BCO

## Slide 34
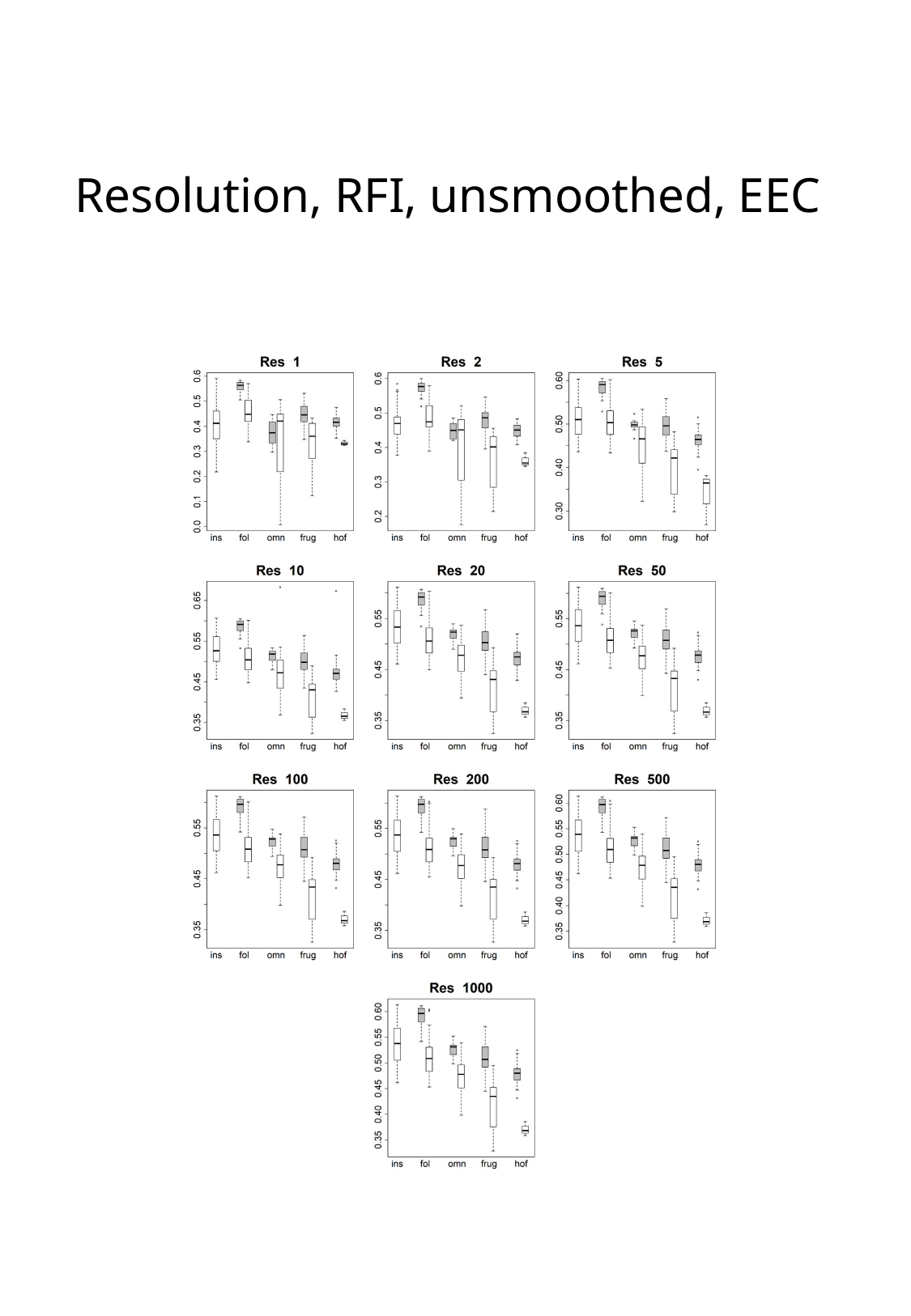

# Resolution, RFI, unsmoothed, EEC

## Slide 35
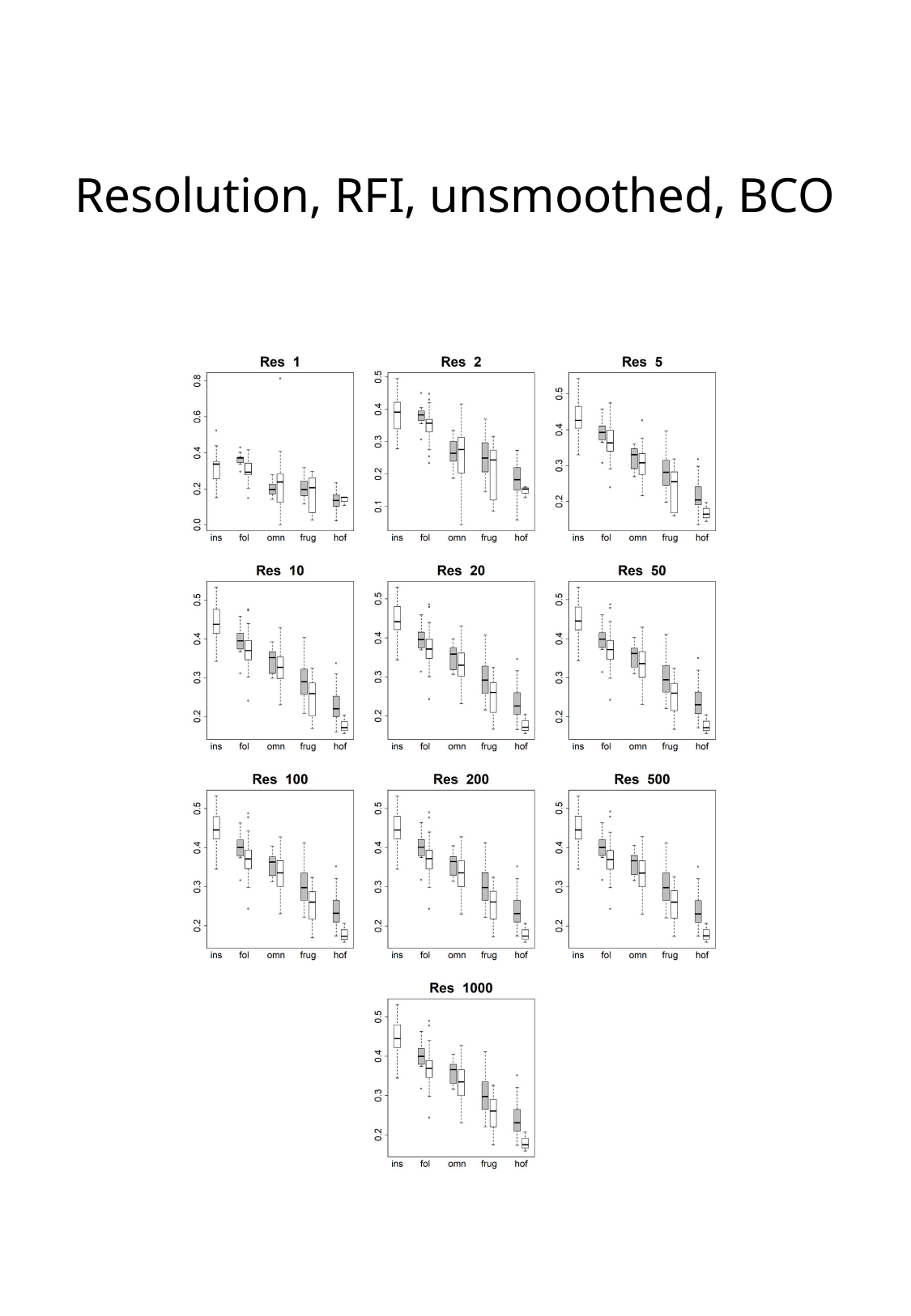

# Resolution, RFI, unsmoothed, BCO

## Slide 36
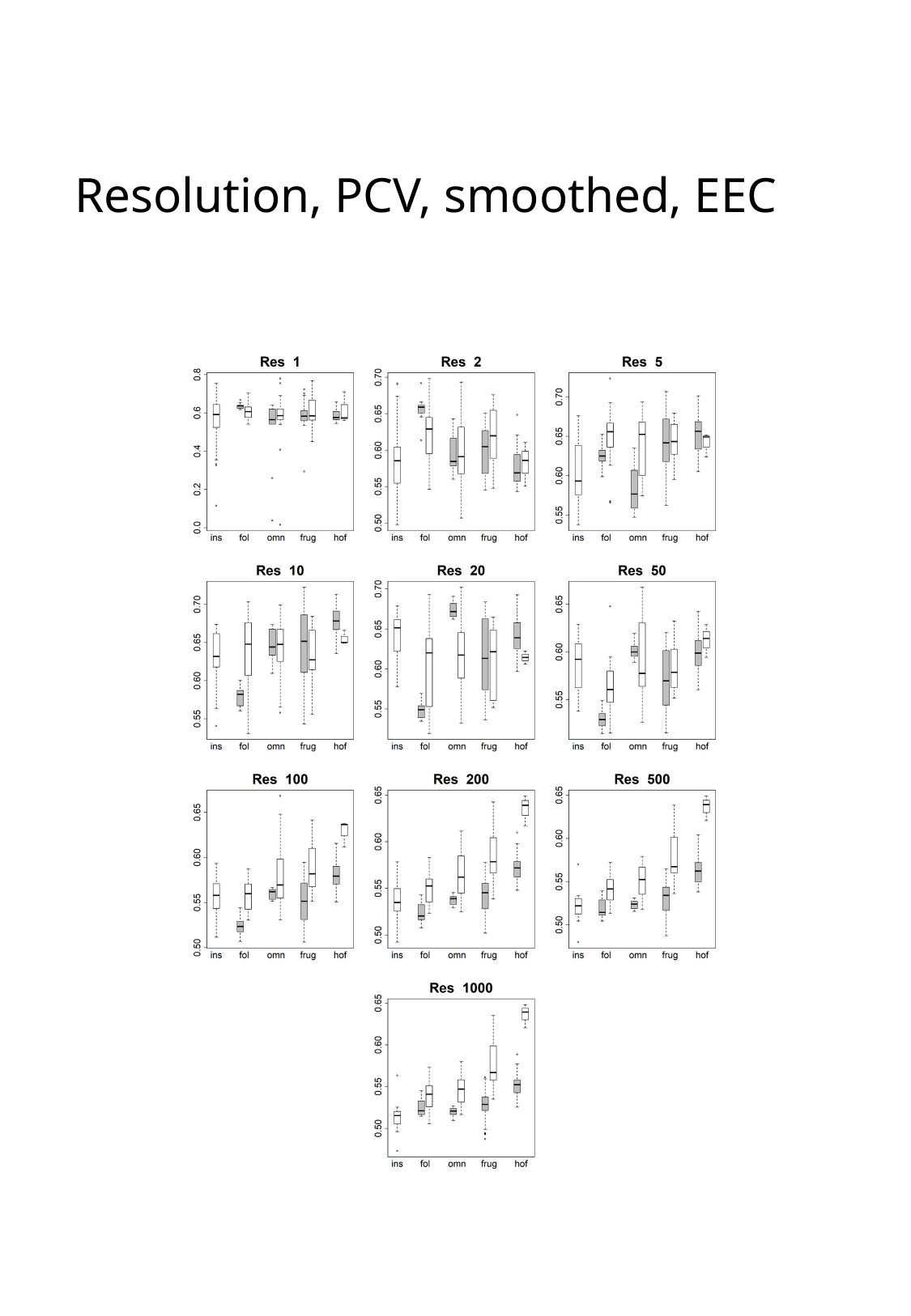

# Resolution, PCV, smoothed, EEC

## Slide 37
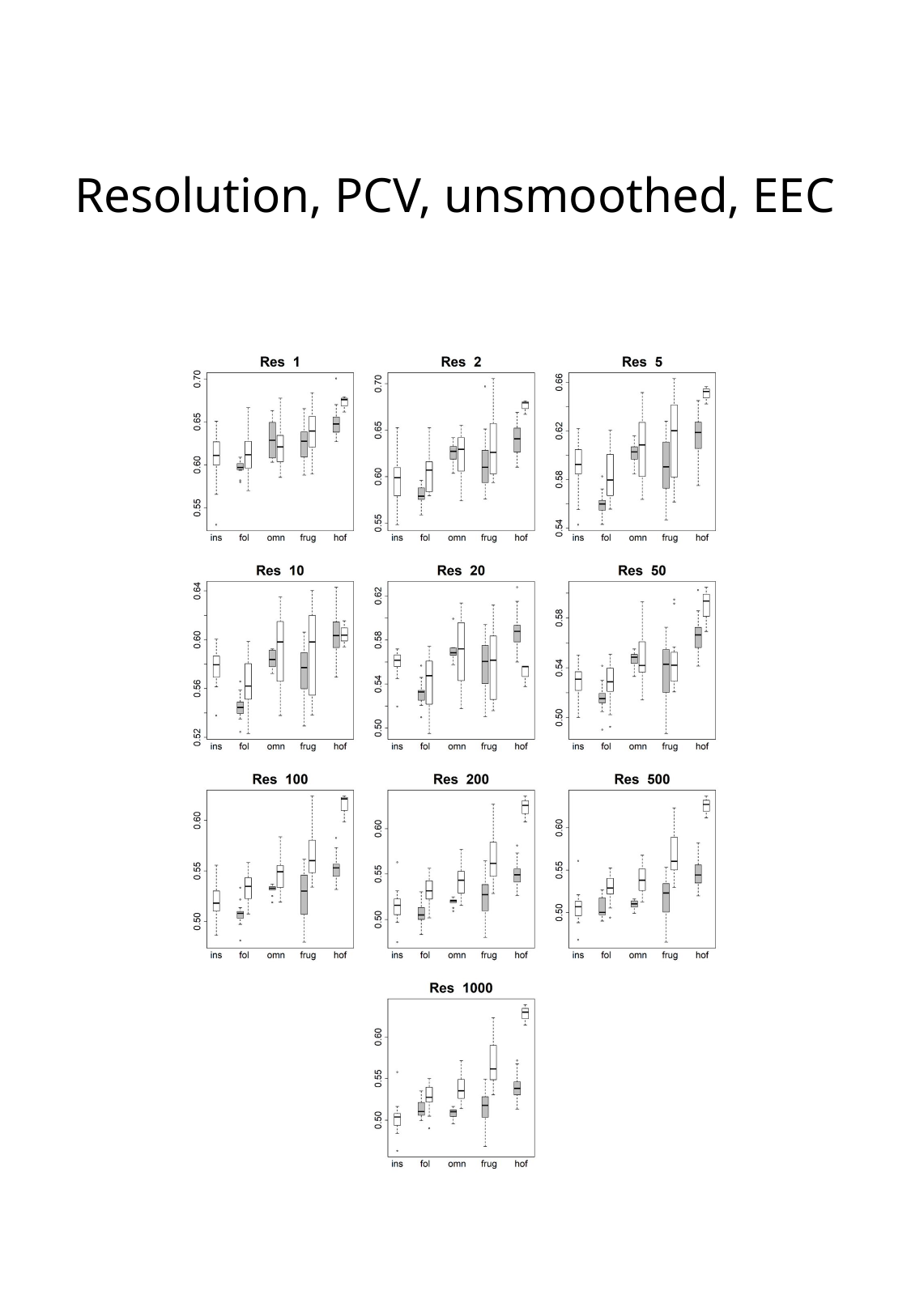

# Resolution, PCV, unsmoothed, EEC

## Slide 38
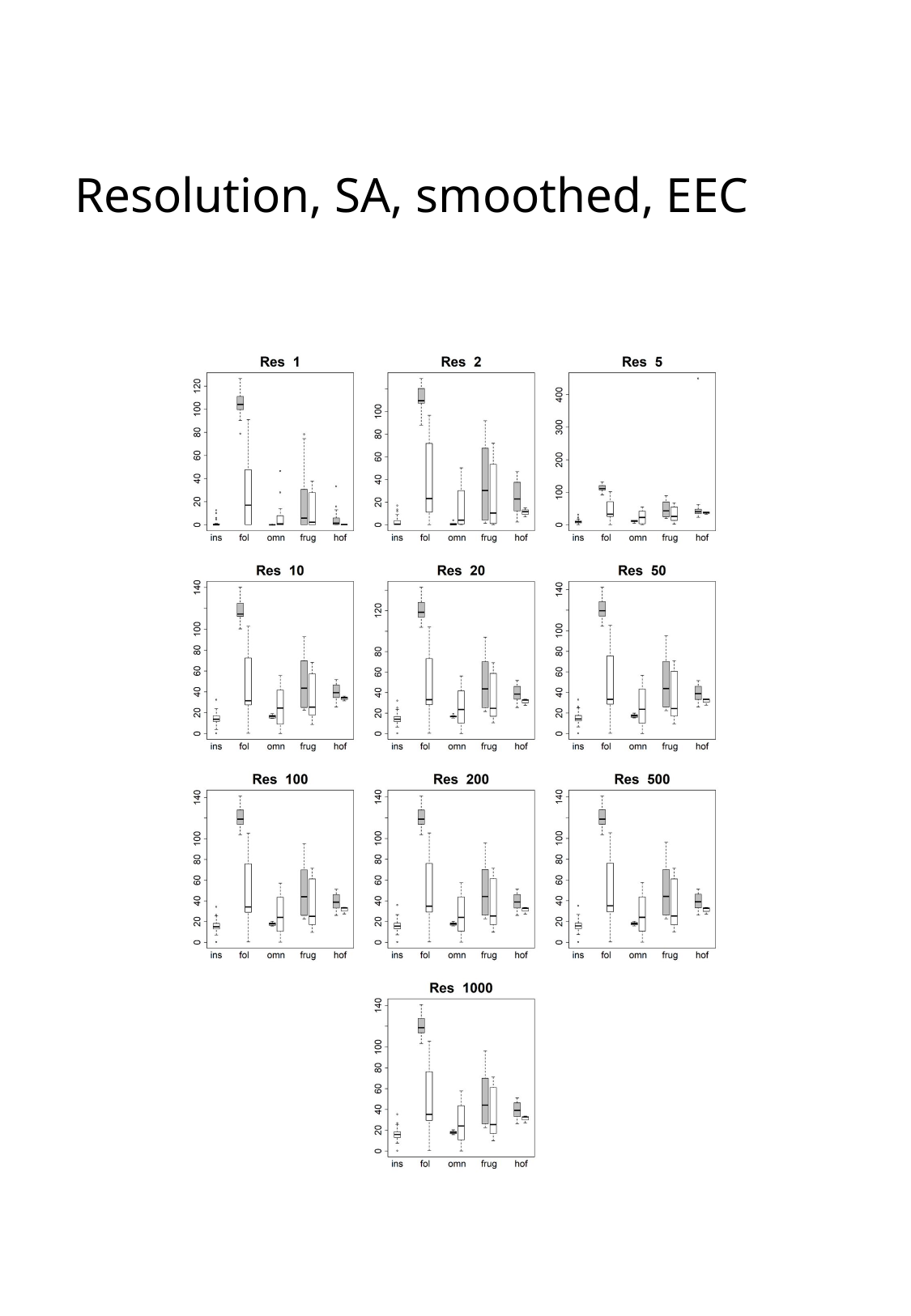

# Resolution, SA, smoothed, EEC

## Slide 39
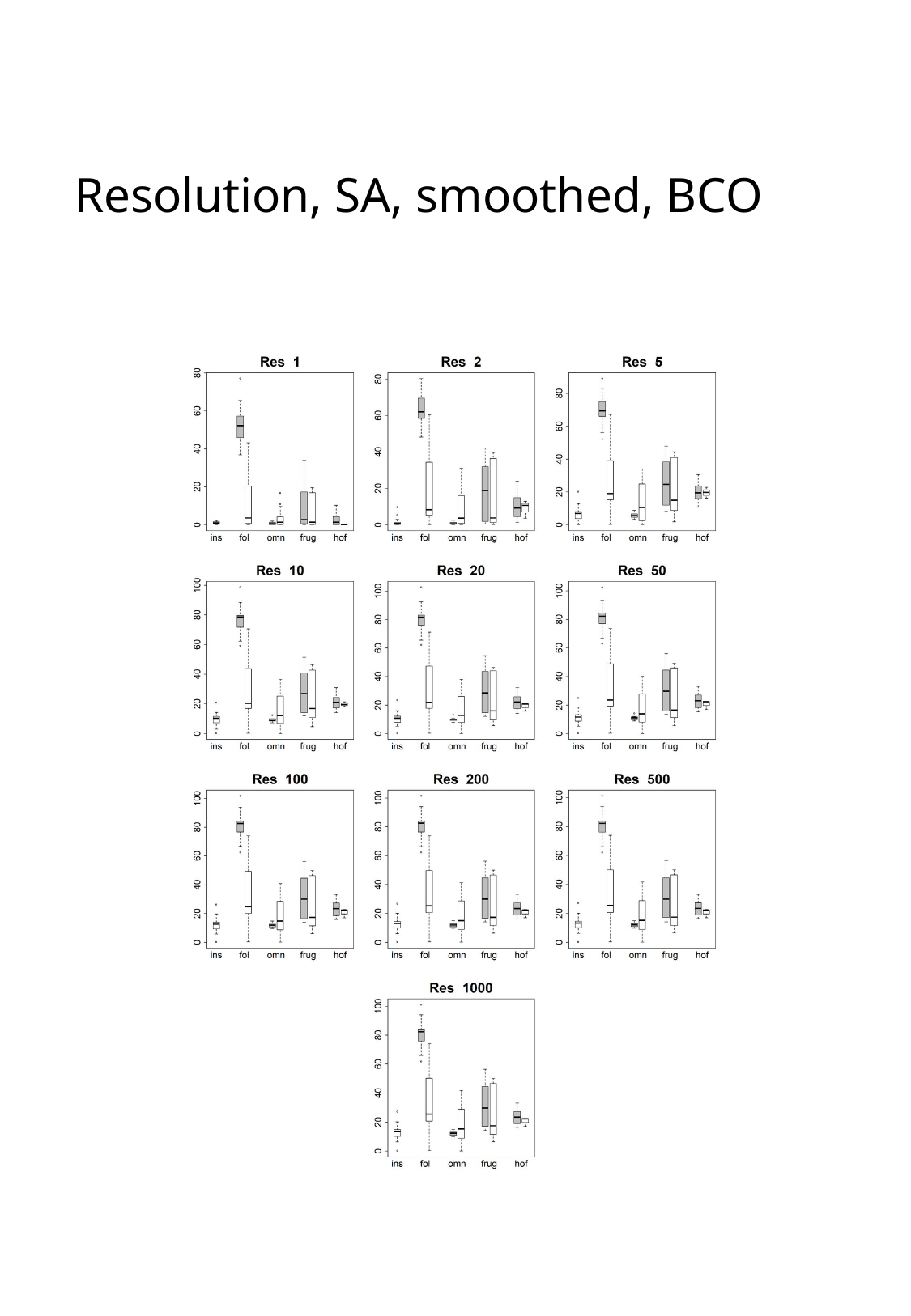

# Resolution, SA, smoothed, BCO

## Slide 40
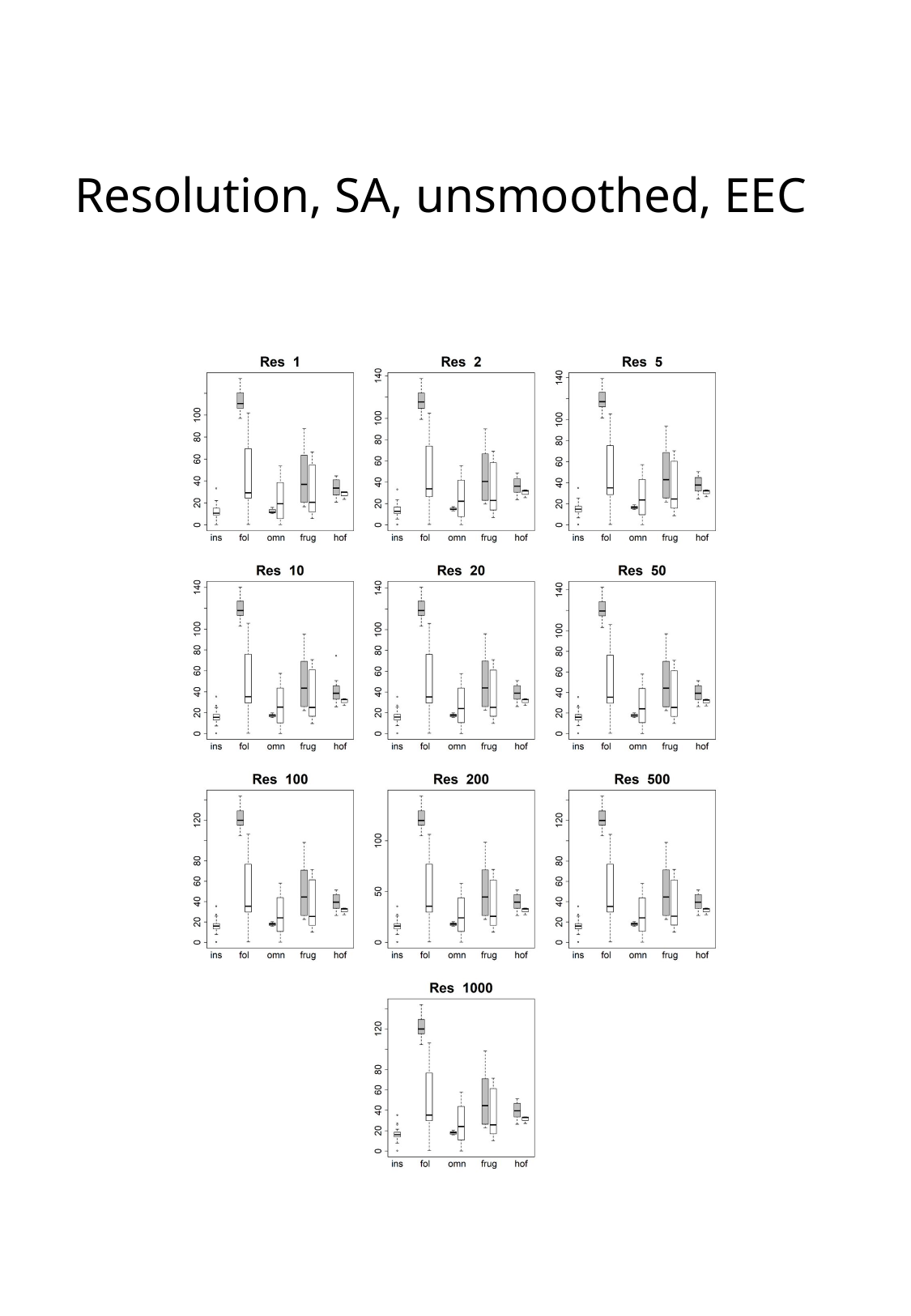

# Resolution, SA, unsmoothed, EEC

## Slide 41
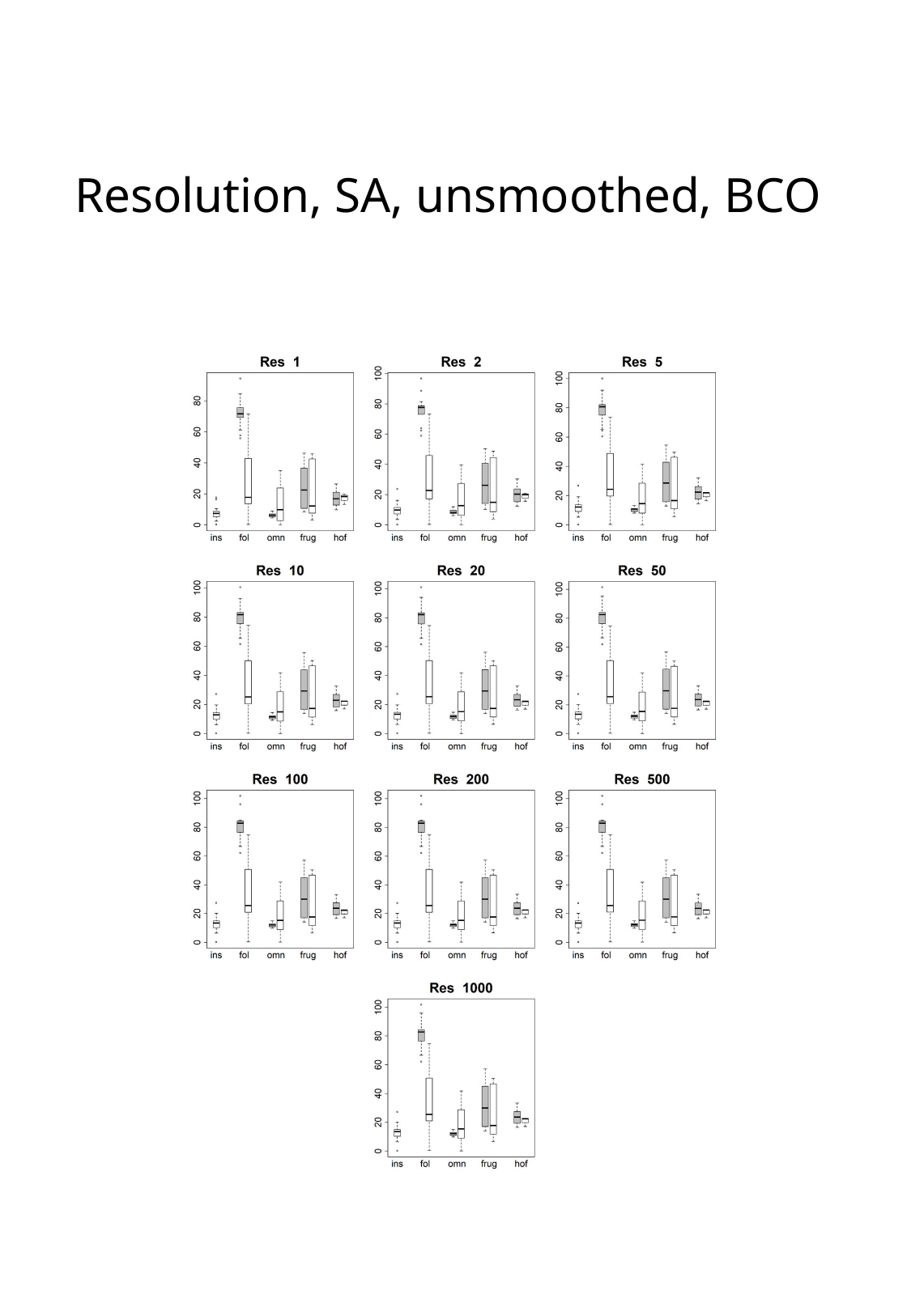

# Resolution, SA, unsmoothed, BCO

## Slide 42
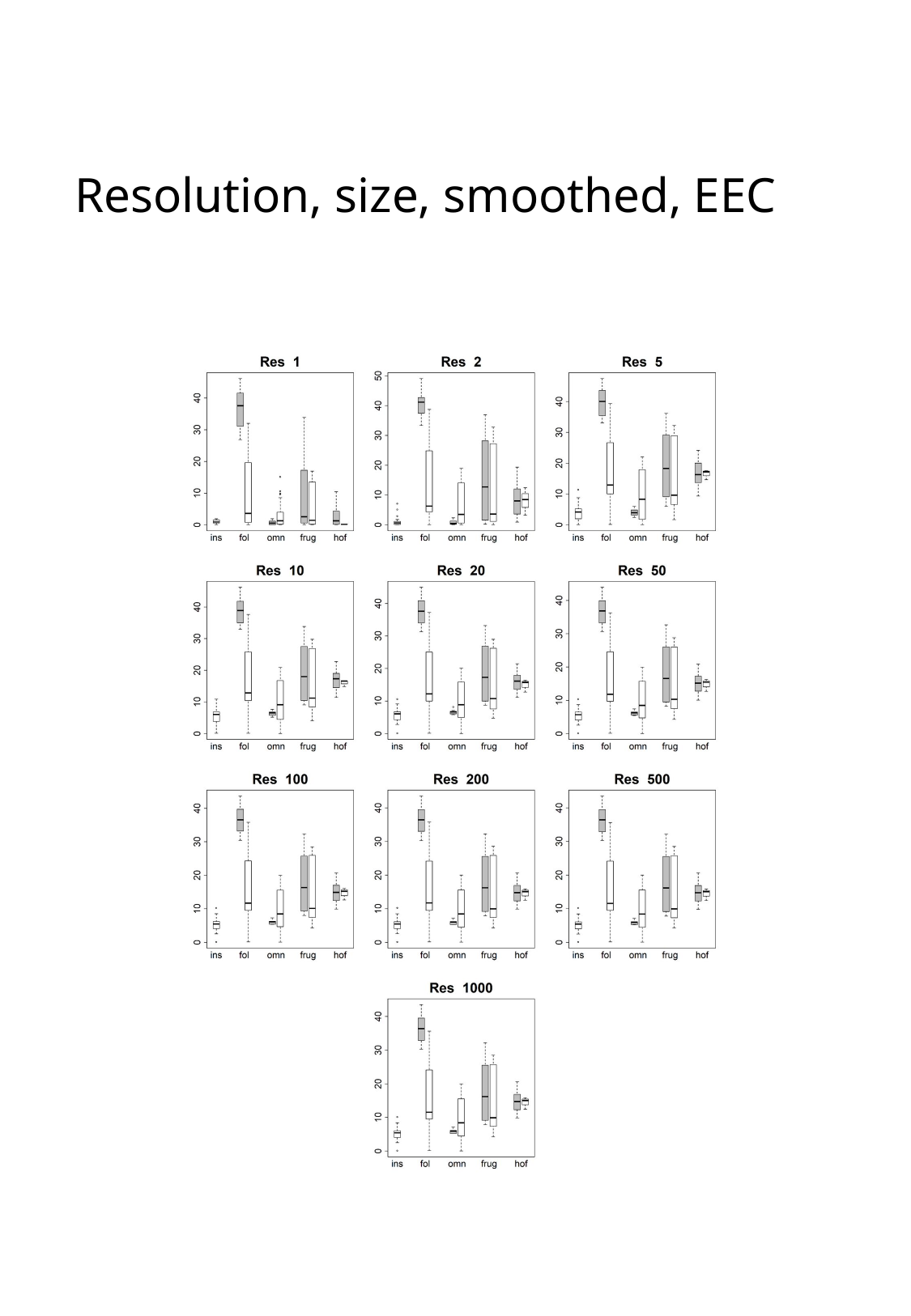

# Resolution, size, smoothed, EEC

## Slide 43
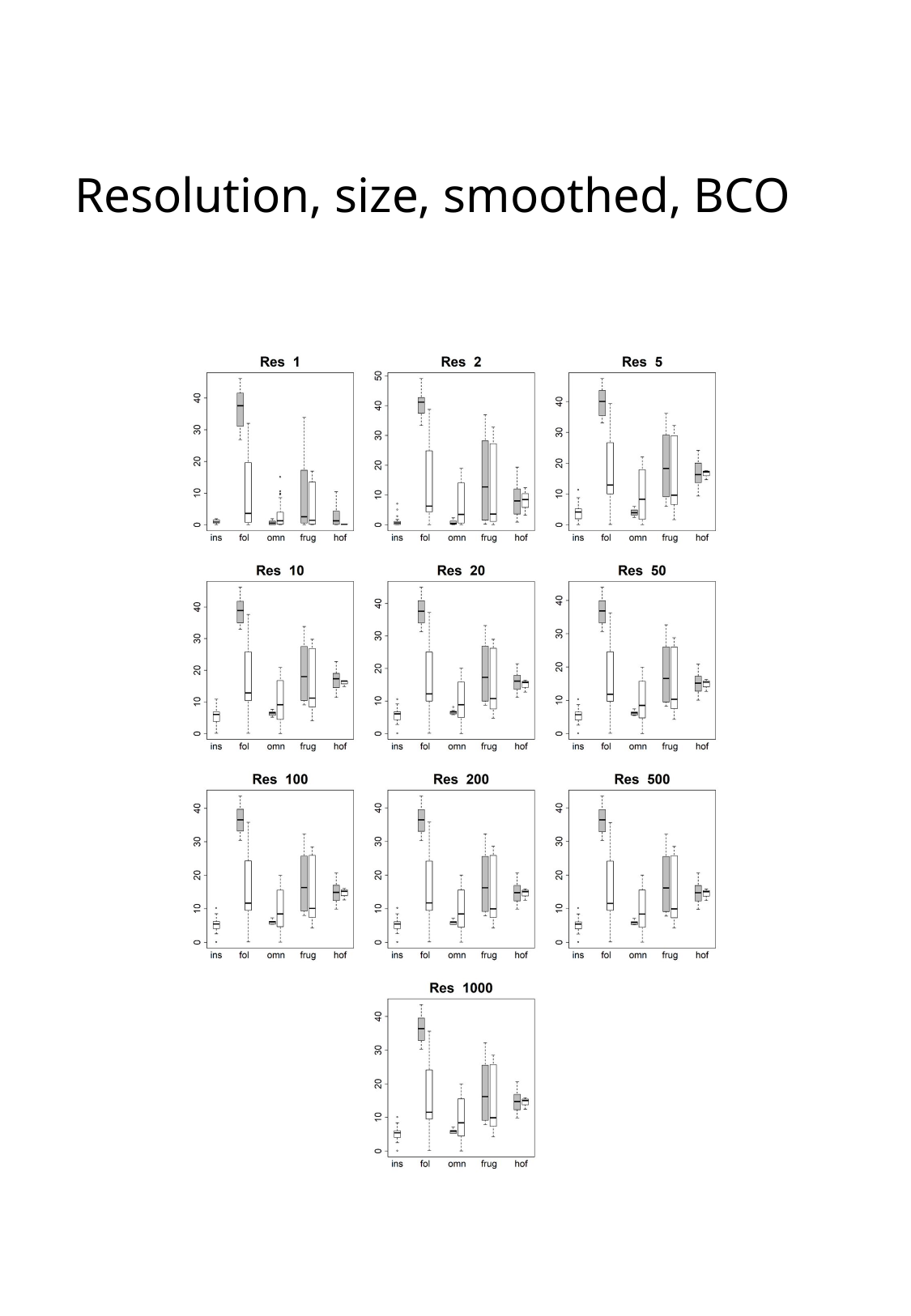

# Resolution, size, smoothed, BCO

## Slide 44
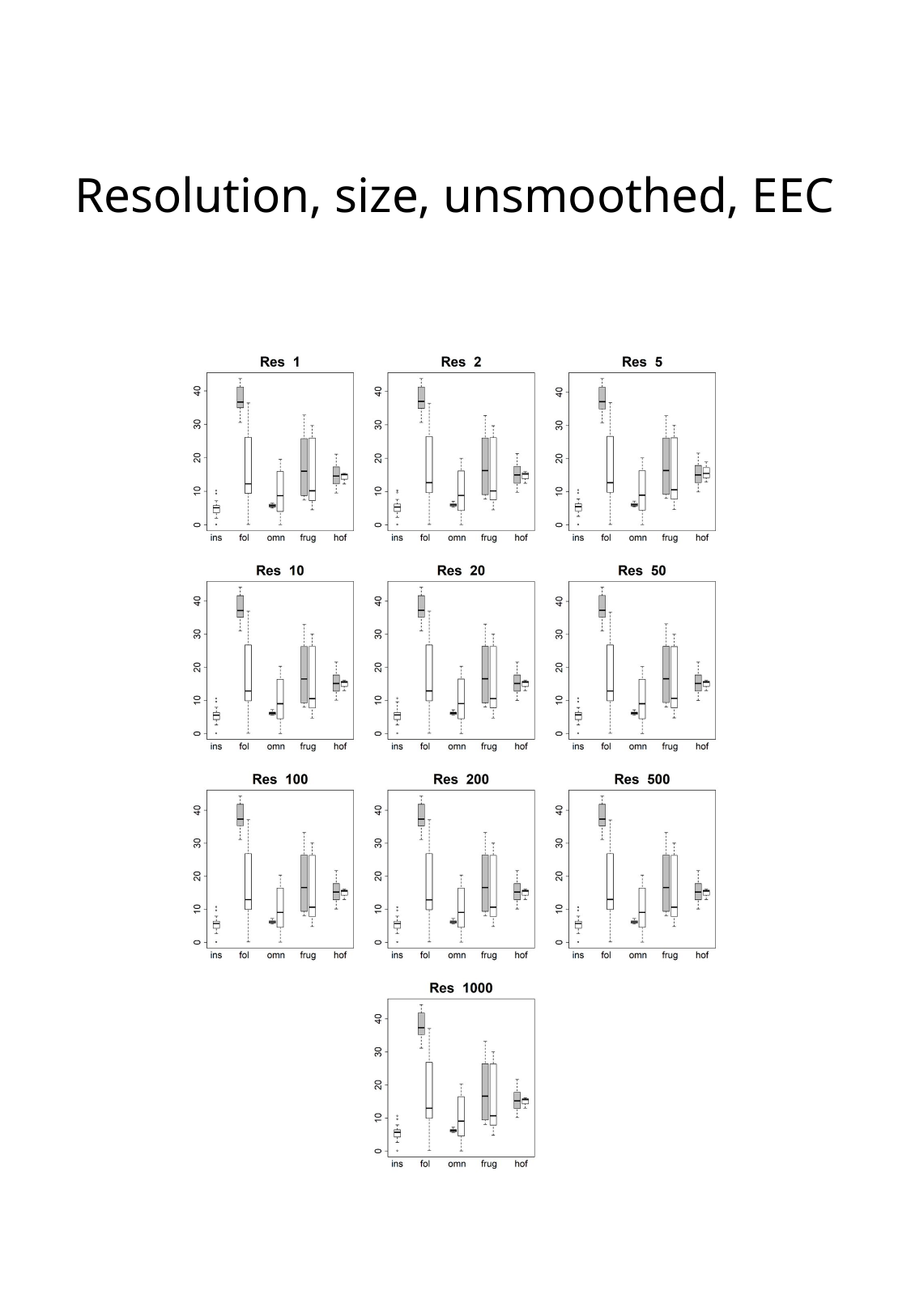

# Resolution, size, unsmoothed, EEC

## Slide 45
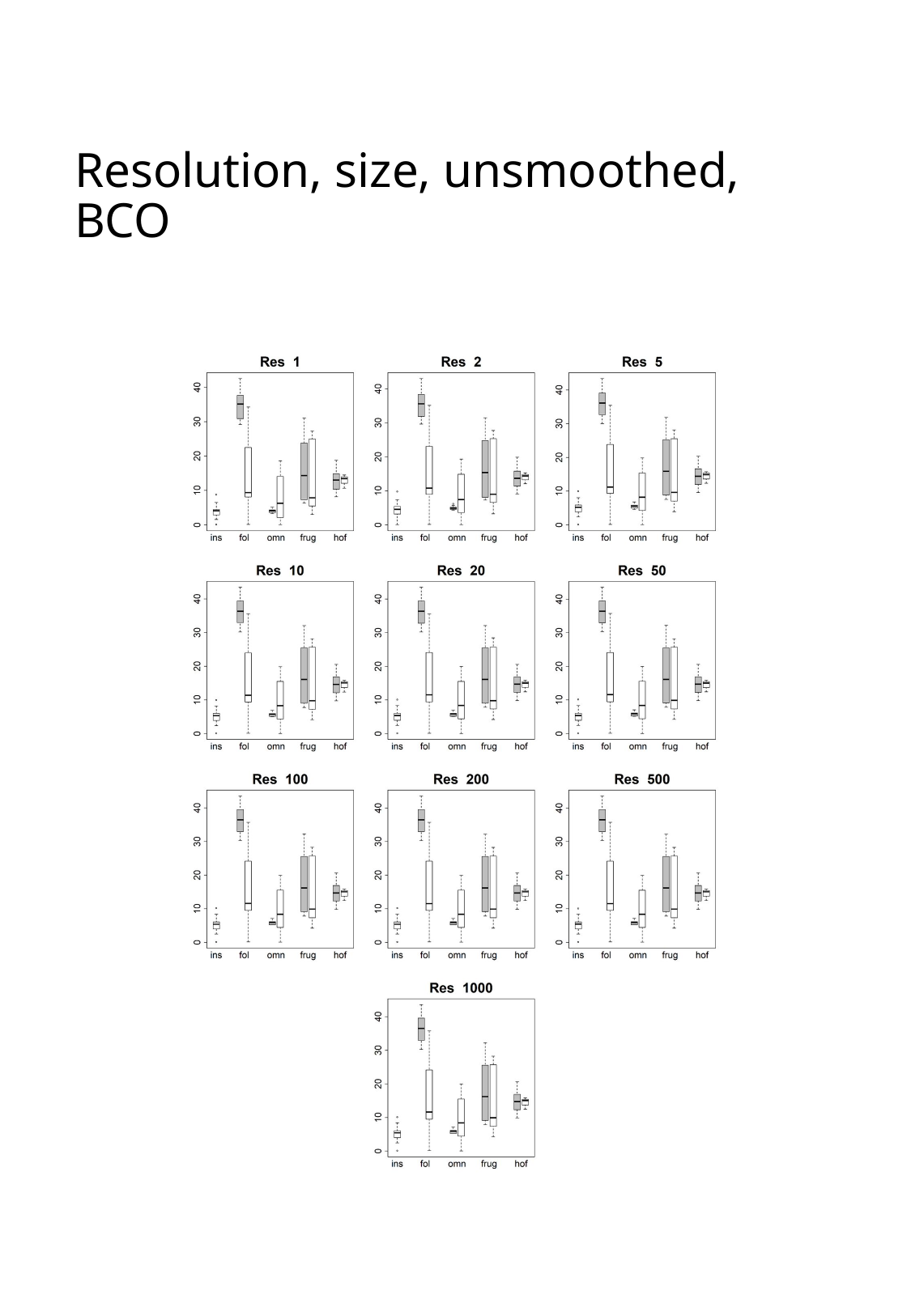

# Resolution, size, unsmoothed, BCO
