# Supplementary material for: Effects of cropping, smoothing, triangle count, and mesh resolution on 6 dental topographic metrics
Source: PLoS One. 2019 May 6;14(5):e0216229. doi: 10.1371/journal.pone.0216229 (PMC6502444; doi:10.1371/journal.pone.0216229)
Supplement: S2 Fig — Tukey HSD visualized for triangle count and resolution. (PPTX) [file pone.0216229.s011.pptx]

## Slide 1
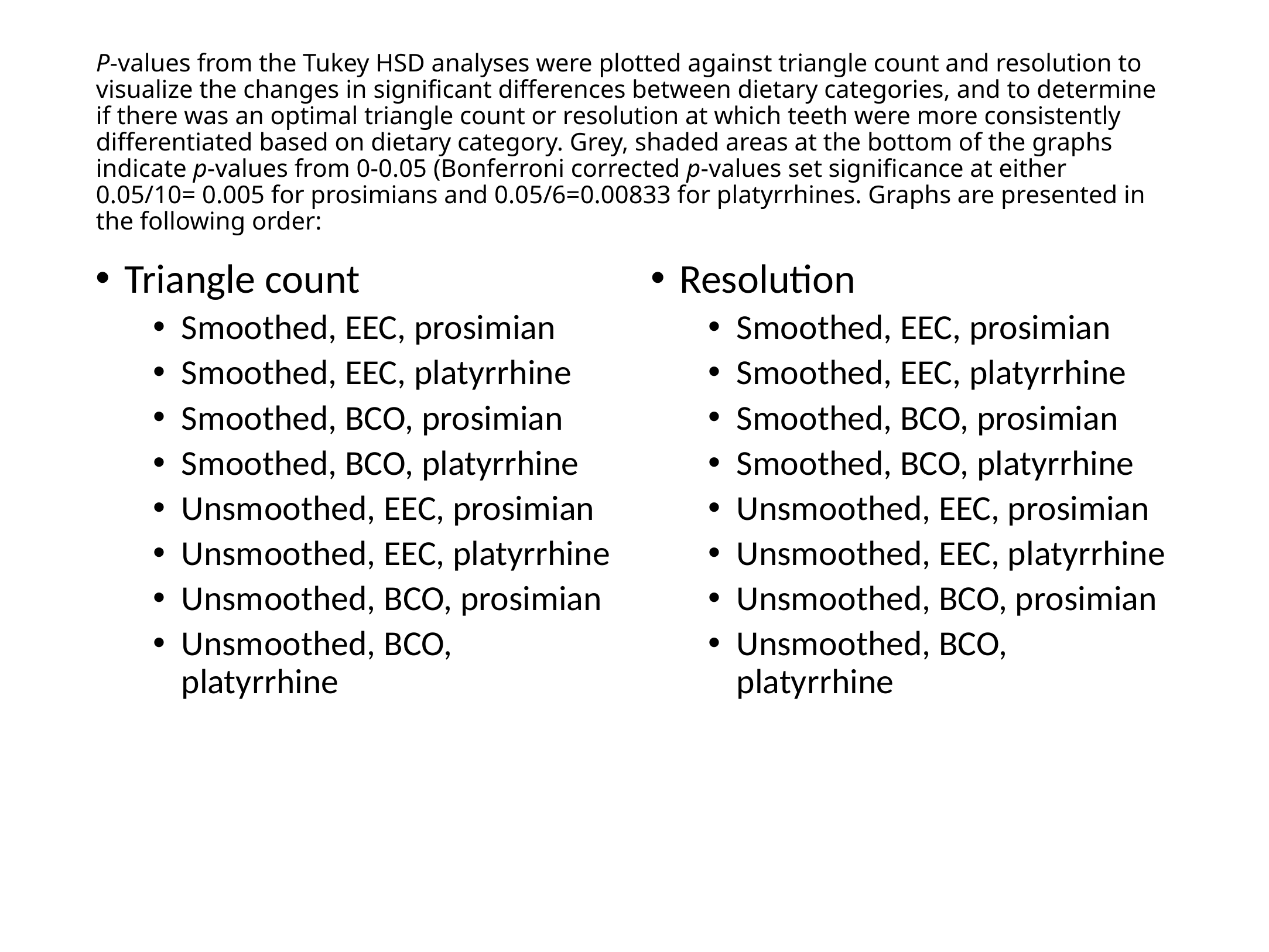

# P-values from the Tukey HSD analyses were plotted against triangle count and resolution to visualize the changes in significant differences between dietary categories, and to determine if there was an optimal triangle count or resolution at which teeth were more consistently differentiated based on dietary category. Grey, shaded areas at the bottom of the graphs indicate p-values from 0-0.05 (Bonferroni corrected p-values set significance at either 0.05/10= 0.005 for prosimians and 0.05/6=0.00833 for platyrrhines. Graphs are presented in the following order:
Triangle count
Smoothed, EEC, prosimian
Smoothed, EEC, platyrrhine
Smoothed, BCO, prosimian
Smoothed, BCO, platyrrhine
Unsmoothed, EEC, prosimian
Unsmoothed, EEC, platyrrhine
Unsmoothed, BCO, prosimian
Unsmoothed, BCO, platyrrhine
Resolution
Smoothed, EEC, prosimian
Smoothed, EEC, platyrrhine
Smoothed, BCO, prosimian
Smoothed, BCO, platyrrhine
Unsmoothed, EEC, prosimian
Unsmoothed, EEC, platyrrhine
Unsmoothed, BCO, prosimian
Unsmoothed, BCO, platyrrhine

## Slide 2
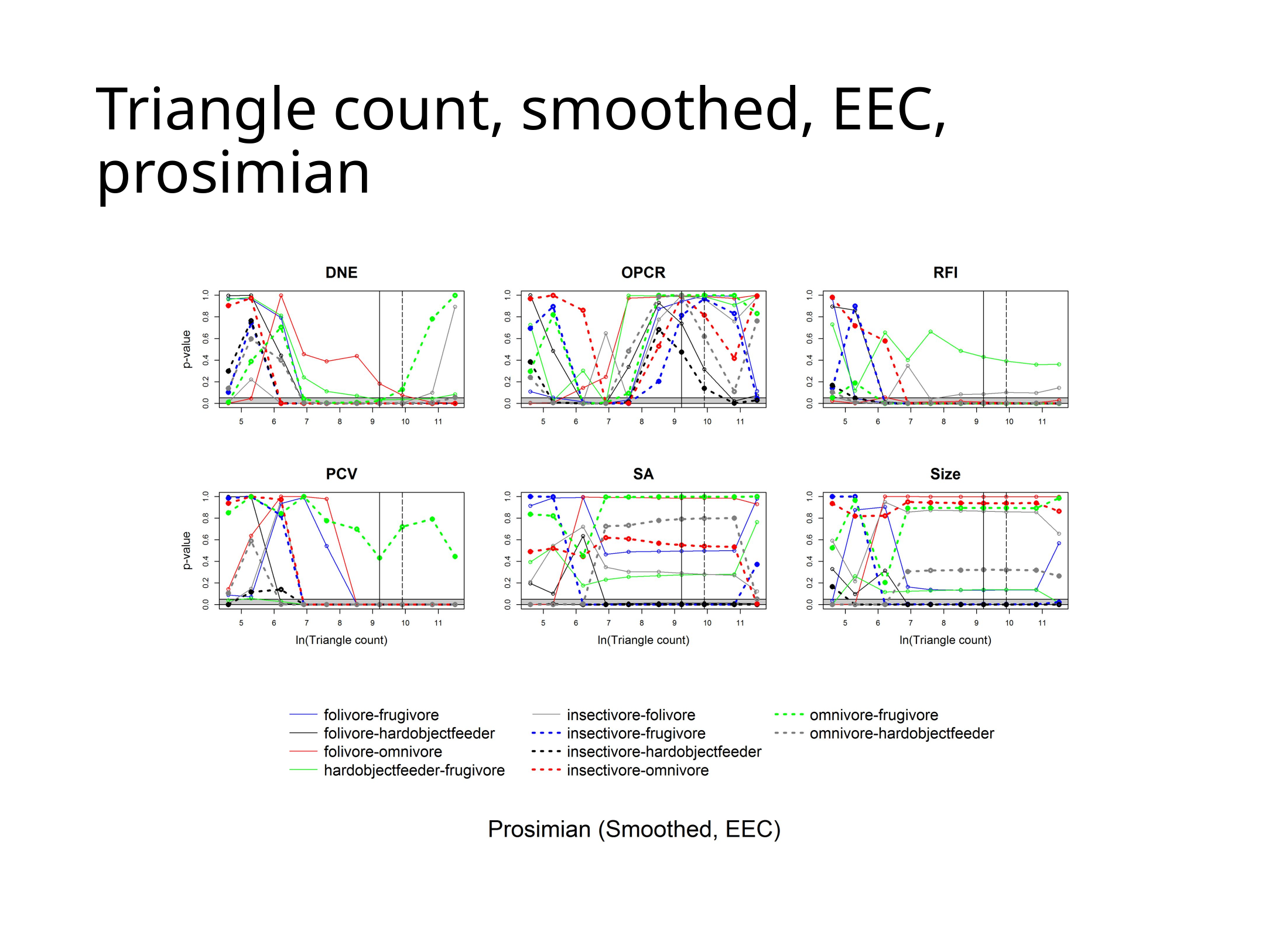

# Triangle count, smoothed, EEC, prosimian

## Slide 3
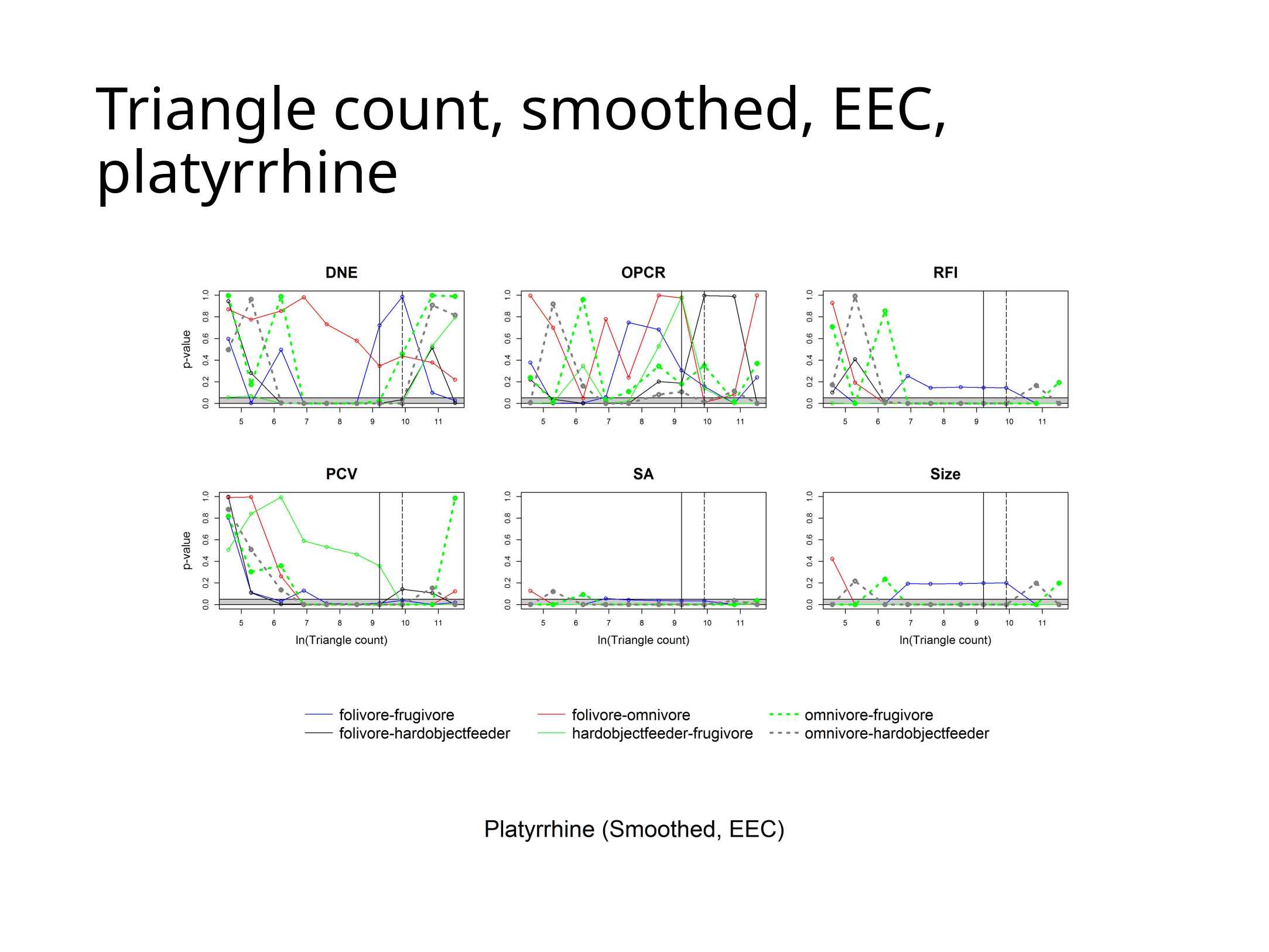

# Triangle count, smoothed, EEC, platyrrhine

## Slide 4
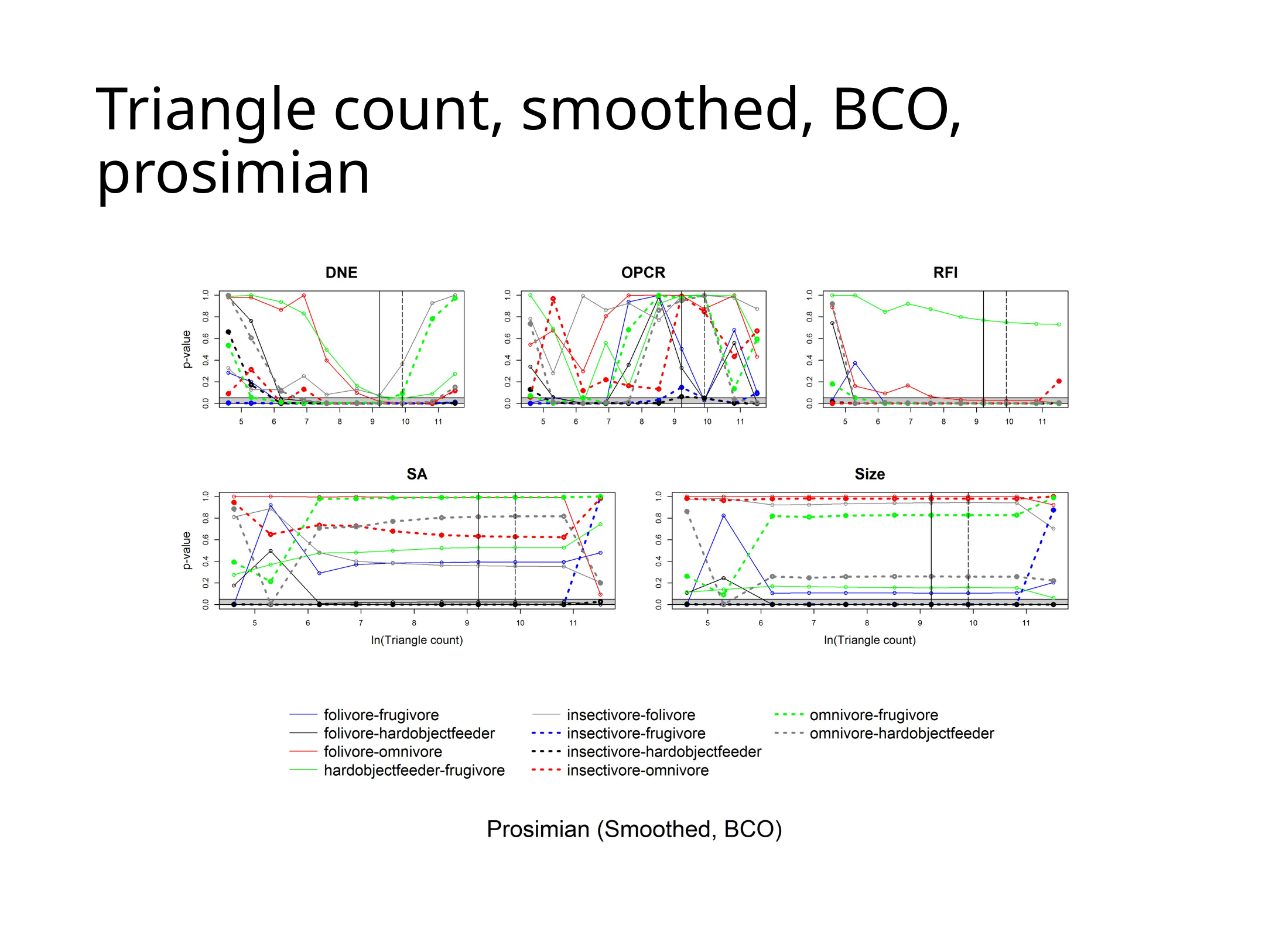

# Triangle count, smoothed, BCO, prosimian

## Slide 5
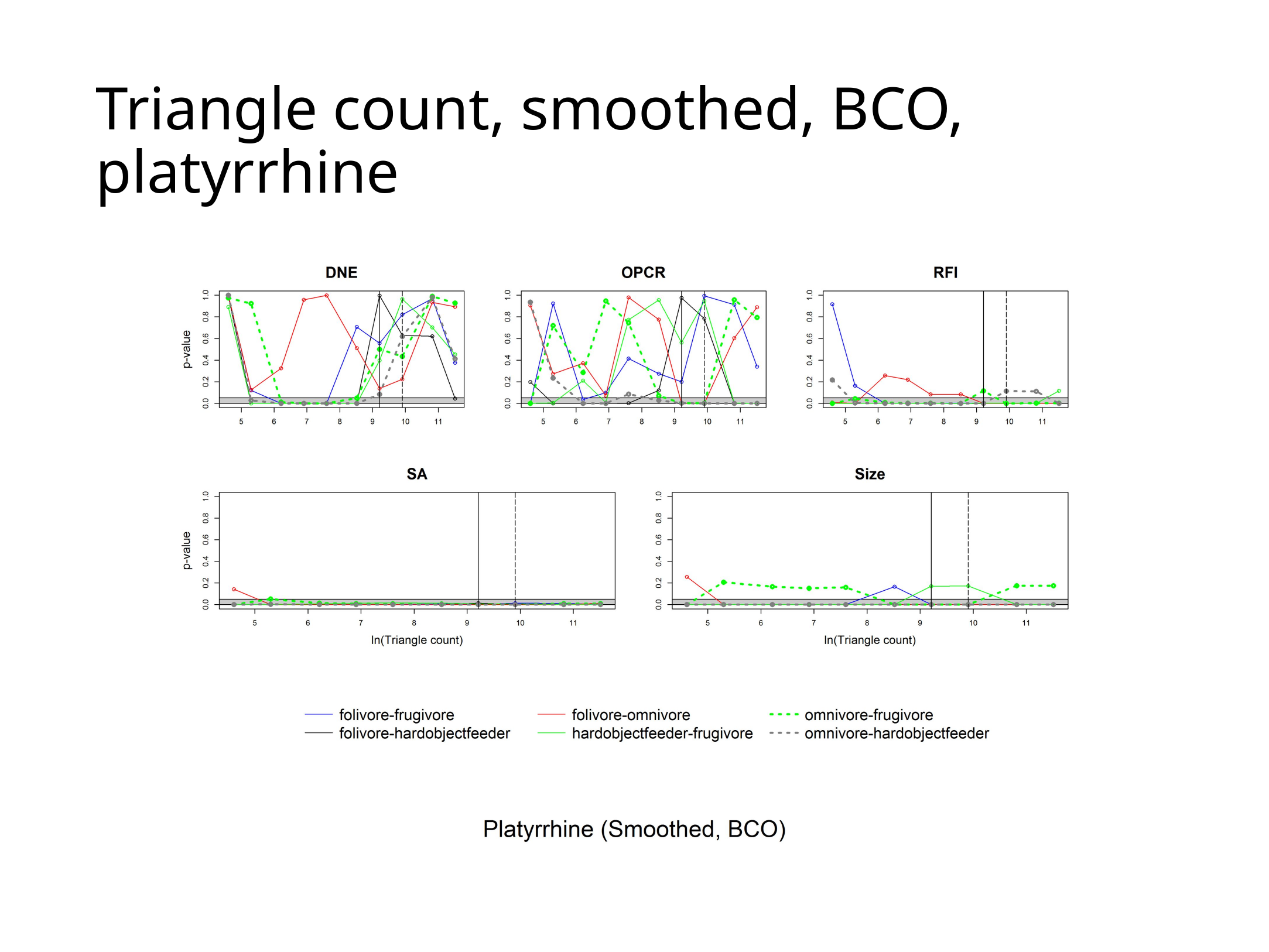

# Triangle count, smoothed, BCO, platyrrhine

## Slide 6
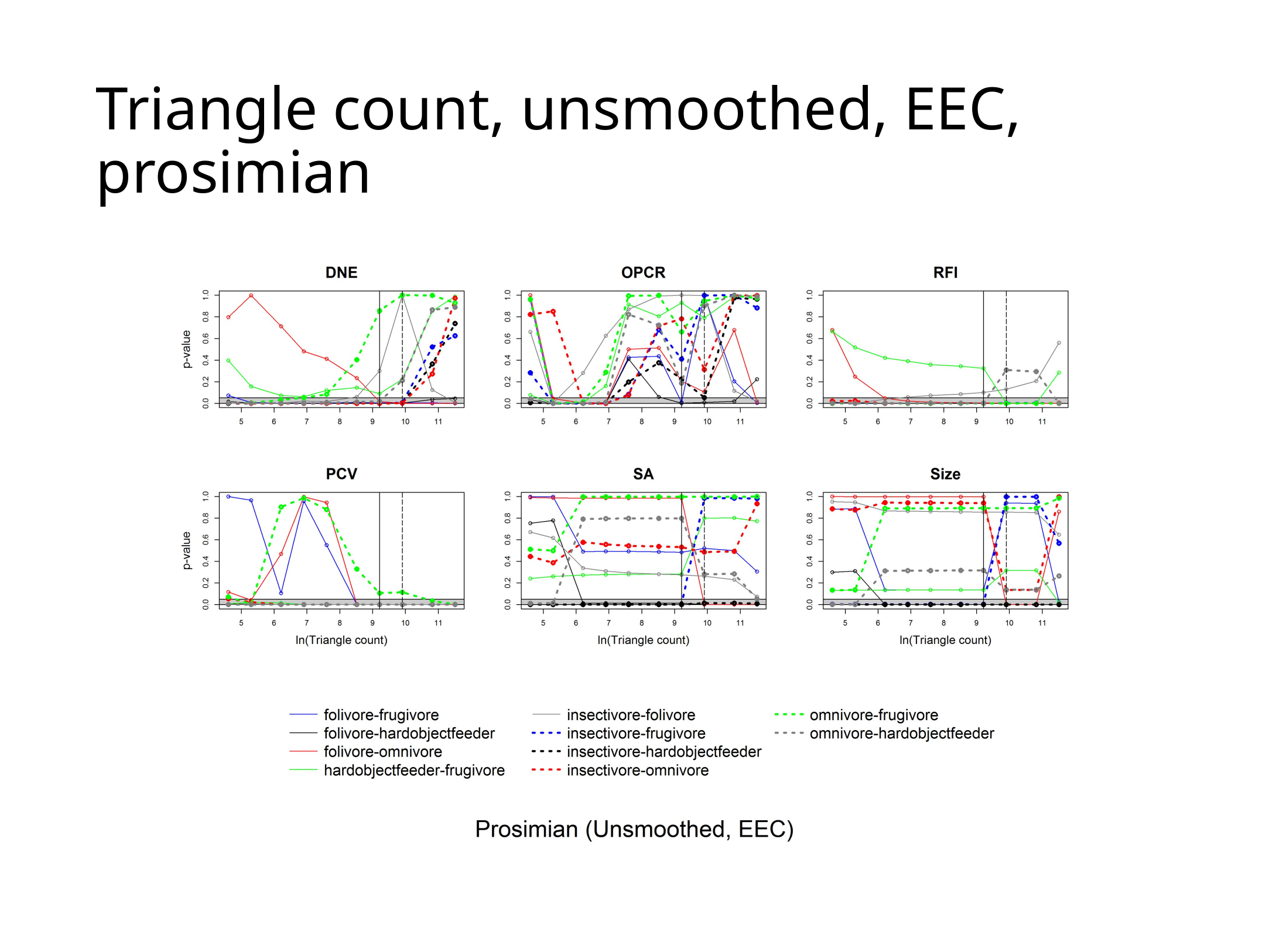

# Triangle count, unsmoothed, EEC, prosimian

## Slide 7
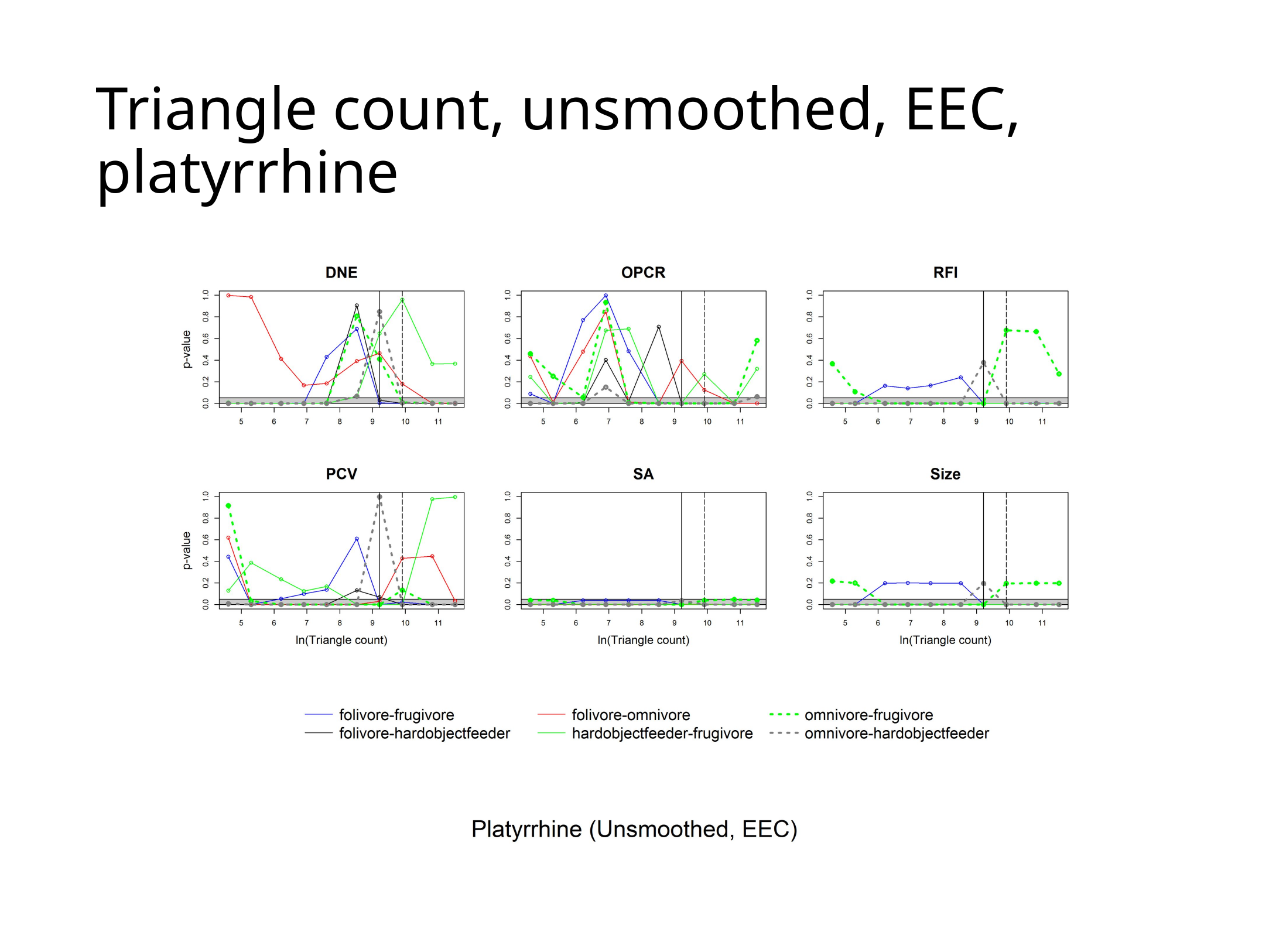

# Triangle count, unsmoothed, EEC, platyrrhine

## Slide 8
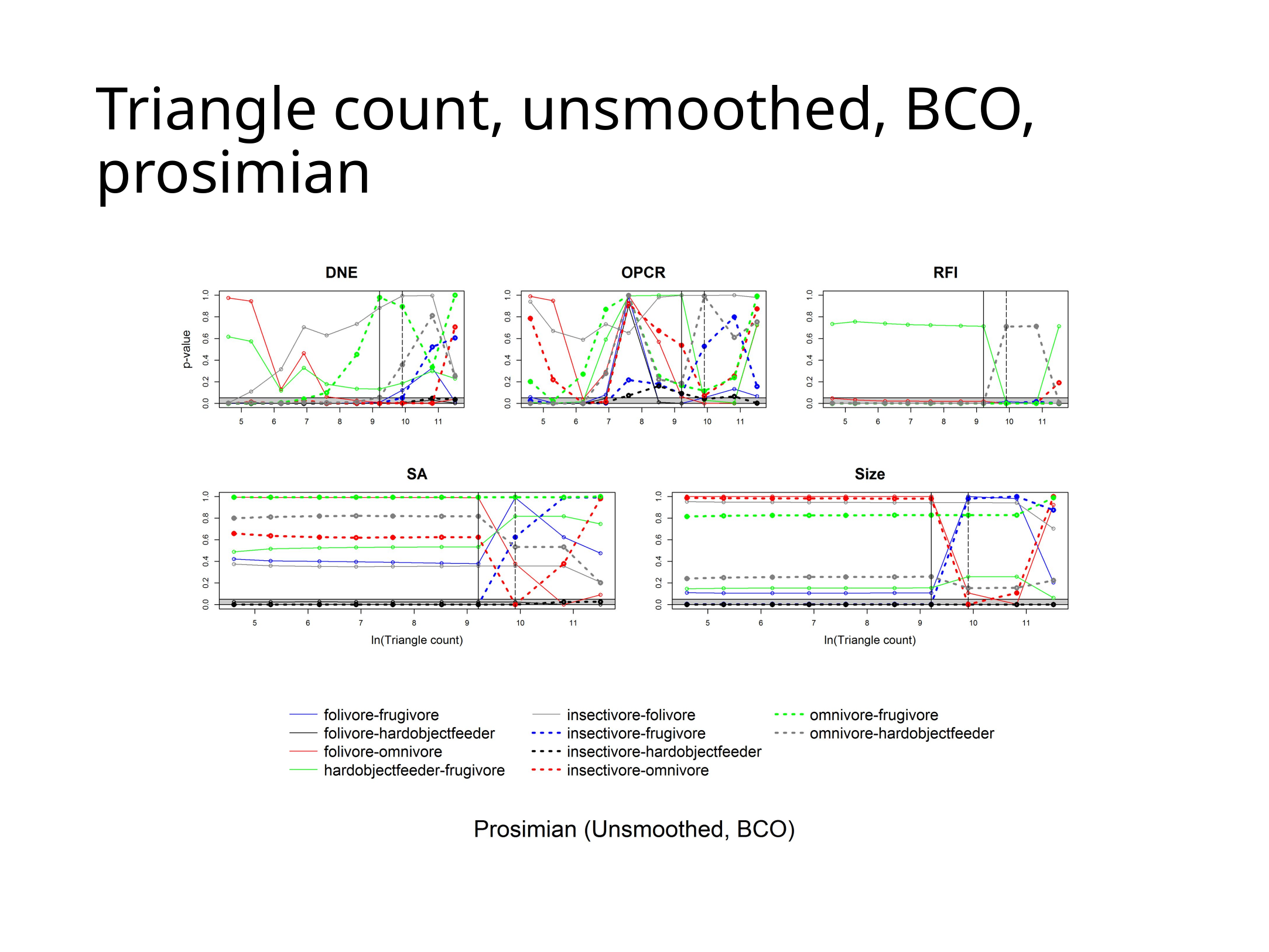

# Triangle count, unsmoothed, BCO, prosimian

## Slide 9
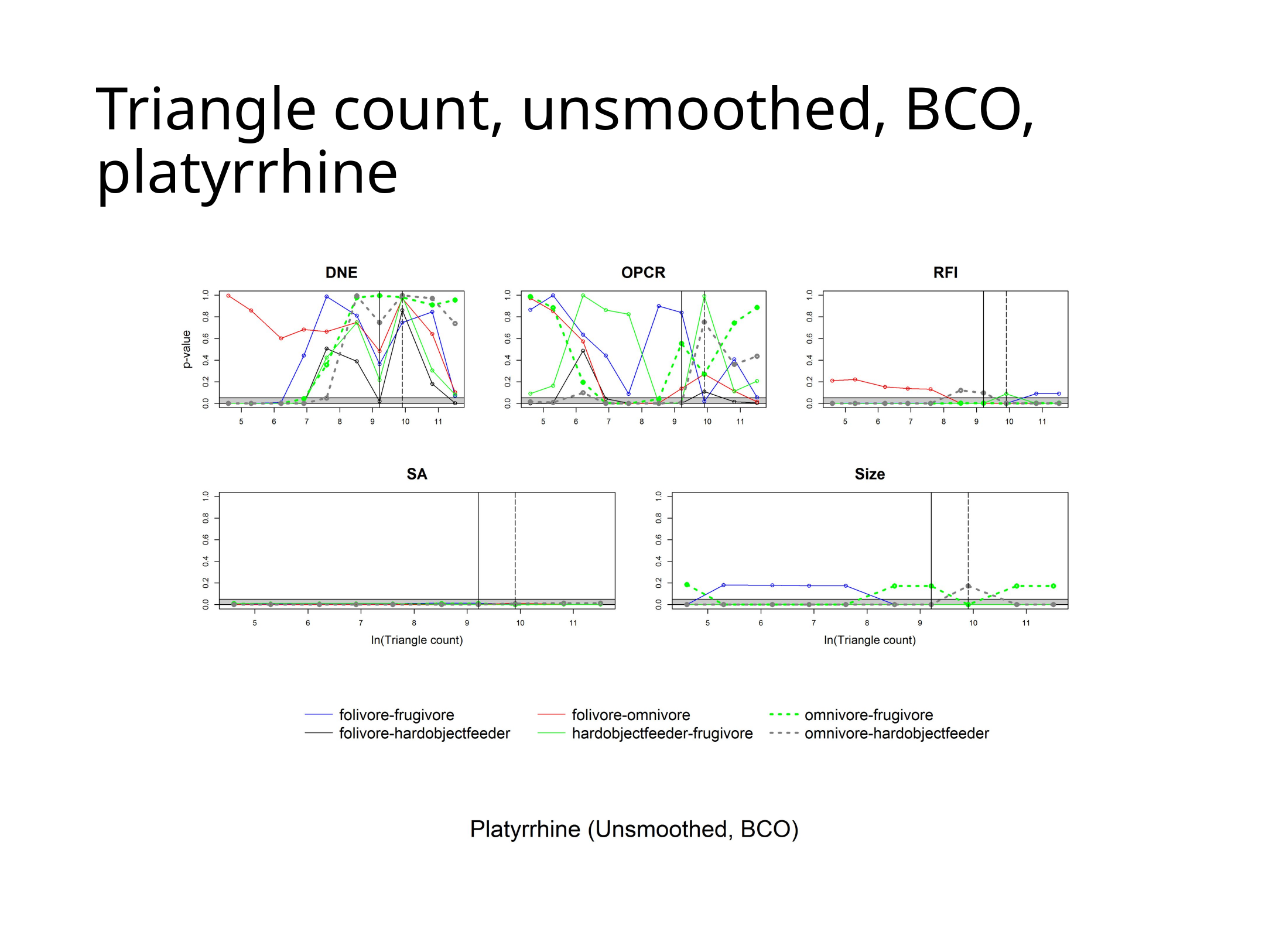

# Triangle count, unsmoothed, BCO, platyrrhine

## Slide 10
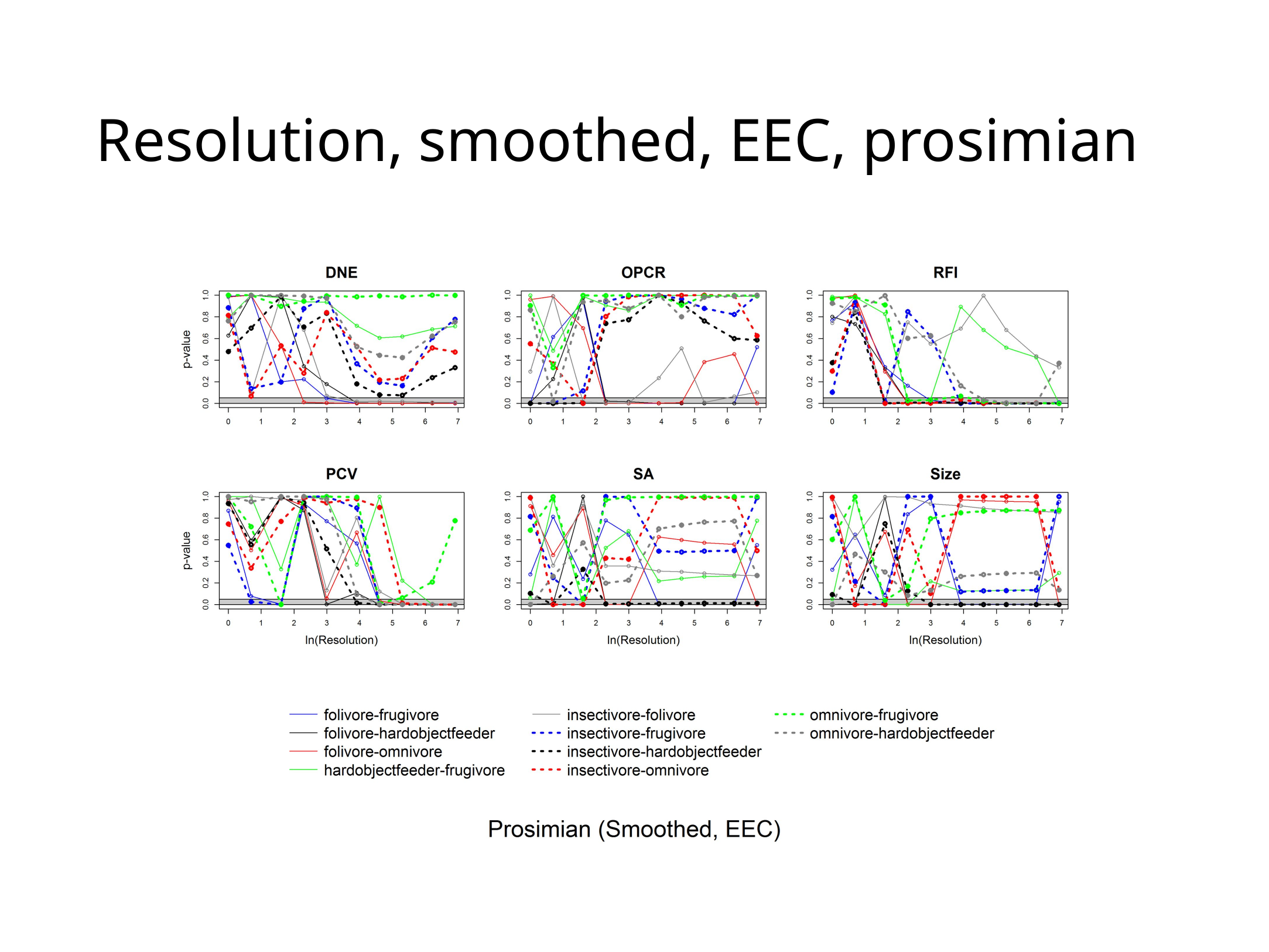

# Resolution, smoothed, EEC, prosimian

## Slide 11
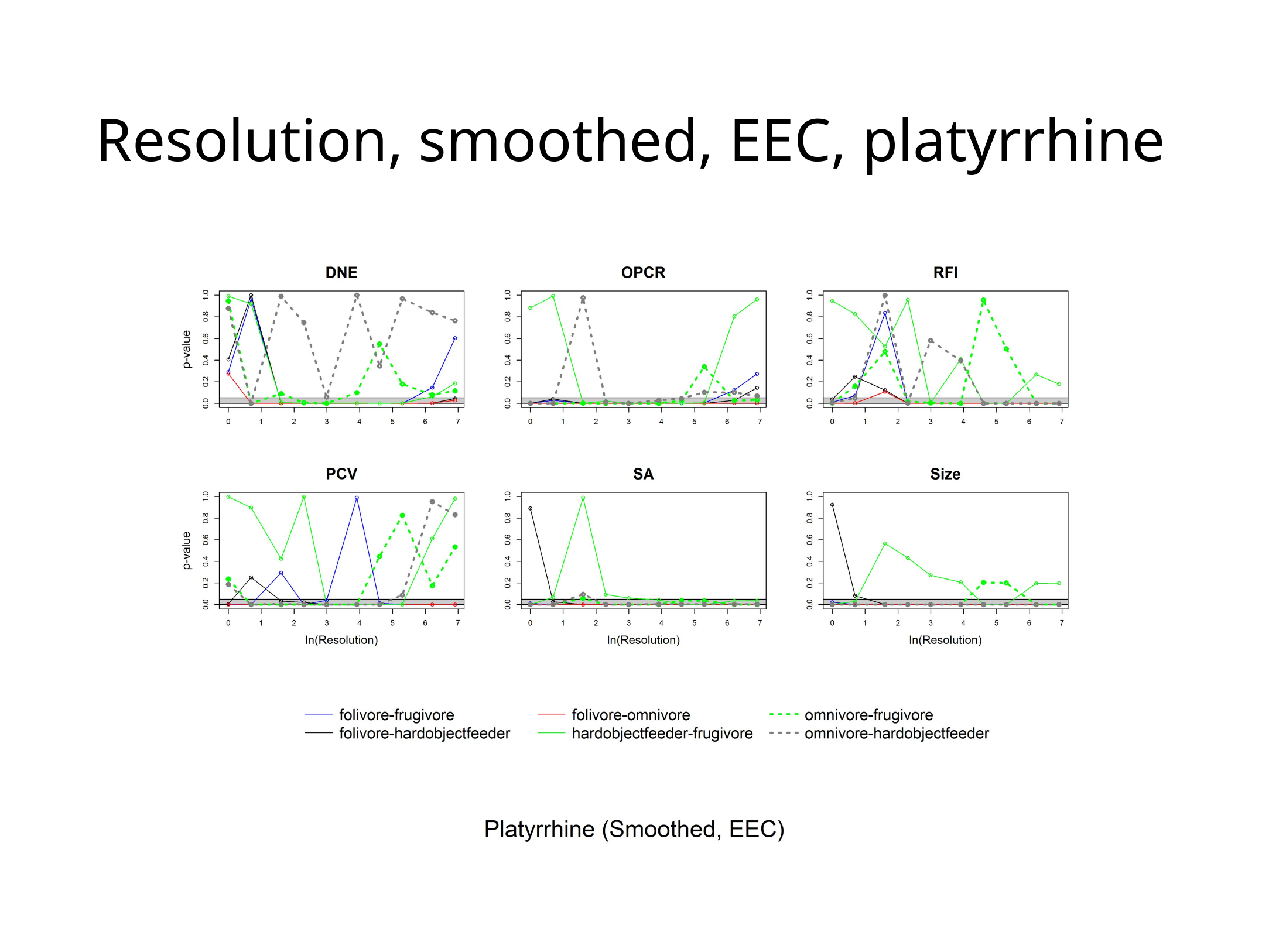

# Resolution, smoothed, EEC, platyrrhine

## Slide 12
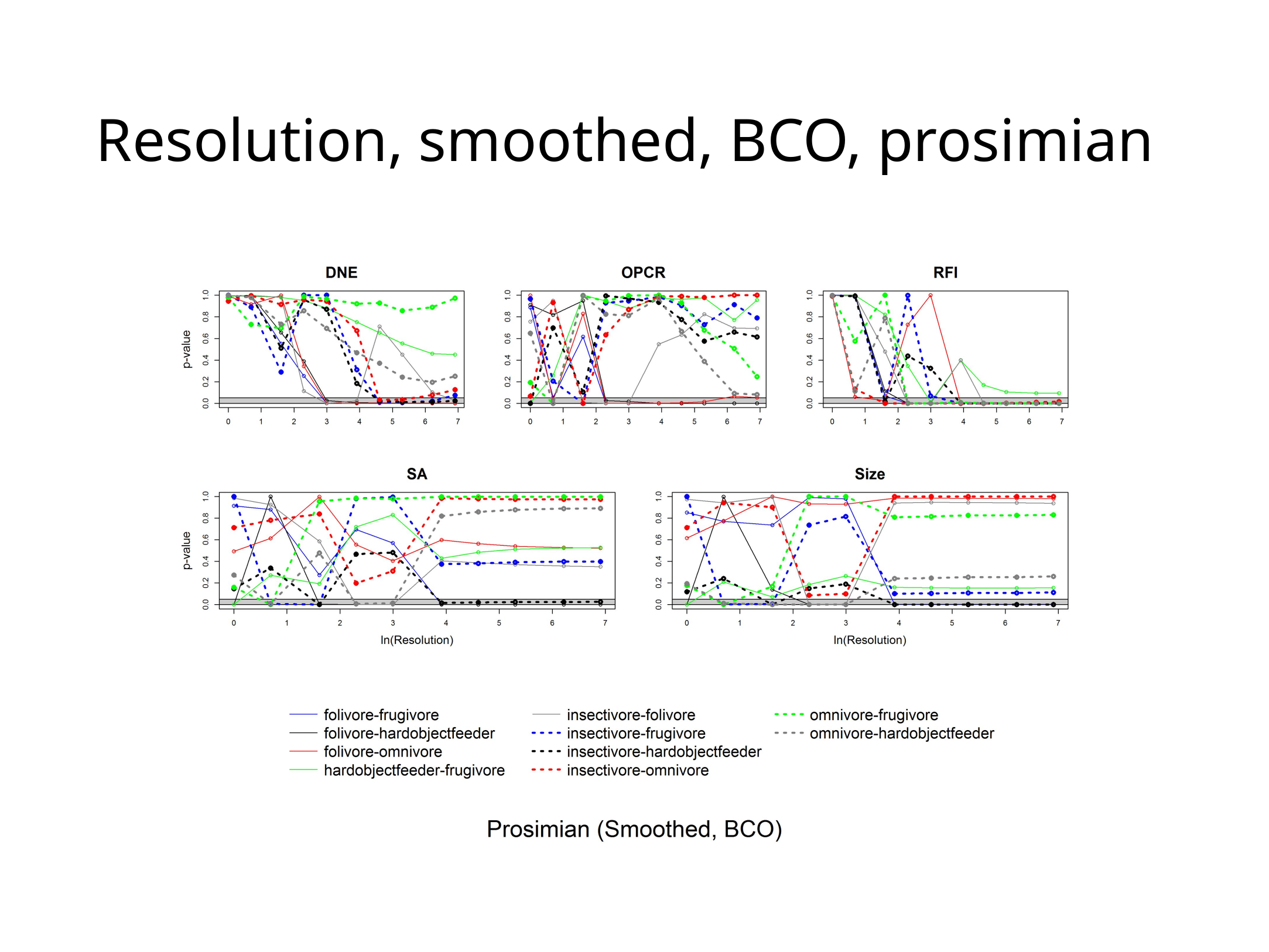

# Resolution, smoothed, BCO, prosimian

## Slide 13
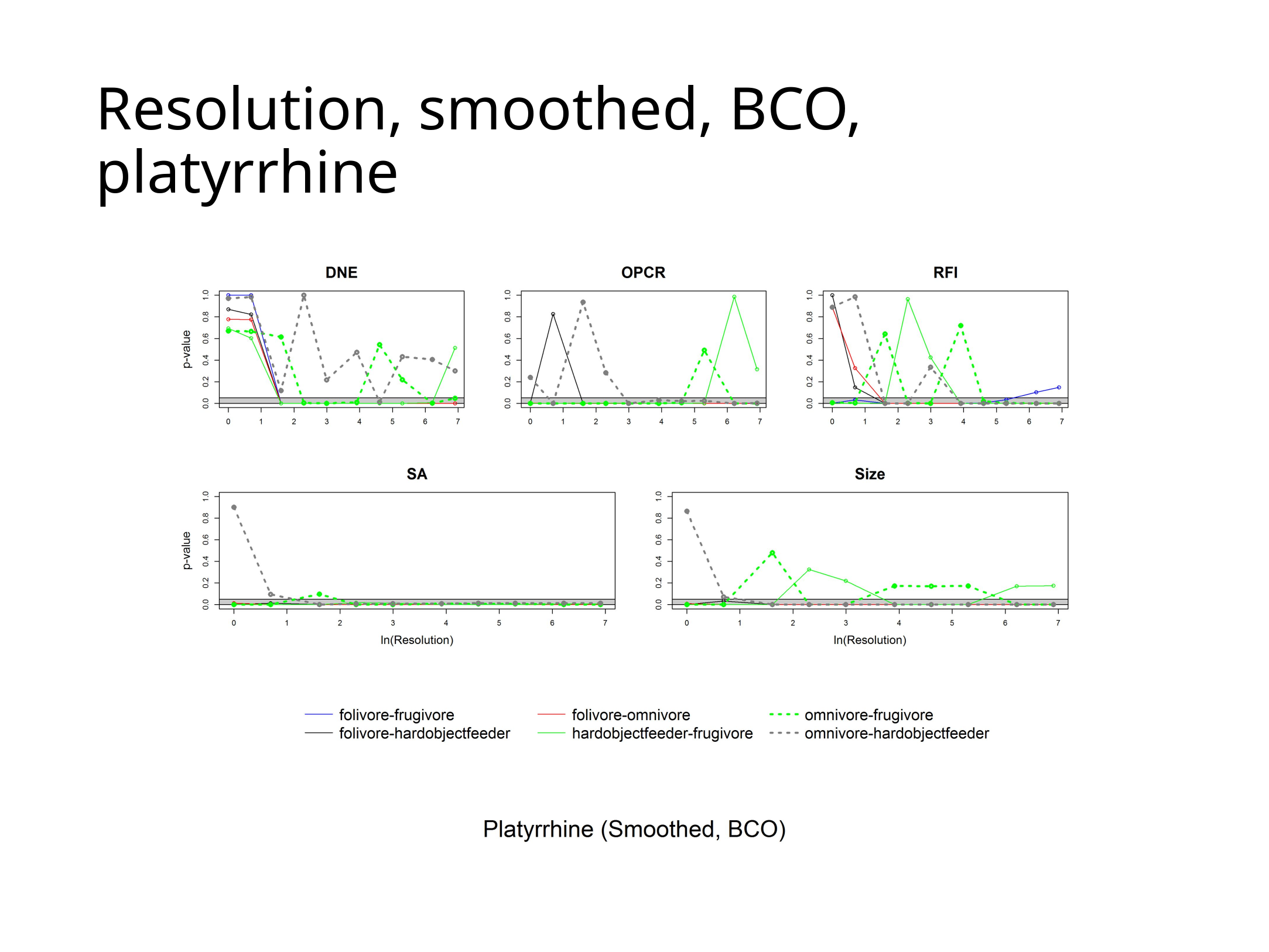

# Resolution, smoothed, BCO, platyrrhine

## Slide 14
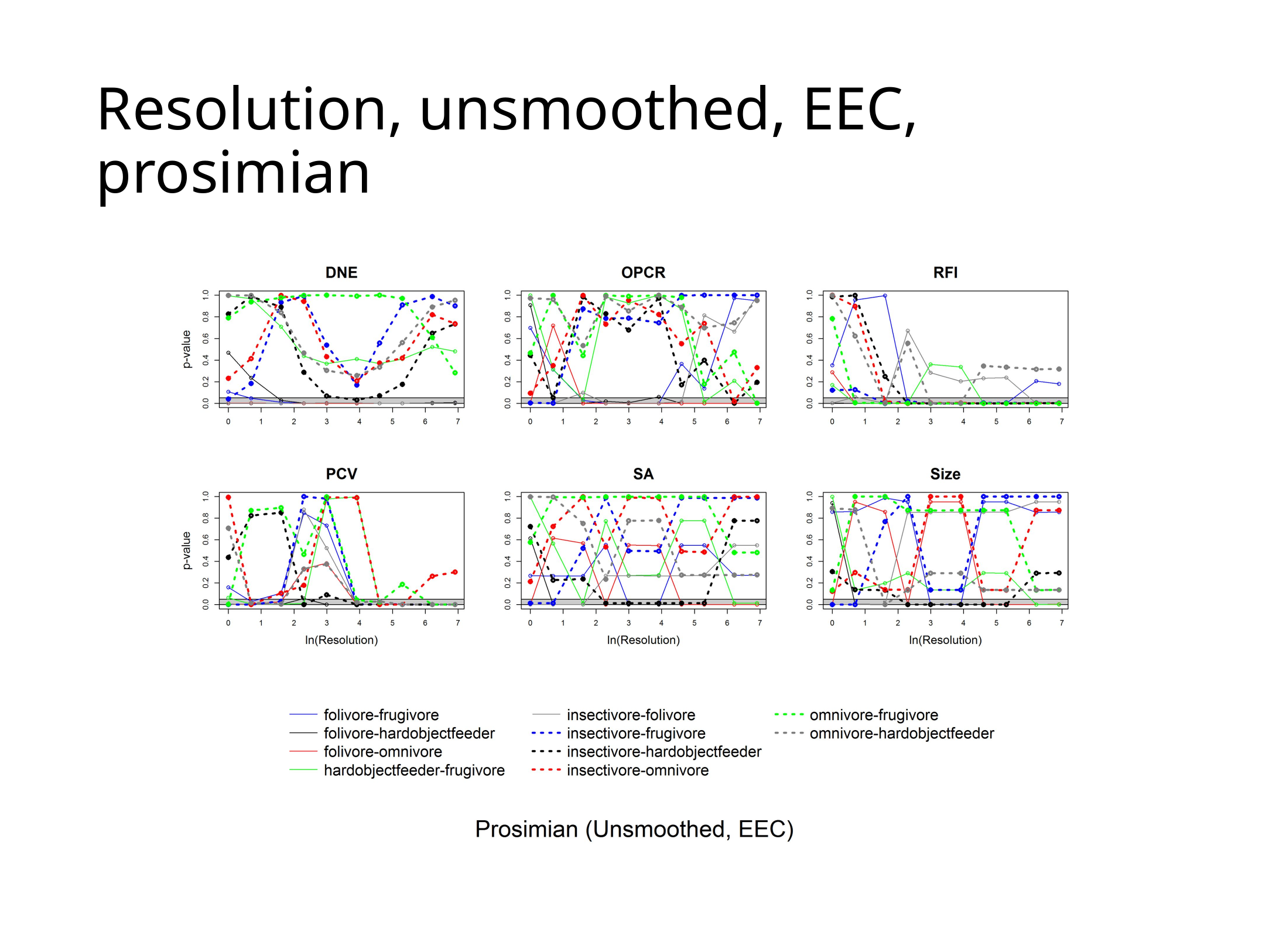

# Resolution, unsmoothed, EEC, prosimian

## Slide 15
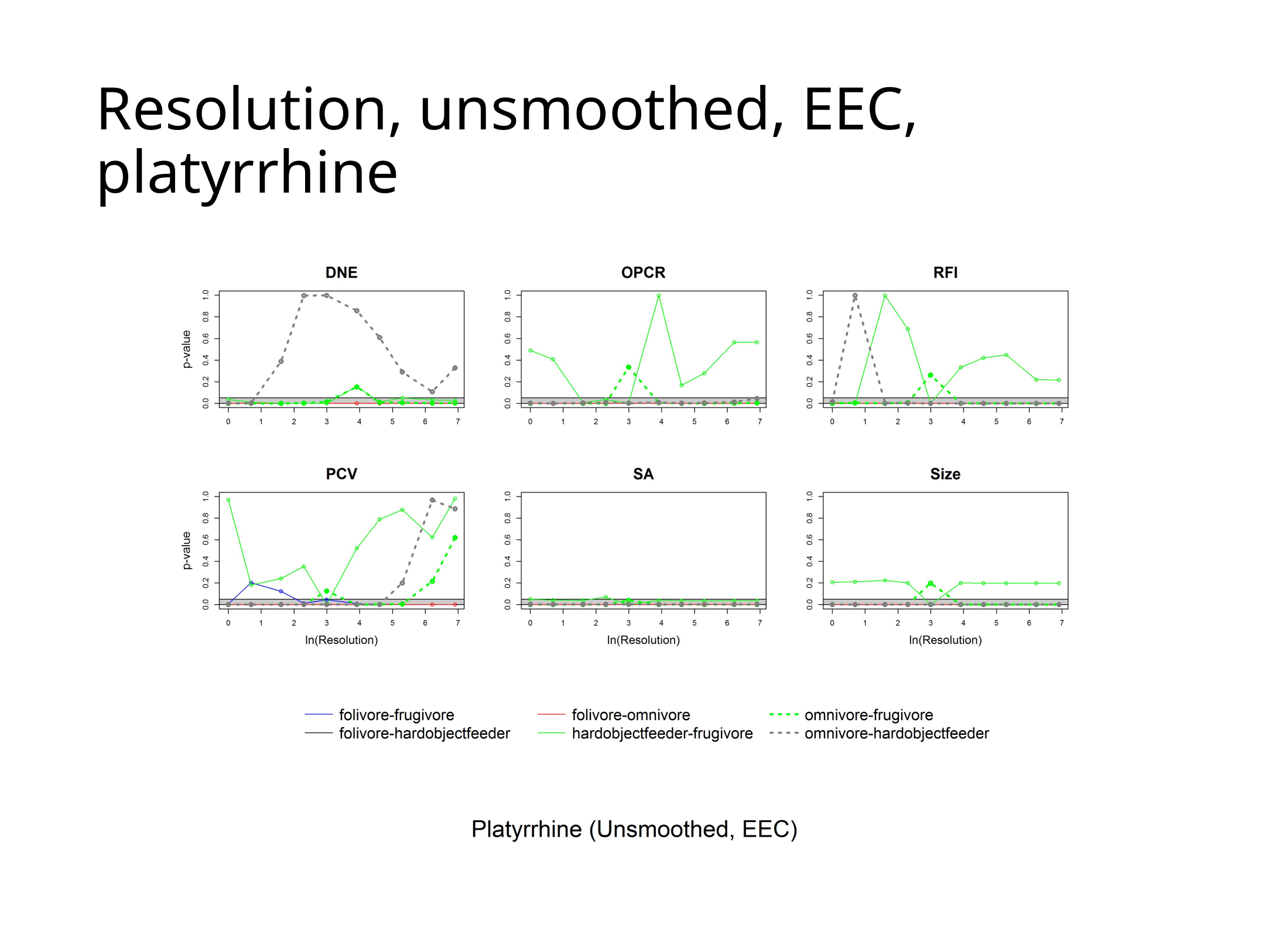

# Resolution, unsmoothed, EEC, platyrrhine

## Slide 16
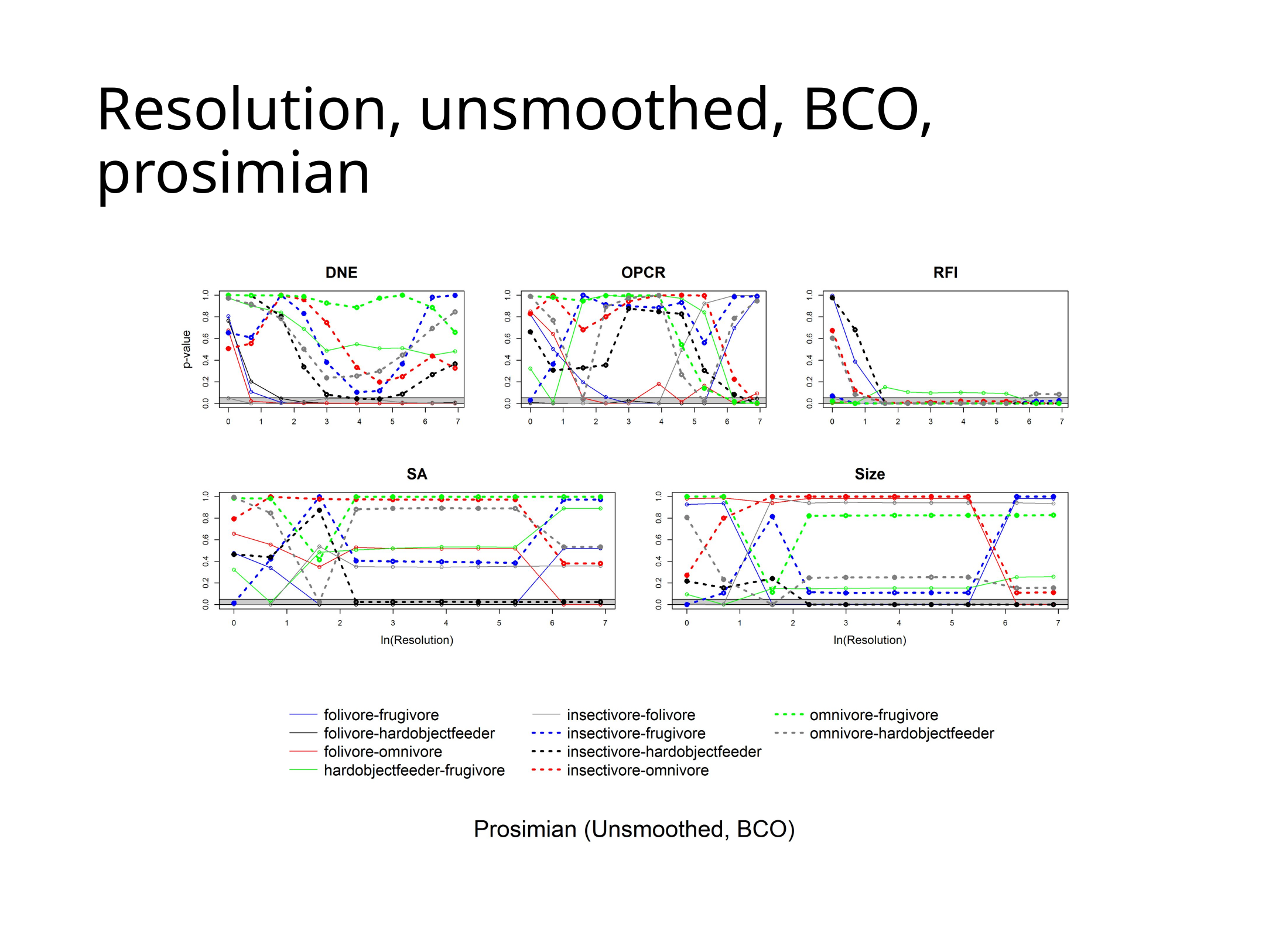

# Resolution, unsmoothed, BCO, prosimian

## Slide 17
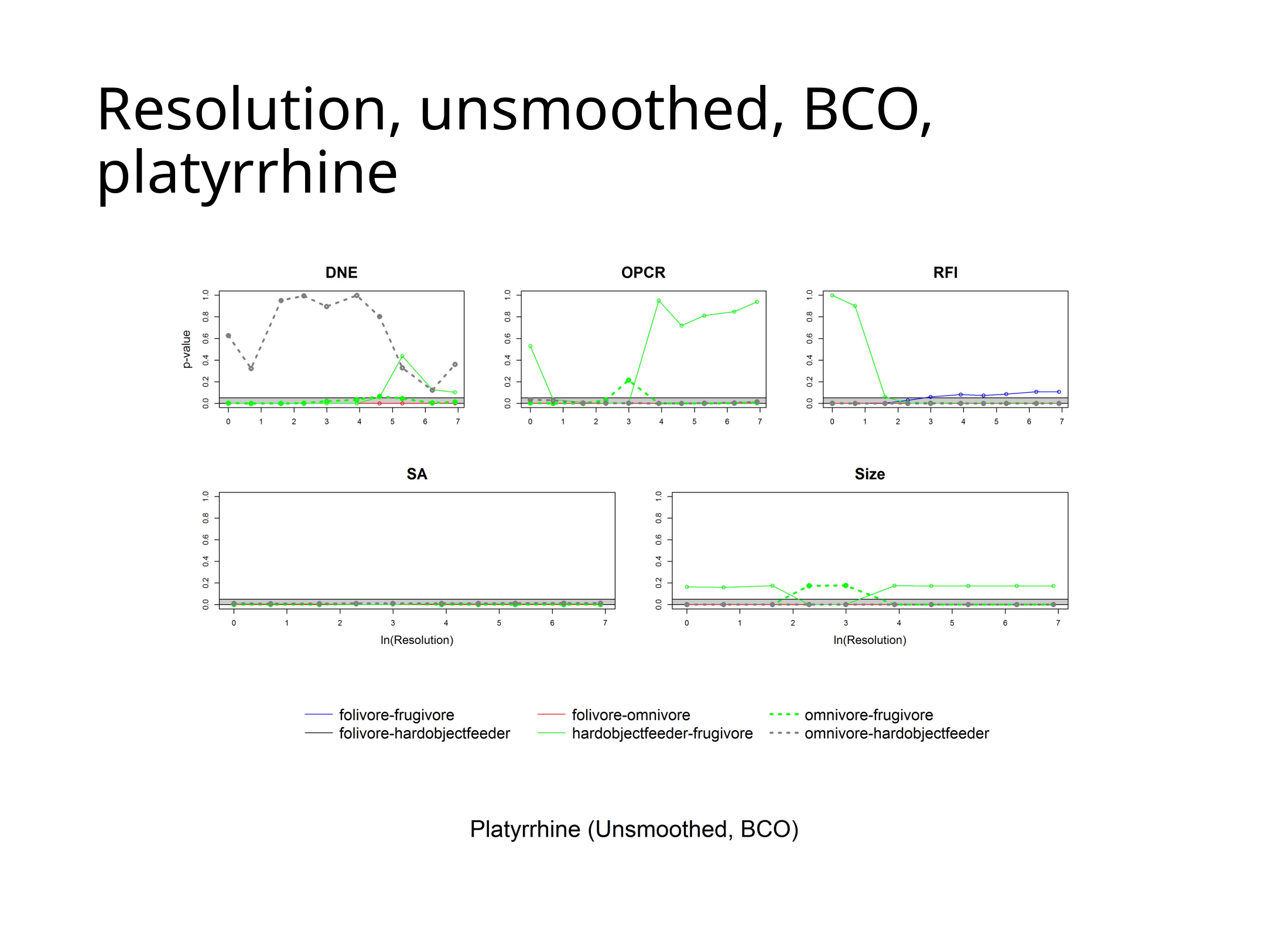

# Resolution, unsmoothed, BCO, platyrrhine
