# Supplementary material for: Effects of cropping, smoothing, triangle count, and mesh resolution on 6 dental topographic metrics
Source: PLoS One. 2019 May 6;14(5):e0216229. doi: 10.1371/journal.pone.0216229 (PMC6502444; doi:10.1371/journal.pone.0216229)
Supplement: S3 Fig — Linear plots, effect of triangle count and resolution on topographic variables. (PPTX) [file pone.0216229.s012.pptx]

## Slide 1
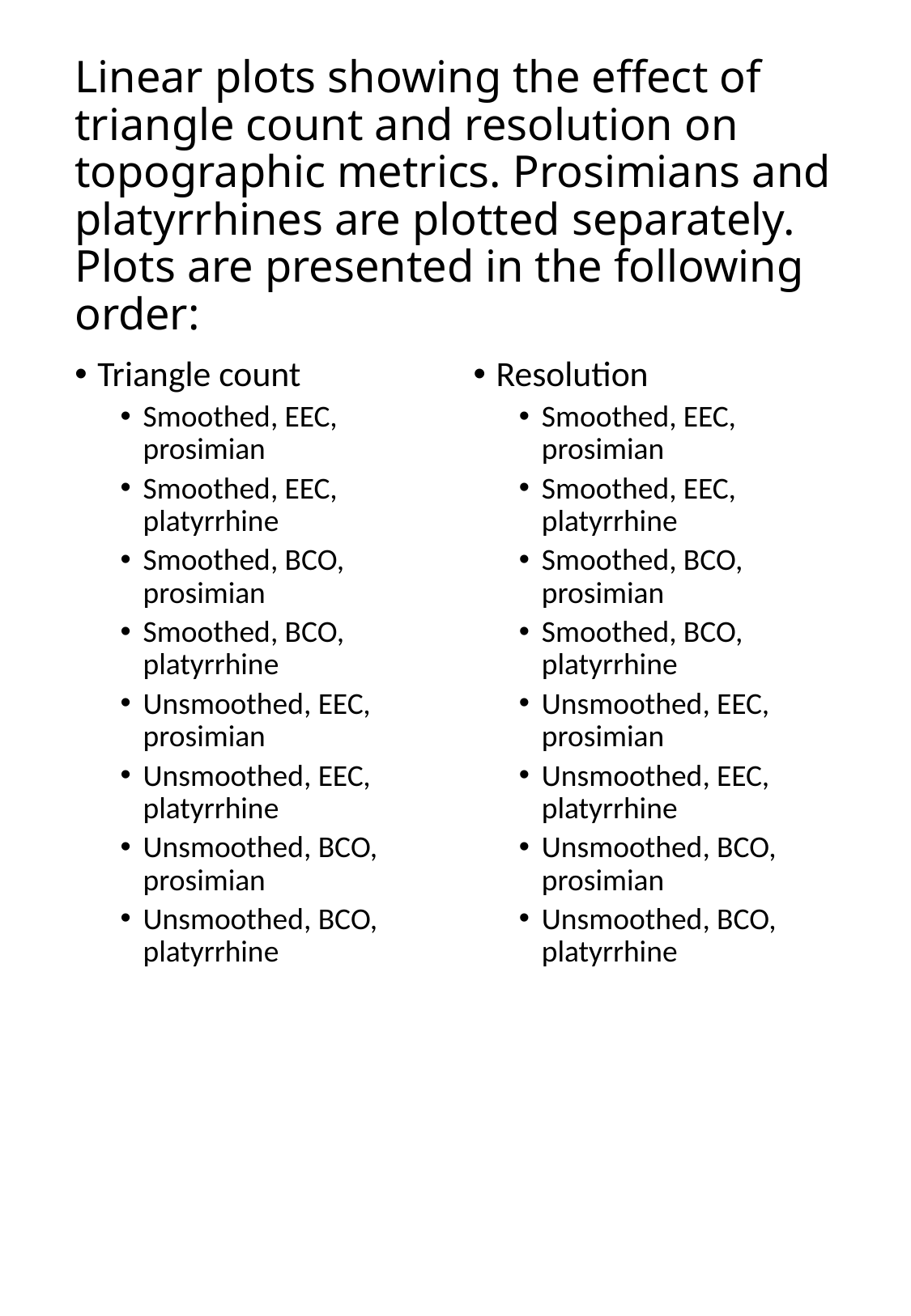

# Linear plots showing the effect of triangle count and resolution on topographic metrics. Prosimians and platyrrhines are plotted separately. Plots are presented in the following order:
Triangle count
Smoothed, EEC, prosimian
Smoothed, EEC, platyrrhine
Smoothed, BCO, prosimian
Smoothed, BCO, platyrrhine
Unsmoothed, EEC, prosimian
Unsmoothed, EEC, platyrrhine
Unsmoothed, BCO, prosimian
Unsmoothed, BCO, platyrrhine
Resolution
Smoothed, EEC, prosimian
Smoothed, EEC, platyrrhine
Smoothed, BCO, prosimian
Smoothed, BCO, platyrrhine
Unsmoothed, EEC, prosimian
Unsmoothed, EEC, platyrrhine
Unsmoothed, BCO, prosimian
Unsmoothed, BCO, platyrrhine

## Slide 2
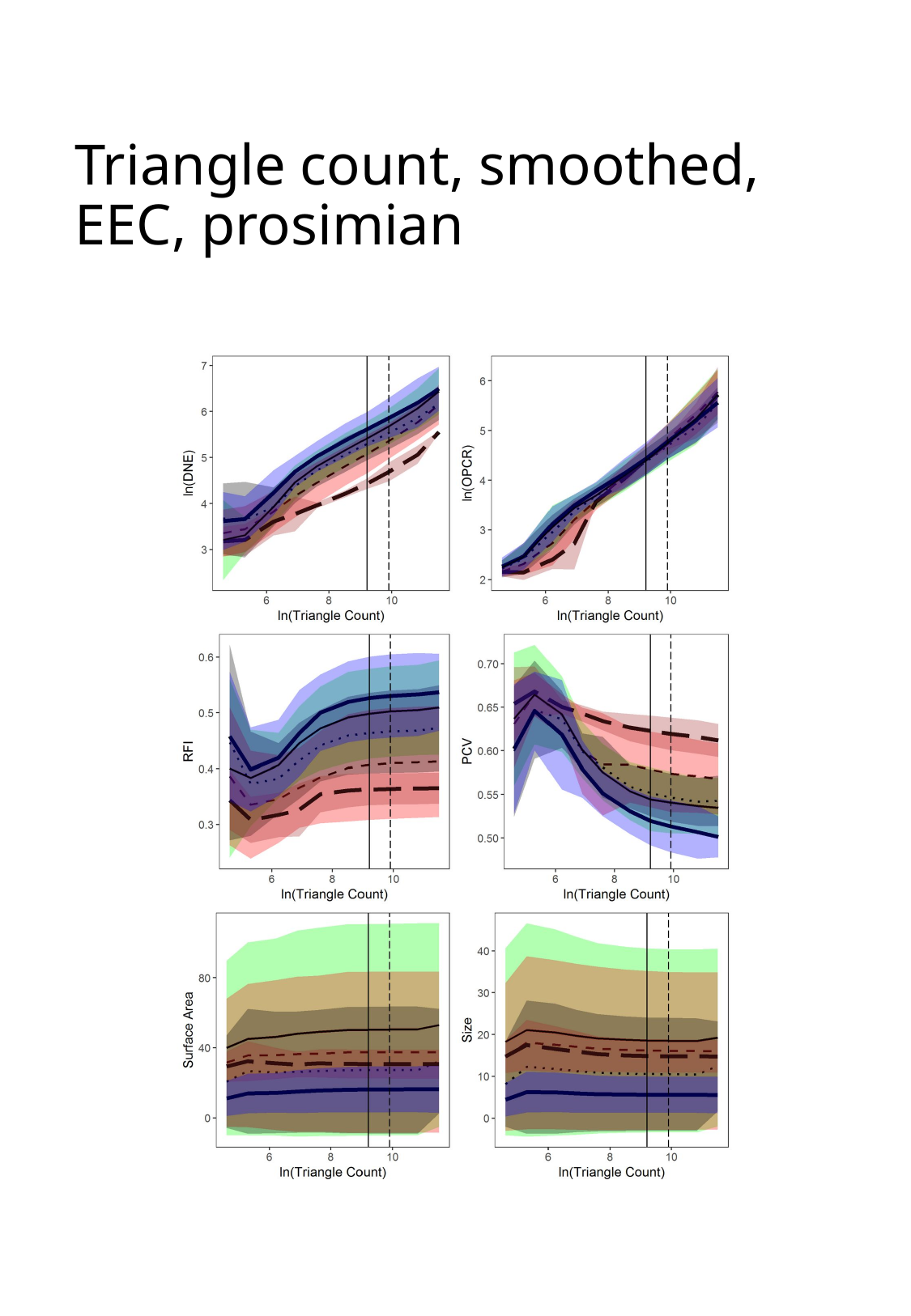

# Triangle count, smoothed, EEC, prosimian

## Slide 3
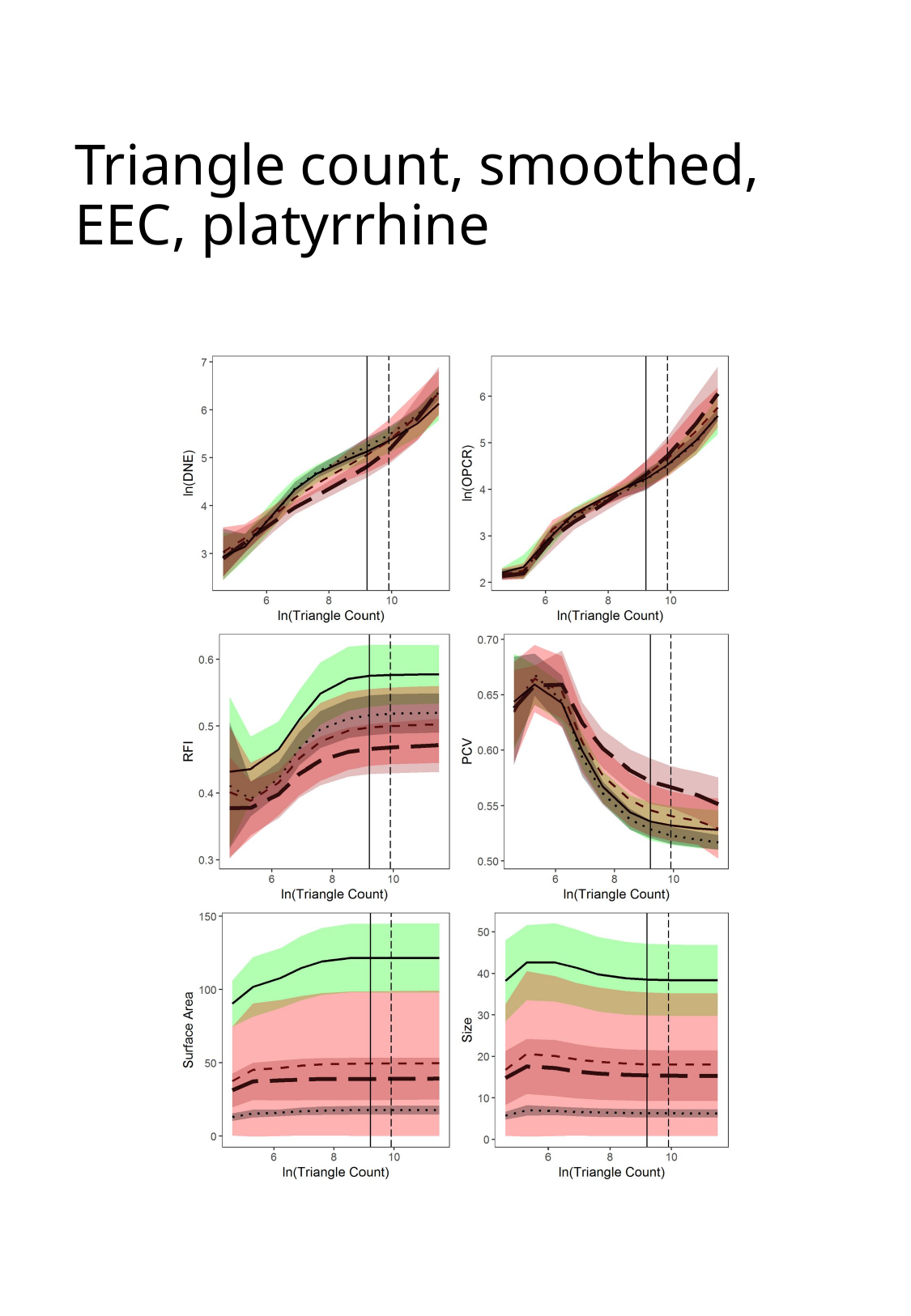

# Triangle count, smoothed, EEC, platyrrhine

## Slide 4
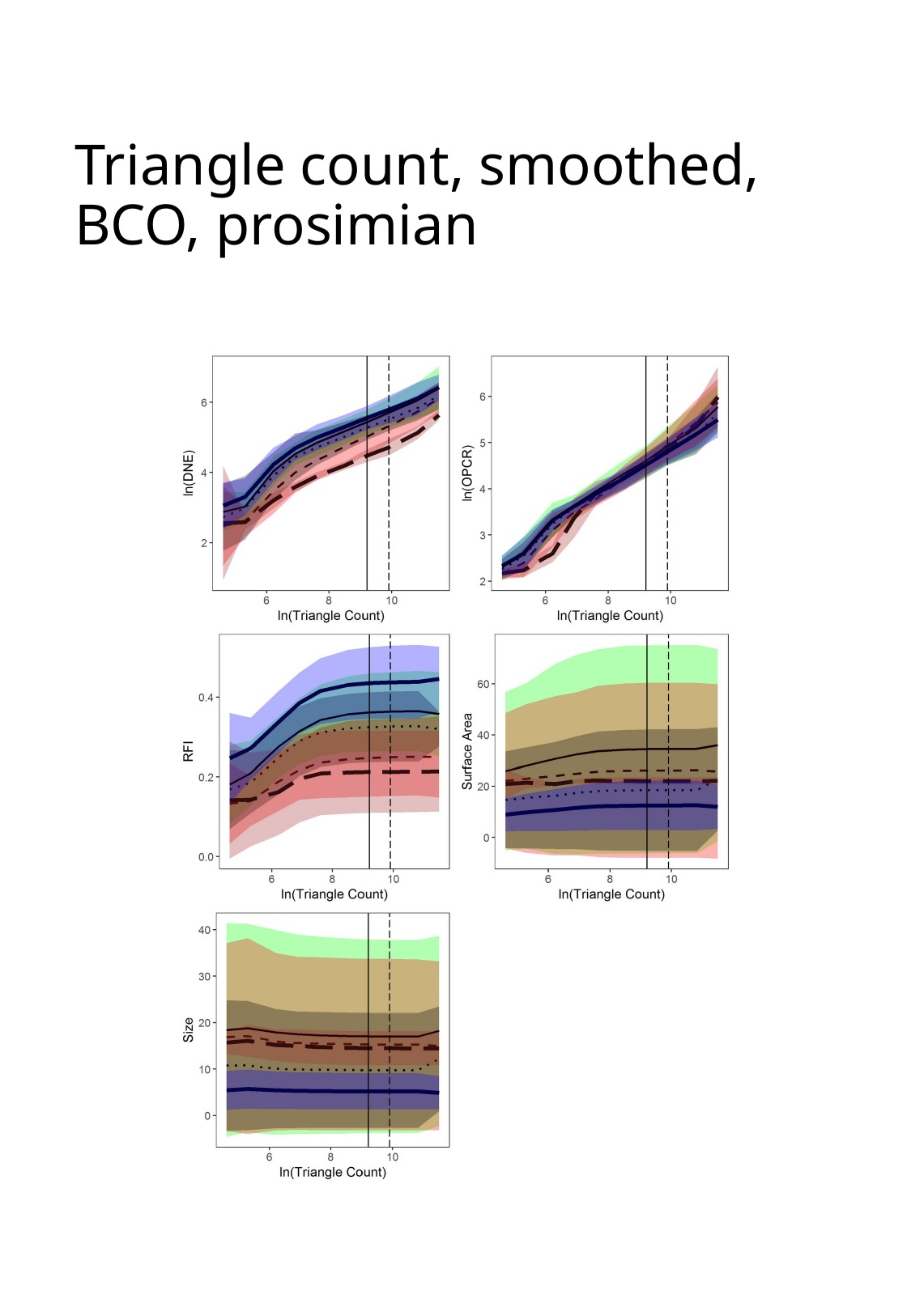

# Triangle count, smoothed, BCO, prosimian

## Slide 5
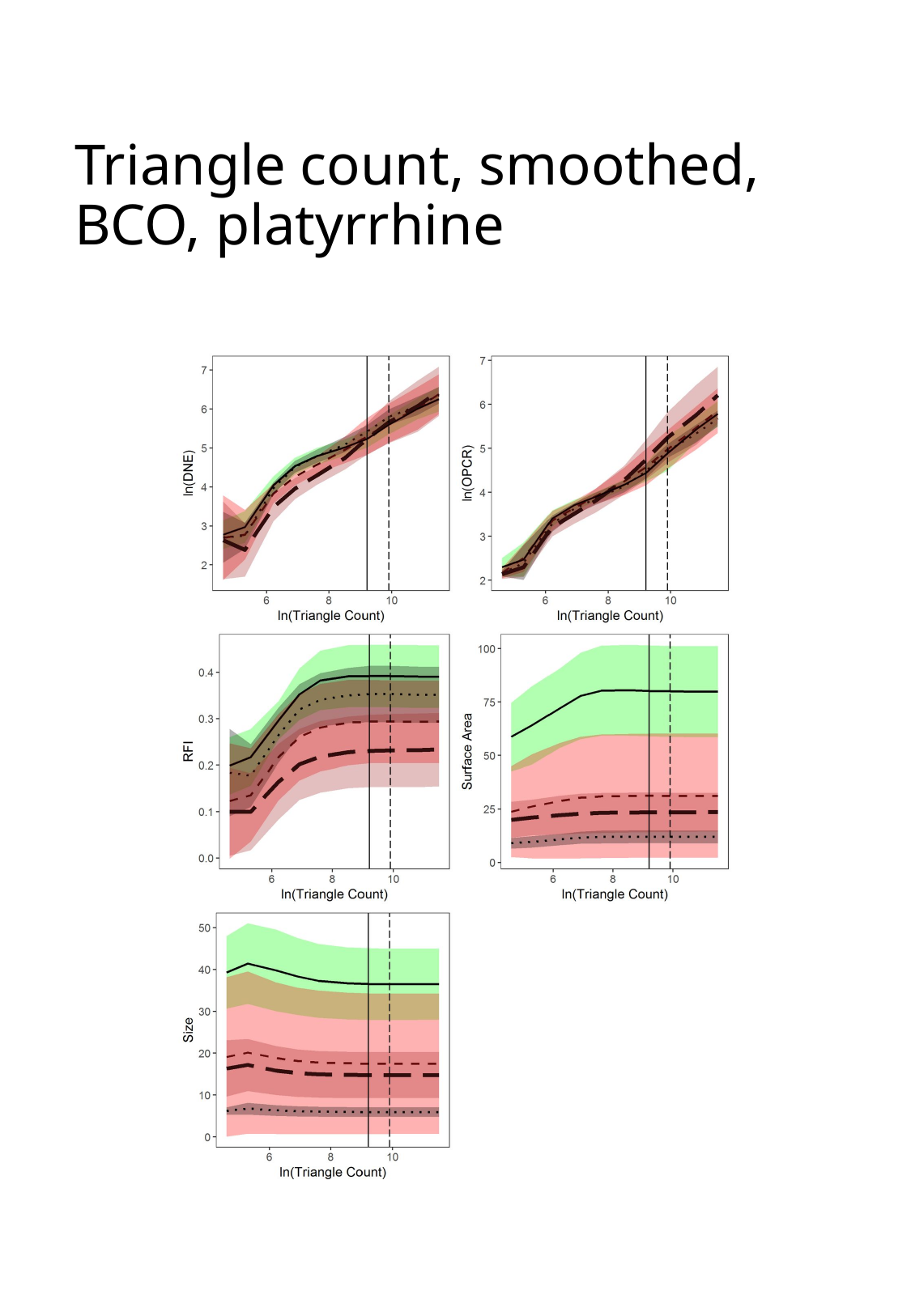

# Triangle count, smoothed, BCO, platyrrhine

## Slide 6
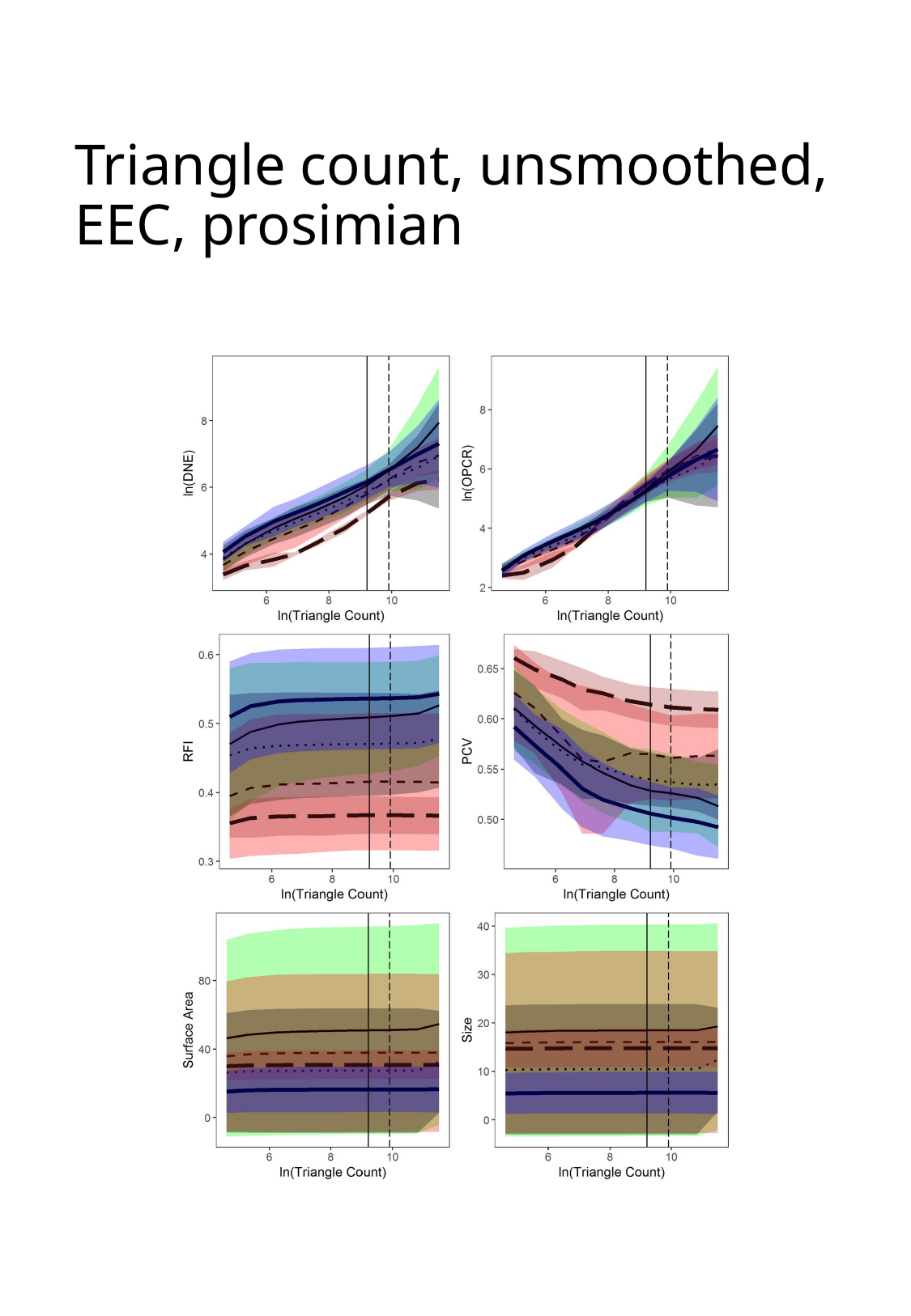

# Triangle count, unsmoothed, EEC, prosimian

## Slide 7
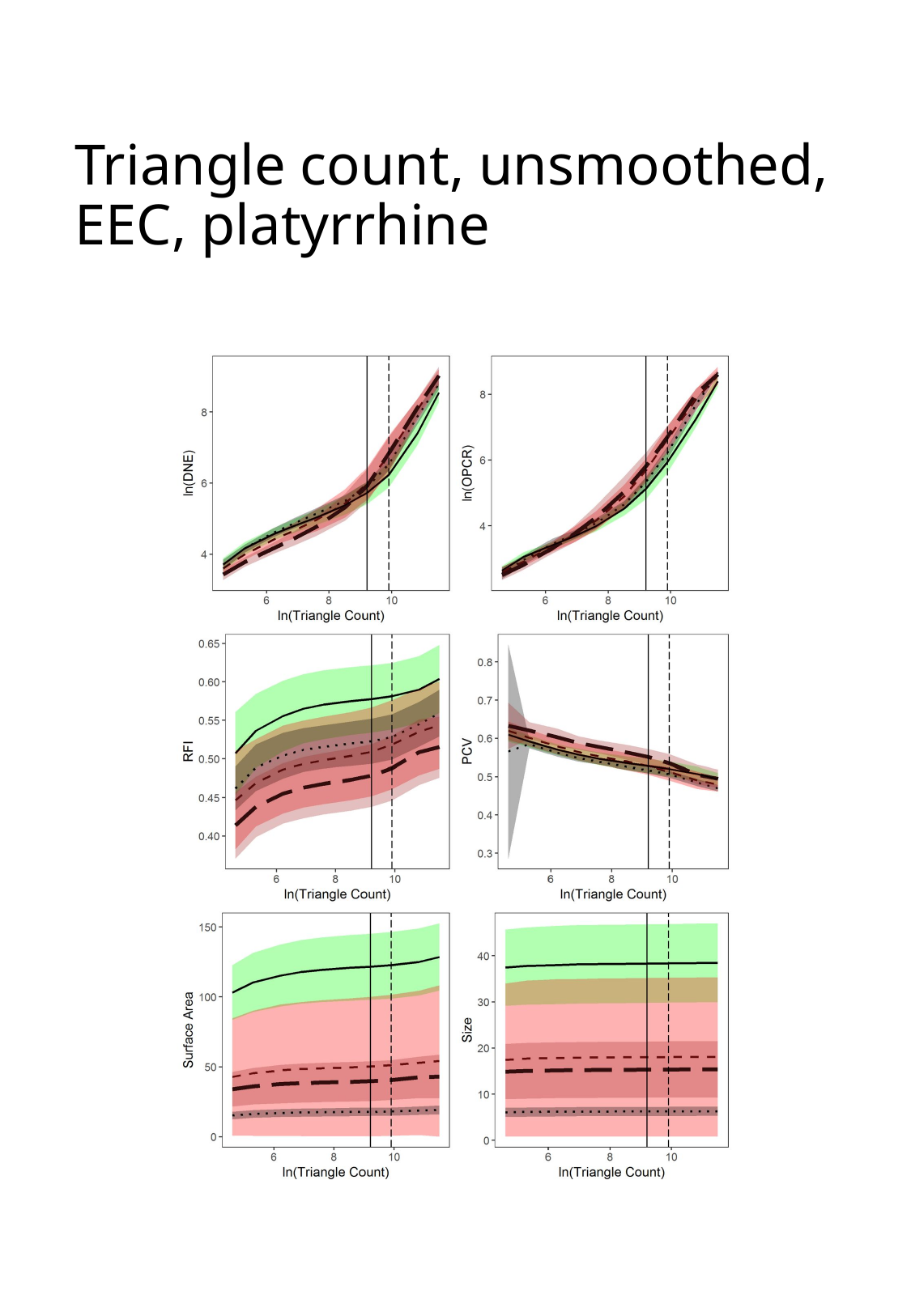

# Triangle count, unsmoothed, EEC, platyrrhine

## Slide 8
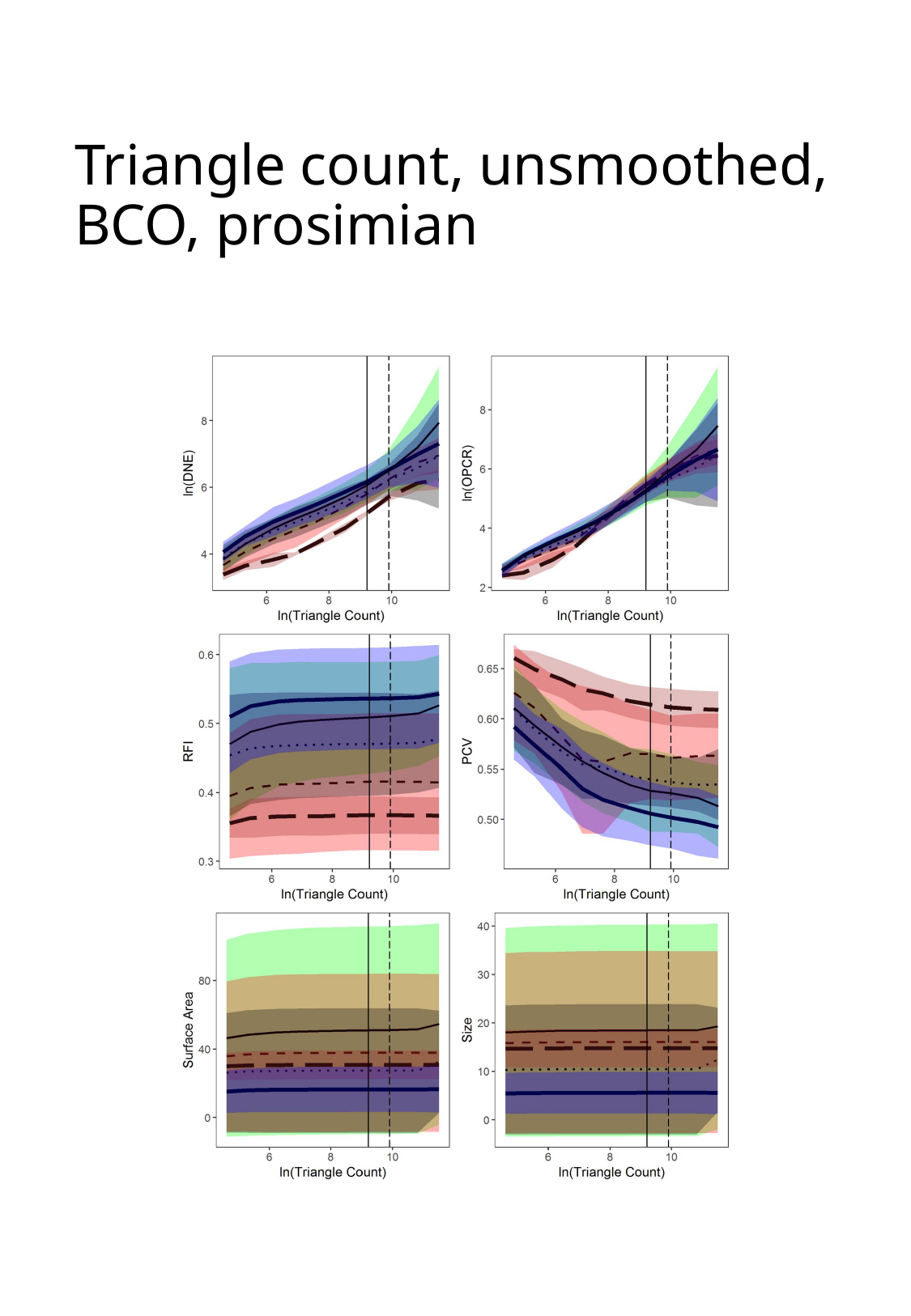

# Triangle count, unsmoothed, BCO, prosimian

## Slide 9
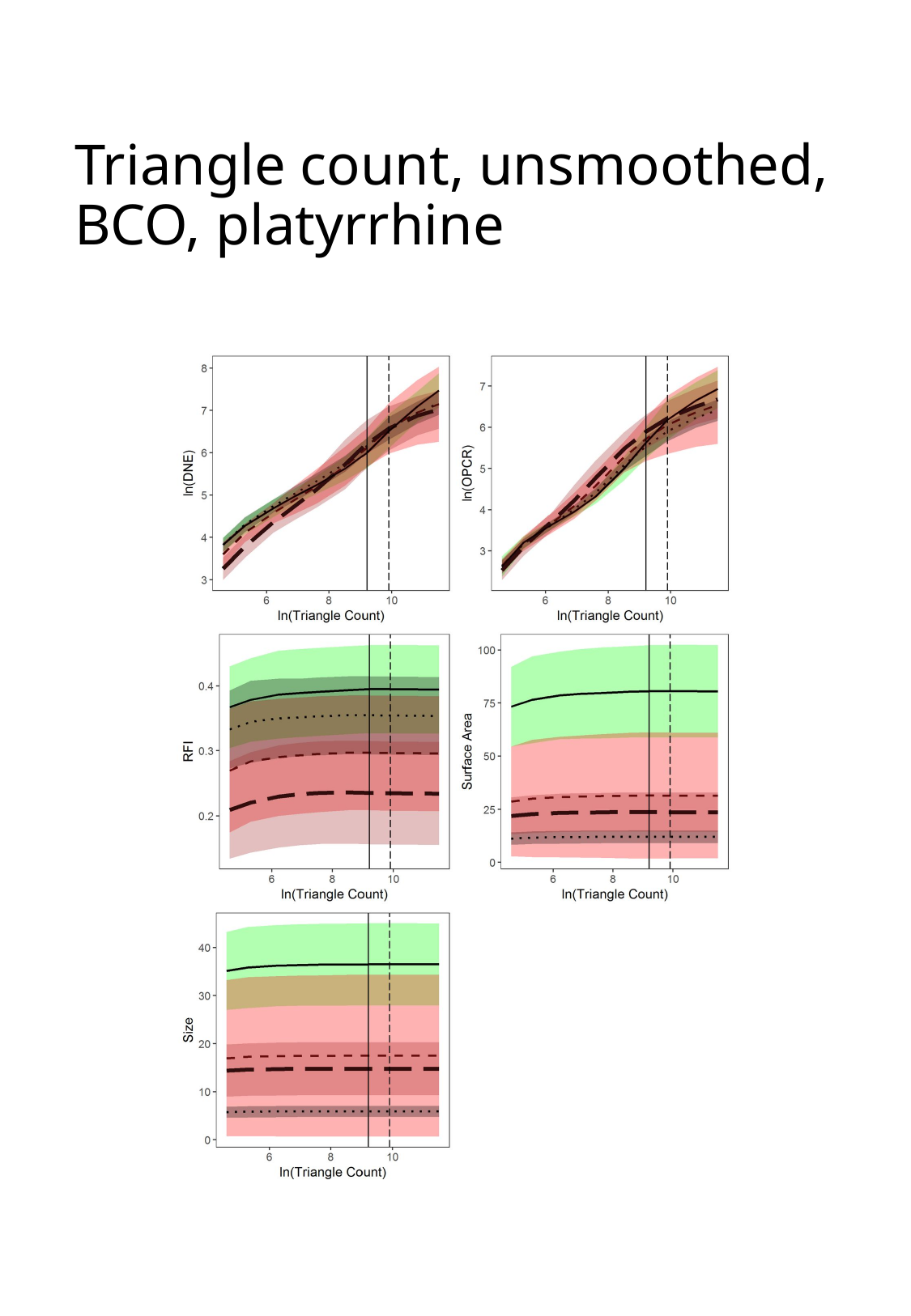

# Triangle count, unsmoothed, BCO, platyrrhine

## Slide 10
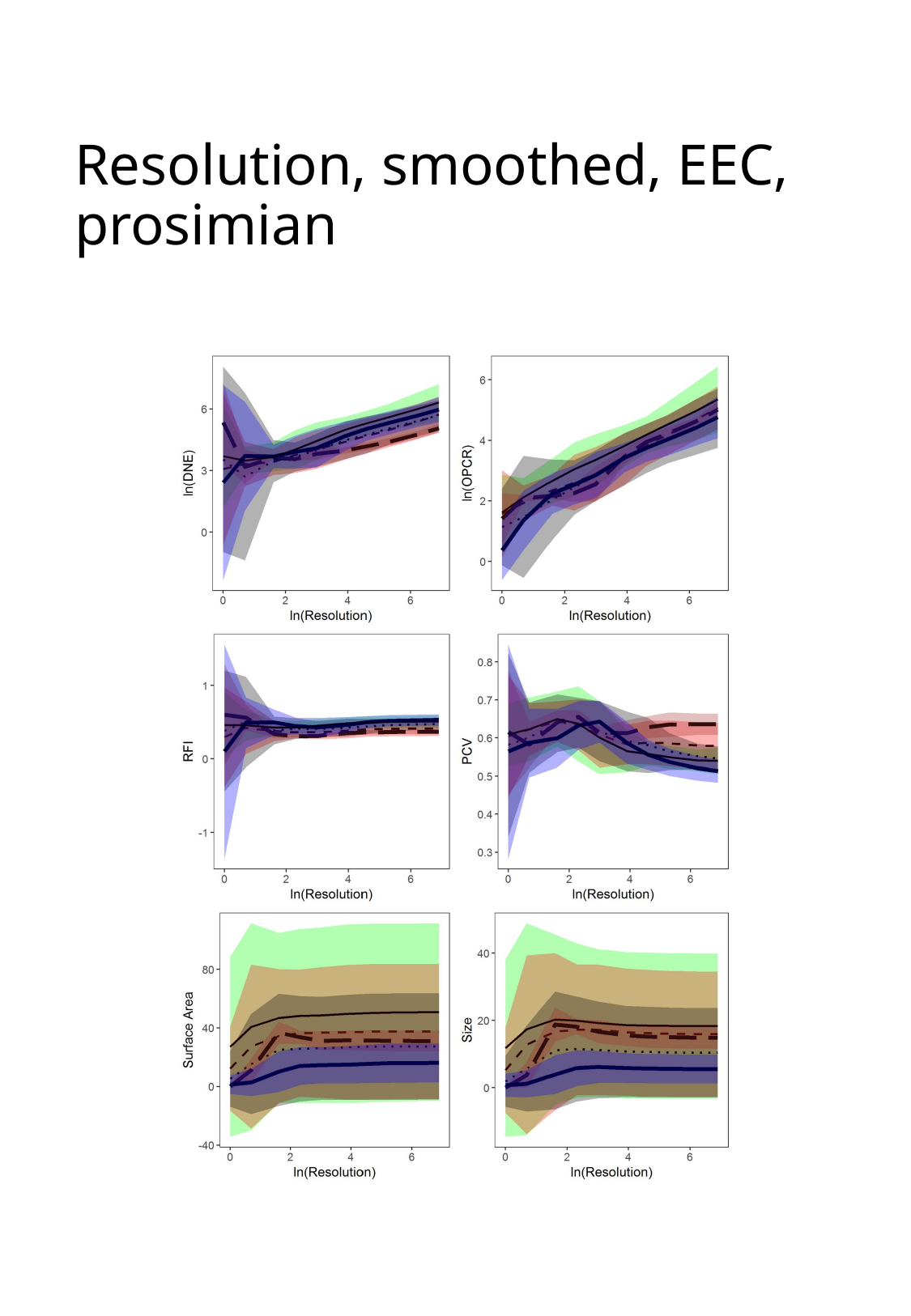

# Resolution, smoothed, EEC, prosimian

## Slide 11
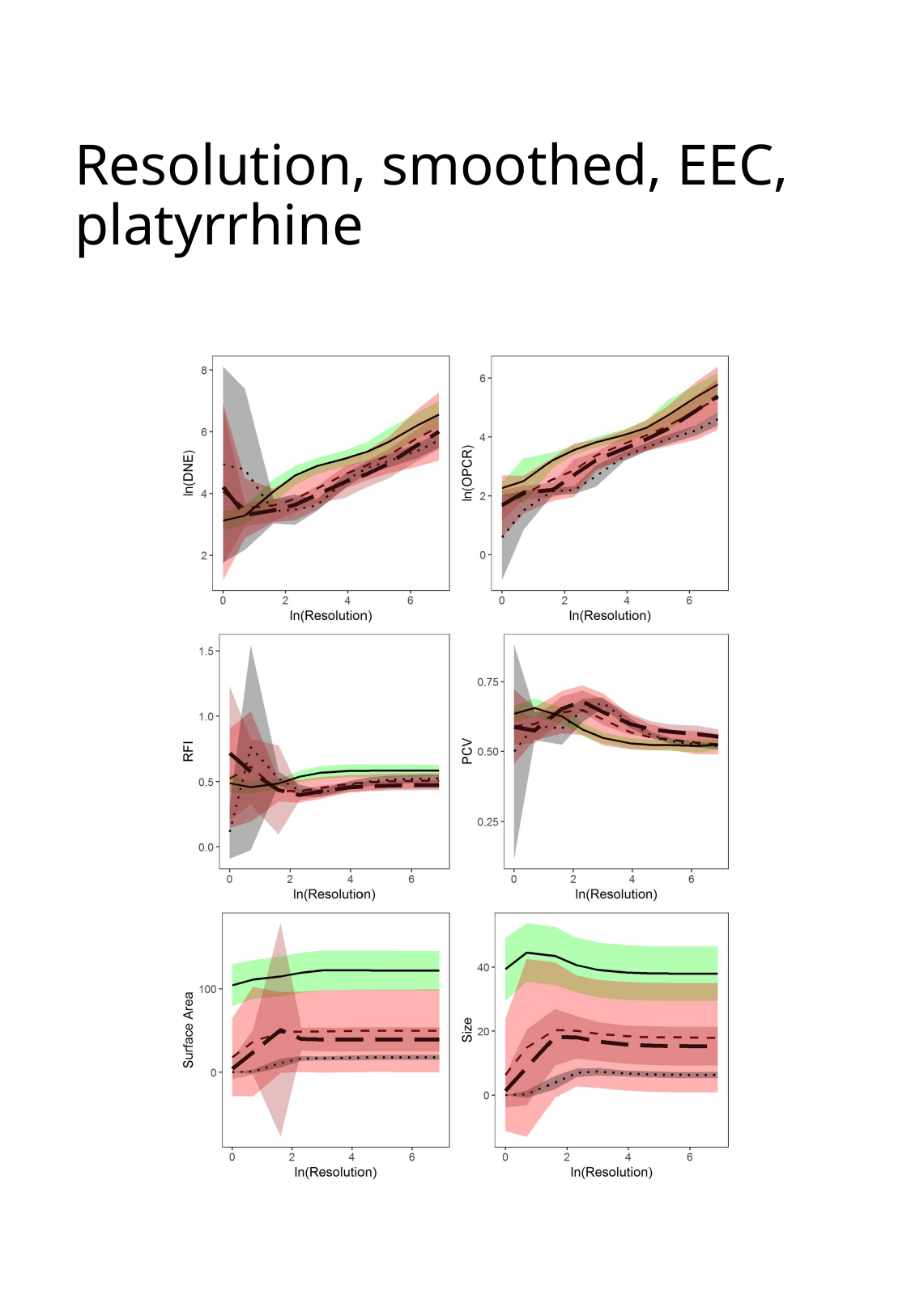

# Resolution, smoothed, EEC, platyrrhine

## Slide 12
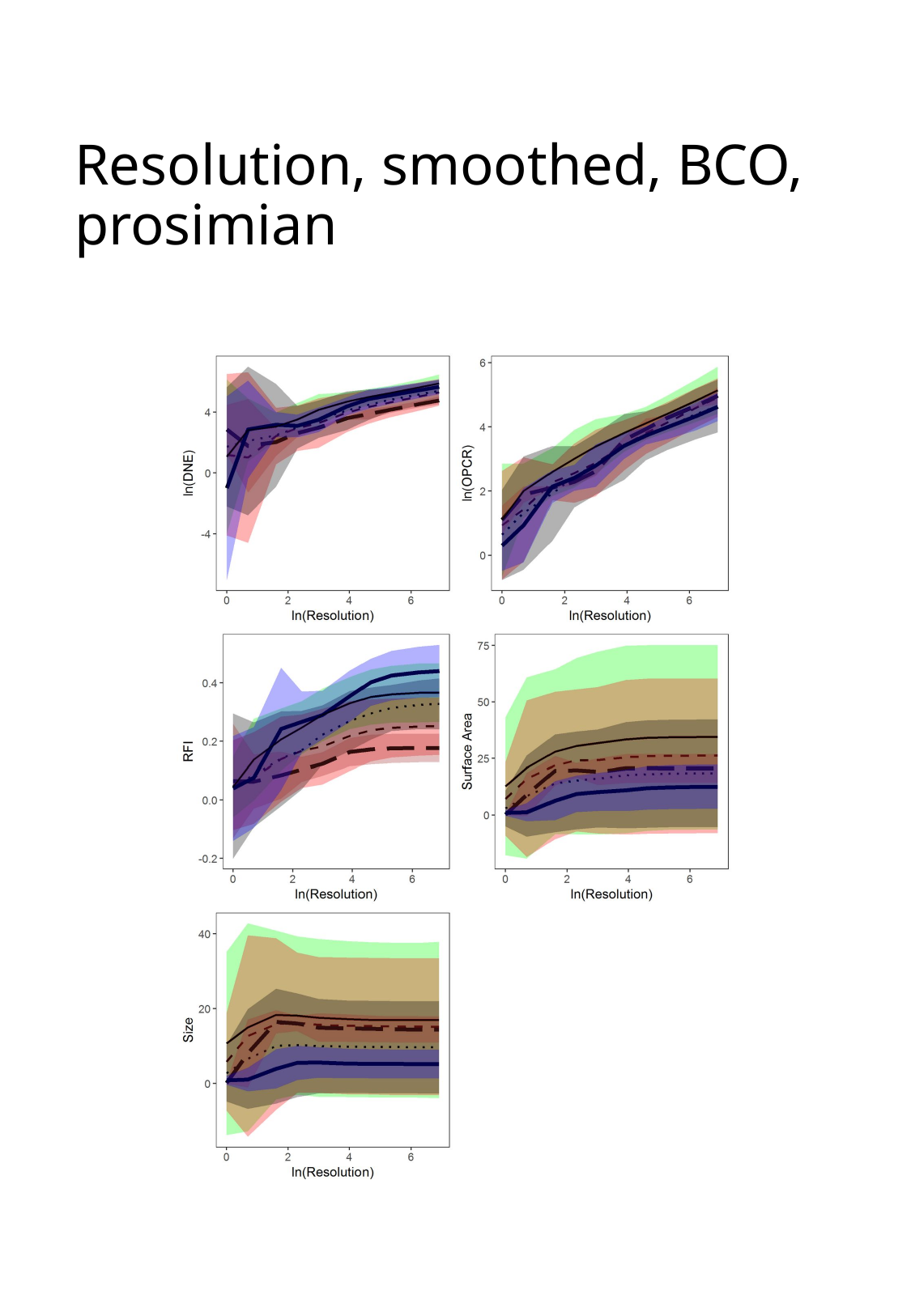

# Resolution, smoothed, BCO, prosimian

## Slide 13
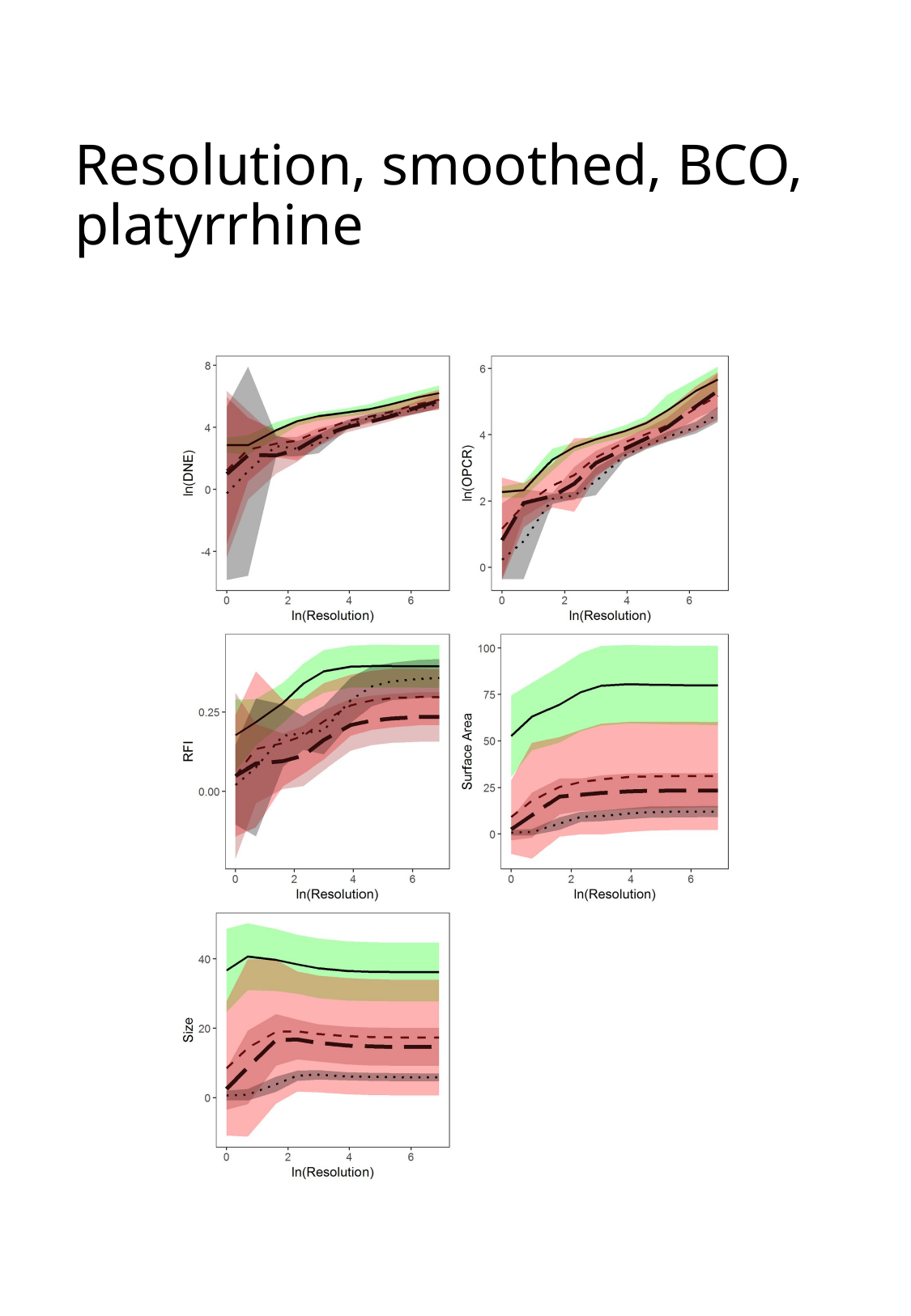

# Resolution, smoothed, BCO, platyrrhine

## Slide 14
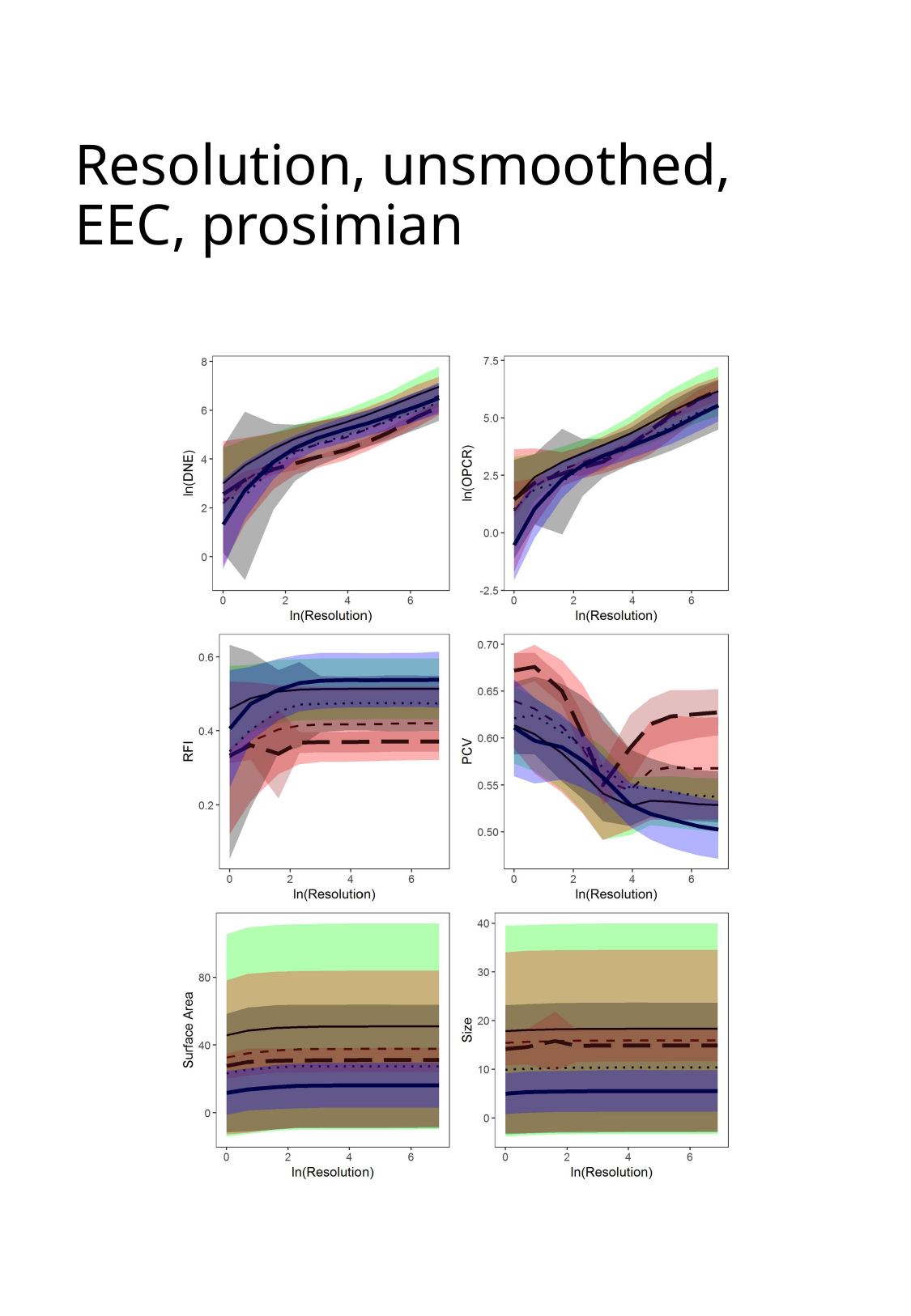

# Resolution, unsmoothed, EEC, prosimian

## Slide 15
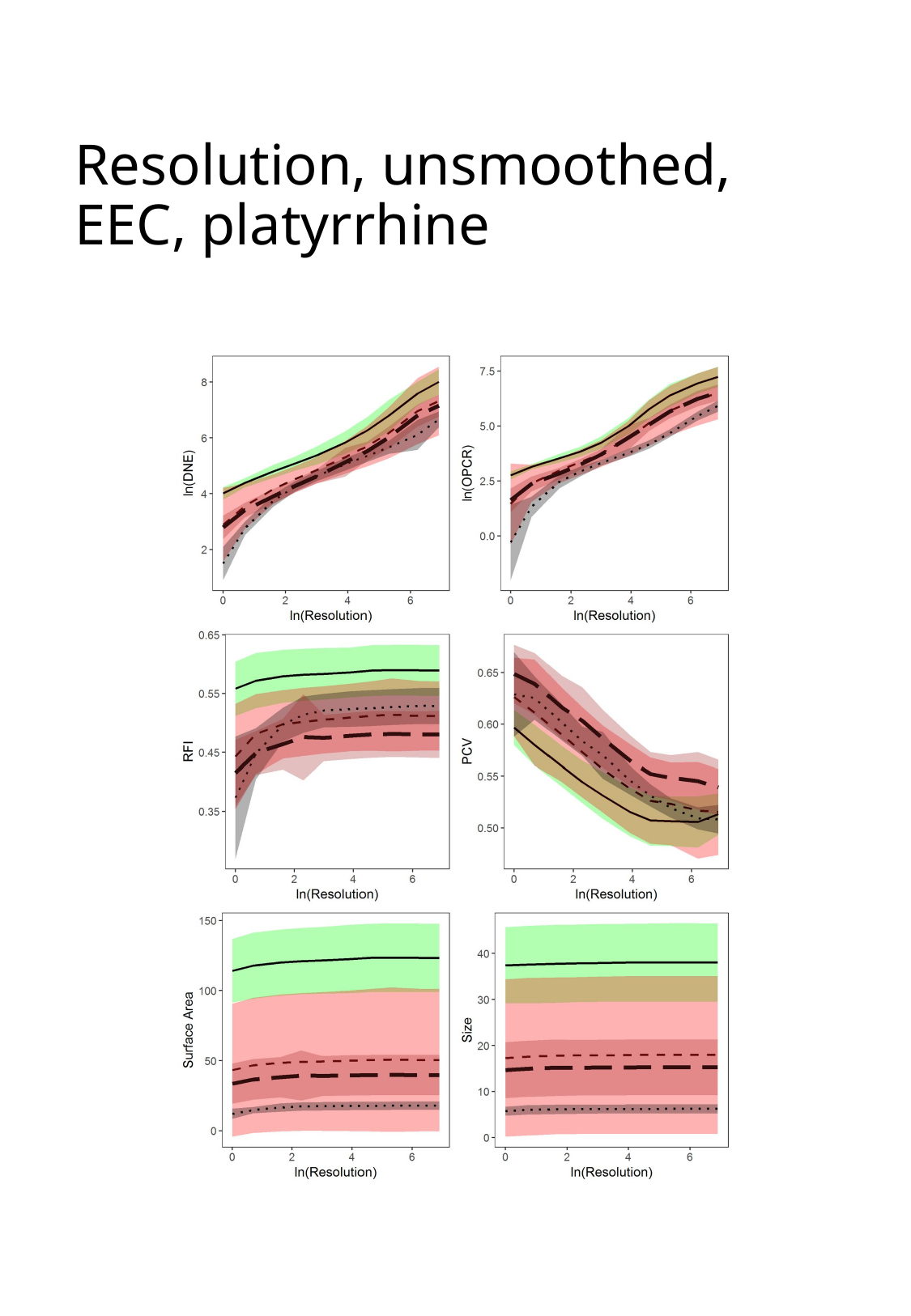

# Resolution, unsmoothed, EEC, platyrrhine

## Slide 16
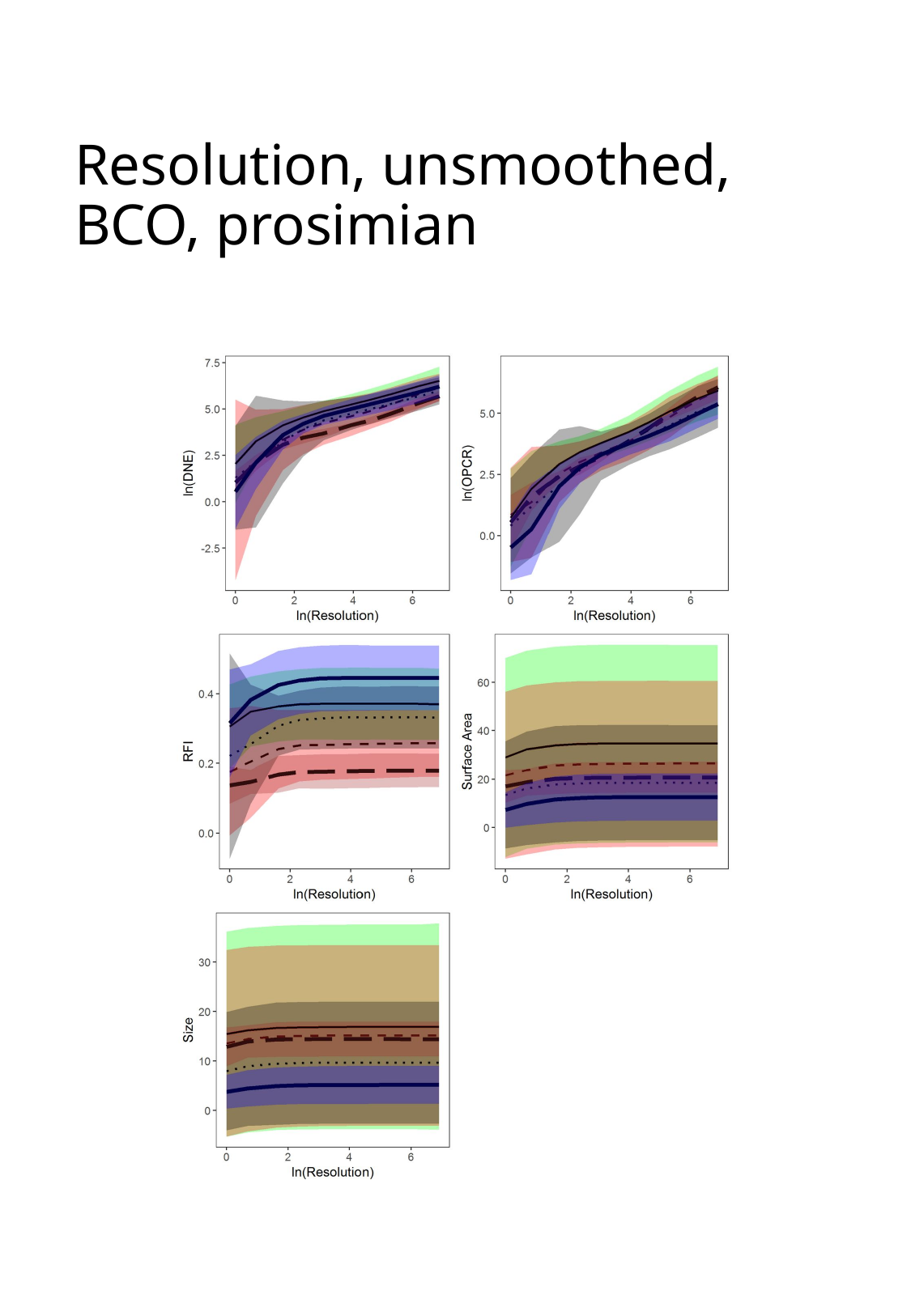

# Resolution, unsmoothed, BCO, prosimian

## Slide 17
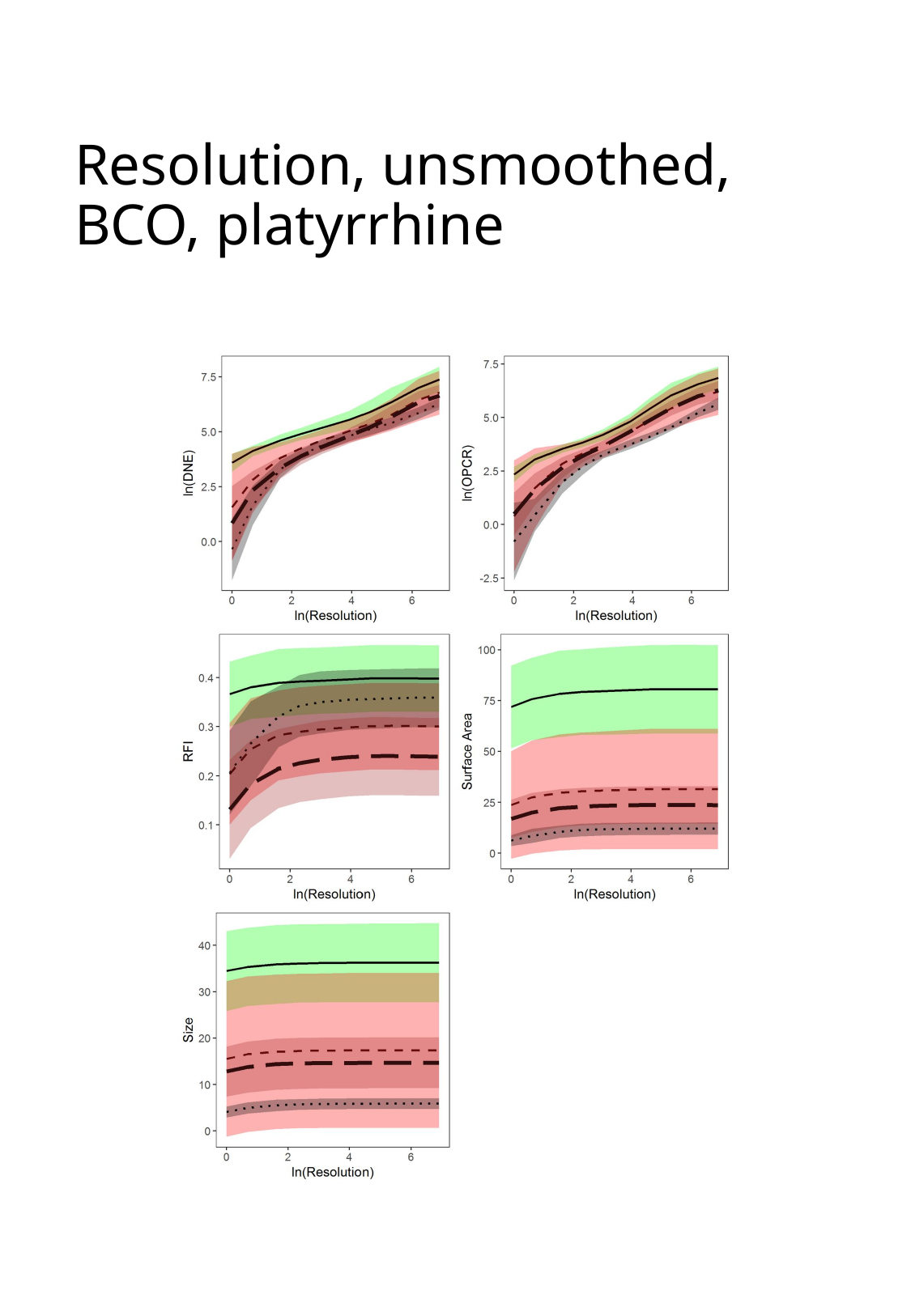

# Resolution, unsmoothed, BCO, platyrrhine
