# Supplementary material for: Effects of cropping, smoothing, triangle count, and mesh resolution on 6 dental topographic metrics
Source: PLoS One. 2019 May 6;14(5):e0216229. doi: 10.1371/journal.pone.0216229 (PMC6502444; doi:10.1371/journal.pone.0216229)
Supplement: S4 Fig — . Boxplots for DNE and OPCR slopes and intercepts vs. diet. (PPTX) [file pone.0216229.s013.pptx]

## Slide 1
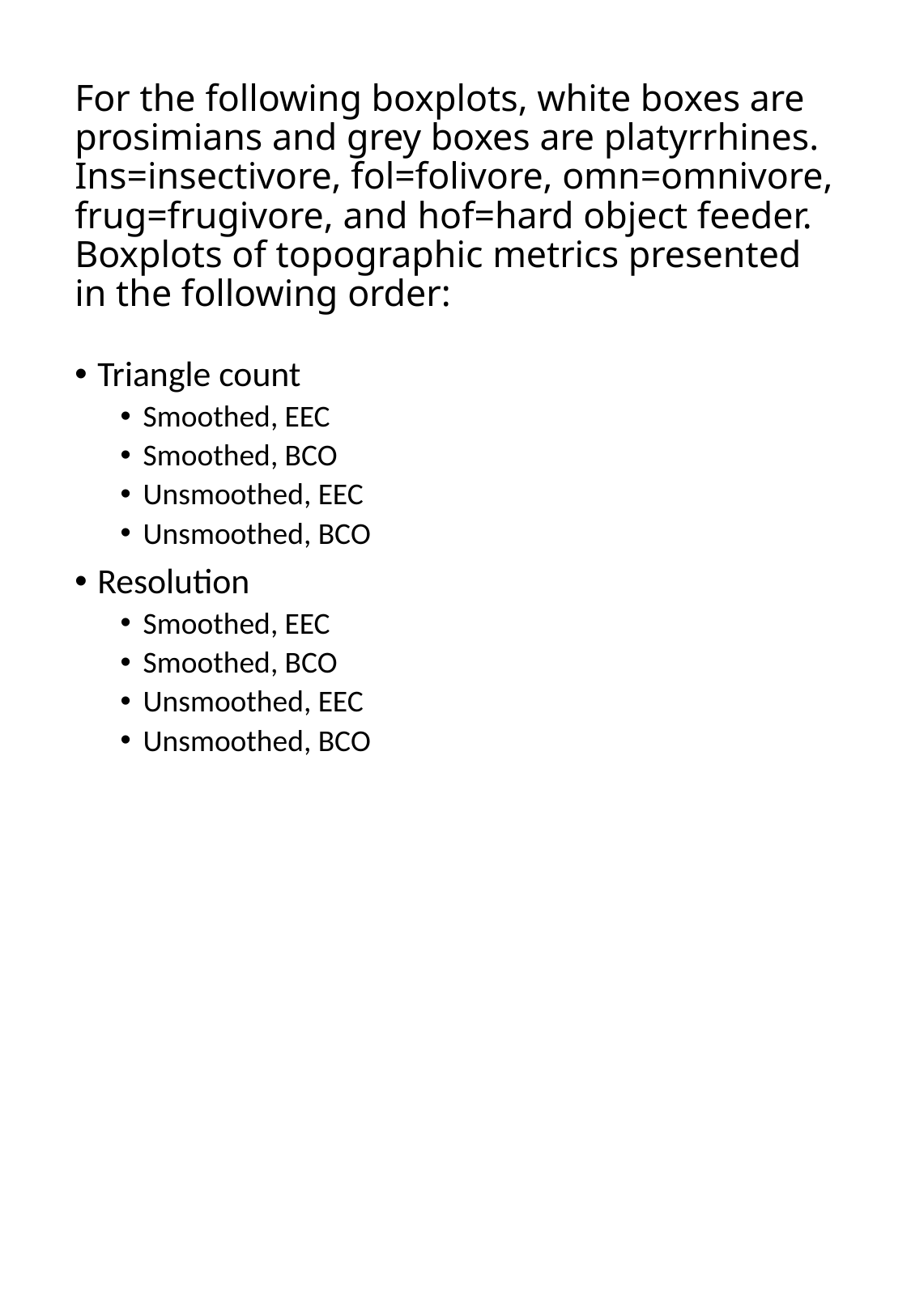

# For the following boxplots, white boxes are prosimians and grey boxes are platyrrhines. Ins=insectivore, fol=folivore, omn=omnivore, frug=frugivore, and hof=hard object feeder. Boxplots of topographic metrics presented in the following order:
Triangle count
Smoothed, EEC
Smoothed, BCO
Unsmoothed, EEC
Unsmoothed, BCO
Resolution
Smoothed, EEC
Smoothed, BCO
Unsmoothed, EEC
Unsmoothed, BCO

## Slide 2
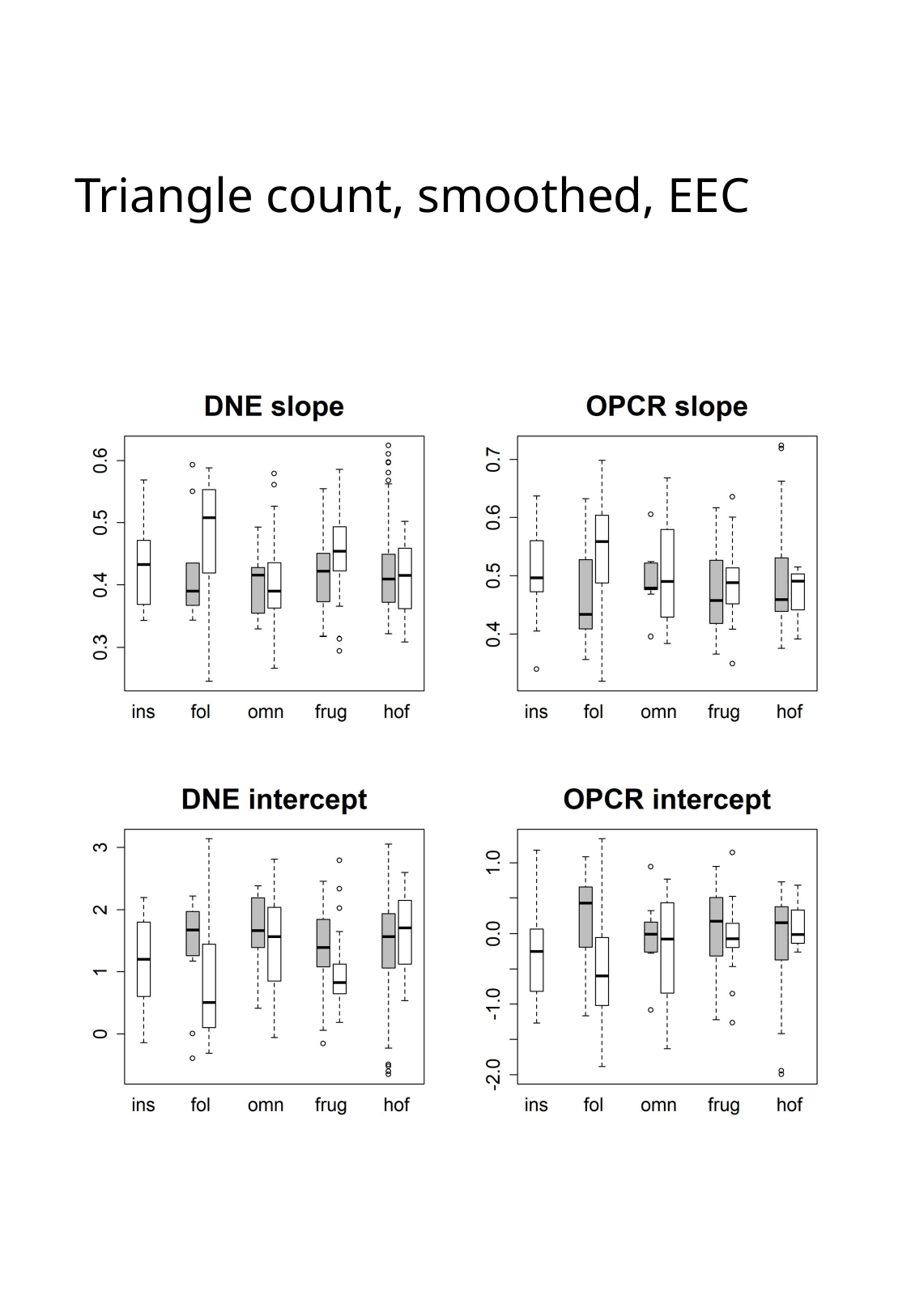

# Triangle count, smoothed, EEC

## Slide 3
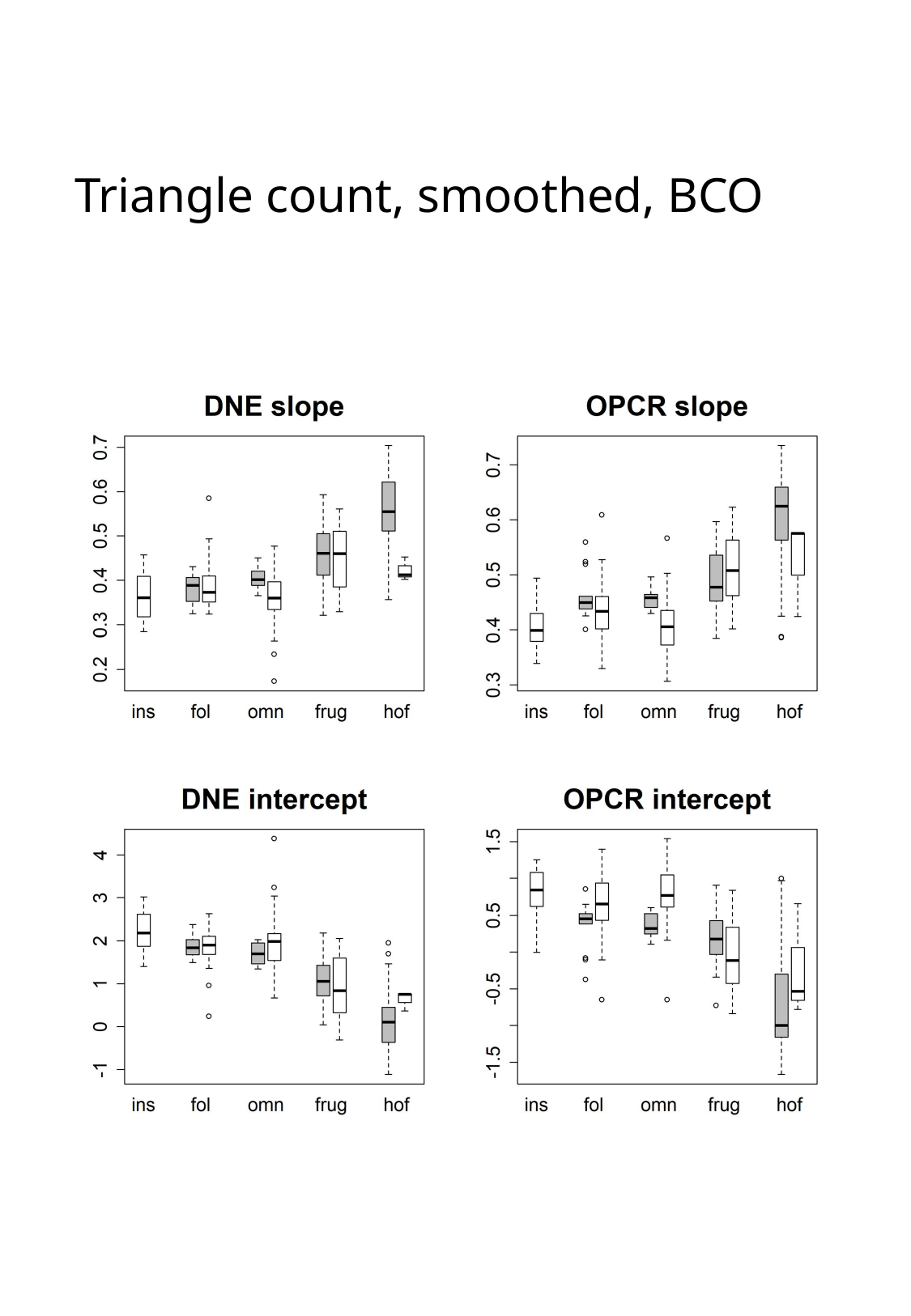

# Triangle count, smoothed, BCO

## Slide 4
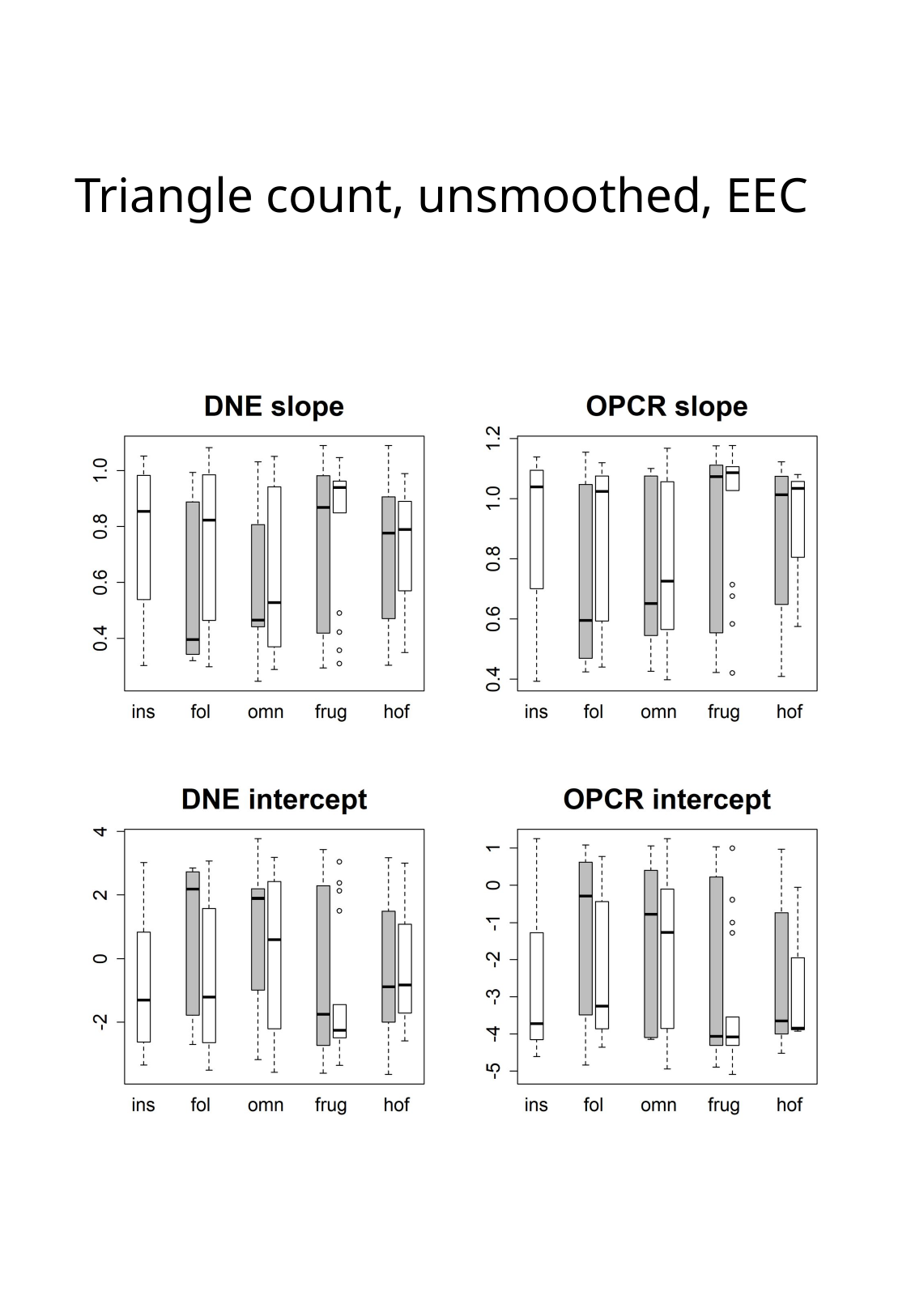

# Triangle count, unsmoothed, EEC

## Slide 5
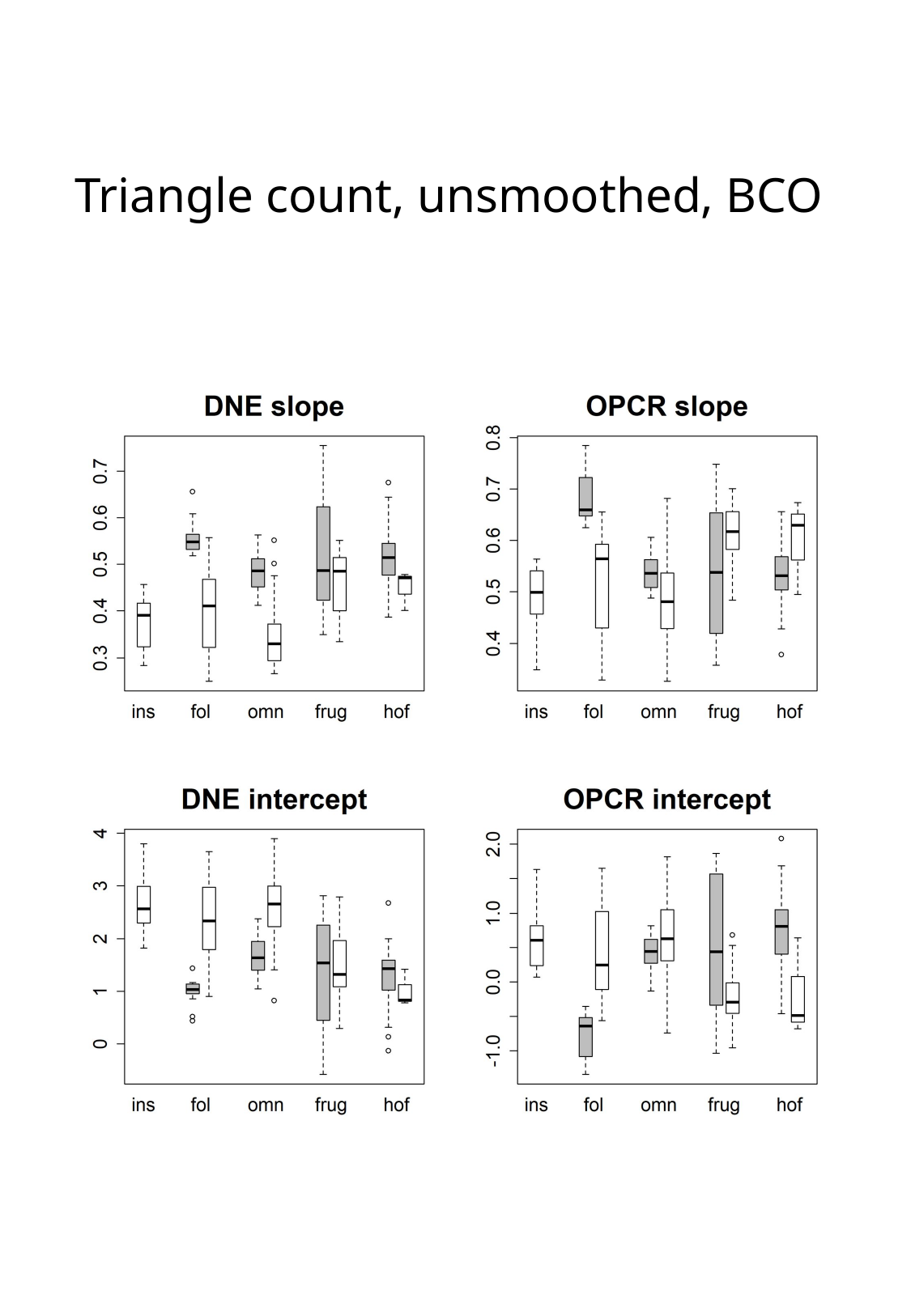

# Triangle count, unsmoothed, BCO

## Slide 6
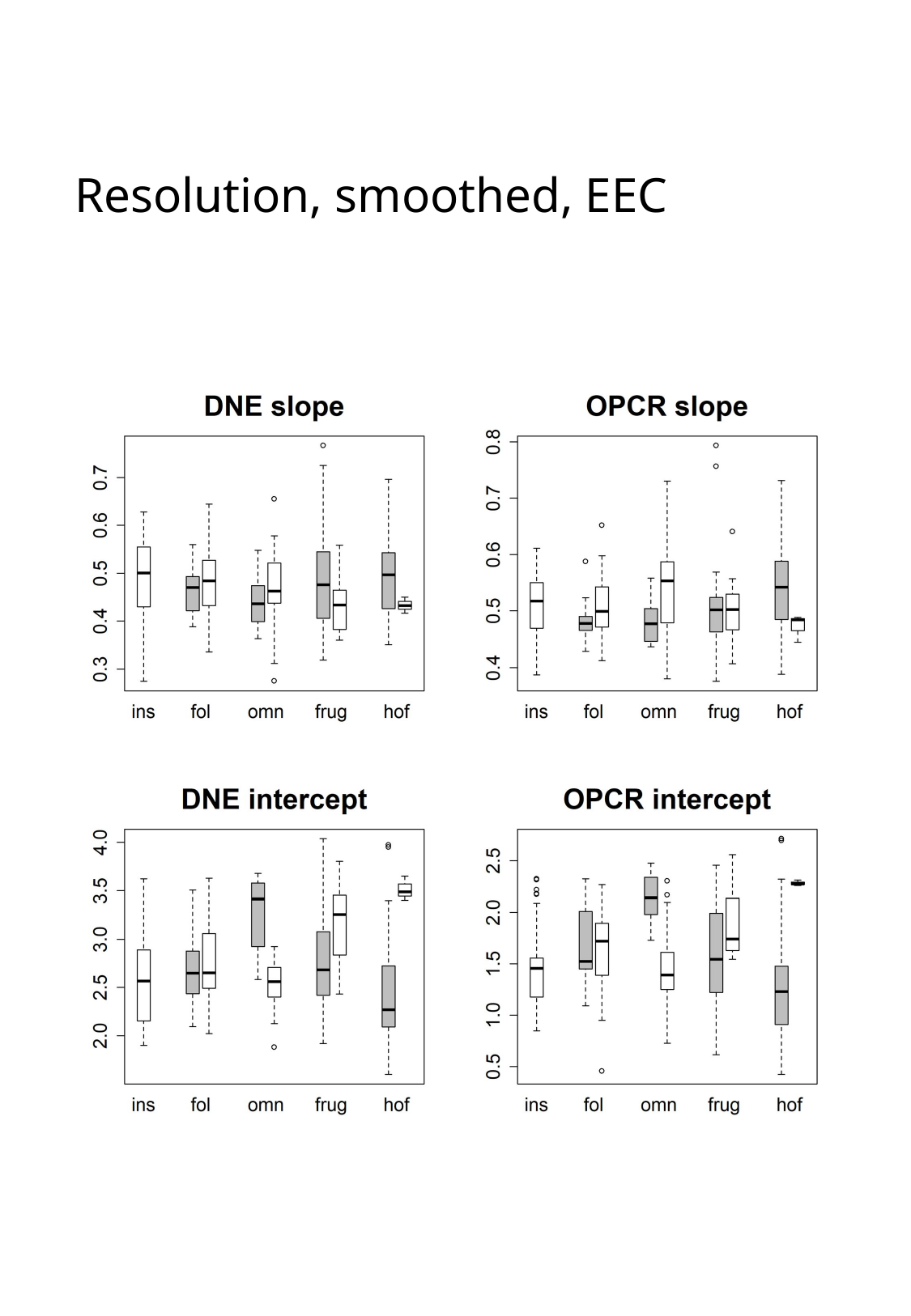

# Resolution, smoothed, EEC

## Slide 7
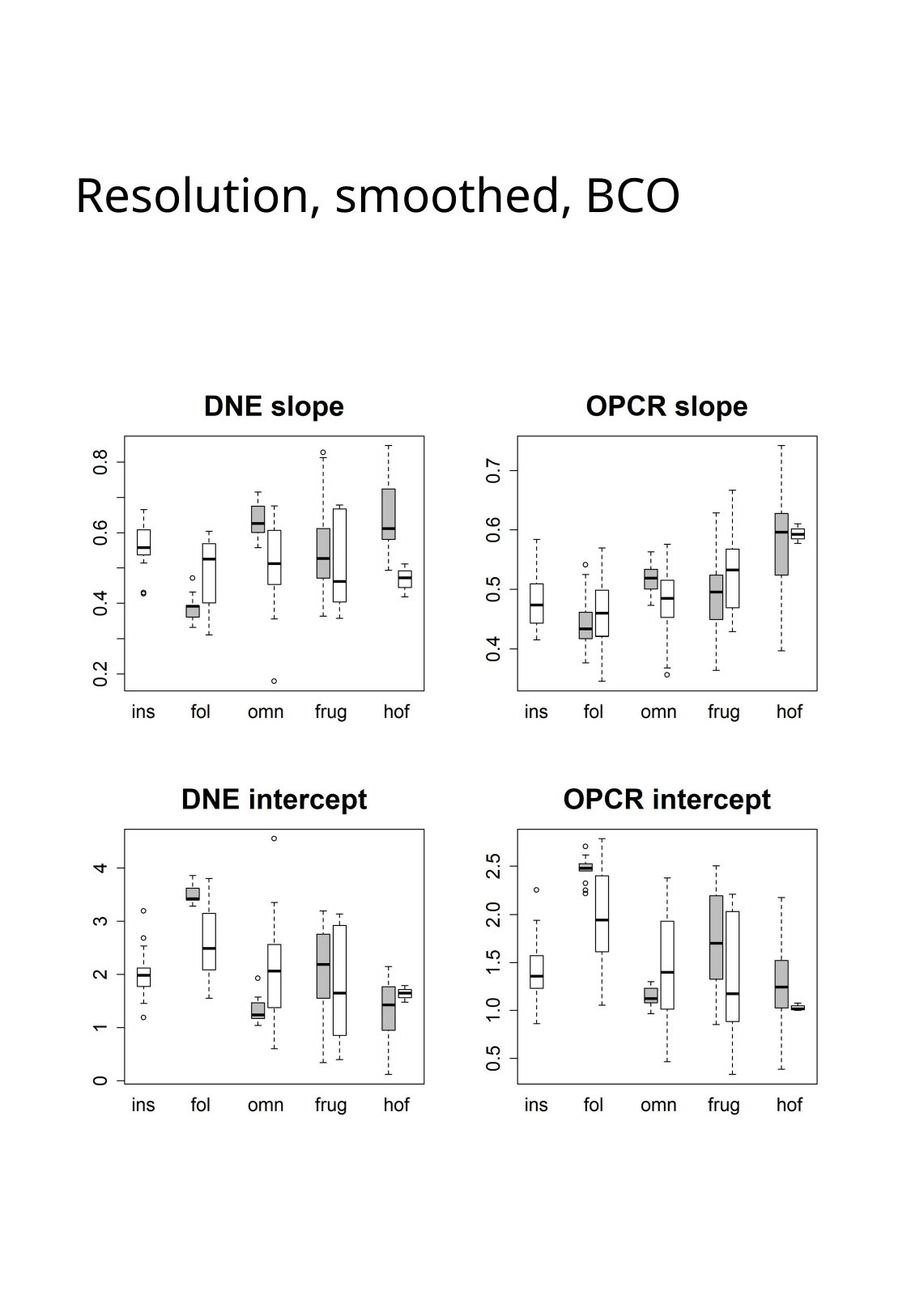

# Resolution, smoothed, BCO

## Slide 8
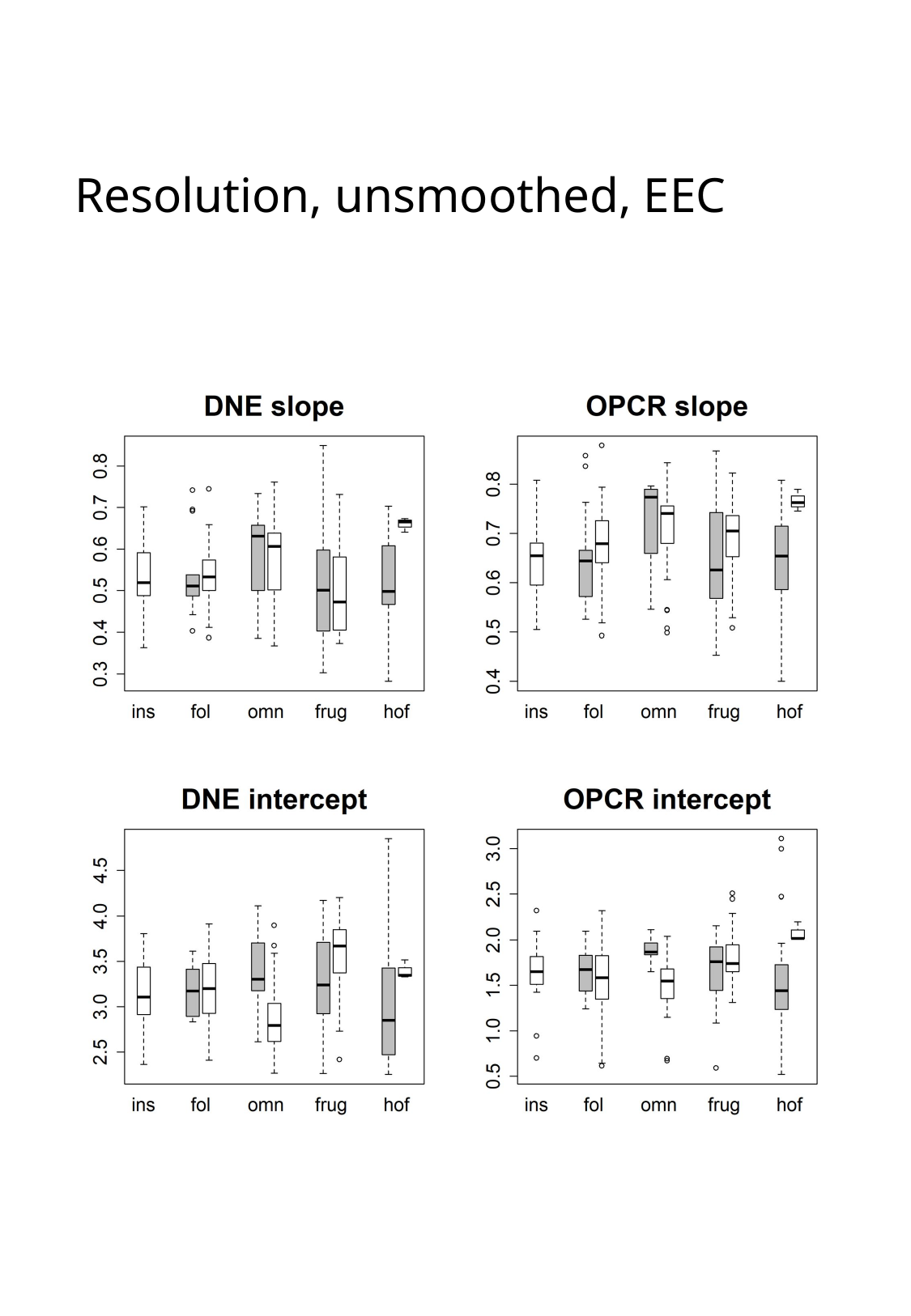

# Resolution, unsmoothed, EEC

## Slide 9
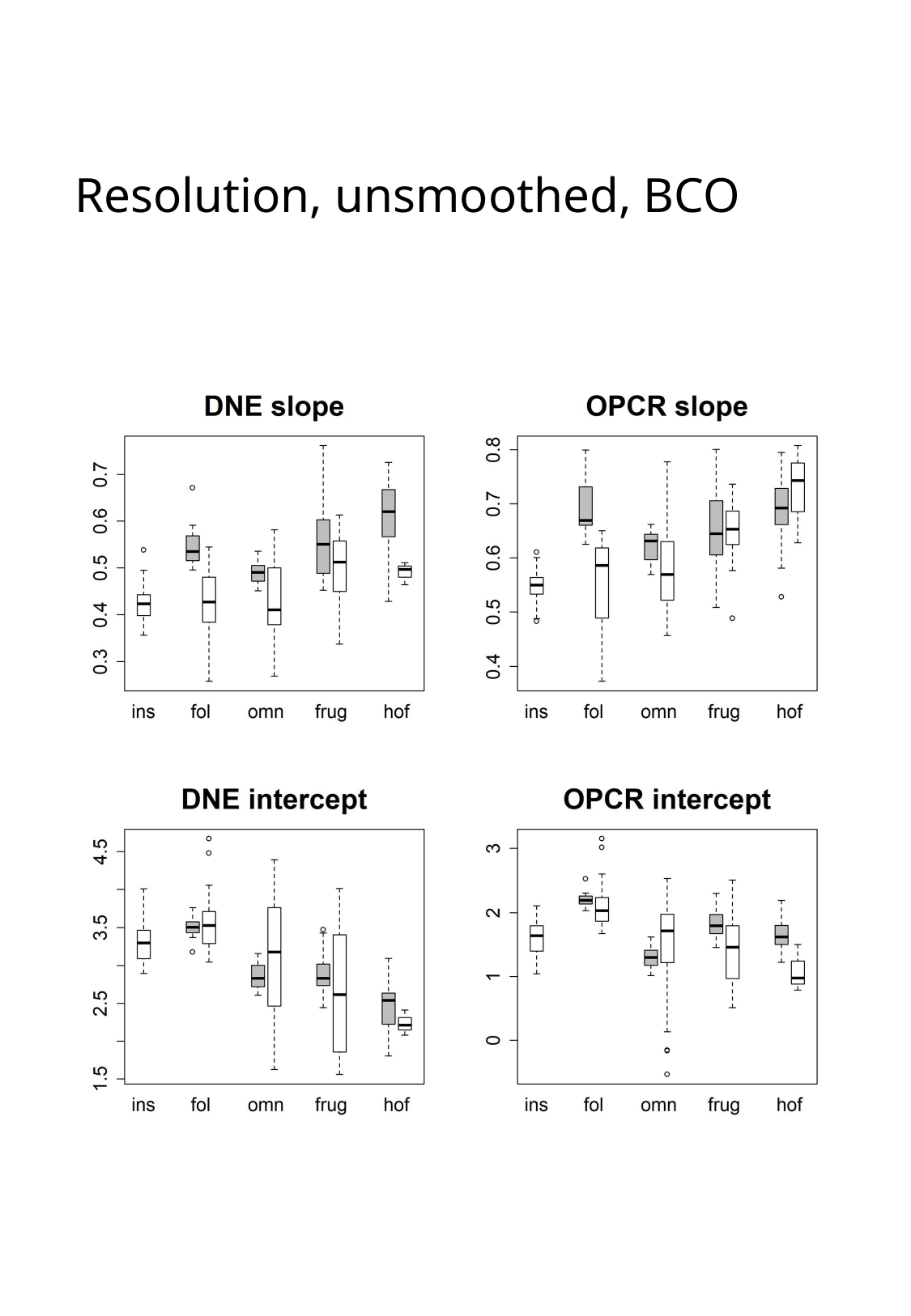

# Resolution, unsmoothed, BCO
