# Supplementary material for: Effects of cropping, smoothing, triangle count, and mesh resolution on 6 dental topographic metrics
Source: PLoS One. 2019 May 6;14(5):e0216229. doi: 10.1371/journal.pone.0216229 (PMC6502444; doi:10.1371/journal.pone.0216229)
Supplement: S5 Fig — Graphical representations of Tukey HSD results. (PPTX) [file pone.0216229.s014.pptx]

## Slide 1
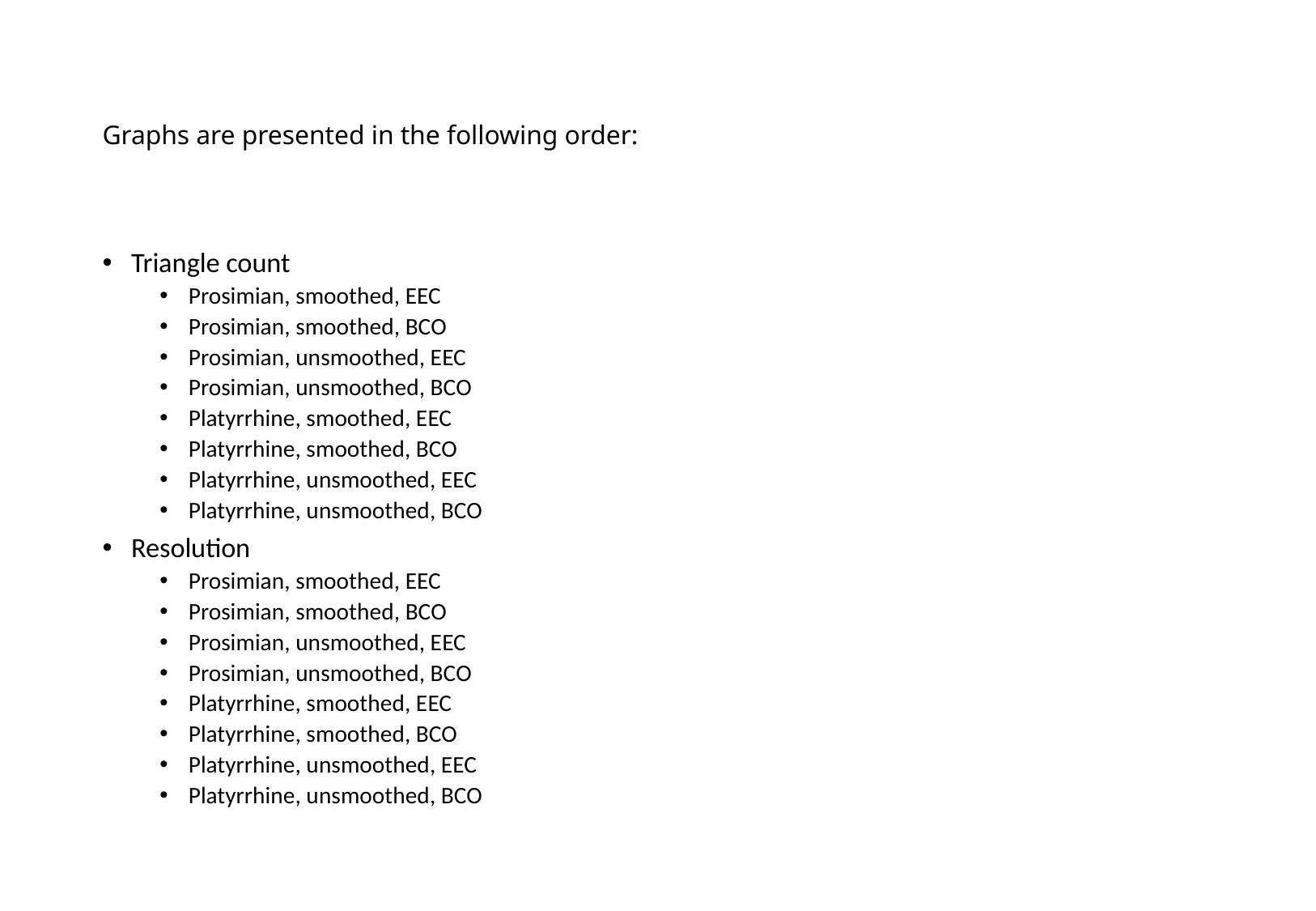

# Graphs are presented in the following order:
Triangle count
Prosimian, smoothed, EEC
Prosimian, smoothed, BCO
Prosimian, unsmoothed, EEC
Prosimian, unsmoothed, BCO
Platyrrhine, smoothed, EEC
Platyrrhine, smoothed, BCO
Platyrrhine, unsmoothed, EEC
Platyrrhine, unsmoothed, BCO
Resolution
Prosimian, smoothed, EEC
Prosimian, smoothed, BCO
Prosimian, unsmoothed, EEC
Prosimian, unsmoothed, BCO
Platyrrhine, smoothed, EEC
Platyrrhine, smoothed, BCO
Platyrrhine, unsmoothed, EEC
Platyrrhine, unsmoothed, BCO

## Slide 2
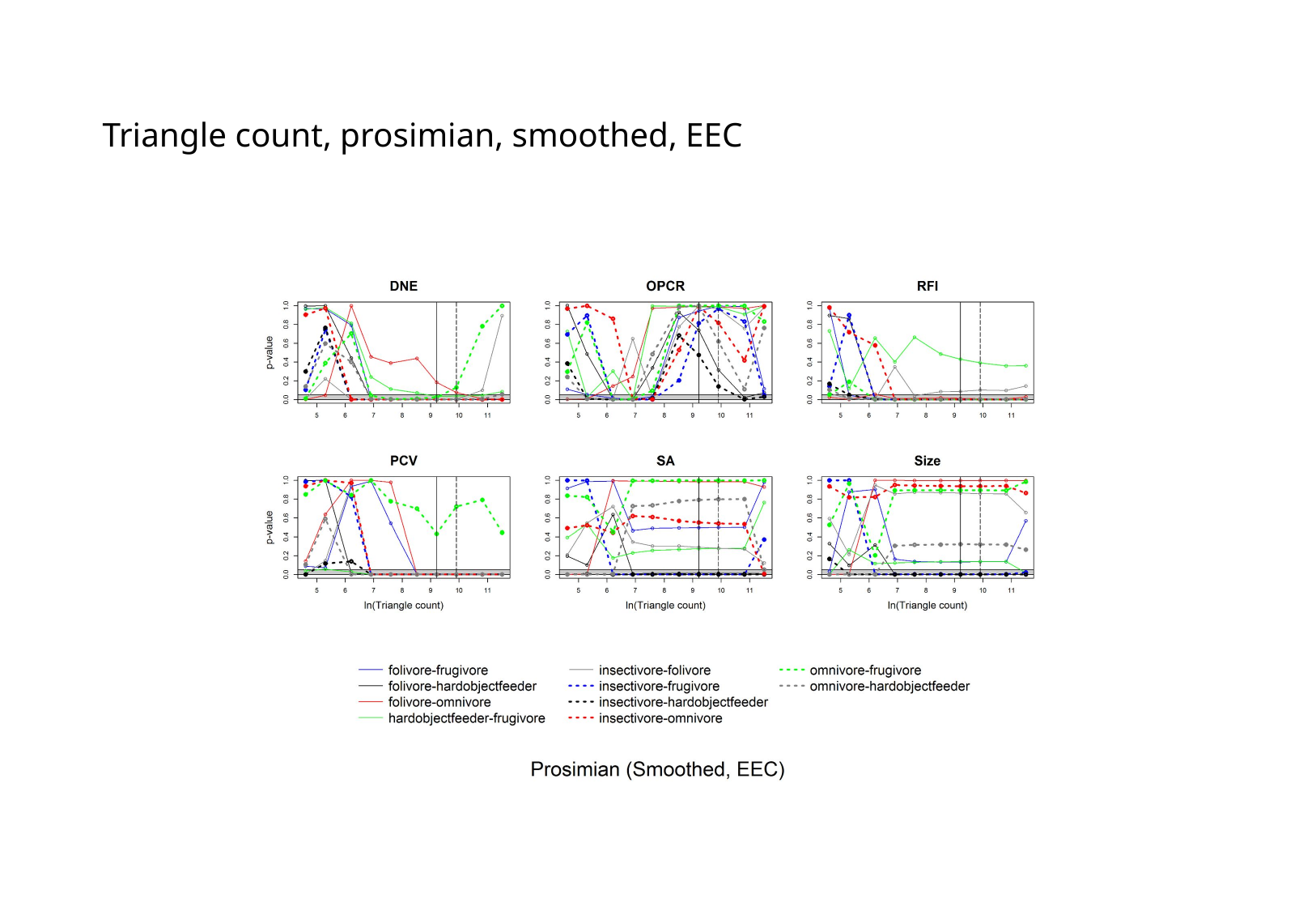

# Triangle count, prosimian, smoothed, EEC

## Slide 3
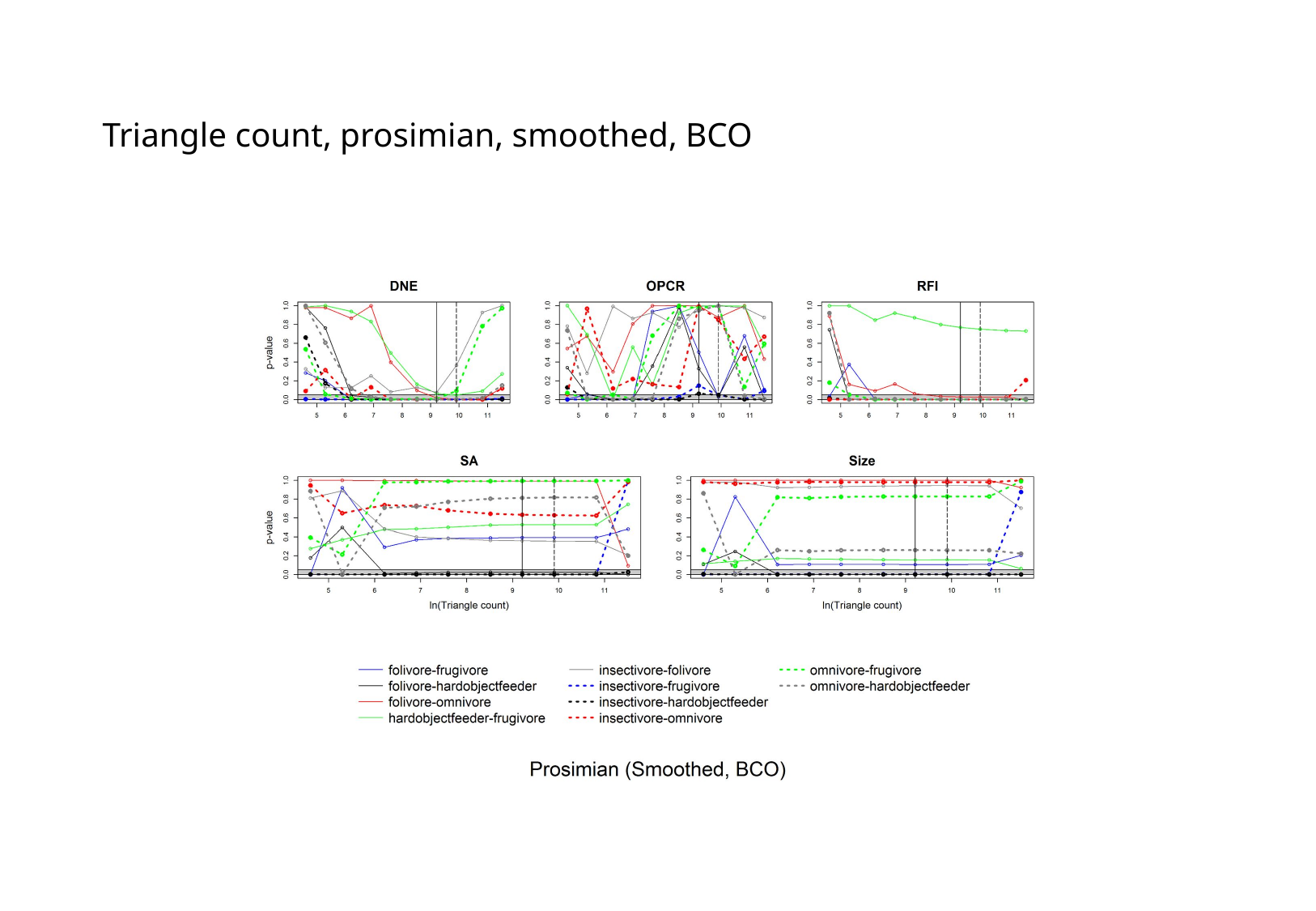

# Triangle count, prosimian, smoothed, BCO

## Slide 4
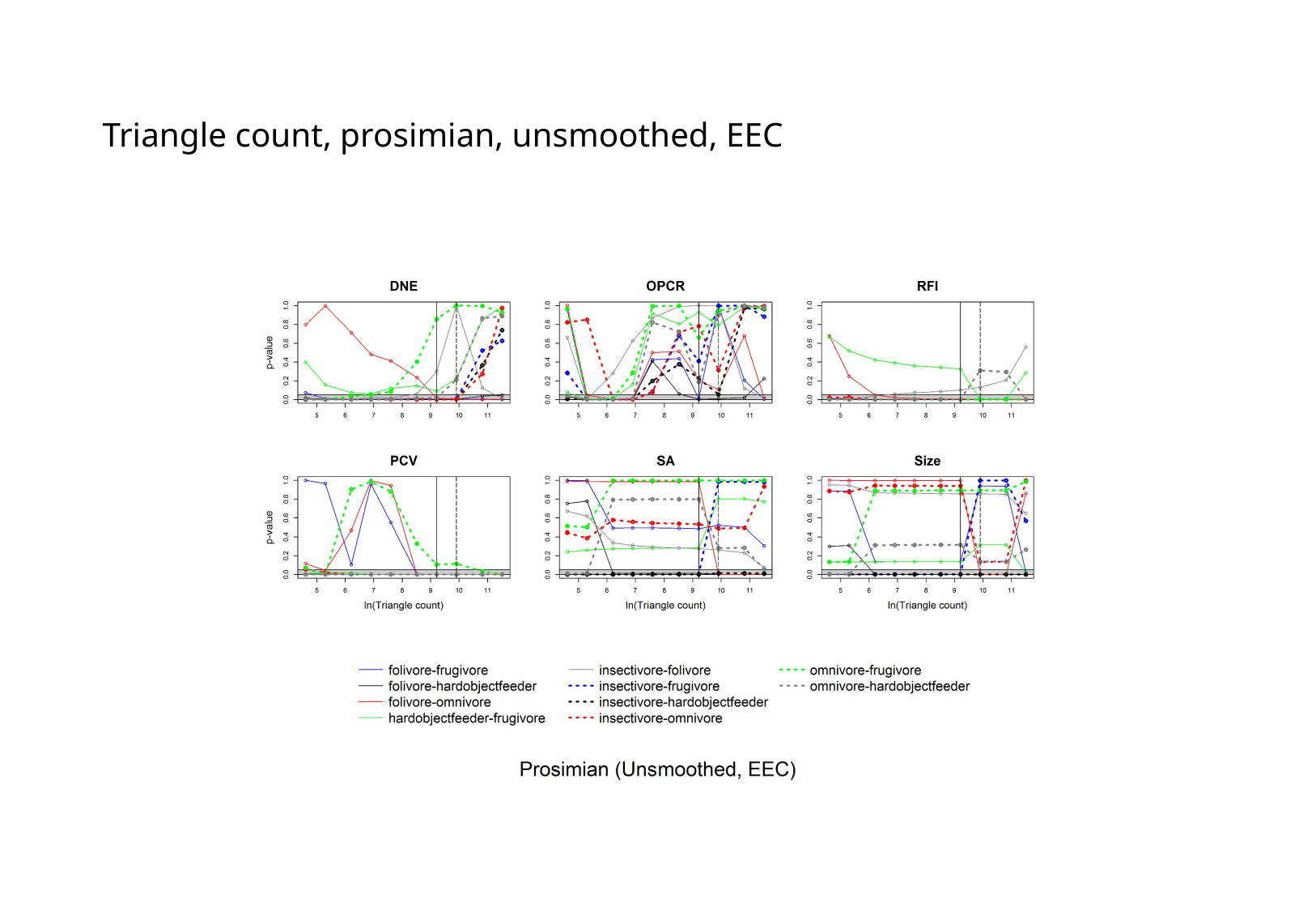

# Triangle count, prosimian, unsmoothed, EEC

## Slide 5
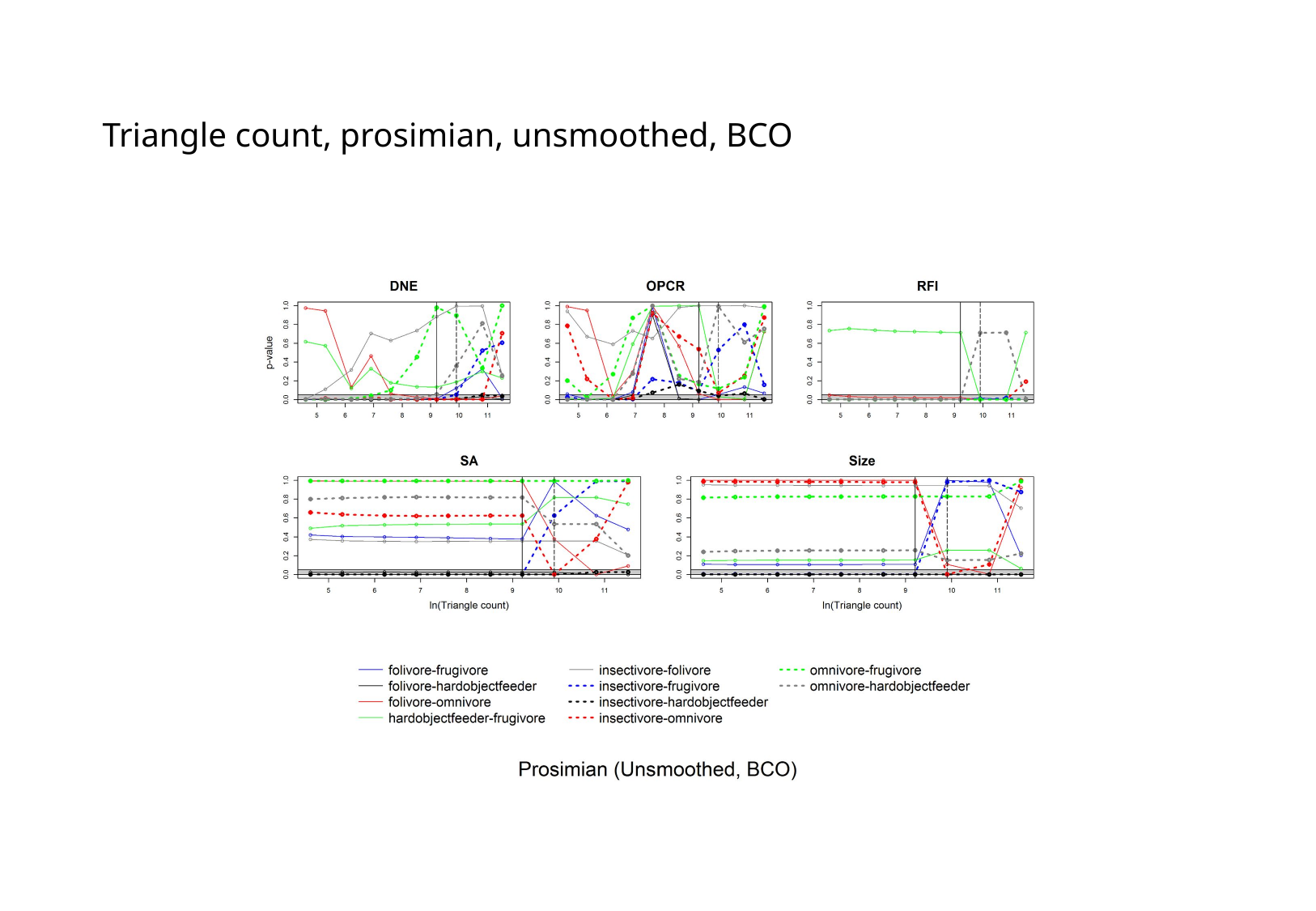

# Triangle count, prosimian, unsmoothed, BCO

## Slide 6
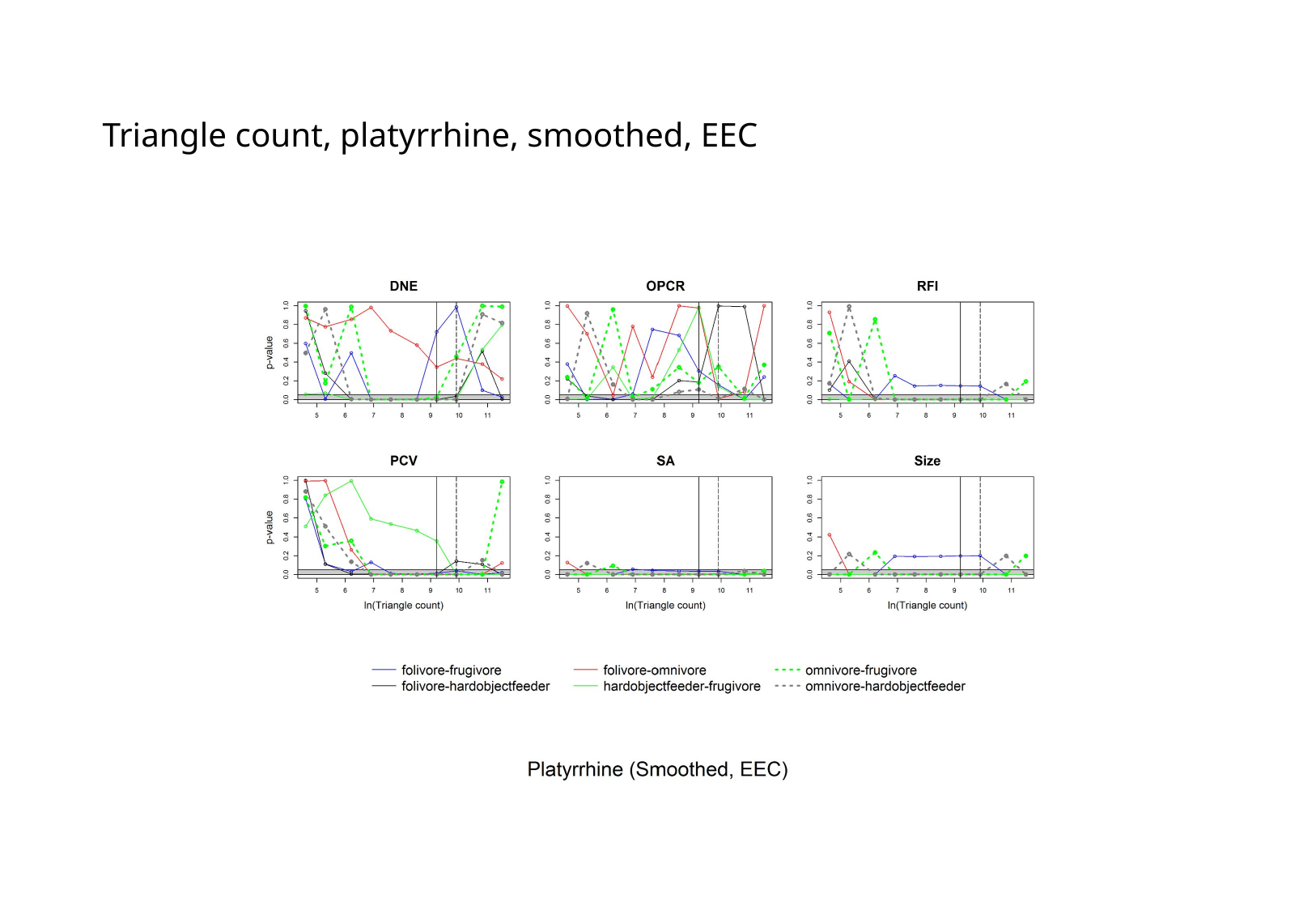

# Triangle count, platyrrhine, smoothed, EEC

## Slide 7
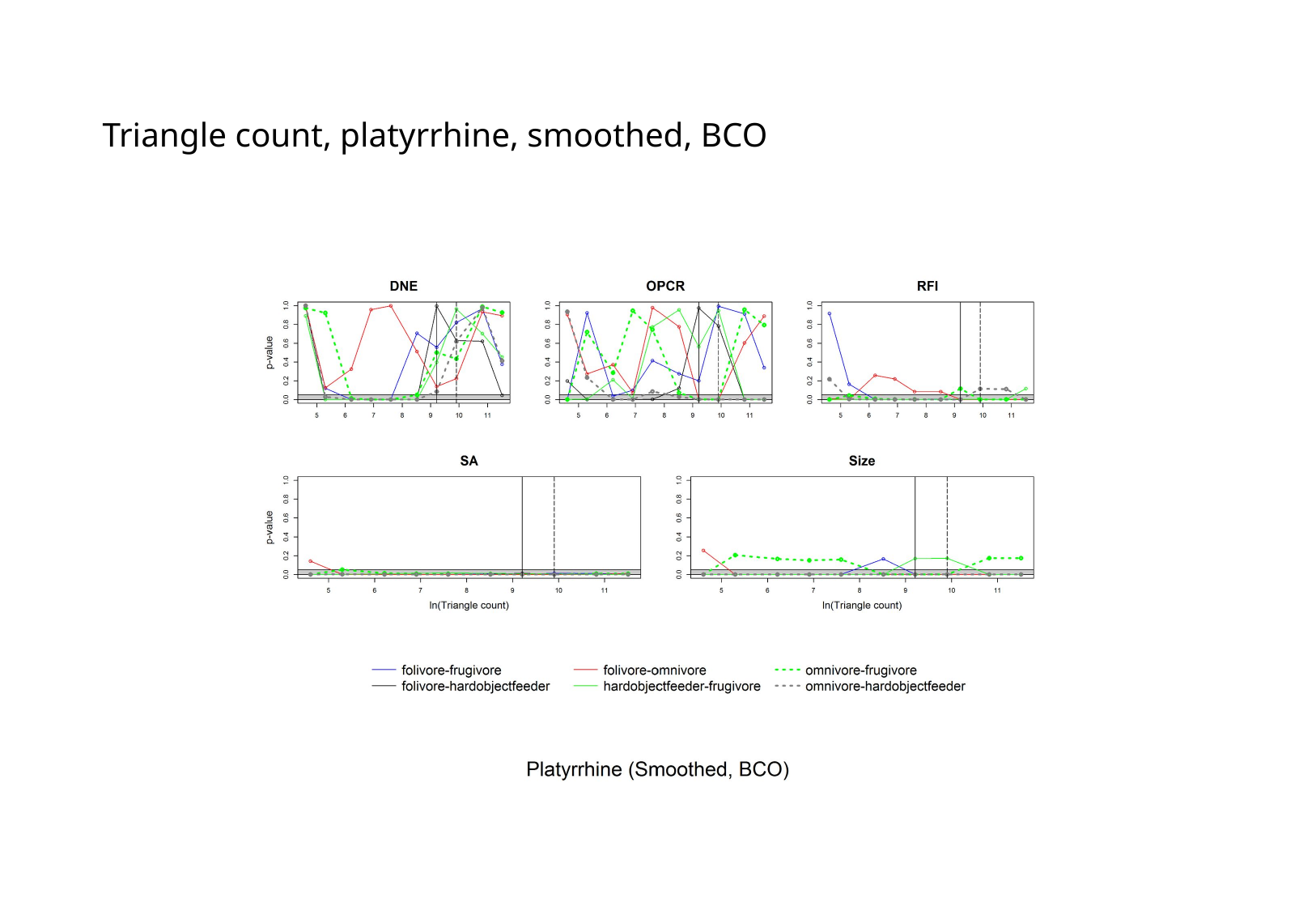

# Triangle count, platyrrhine, smoothed, BCO

## Slide 8
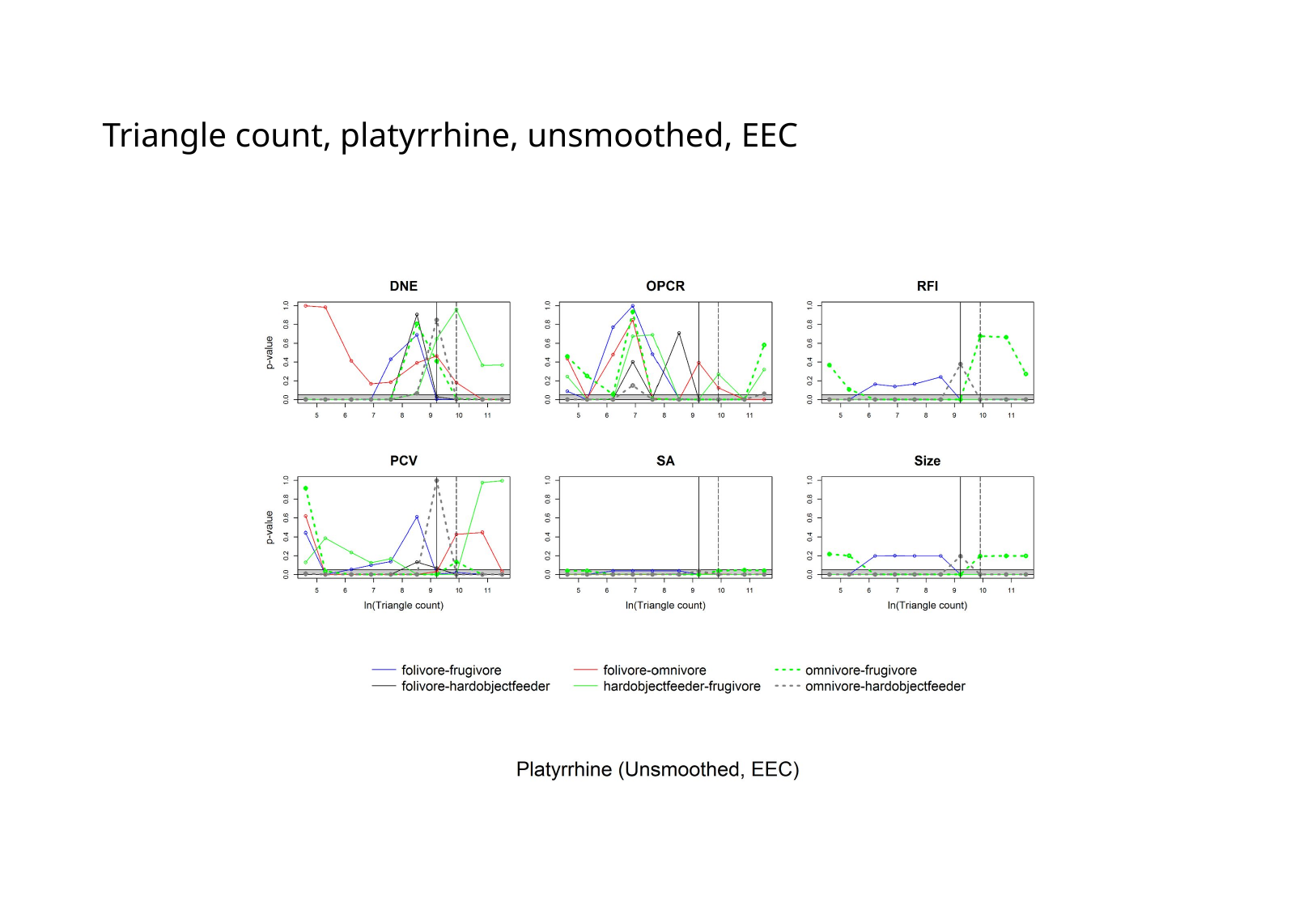

# Triangle count, platyrrhine, unsmoothed, EEC

## Slide 9
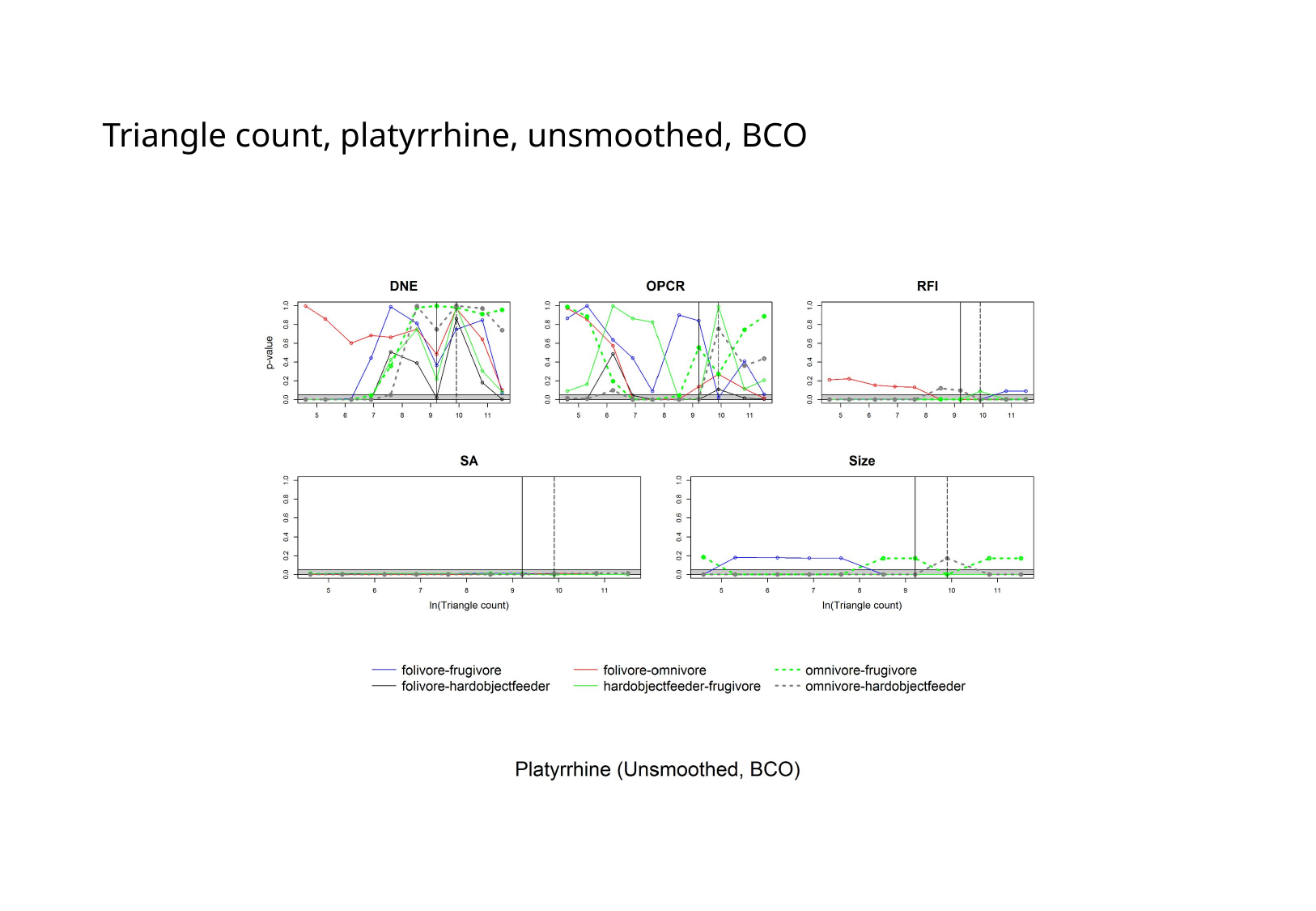

# Triangle count, platyrrhine, unsmoothed, BCO

## Slide 10
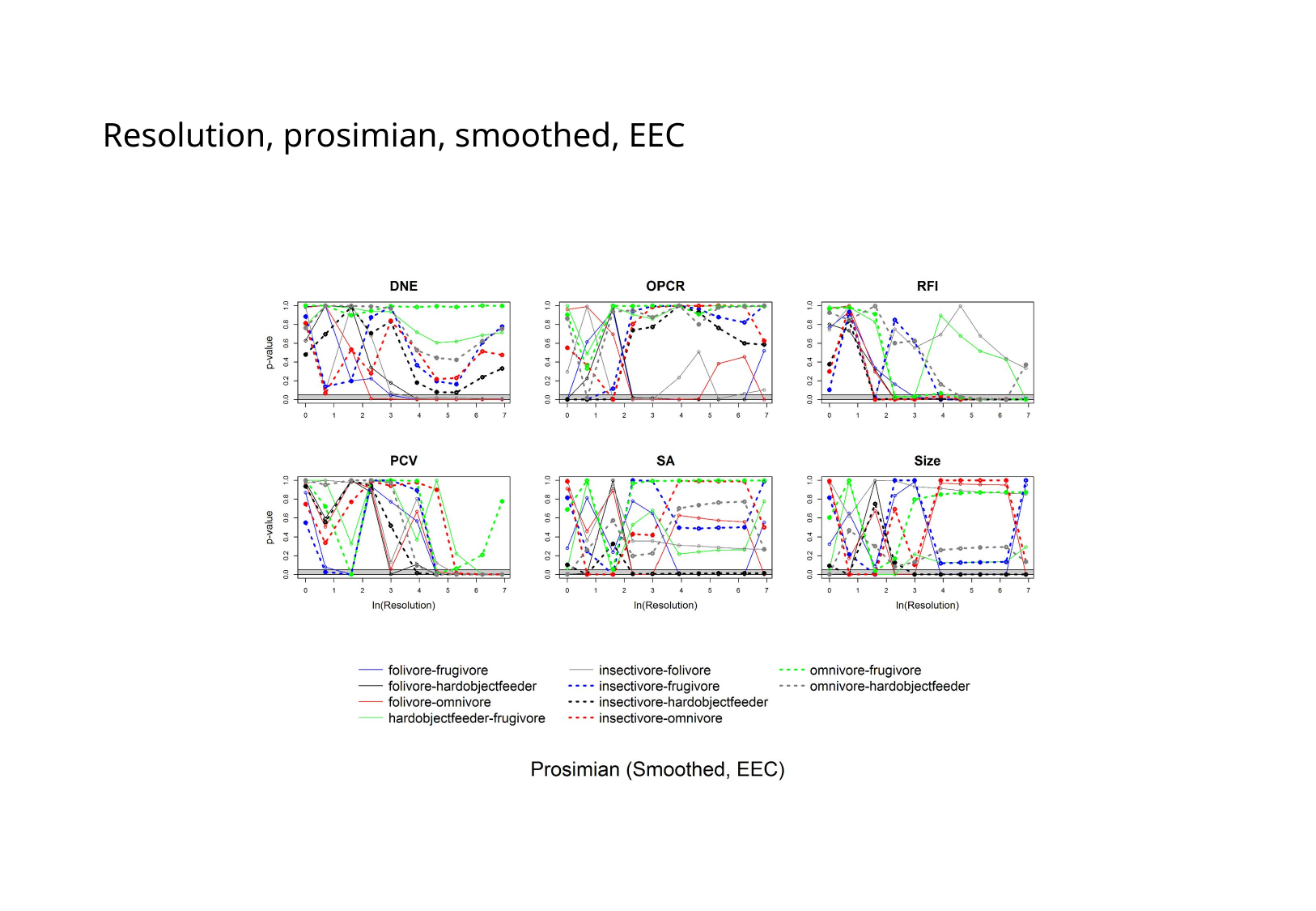

# Resolution, prosimian, smoothed, EEC

## Slide 11
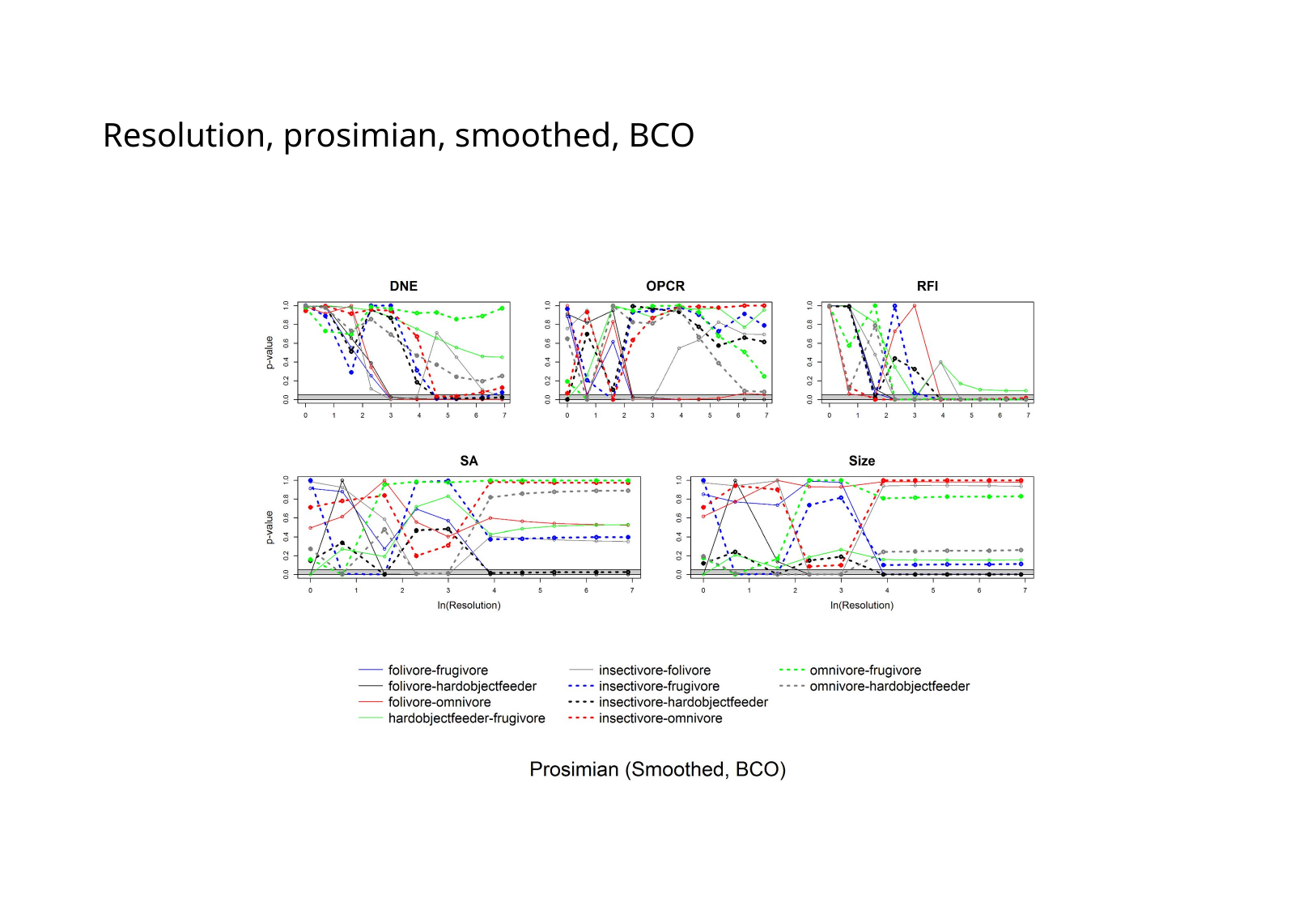

# Resolution, prosimian, smoothed, BCO

## Slide 12
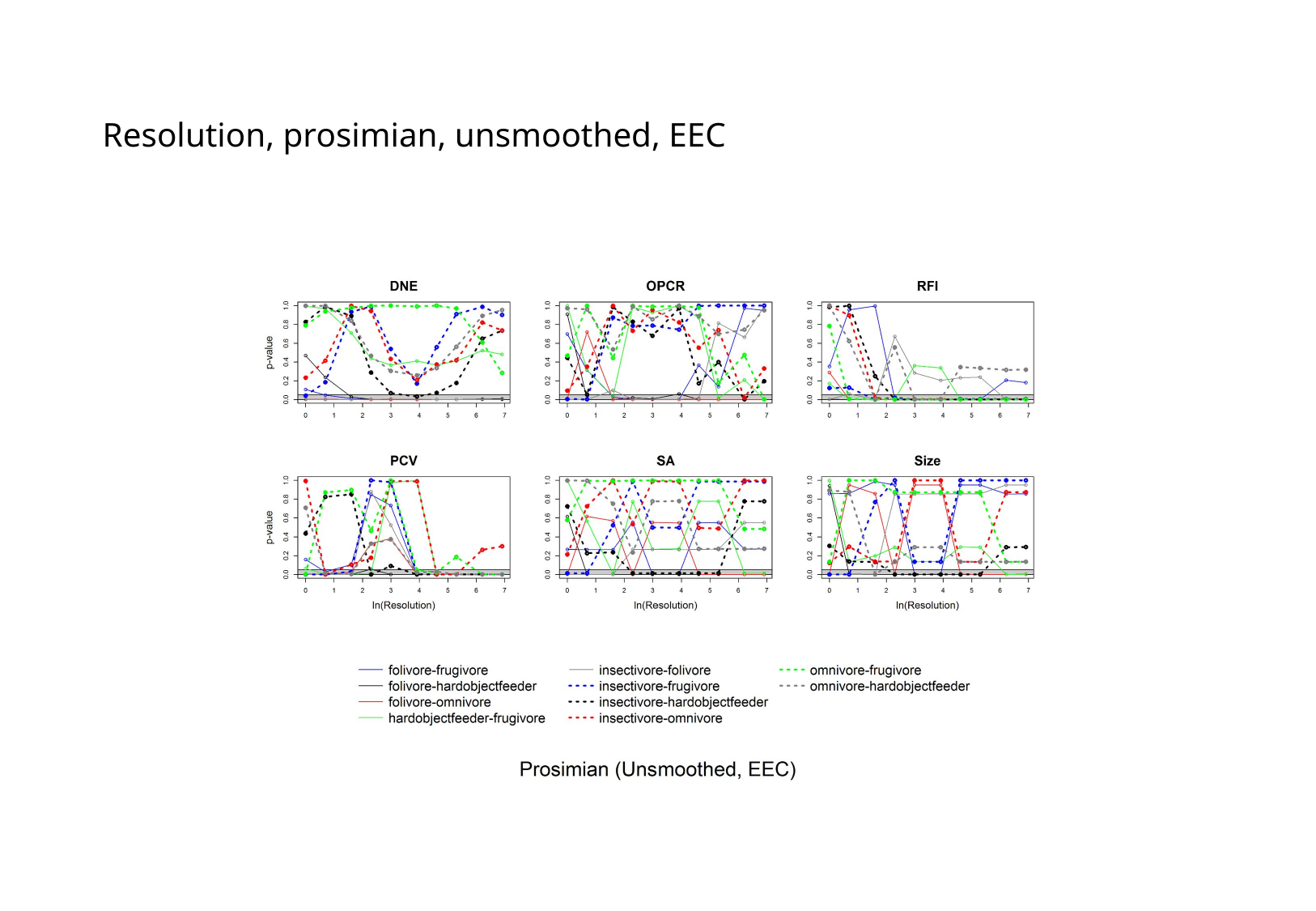

# Resolution, prosimian, unsmoothed, EEC

## Slide 13
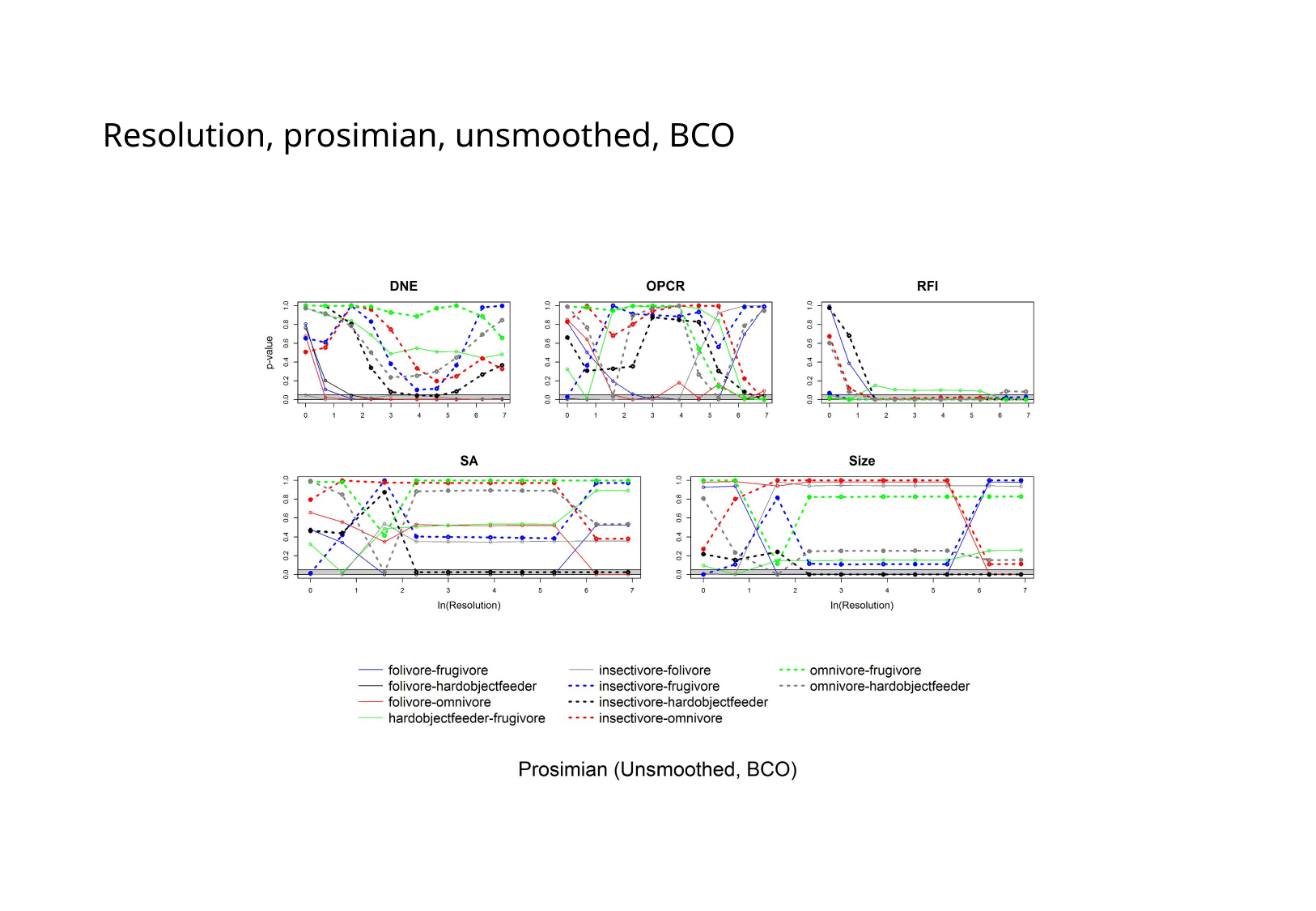

# Resolution, prosimian, unsmoothed, BCO

## Slide 14
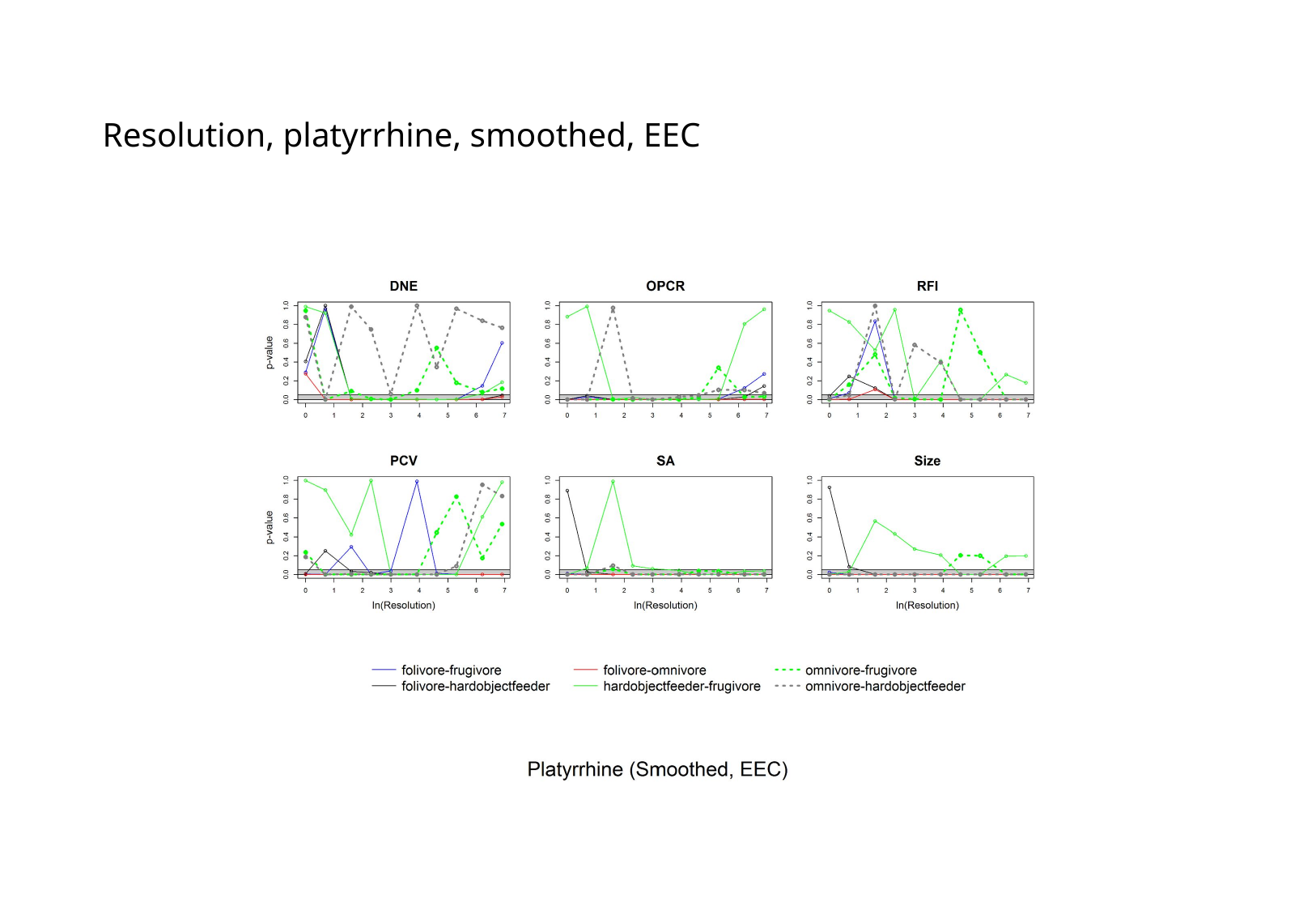

# Resolution, platyrrhine, smoothed, EEC

## Slide 15
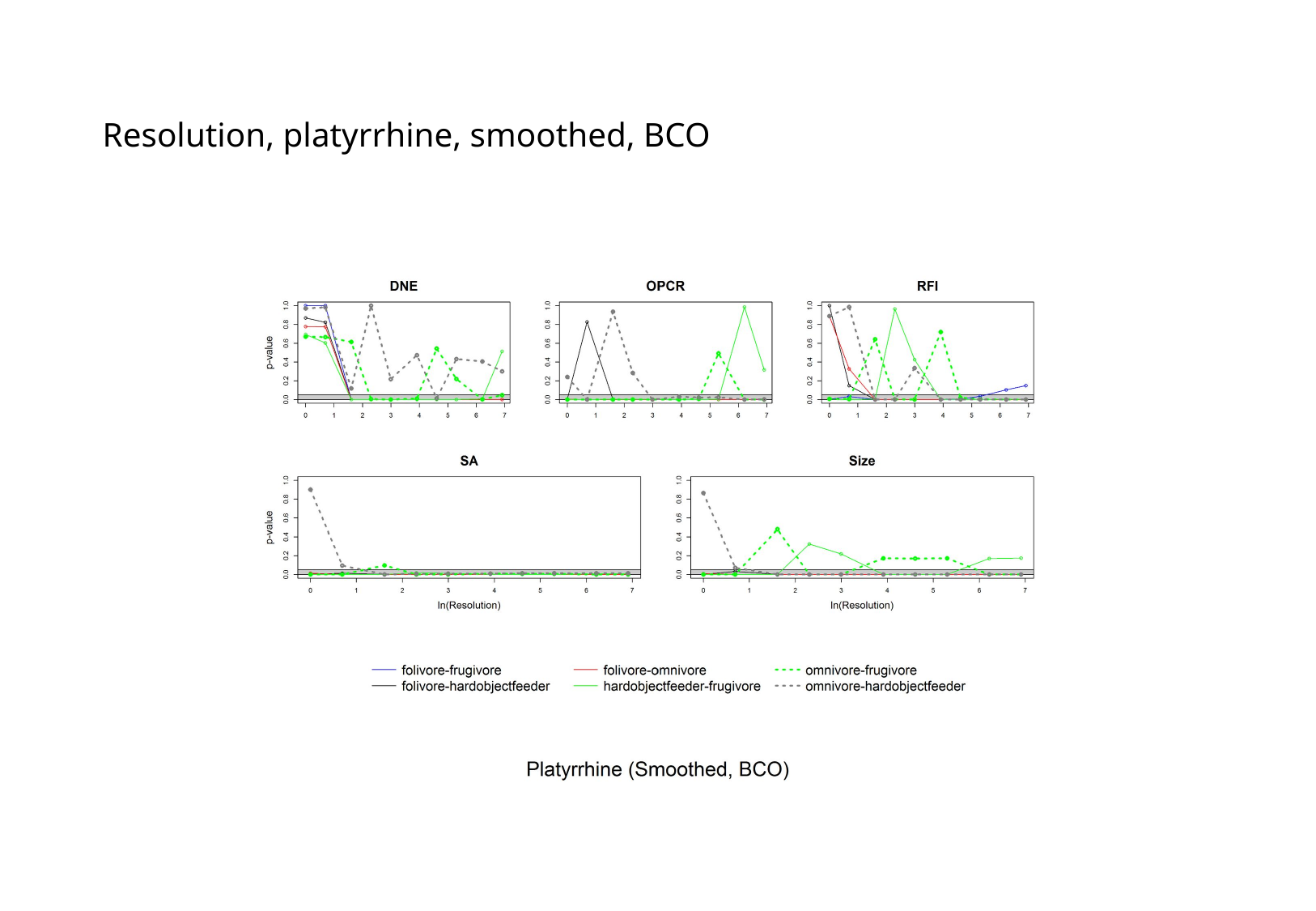

# Resolution, platyrrhine, smoothed, BCO

## Slide 16
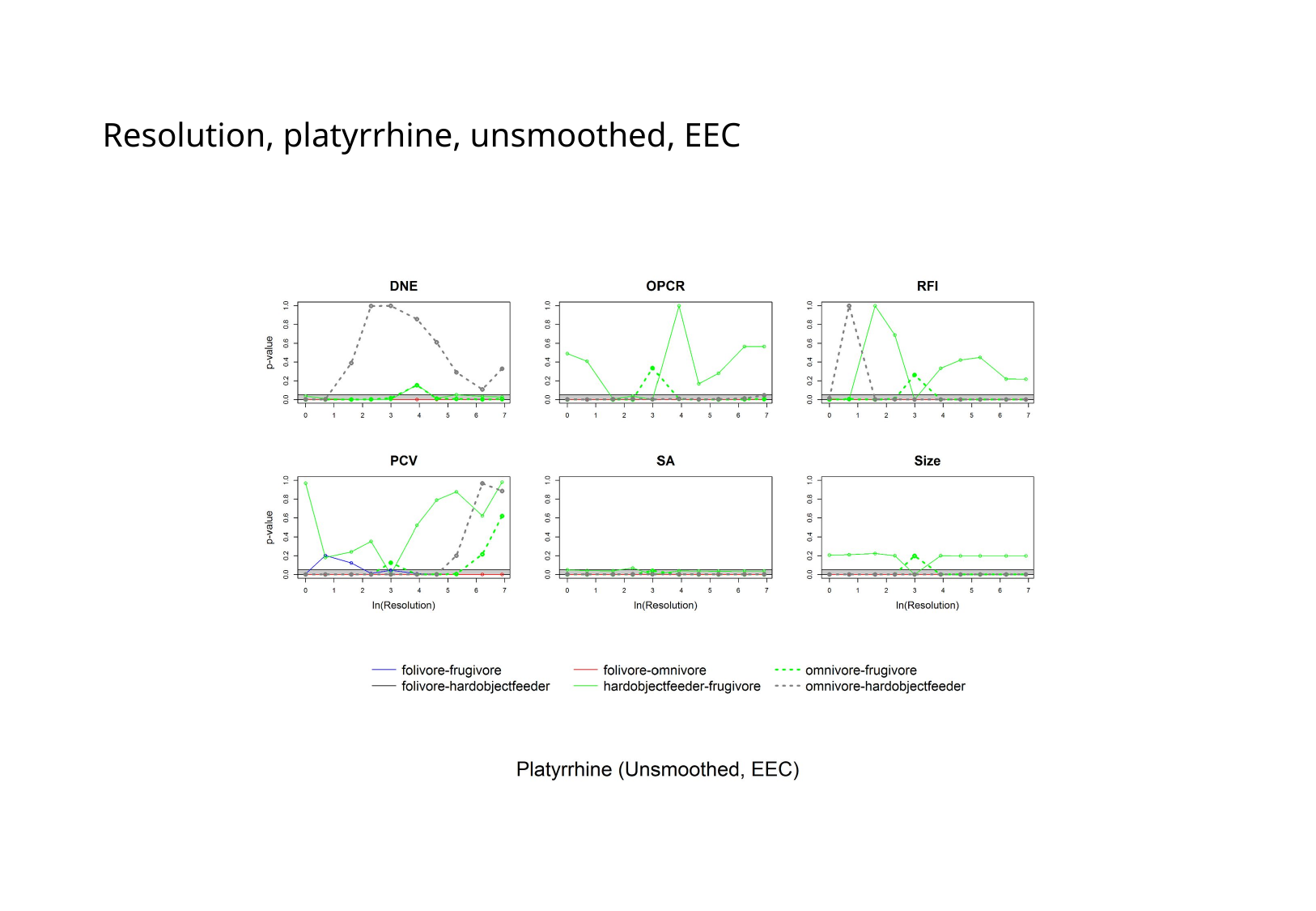

# Resolution, platyrrhine, unsmoothed, EEC

## Slide 17
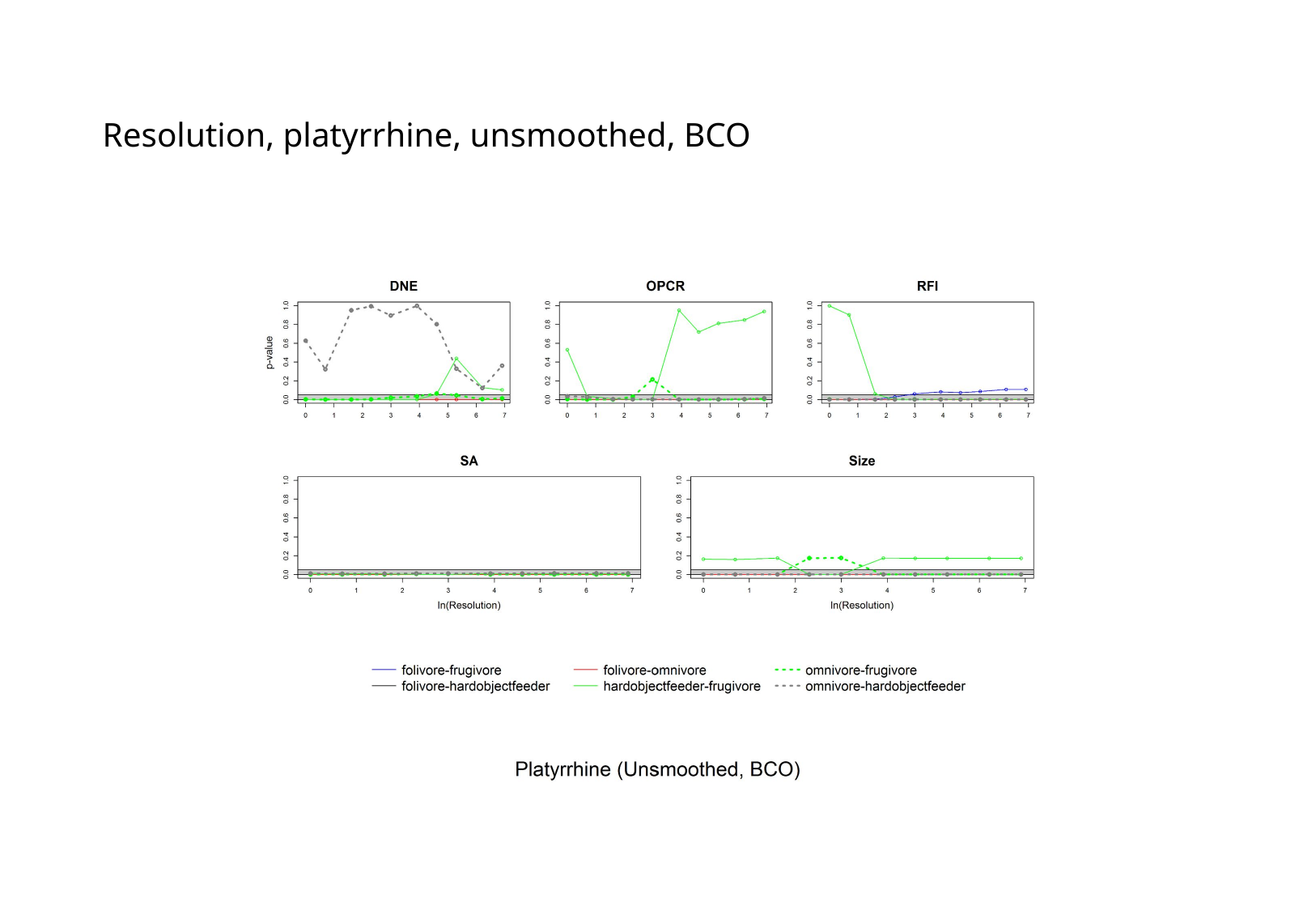

# Resolution, platyrrhine, unsmoothed, BCO
